# Experimental evidence for the adaptive response of aquatic invertebrates to chronic predation risk

**DOI:** 10.1007/s00442-020-04594-z

**Published:** 2020-01-09

**Authors:** Łukasz Jermacz, Anna Nowakowska, Hanna Kletkiewicz, Jarosław Kobak

**Affiliations:** 1 Department of Invertebrate Zoology, Faculty of Biology and Environmental Protection, Nicolaus Copernicus University, Lwowska 1, 87-100 Toruń, Poland; 2 Department of Ecology and Biogeography, Faculty of Biology and Environmental Protection, Nicolaus Copernicus University, Lwowska 1, 87-100 Toruń, Poland; 3 Department of Animal Physiology, Faculty of Biology and Environmental Protection, Nicolaus Copernicus University, Lwowska 1, 87-100 Toruń, Poland

**Keywords:** Fear effects, Stress physiology, Antioxidant defence, Non-consumptive effects, Fight-or-flight response

## Abstract

As acute stress induced by predation risk can generate significant oxidative damage, prey organisms are forced to balance their defence reaction and the cost of activating the cellular defence system. Stress tolerance differs significantly among species; therefore predator pressure indirectly shapes the community structure. To test adaptation abilities of amphipod crustaceans (*Dikerogammarus villosus* and *Gammarus jazdzewskii*) we exposed them to acute (35 min.) and chronic (1 or 7 days) predation risk (the Eurasian perch). We measured respiration (related to metabolic rate), cellular defence systems (antioxidant enzyme (catalase) activity and heat shock protein (Hsp70) concentration), and the level of oxidative damage (thiobarbituric acid reactive substances (TBARS) concentration). Both amphipods increased their respiration rate in the presence of predation cues, irrespective of the duration of their pre-exposure to danger. This increase in *D. villosus* was initiated more quickly (immediately vs. after 10 min. of the test) and lasted for a longer time (20 vs. 10 min.) than in *G. jazdzewskii*. However, only *G. jazdzewskii* after a short exposure to predation risk exhibited an increase in its catalase activity, Hsp70 concentration and oxidative damage. No changes in these parameters were exhibited by *D. villosus* or after a chronic exposure of *G. jazdzewskii* to predation cues. Our results show that prey organisms are able to reconfigure their physiology to maintain increased metabolic rate under prolonged predator pressure and, at the same time, reduce oxidative damage as well as costs related to anti-oxidant defence.

**Electronic supplementary material:**

The online version of this article (10.1007/s00442-020-04594-z) contains supplementary material, which is available to authorized users.

## Introduction

Predator pressure is a crucial evolutionary force, driving a number of adaptations in prey species (Yoshida et al. 2003; Bollache et al. 2006; Engel and Tollrian 2009; Naddafi and Rudstam 2013). In response to predation risk, prey individuals reallocate available energy from growth and reproduction to defence mechanisms, expressed as changes in behaviour, morphology and life history (Creel et al. 2007; Strobbe et al. 2010; Hawlena et al. 2011). Moreover, at the same time prey individuals limit food acquisition, thus intensifying negative non-consumptive predator effects (Jermacz and Kobak 2017; Beermann et al. 2018). Therefore, at a demographic scale, the costs of indirect predator effects are comparable with those resulting from direct predation (Werner and Peacor 2003; Preisser et al. 2005; Zanette et al. 2014; Sheriff et al. 2015), significantly affecting prey mortality (McCauley et al. 2011; Siepielski et al. 2014). Indirect predator effects are strongly associated with prey growth reduction due to modification of feeding behaviour (Werner and Anholt 1993; Jemacz et al. 2017c; Beermann et al. 2018). However, it was experimentally demonstrated that changes in prey physiology could also be responsible for the lower growth (Slos and Stoks 2008; Hawlena and Schmitz 2010; Janssens and Stoks 2013; Jermacz and Kobak 2017) or even death (McCauley et al. 2011) of prey under predation risk.

In the first step after detection of the predator presence, many prey species increase their metabolism rate and relocate resources to improve their vigilance and capability of a fast response (Hawlena and Schmitz 2010). Such adaptations increase the efficiency of an organism, necessary during a defence reaction (Sapolsky 2002). However, the duration of this maximum efficiency period is limited due to the destructive effect of prolonged and increased reactive oxygen species (ROS) synthesis (Monaghan et al. 2009). To reduce the negative effect of ROS activity, prey species are forced to activate cellular defence systems including anti-oxidant enzymes and heat shock proteins. On the one hand, activation of the cellular defence system is energetically costly (Sørensen et al. 2003; De Block and Stoks 2008) and in consequence the prey organism allocates less energy to other activities. On the other hand, non-neutralized ROS generate damage in key biological molecules such as DNA, proteins and lipids (Monaghan et al. 2009) significantly affecting organism functionality. Therefore, chronic predation risk may cause a number of negative changes in prey physiology and/or behaviour and, in consequence, affect its survival (Hawlena and Schmitz 2010; Janssens and Stoks 2014).

A number of compensatory mechanisms limiting negative physiological consequences of defence reactions exist (Thaler et al. 2012; Dalton and Flecker 2014; Van Dievel et al. 2016). However, their effectiveness varies among prey species (Benard 2004). For example the tobacco hornworm, *Manduca sexta*, increase food assimilation to balance the lower consumption rate under predation risk, but the duration of the increased assimilation is limited and related to the alteration of body composition (Thaler et al. 2012). Therefore, in communities with high predator pressure, the difference among species in resistance to negative effects of chronic predation risk may affect species fitness and, in consequence, community composition (Jermacz and Kobak 2017).

Nevertheless, there are still gaps in our knowledge of the ability of prey to function under prolonged predation risk, especially in the context of metabolic rate. Most of the studies report results of only short or only prolonged prey exposure to predation risk, which do not allow time-related modifications of their physiology to be observed. Here we present one of the first studies focused on the abilities of aquatic invertebrates to deal with prolonged predation risk, using metabolic rate, anti-oxidant defence and level of oxidative damage as markers of adaptations to the predation risk. As a model, we used two freshwater amphipod species *Dikerogammarus villosus* and *Gammarus jazdzewskii*, recently distinguished from *G. fossarum* complex (Rudolph et al. 2018), exposed to short and chronic predation risk generated by kairomones of the Eurasian perch *Perca fluviatilis*. The former species is invasive in many parts of Europe, relatively large, less active and armoured with a harder exoskeleton compared to non-invasive, smaller *G. jazdzewskii* (Rewicz et al. 2015; Błońska et al. 2015)*.*

We hypothesized that under acute predation risk prey organisms would increase their metabolic rate to maximize the efficiency of their defence reaction. Moreover, we predicted that in consequence of the higher metabolic rate prey organisms would activate their cellular defence systems. As the increased metabolic rate generates significant costs due to the activation of the anti-oxidant system, we assumed two scenarios of physiological modifications after prolonged predator pressure. In the first scenario, prey organisms would reduce their metabolic rate to avoid oxidative damage as well as costs related to the cellular defence mechanism. In the second scenario, prey organisms would keep their metabolic rate at the increased level and suffer oxidative damage due to exceeding the efficiency of their cellular defence system.

## Material and methods

### Animal collecting and housing

Individuals of *D. villosus* were collected from the nearshore zone of the Włocławek Reservoir (a dam reservoir on the lower River Vistula, Central Poland, N:52°37′03″, E:19°19′37″) using traps made of artificial Christmas tree branches. Individuals of *G. jazdzewskii* were captured in the Zielona Struga Canal (a left tributary of the River Vistula, N:53°00*′*12*"*, E:18°27*′*39*"*) with a hand net. As predators we used the Eurasian perch (*Perca fluviatilis*), which coexists with both gammarid species in their native and invaded ranges (Kottelat and Freyhof 2007, personal observation). Fish were caught by electrofishing (device type EFGI 650, BSE Bretschneider Spezialelektronik, Germany) from the Włocławek Reservoir (the same place as the location of gammarid collection). Collected perch and gammarids were transported in 20-L containers to the laboratory and placed in 200-L stock aquaria (each species separately) for a week for acclimation. The acclimation period is crucial for our study, as it reduces the thermal sensitivity of the oxygen consumption rate and improves the heat tolerance of tested gammarids (Semsar-kazerouni and Verberk 2018). The stock aquaria were equipped with a system of constant water aeration and filtration. The animals were fed once a day, the fish with live gammarids of both species, the gammarids with frozen chironomid larvae. Moreover, the bottom of the aquaria with the gammarids was covered by plant detritus (additional food source) and gravel. Light conditions were natural (14:10-h light/dark), not supported by any artificial lights. To control water temperature (20 °C) we used air conditioning and aquarium heaters with thermostats placed in each tank.

### Experimental setup and test procedure

To check the effect of short and chronic predation risk, we tested 3 groups of gammarids (Online Resource Fig. S1). The first group was taken directly from the stock aquarium and not pre-exposed to the predation risk before the test. Therefore, the response of these individuals can be interpreted as a short-term reaction, occurring immediately after the appearance of a predator in the environment. The next two groups consisted of gammarids pre-exposed to predation risk for one or seven days before measurements, respectively, which allowed us to check the effect of sustained stress conditions on prey responses and to detect potential habituation and/or appearance of negative symptoms of chronic stress. Pre-exposure to the predator presence took place in 40-L tanks (water temperature: 20 °C, as in the stock tanks) equipped with a net cage (12 × 14 × 12 cm) suspended in the water column. The experimental water temperature was suitable for both species, as confirmed by measurements at their collection sites, as well as an experimental test (van der Velde et al. 2009; Semsar-kazerouni and Verberk 2018). Gammarids (20 individuals per cage) were introduced to the cage, which protected them from perch predation (Supplement Fig. S1). The bottom of the cage was covered with gravel (11–15 mm in diameter), which could be used by gammarids as a shelter (Kobak et al. 2014, 2015), and detritus, similar to that in the stock tanks. During the pre-exposure, gammarids were fed daily, as during the acclimation. Gammarids in natural conditions occur at high density (Żytkowicz et al. 2008) and the effectiveness of their defence responses depends on the presence of conspecifics (Jermacz et al. 2017a). Therefore, both during the pre-exposure and during the test procedure gammarids were kept in groups. The individuals were not injured or damaged after the experiment, showing that antagonistic interactions among them were not common. Single fish were placed in the pre-exposure tanks 3 days before the gammarid introduction and fed every day (including the pre-exposure time) with the same gammarid species as that exposed in the cage (10 individuals of *D. villosus* or 20 individuals of *G. jazdzewskii* to account for the difference in gammarid size). Such a procedure guaranteed highly stressful conditions, resulting from the presence of cues emitted directly by predatory fish as well as of alarm cues from injured conspecifics. Simultaneously, we pre-exposed control individuals of each species for the same duration and under the same conditions, but in the absence of fish.

After the pre-exposure period, we placed a single-species group of 10 individuals randomly selected from the same cage in a tightly closed Karlsruhe bottle (250 ml), filled with water containing the predator signal or control water. The pre-exposed gammarids were tested in the water taken from their pre-exposure tanks to avoid any new signals, masking potential habituation effects. Thus, we tested responses of animals exposed to a given signal for a specified time at the end of this exposure. This allowed us to check the effect of exposure duration, which was the primary goal of our study. In the case of gammarids not pre-exposed to the predator presence, the predator signal was prepared by incubation of a single fish for 3 days in a 40-L tank under the same conditions as those used during the pre-exposure of the other gammarid groups (Jermacz et al. 2017b). A similar fishless tank was used as a source of water for the control treatments with gammarids not pre-exposed to predators. We placed five pieces of gravel (15 ± 3.2 mm) in the test bottle to provide shelters for the gammarids.

Altogether, we had 12 different experimental groups: 3 durations of pre-exposure to predators (none, 1 day, 7 days) × 2 gammarid species × 2 treatments (the predation cue present or absent during the test). The tests (7–21 replicates per each treatment) lasted for 35 min, including an initial 5-min. period of acclimation. Gammarid oxygen consumption was measured during the subsequent 30 min. of the test. After the test, gammarids were gently removed from the bottle, weighed to the nearest 0.1 mg with Radwag AS 110/C/2 laboratory scales (Radom, Poland), homogenized (see below) and frozen (− 80 °C) until the determination of physiological markers.

### Response variables

To determine the effects of predation risk on gammarid metabolism rate, we measured their oxygen consumption corrected for weight and locomotory activity (time spent in movement). We estimated the strength of gammarid defence against oxidative stress by measuring the activity of the key antioxidant enzyme—catalase (CAT) (Monaghan et al. 2009). We also evaluated the heat shock protein 70 (Hsp 70) level, which is often increased in stress conditions (Sørensen et al. 2003), including predator presence (Slos and Stoks 2008). Moreover, to determine the level of oxidative stress (i.e. oxidative damage at the cellular level), we measured thiobarbituric acid reactive substances (TBARS) concentration, a commonly used marker of lipid peroxidation (Rice-Evans et al. 1991).

Oxygen concentration in the bottle was measured four times during the test: immediately after the acclimation period and after a subsequent 10, 20 and 30 min. of exposure, using an optical oxygen sensor (FDO 925, WTW, Germany) connected with a multiparameter benchtop meter (inoLab Multi 9620 IDS, Germany). Gammarid respiration during the three 10-min. periods of exposure was calculated as the reduction in oxygen concentration in the bottle. Preliminary tests conducted in the absence of gammarids showed that the background reduction in oxygen concentration (caused, for example, by bacteria present in experimental water) was negligible and did not differ among the treatments. To control for the effect of gammarid activity (percentage of time spent in moving) on oxygen consumption, we simultaneously video-recorded their behaviour with an IP video camera (SNB-6004, Samsung, South Korea) placed vertically under the bottle. Then, we measured the percentage of time spent by gammarids in movement (average value of 10 individuals in the bottle) in three 10-min. periods corresponding to the above-mentioned measurements of oxygen concentration, using Noldus Ethovision XT 10.1 (Noldus Information Technology Inc., Leesburg, Netherlands) video analysis software. Dividing the measurement into three 10-min. periods allowed us to determine the duration of the acute gammarid response, as well as to check for any potential changes in this response with time.

Biochemical parameters (CAT activity, TBARS and Hsp70 concentration) were measured after a 35-min. exposure of gammarids to experimental conditions. Extracts for biochemical analyses were prepared from pooled specimens (samples of 250–500 mg) to obtain a sufficient amount of material. Live gammarids were homogenized in 6 ml of potassium phosphate buffer at pH 7.4 using a Potter homogenizer with a Teflon piston (200 rotations per minute) at 10 °C. After centrifugation at 12,000 g for 10 min at 4 °C, the supernatants were collected in Eppendorf tubes and stored at − 80 °C until analysis. Then, the samples were used for determination of CAT activity, as well as Hsp70 and TBARS concentration.

CAT activity was determined according to Bartosz (2008) by monitoring decomposition of 54 mM H_2_O_2_ in 50 mM phosphate buffer (pH 7.0) in a total volume of 3 ml. For each sample, 20 µL of homogenate was added to start the reaction and the decrease in absorbance during 3 min. at an ambient temperature of 25 °C was measured at 240 nm with a spectrophotometer (Schimadzu UV-1800 spectrophotometer, Shimadzu Inc., Kyoto, Japan). The enzyme activity was expressed as U mg protein^−1^. The unit of CAT activity is defined as the reduction of 1 µmol of the peroxide per minute. To relate the result to unit protein mass, the protein concentration was measured by the Folin-Phenol method described by Lowry et al. (1951). Bovine serum albumin (Sigma Chemical, Steinheim, Germany) was used as a standard.

The concentration of Hsp 70 was determined by a commercial sandwich Elisa kit from Biorbyt (UK catalogue number orb397140) according to the manufacturer’s instruction. Colorimetric changes in the assay were detected using a multi-mode microplate reader Epoch 2 (BioTek Instruments, Inc., Winooski, UT, USA) and data were analysed using BioTek GEN 5 software. The sensitivity of the Hsp 70 kit was less than 80 pg ml^−1^.

TBARS concentration was determined according to Rice-Evans, Diplock and Symons (1991). For each sample, 1 ml of homogenate was added to 1 ml of 15% TCA (w/v) and 0.37% (w/v) TBA in 25 mM HCl. The samples were heated for 10 min in a boiling water bath to release MDA (the end product of lipid peroxidation) from proteins, then quickly cooled to avoid adsorption of MDA to insoluble proteins and immediately centrifuged for 5 min at 6500 g. In parallel, two blank samples were prepared: one without TBA and the other without the homogenate. All samples were subjected to spectrophotometric analysis at 535 nm. TBARS concentration was calculated using the molar extinction coefficient for MDA-TBA complex of 1.56 × 10^5^ M^–1^ cm^–1^ and expressed as µmol g wet mass^−1^.

### Statistical analyses

To analyse factors affecting oxygen consumption by gammarids (adjusted for their biomass and activity), we applied a General Linear Model (GLM) with predator presence, gammarid species and pre-exposure time as fixed categorical factors, as well as gammarid biomass and activity as continuous covariates. We log-transformed the variables for this analysis to linearize a potentially allometric relationship between biomass and metabolism. We carried out separate analyses for each 10-min. period of exposure (0–10, 11–20 and 21–30 min.). To analyse the effect of the predator presence, pre-exposure duration and species on biochemical markers (measured at the end of the test period), we applied a 3-way General Linear Model with predator presence, gammarid species and pre-exposure time as fixed factors.

As the pre-exposure time in itself could not explain gammarid behaviour adequately (they were pre-exposed to predation cues or control water), we included this variable in the models only in its interactions with the predation cue. This allowed us to check whether physiological traits of gammarids depended on their species and predation pressure (past or present), as well as whether their responses to predators differed between species and pre-exposure conditions. If needed, significant terms were further explored using sequential Bonferroni corrected LSD post hoc tests. We used SPSS Statistics 25.0 to conduct statistical analyses.

## Results

**Fig. 1:**
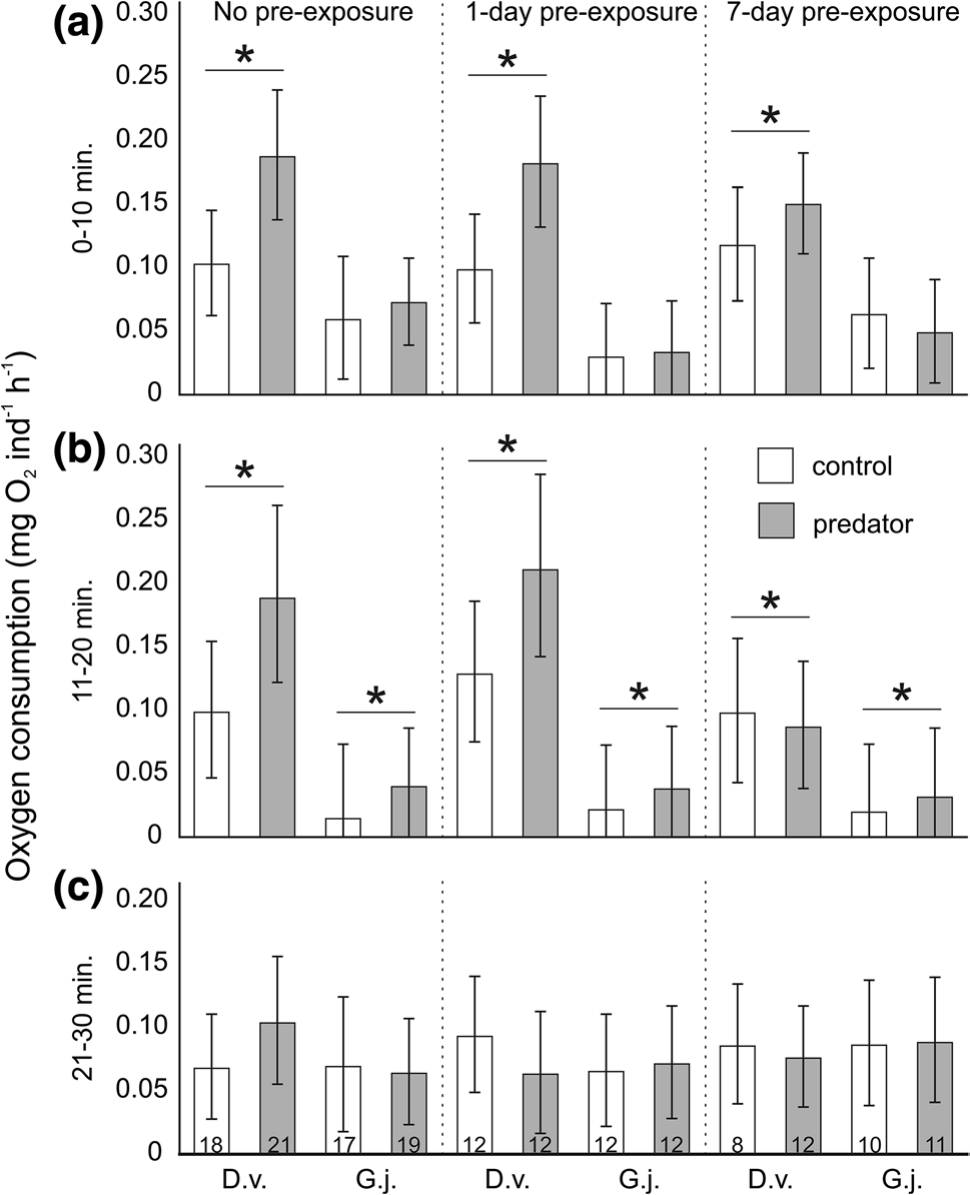
Oxygen consumption by *Dikerogammarus villosus* (D.v.) and *Gammarus jazdzewskii* (G.j.) not pre-exposed, pre-exposed for 1 day or 7 days to the predation cue (grey bars) or control water (white bars) during consecutive periods of the test: (**a**) 0–10 min., (**b**) 11–20 min., (**c**) 21–30 min. The presented values are least square means (back-transformed after the analysis of log-transformed data) predicted by the General Linear Model for average gammarid biomass and activity during the respective test period. Asterisks indicate significantly higher or lower oxygen consumption rate by predator-stressed gammarids compared to corresponding control individuals. Error bars show 95% confidence intervals, numbers on the bars indicate sample sizes (the same for all panels)

Respiration rate of gammarids (adjusted for their activity and biomass) during the first 10 min. of the test depended on an interaction between gammarid species and predator presence (Table 1a). During this period, only *D. villosus* responded to the predation cue, doubling its oxygen consumption compared to the control treatment (Fig. 1a). During the period from 11 to 20 min. of the test, oxygen consumption depended on gammarid species and predator presence, but not on the interaction between these factors (Table 1b). During this period, both species exhibited a higher oxygen consumption under predation cue than under control conditions and *D. villosus* showed a higher oxygen consumption than *G. jazdzewskii* (Fig. 1b). During the last period (21–30 min.) of the test we did not note any significant effects of predation risk or gammarid species on oxygen consumption of gammarids (Table 1c, Fig. 1c). Moreover, during the entire exposure period, gammarid respiration did not depend significantly on their pre-exposure conditions (Table 1), though a non-significant tendency to reduce the effect of predators on *D. villosus* pre-exposed to the predation cue for 7 days was visible (Fig. 1a, b).

**Table 1:** The General Linear Model to test the effect of the gammarid species, predator presence, pre-exposure time, gammarid mass and gammarid activity on the oxygen consumption during the three exposure periods (1–10, 11–20 and 21–30 min.)

	Analysed period	Effect		*df*	MS	*F*	*P*
A	1–10 min	Experimental factors	Species (S)	1	0.059	9.200	0.003
			Predator (P)	1	0.071	10.976	0.001
			S × P	1	0.070	10.943	0.001
			P x Pre-exposure	4	0.010	1.615	0.174
			S × P × Pre-Exposure	4	0.005	0.744	0.563
		Covariates	Mass	1	0.010	1.479	0.226
			Activity	1	0.001	0.161	0.689
		Error		146	0.006		
B	11–20 min	Experimental factors	Species (S)	1	0.087	8.573	0.004
			Predator (P)	1	0.082	8.066	0.005
			S × P	1	0.016	1.568	0.213
			P × Pre-exposure	4	0.017	1.706	0.152
			S × P × Pre-Exposure	4	0.016	1.585	0.181
		Covariates	Mass	1	0.004	0.416	0.520
			Activity	1	0.012	1.202	0.275
		Error		142	0.01		
C	21–30 min	Experimental factors	Species (S)	1	0.000	0.063	0.803
			Predator (P)	1	0.000	0.000	0.988
			S × P	1	0.000	0.018	0.893
			P × Pre-exposure	4	0.004	0.566	0.687
			S × P × Pre-Exposure	4	0.008	1.091	0.363
		Covariates	Mass	1	0.048	6.347	0.013
			Activity	1	0.017	2.258	0.135
		Error		146	0.008

**Fig. 2:**
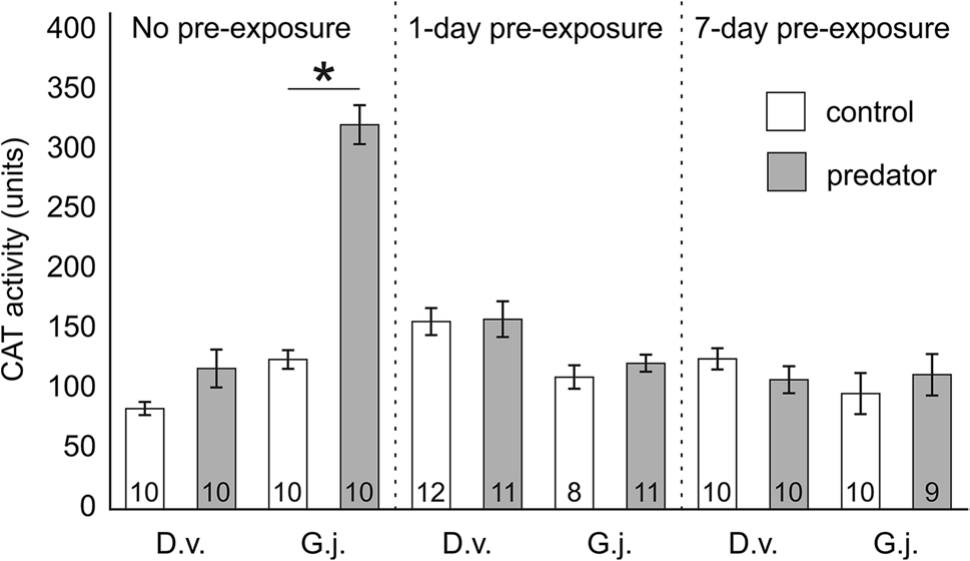
Catalase (CAT) activity in *Dikerogammarus villosus* (D.v.) and *Gammarus jazdzewskii* (G.j.) not pre-exposed, pre-exposed for 1 day or 7 days to the predation cue (grey bars) or control water (white bars). Asterisks indicate significantly higher enzyme activity in predator-stressed gammarids compared to corresponding control individuals. Error bars are standard errors of the mean, numbers on the bars indicate sample sizes

**Fig. 3:**
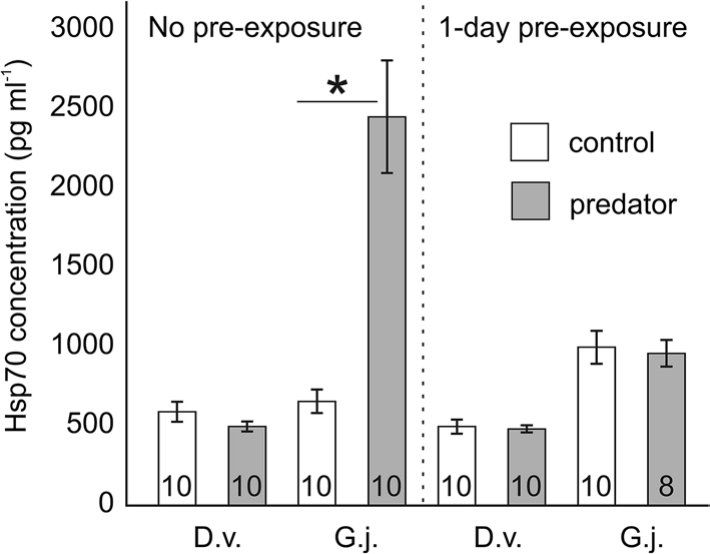
Hsp 70 concentration in *Dikerogammarus villosus* (D.v.) and *Gammarus jazdzewskii* (G.j.) not pre-exposed or pre-exposed for 1 day to the predation cue (grey bars) or control water (white bars). Asterisks indicate significantly higher Hsp 70 concentration in predator-stressed gammarids compared to corresponding control individuals. Error bars are standard errors of the mean, numbers on the bars indicate sample sizes

**Fig. 4:**
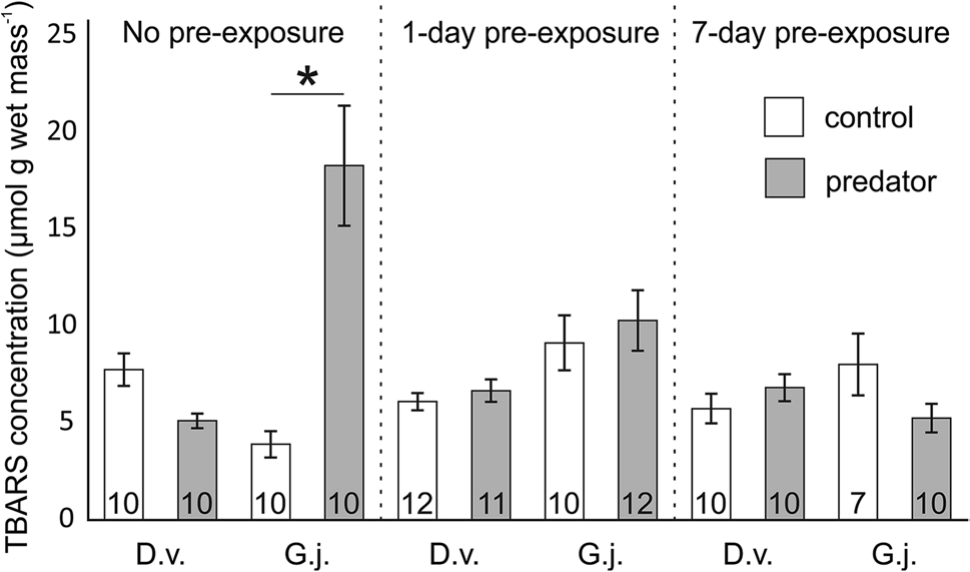
TBARS concentration in *Dikerogammarus villosus* (D.v.) and *Gammarus jazdzewskii* (G.j.) not pre-exposed, pre-exposed for 1 day or 7 days to the predation cue (grey bars) or control water (white bars). Asterisks indicate significantly higher TBARS concentration in predator-stressed gammarids compared to corresponding control individuals. Error bars are standard errors of the mean, numbers on the bars indicate sample sizes

The CAT activity as well as TBARS and Hsp70 concentrations depended on gammarid species, predator presence and pre-exposure time, as shown by significant interactions among these factors in the GLMs (Table 2a, b, c). After exposure to the predation cue, non-pre-exposed individuals of *G. jazdzewskii* exhibited about 150% higher CAT activity (Fig. 2), 270% higher level of Hsp70 protein (Fig. 3) and almost 370% higher lipid peroxidation (Fig. 4) compared to the respective control treatment. Individuals of *G. jazdzewskii* pre-exposed to predation risk did not respond to the predation cue with changes in any of the above-mentioned biomarkers. Regardless of pre-exposure time, individuals of *D. villosus* exposed to the predation cue did not demonstrate any significant changes in any measured biomarkers compared to the respective control treatments.

**Table 2:** The 3-way General Linear Model to test the effect of the gammarid species, predator presence and pre-exposure time on the catalase activity (CAT) (A), heat shock protein (Hsp70) concentration (B) and concentration of thiobarbituric acid reactive substances (TBARS) (C)

	Dependent variable	Effect	*df*	*MS*	*F*	*P*
A	CAT activity	Species (S)	1	15,634.2	9.5	0.003
		Predator (P)	1	48,641.6	29.7	< 0.001
		S × P	1	35,257.5	21.5	< 0.001
		P × pre-exposure	4	33,645.9	20.5	< 0.001
		S × P × pre-exposure	4	47,673.8	29.1	< 0.001
		Error	109	1638.7		
B	HSP 70 level	Species (S)	1	10,893,704.1	54.8	< 0.001
		Predator (P)	1	3,300,175.0	16.6	< 0.001
		S × P	1	4,220,609.5	21.2	< 0.001
		P × pre-exposure	2	2,754,842.7	13.9	< 0.001
		S × P × pre-exposure	2	2,805,239.9	14.1	< 0.001
		Error	70	198,657.4		
C	TBARS level	Species (S)	1	231.6	13.8	< 0.001
		Predator (P)	1	115.5	6.9	0.010
		S × P	1	157.8	9.4	0.003
		P × pre-exposure	4	89.1	5.3	0.001
		S × P × pre-exposure	4	176.0	10.5	< 0.001
		Error	110	16.8

## Discussion

Predator presence induced significant modifications in the physiology of both gammarid species. However, the quality, strength and consequences of these responses differed between the tested species and depended on the time spent by gammarids in the presence of the predator. Under predation risk, both gammarid species significantly boosted their oxygen consumption rates, independent of their activity and pre-exposure time. However, the timing of the increased oxygen consumption under predation risk was different for both species. *Dikerogammarus villosus* consumed more oxygen during the first and second period (0–20 min. after the start of the exposure) while *G. jazdzewskii* exhibited higher oxygen consumption only during the second period (10–20 min. after the start of the exposure). During the last period (20–30 min.), both prey species reduced their oxygen consumption to the level presented by non-stressed individuals. Increased oxygen consumption indicates increased metabolism, resulting in higher muscle efficiency. This is a common modification of prey physiology induced by predation risk (Rovero et al. 2000; Woodley and Peterson 2003; Slos and Stoks 2008). However, apart from its beneficial effects on prey survival, such a response also has negative physiological consequences for prey fitness due to the increased synthesis of ROS (De Block and Stoks 2008; Monaghan et al. 2009). The excess ROS influence key biological molecules, such as lipids, proteins and DNA (Hulbert et al. 2007; Monaghan et al. 2009), which impairs many aspects of organism functionality. For example, ROS may induce 10,000 DNA base modifications per cell per day (Ames 1991), generating negative phenotypic consequences (Falnes et al. 2007). ROS activity is also responsible for damage of lipids which are particularly important for membrane structure and function (Hulbert et al. 2007). Finally, ROS generate protein damage responsible for the decrease in muscle efficiency and reduction in defence capabilities (Janssens and Stoks 2014). Thus, the duration of a period of increased metabolic rate is limited and an organism is forced to mobilize its antioxidant defence to reduce the negative consequences of ROS activity. Therefore, in our study, gammarids exposed to predation cues exhibited only short-time events of increased metabolic rate, which returned to its control value at the end of the measurement period.

An initial contact with the predator cue caused an increase in the activity of an enzymatic antioxidant (CAT) only in *G. jazdzewskii*. Previous studies demonstrated that long-term (chronic) predation risk can both increase (Slos et al. 2009) or reduce (Slos and Stoks 2008; Janssens and Stoks 2013) the activity of the antioxidant system. Here we demonstrated that the short-term (35 min) predation risk caused activation of antioxidant defence. However, in contrast to the previously observed effects of chronic predation risk (Slos and Stoks 2008), we observed no inhibition of the antioxidant defence caused by chronic exposure. The observed short-term mobilization of an antioxidant system is likely to counteract negative effects of ROS released due to the increased metabolic rate and respiration. Nevertheless, the increased level of TBARS indicates that, despite the mobilization of antioxidant defence*, G. jazdzewskii* was unable to neutralize the higher productivity of ROS. Another physiological change in *G. jazdzewskii* during the initial contact with the predation cue was the increased concentration of heat shock proteins (Hsp70). These proteins are crucial for keeping the cell homeostasis under stress conditions induced by diverse internal and external factors, including predator presence (Slos and Stoks 2008; Hawlena and Schmitz 2010). However, their synthesis and maintenance are costly (Sørensen et al. 2003), and often positively correlated with growth reduction (Slos and Stoks 2008; Janssens and Stoks 2013).

We showed that in *D. villosus*, which maintained the higher metabolic rate for a longer period than the *G. jazdzewskii*, oxidative damage was not increased in the presence of the predation cue. This indicates its high tolerance to stress conditions in the presence of the predator, allowing it to save energetic resources, which could be spent on other functions, such as growth and/or reproduction. On the other hand, *G. jazdzewskii* had to expend additional resources for its physiological responses (mobilization of antioxidant enzymes and heat shock proteins) associated with the increased metabolism. Moreover, these physiological reactions were unable to fully counteract negative changes at cellular level. Generally, the more resources prey allocate into a defence mechanism aimed at reducing predation risk (including physiological adaptations), the higher the negative consequences of these mechanisms are, such as growth reduction. A relatively weak physiological response shown by *D. villosus* in our study can explain its resistance to non-consumptive predator effects, allowing it to sustain undisturbed growth rate under predatory pressure, in contrast to the less armoured and more active related species (Jermacz and Kobak 2017).

We have demonstrated that the tested species bear different costs of their initial responses to the predation cue. One of the potential explanations of this difference is related to different defence strategies exhibited by each species. Compared to *G. jazdzewskii*, *D. villosus* is well armoured with an exoskeleton harder than that of other gammarids (Błońska et al. 2015) and exhibits a “sit-and-wait” strategy with an inclination to cling to complex hard substrata independently of the current level of predation risk (Kobak et al. 2014; Jermacz et al. 2017a). Moreover, even immobilized individuals of *D. villosus* were selected by fish less often than *G. jazdzewskii* (Błońska et al. 2015), confirming the effectiveness of their non-inducible morphological anti-predator adaptations. Thus, many elements of *D. villosus*’ defence strategy are not induced by predator presence. Accordingly, its physiological response is not as pronounced as that exhibited by species relying on their inducible defence mechanisms. On the other hand, *G. jazdzewskii* exposed to predation in a limited space has been experimentally demonstrated to have a lower survival rate than other gammarids, even in sheltered conditions (Kobak et al. 2014; Błońska et al. 2015, 2016; Schmidt-Drewello et al. 2016). Therefore, to reduce predation risk in natural conditions, *G. jazdzewskii* has to relocate immediately after the detection of a predator. When movement is the optimum way to avoid predators, *G. jazdzewskii* maximizes its muscle efficiency to increase response effectiveness despite the negative consequences of sustaining a higher metabolic rate. Nevertheless, further studies on a range of species differing in their anti-predator defence strategies are needed to confirm this hypothesized relationship.

Contrary to our prediction, only *G. jazdzewskii* exhibited the most pronounced response immediately after predator detection, while its reactions after 1 and 7 days of pre-exposure to predation risk were significantly reduced. Nevertheless, chronic predation risk was demonstrated to generate significant oxidative damage (Slos and Stoks 2008; Janssens and Stoks 2014) affecting prey defence abilities (Janssens and Stoks 2014). Here we showed that only *G. jazdzewskii* that activated its cellular defence at the initial contact with the predation cue, significantly reduced its long-term responses, despite the fact that its metabolic level was still higher than that of the control individuals. Such a situation indicates that under prolonged predation risk prey organisms were able to reconfigure their physiology to keep their increased metabolism simultaneously reducing costs related to the higher ROS synthesis. Another proof of the cost reduction during the long-term response to predators was shown by Van Dievel et al. (2016), who demonstrated the predator-induced growth reduction in damselfly larvae during a shorter exposure (3 days), while the impact of a longer contact with a predator was negligible. In contrast to *G. jazdzewskii*, *D. villosus* did not demonstrate any symptoms of physiological reconfiguration related to the prolonged exposure to predation risk. Nevertheless, its higher metabolism was also not followed by oxidative damage. Therefore, we assume that modifications in the physiology of this species were not necessary.

Understanding the role of non-consumptive predator effects, including adaptive abilities of prey organisms, is important as it may affect a prey population even more than direct predation (Preisser et al. 2005). We experimentally demonstrated that both prey species increased their metabolism rates under predation risk; however, their costs related to the cellular defence system were significantly different, pointing to a better adaptation of *D. villosus* to prolonged predator pressure. This might be one of the reasons for its high invasive success in many areas to which this species has recently expanded (Rewicz et al. 2014). On the other hand, we showed that *G. jazdzewskii*, suffering higher costs of the initial contact with the predation cue, was able to reconfigure its physiology during the prolonged exposure and reduce its negative effects on its organism.

## Electronic supplementary material

Below is the link to the electronic supplementary material.

Supplementary file1 (PDF 3884 kb)
