# Supplementary material for: Experimental evidence for the adaptive response of aquatic invertebrates to chronic predation risk
Source: Oecologia. 2020 Jan 9;192(2):341–50. doi: 10.1007/s00442-020-04594-z (PMC7002334; doi:10.1007/s00442-020-04594-z)
Supplement: Supplementary file 1 — Supplementary file1 (PDF 3884 kb) [file 442_2020_4594_MOESM1_ESM.pdf]

Supporting information

Experimental evidence for the adaptive response of aquatic invertebrates to chronic predation risk

Łukasz Jermacz[ab*], Anna Nowakowska[c], Hanna Kletkiewicz[c] and Jarosław Kobak[a]

[a] Nicolaus Copernicus University, Faculty of Biology and Environmental Protection, Department of Invertebrate Zoology, Lwowska 1, 87-100 Toruń, Poland

[b] Nicolaus Copernicus University, Faculty of Biology and Environmental Protection, Department of Ecology and Biogeography, Lwowska 1, 87-100 Toruń, Poland

[c] Nicolaus Copernicus University, Faculty of Biology and Environmental Protection, Department of Animal Physiology, Lwowska 1, 87-100 Toruń, Poland

* Corresponding author: jermacz@umk.pl

Fig. S1 The outline presenting step-by-step experimental procedures

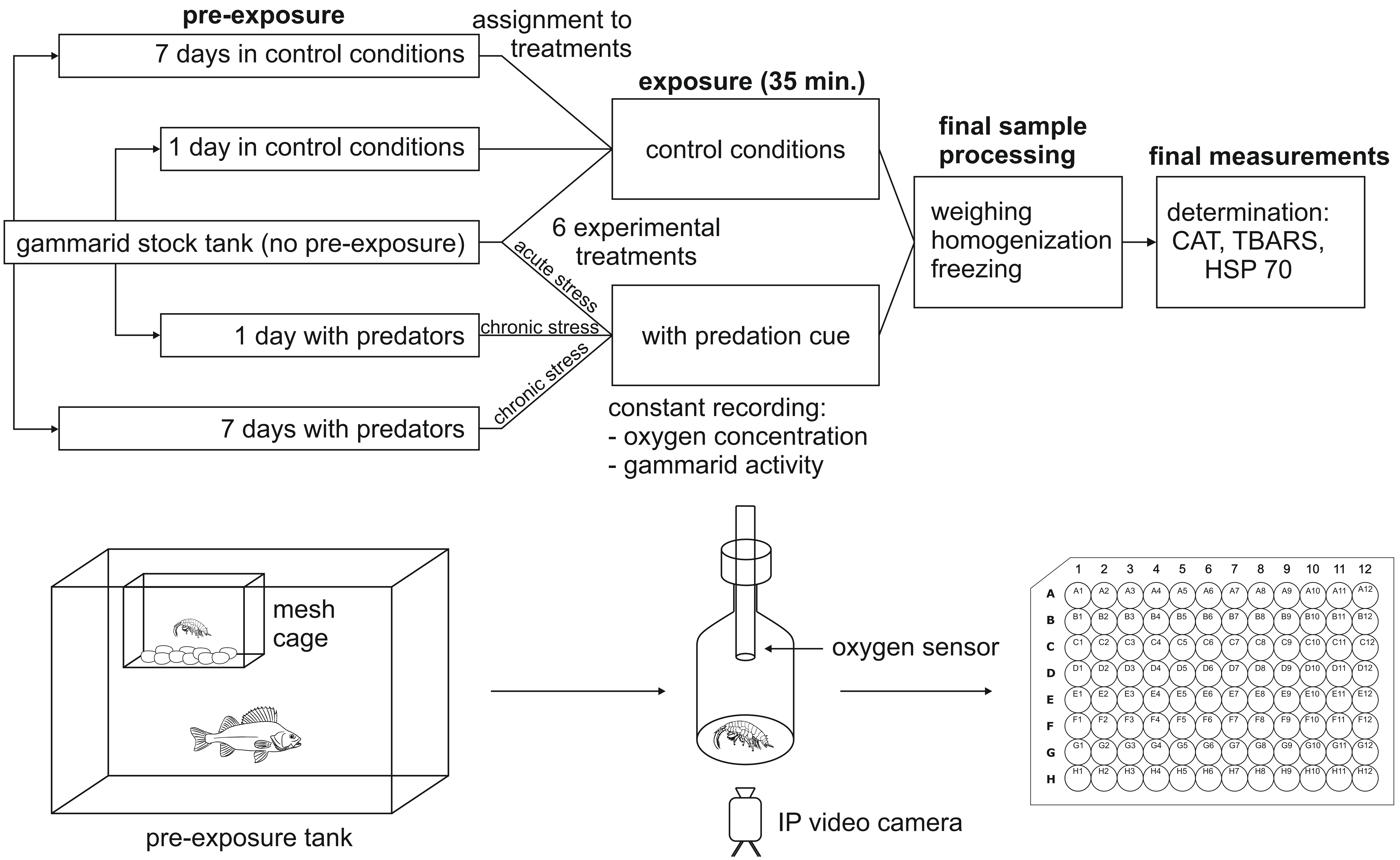

Gammarid species	treatment	weight [g]	pre-exposure time	trial	time [min.]	oxygen level [mg/l]	activity
D. villosus	perch	0.6821	0	1	1	8.33	20.29853
D. villosus	perch	0.6821	0	1	2	8.31	13.96075
D. villosus	perch	0.6821	0	1	3	8.28	18.5407
D. villosus	perch	0.6821	0	1	4	8.21	15.52002
D. villosus	perch	0.6821	0	1	5	8.17	17.50097
D. villosus	perch	0.6821	0	1	6	8.12	23.59846
D. villosus	perch	0.6821	0	1	7	8.08	20.16231
D. villosus	perch	0.6821	0	1	8	8.04	23.83882
D. villosus	perch	0.6821	0	1	9	7.98	23.75994
D. villosus	perch	0.6821	0	1	10	7.89	20.51842
D. villosus	perch	0.6821	0	1	11	7.9	20.22404
D. villosus	perch	0.6821	0	1	12	7.88	26.99559
D. villosus	perch	0.6821	0	1	13	7.86	22.92271
D. villosus	perch	0.6821	0	1	14	7.85	26.481
D. villosus	perch	0.6821	0	1	15	7.82	21.51787
D. villosus	perch	0.6821	0	1	16	7.8	24.5643
D. villosus	perch	0.6821	0	1	17	7.76	20.94356
D. villosus	perch	0.6821	0	1	18	7.68	28.57379
D. villosus	perch	0.6821	0	1	19	7.65	24.78246
D. villosus	perch	0.6821	0	1	20	7.63	22.79996
D. villosus	perch	0.6821	0	1	21	7.62	33.49979
D. villosus	perch	0.6821	0	1	22	7.6	21.7214
D. villosus	perch	0.6821	0	1	23	7.56	26.47834
D. villosus	perch	0.6821	0	1	24	7.49	19.12803
D. villosus	perch	0.6821	0	1	25	7.46	25.03842
D. villosus	perch	0.6821	0	1	26	7.45	22.08182
D. villosus	perch	0.6821	0	1	27	7.44	25.13465
D. villosus	perch	0.6821	0	1	28	7.41	24.40173
D. villosus	perch	0.6821	0	1	29	7.38	25.71987
D. villosus	perch	0.6821	0	1	30	7.35	21.3838
D. villosus	perch	0.7904	0	2	1	8.62	16.48033
D. villosus	perch	0.7904	0	2	2	8.58	15.70177
D. villosus	perch	0.7904	0	2	3	8.47	14.09855
D. villosus	perch	0.7904	0	2	4	8.42	12.81999
D. villosus	perch	0.7904	0	2	5	8.39	11.34
D. villosus	perch	0.7904	0	2	6	8.35	16.64097
D. villosus	perch	0.7904	0	2	7	8.34	14.59883
D. villosus	perch	0.7904	0	2	8	8.3	20.24046
D. villosus	perch	0.7904	0	2	9	8.3	16.09989
D. villosus	perch	0.7904	0	2	10	8.26	17.36068
D. villosus	perch	0.7904	0	2	11	8.24	15.87997
D. villosus	perch	0.7904	0	2	12	8.22	14
D. villosus	perch	0.7904	0	2	13	8.22	14.3419
D. villosus	perch	0.7904	0	2	14	8.19	14.71901
D. villosus	perch	0.7904	0	2	15	8.15	10.53786
D. villosus	perch	0.7904	0	2	16	8.13	17.14438
D. villosus	perch	0.7904	0	2	17	8.12	14.77649
D. villosus	perch	0.7904	0	2	18	8.1	11.54373

Gammarid species	treatment	weight [g]	pre-exposure time	trial	time [min.]	oxygen level [mg/l]	activity
D. villosus	perch	0.7904	0	2	19	8.07	12.54006
D. villosus	perch	0.7904	0	2	20	8.05	20.79859
D. villosus	perch	0.7904	0	2	21	8.03	17.89849
D. villosus	perch	0.7904	0	2	22	7.99	11.58144
D. villosus	perch	0.7904	0	2	23	7.95	18.92305
D. villosus	perch	0.7904	0	2	24	7.92	15.81361
D. villosus	perch	0.7904	0	2	25	7.89	17.28487
D. villosus	perch	0.7904	0	2	26	7.87	14.37999
D. villosus	perch	0.7904	0	2	27	7.84	8.818233
D. villosus	perch	0.7904	0	2	28	7.82	10.3181
D. villosus	perch	0.7904	0	2	29	7.8	15.30177
D. villosus	perch	0.7904	0	2	30	7.79	10.13996
D. villosus	perch	0.628	0	4	1	8.41	18.27981
D. villosus	perch	0.628	0	4	2	8.38	23.59996
D. villosus	perch	0.628	0	4	3	8.36	10.63929
D. villosus	perch	0.628	0	4	4	8.36	16.12069
D. villosus	perch	0.628	0	4	5	8.35	17.30132
D. villosus	perch	0.628	0	4	6	8.27	21.71895
D. villosus	perch	0.628	0	4	7	8.15	22.5817
D. villosus	perch	0.628	0	4	8	8.16	22.82009
D. villosus	perch	0.628	0	4	9	8.15	16.12006
D. villosus	perch	0.628	0	4	10	8.15	19.49849
D. villosus	perch	0.628	0	4	11	8.15	18.48078
D. villosus	perch	0.628	0	4	12	8.12	12.91916
D. villosus	perch	0.628	0	4	13	8.1	21.58372
D. villosus	perch	0.628	0	4	14	8.07	17.26215
D. villosus	perch	0.628	0	4	15	8.05	12.75596
D. villosus	perch	0.628	0	4	16	8.03	24.96107
D. villosus	perch	0.628	0	4	17	8.02	20.93764
D. villosus	perch	0.628	0	4	18	7.98	22.1599
D. villosus	perch	0.628	0	4	19	7.98	23.15725
D. villosus	perch	0.628	0	4	20	7.94	12.56264
D. villosus	perch	0.628	0	4	21	7.92	17.96559
D. villosus	perch	0.628	0	4	22	7.87	13.29861
D. villosus	perch	0.628	0	4	23	7.83	13.26467
D. villosus	perch	0.628	0	4	24	7.79	29.56163
D. villosus	perch	0.628	0	4	25	7.73	29.09671
D. villosus	perch	0.628	0	4	26	7.66	14.70011
D. villosus	perch	0.628	0	4	27	7.64	22.25815
D. villosus	perch	0.628	0	4	28	7.66	18.11994
D. villosus	perch	0.628	0	4	29	7.66	22.66374
D. villosus	perch	0.628	0	4	30	7.65	16.99216
D. villosus	perch	0.8299	0	5	1	8.57	12.16067
D. villosus	perch	0.8299	0	5	2	8.49	17.81971
D. villosus	perch	0.8299	0	5	3	8.45	17.22211
D. villosus	perch	0.8299	0	5	4	8.43	11.77778
D. villosus	perch	0.8299	0	5	5	8.35	8.699499
D. villosus	perch	0.8299	0	5	6	8.33	13.86202

Gammarid species	treatment	weight [g]	pre-exposure time	trial	time [min.]	oxygen level [mg/l]	activity
D. villosus	perch	0.8299	0	5	7	8.28	11.93945
D. villosus	perch	0.8299	0	5	8	8.27	13.79932
D. villosus	perch	0.8299	0	5	9	8.24	7.501368
D. villosus	perch	0.8299	0	5	10	8.2	11.68
D. villosus	perch	0.8299	0	5	11	8.18	12.84168
D. villosus	perch	0.8299	0	5	12	8.13	17.65736
D. villosus	perch	0.8299	0	5	13	8.1	5.920965
D. villosus	perch	0.8299	0	5	14	8.06	10.62206
D. villosus	perch	0.8299	0	5	15	7.96	10.55691
D. villosus	perch	0.8299	0	5	16	7.91	6.16222
D. villosus	perch	0.8299	0	5	17	7.86	8.217639
D. villosus	perch	0.8299	0	5	18	7.83	9.87991
D. villosus	perch	0.8299	0	5	19	7.81	4.679956
D. villosus	perch	0.8299	0	5	20	7.79	7.804062
D. villosus	perch	0.8299	0	5	21	7.8	16.19859
D. villosus	perch	0.8299	0	5	22	7.78	14.10897
D. villosus	perch	0.8299	0	5	23	7.79	10.58946
D. villosus	perch	0.8299	0	5	24	7.76	13.34156
D. villosus	perch	0.8299	0	5	25	7.67	7.956682
D. villosus	perch	0.8299	0	5	26	7.57	19.16154
D. villosus	perch	0.8299	0	5	27	7.54	5.860001
D. villosus	perch	0.8299	0	5	28	7.54	12.78365
D. villosus	perch	0.8299	0	5	29	7.55	8.218181
D. villosus	perch	0.8299	0	5	30	7.53	7.696089
D. villosus	perch	0.9908	0	6	1	8.16	18.49976
D. villosus	perch	0.9908	0	6	2	8.11	17.50084
D. villosus	perch	0.9908	0	6	3	8.06	21.4611
D. villosus	perch	0.9908	0	6	4	8.01	19.79807
D. villosus	perch	0.9908	0	6	5	7.94	23.60174
D. villosus	perch	0.9908	0	6	6	7.9	20.15903
D. villosus	perch	0.9908	0	6	7	7.86	22.01816
D. villosus	perch	0.9908	0	6	8	7.82	22.82239
D. villosus	perch	0.9908	0	6	9	7.77	23.11929
D. villosus	perch	0.9908	0	6	10	7.7	17.77916
D. villosus	perch	0.9908	0	6	11	7.67	24.04234
D. villosus	perch	0.9908	0	6	12	7.63	22.24092
D. villosus	perch	0.9908	0	6	13	7.56	17.27726
D. villosus	perch	0.9908	0	6	14	7.52	17.25996
D. villosus	perch	0.9908	0	6	15	7.51	21.92206
D. villosus	perch	0.9908	0	6	16	7.47	23.76335
D. villosus	perch	0.9908	0	6	17	7.42	12.65423
D. villosus	perch	0.9908	0	6	18	7.38	20.80489
D. villosus	perch	0.9908	0	6	19	7.33	18.59611
D. villosus	perch	0.9908	0	6	20	7.29	15.32268
D. villosus	perch	0.9908	0	6	21	7.26	17.30139
D. villosus	perch	0.9908	0	6	22	7.2	18.19992
D. villosus	perch	0.9908	0	6	23	7.19	17.19993
D. villosus	perch	0.9908	0	6	24	7.15	21.9999

Gammarid species	treatment	weight [g]	pre-exposure time	trial	time [min.]	oxygen level [mg/l]	activity
D. villosus	perch	0.9908	0	6	25	7.12	15.91827
D. villosus	perch	0.9908	0	6	26	7.06	9.583443
D. villosus	perch	0.9908	0	6	27	6.99	18.03822
D. villosus	perch	0.9908	0	6	28	6.91	13.60185
D. villosus	perch	0.9908	0	6	29	6.9	19.27422
D. villosus	perch	0.9908	0	6	30	6.88	14.42184
D. villosus	perch	0.695	0	7	1	8.51	6.040125
D. villosus	perch	0.695	0	7	2	8.48	10.27971
D. villosus	perch	0.695	0	7	3	8.43	8.14106
D. villosus	perch	0.695	0	7	4	8.36	9.638462
D. villosus	perch	0.695	0	7	5	8.31	15.45985
D. villosus	perch	0.695	0	7	6	8.27	7.299933
D. villosus	perch	0.695	0	7	7	8.27	9.22351
D. villosus	perch	0.695	0	7	8	8.23	16.15683
D. villosus	perch	0.695	0	7	9	8.2	5.802102
D. villosus	perch	0.695	0	7	10	8.18	6.099338
D. villosus	perch	0.695	0	7	11	8.15	9.320815
D. villosus	perch	0.695	0	7	12	8.12	10.31821
D. villosus	perch	0.695	0	7	13	8.1	5.660907
D. villosus	perch	0.695	0	7	14	8.08	4.962048
D. villosus	perch	0.695	0	7	15	8.05	6.916876
D. villosus	perch	0.695	0	7	16	8.02	7.901064
D. villosus	perch	0.695	0	7	17	7.99	8.182385
D. villosus	perch	0.695	0	7	18	7.94	5.237544
D. villosus	perch	0.695	0	7	19	7.91	13.36251
D. villosus	perch	0.695	0	7	20	7.86	8.580038
D. villosus	perch	0.695	0	7	21	7.83	10.4629
D. villosus	perch	0.695	0	7	22	7.8	18.28154
D. villosus	perch	0.695	0	7	23	7.77	8.153982
D. villosus	perch	0.695	0	7	24	7.7	15.44152
D. villosus	perch	0.695	0	7	25	7.69	10.78001
D. villosus	perch	0.695	0	7	26	7.64	10.56349
D. villosus	perch	0.695	0	7	27	7.64	9.914748
D. villosus	perch	0.695	0	7	28	7.63	13.03808
D. villosus	perch	0.695	0	7	29	7.61	5.287627
D. villosus	perch	0.695	0	7	30	7.59	7.338065
D. villosus	perch	0.8431	0	9	1	8.42	16.40051
D. villosus	perch	0.8431	0	9	2	8.37	22.77942
D. villosus	perch	0.8431	0	9	3	8.32	17.36066
D. villosus	perch	0.8431	0	9	4	8.28	11.33881
D. villosus	perch	0.8431	0	9	5	8.27	25.10168
D. villosus	perch	0.8431	0	9	6	8.23	19.11952
D. villosus	perch	0.8431	0	9	7	8.22	17.60061
D. villosus	perch	0.8431	0	9	8	8.17	12.45808
D. villosus	perch	0.8431	0	9	9	8.14	12.13988
D. villosus	perch	0.8431	0	9	10	8.13	12.35986
D. villosus	perch	0.8431	0	9	11	8.11	12.14073
D. villosus	perch	0.8431	0	9	12	8.1	5.96

Gammarid species	treatment	weight [g]	pre-exposure time	trial	time [min.]	oxygen level [mg/l]	activity
D. villosus	perch	0.8431	0	9	13	8.1	13.50183
D. villosus	perch	0.8431	0	9	14	8.07	11.66108
D. villosus	perch	0.8431	0	9	15	8.06	12.26113
D. villosus	perch	0.8431	0	9	16	8	13.50112
D. villosus	perch	0.8431	0	9	17	7.97	15.13642
D. villosus	perch	0.8431	0	9	18	7.94	13.48112
D. villosus	perch	0.8431	0	9	19	7.92	18.0878
D. villosus	perch	0.8431	0	9	20	7.86	14.91464
D. villosus	perch	0.8431	0	9	21	7.85	7.485752
D. villosus	perch	0.8431	0	9	22	7.81	16.05265
D. villosus	perch	0.8431	0	9	23	7.79	13.76455
D. villosus	perch	0.8431	0	9	24	7.77	19.17668
D. villosus	perch	0.8431	0	9	25	7.76	13.28828
D. villosus	perch	0.8431	0	9	26	7.71	12.47496
D. villosus	perch	0.8431	0	9	27	7.66	21.69629
D. villosus	perch	0.8431	0	9	28	7.62	22.28536
D. villosus	perch	0.8431	0	9	29	7.59	15.61811
D. villosus	perch	0.8431	0	9	30	7.58	18.72192
D. villosus	perch	0.7231	0	10	1	8.58	6.099395
D. villosus	perch	0.7231	0	10	2	8.53	8.780213
D. villosus	perch	0.7231	0	10	3	8.49	8.399619
D. villosus	perch	0.7231	0	10	4	8.45	6.959933
D. villosus	perch	0.7231	0	10	5	8.41	5.819943
D. villosus	perch	0.7231	0	10	6	8.34	16.12038
D. villosus	perch	0.7231	0	10	7	8.25	7.401185
D. villosus	perch	0.7231	0	10	8	8.21	8.940091
D. villosus	perch	0.7231	0	10	9	8.2	11.8179
D. villosus	perch	0.7231	0	10	10	8.19	9.121472
D. villosus	perch	0.7231	0	10	11	8.15	10.42164
D. villosus	perch	0.7231	0	10	12	8.11	11.66092
D. villosus	perch	0.7231	0	10	13	8.1	10.51816
D. villosus	perch	0.7231	0	10	14	8.07	11.71993
D. villosus	perch	0.7231	0	10	15	8.03	13.50101
D. villosus	perch	0.7231	0	10	16	8	11.6423
D. villosus	perch	0.7231	0	10	17	7.99	12.40252
D. villosus	perch	0.7231	0	10	18	7.96	9.397755
D. villosus	perch	0.7231	0	10	19	7.91	15.27612
D. villosus	perch	0.7231	0	10	20	7.9	9.798585
D. villosus	perch	0.7231	0	10	21	7.87	17.68991
D. villosus	perch	0.7231	0	10	22	7.82	15.75278
D. villosus	perch	0.7231	0	10	23	7.81	10.87689
D. villosus	perch	0.7231	0	10	24	7.77	10.68313
D. villosus	perch	0.7231	0	10	25	7.74	14.01664
D. villosus	perch	0.7231	0	10	26	7.72	9.865122
D. villosus	perch	0.7231	0	10	27	7.71	11.53647
D. villosus	perch	0.7231	0	10	28	7.71	10.2181
D. villosus	perch	0.7231	0	10	29	7.68	6.241856
D. villosus	perch	0.7231	0	10	30	7.63	15.41991

Gammarid species	treatment	weight [g]	pre-exposure time	trial	time [min.]	oxygen level [mg/l]	activity
D. villosus	perch	0.7827	0	11	1	8.54	21.54123
D. villosus	perch	0.7827	0	11	2	8.52	12.73904
D. villosus	perch	0.7827	0	11	3	8.47	9.500378
D. villosus	perch	0.7827	0	11	4	8.41	14.85878
D. villosus	perch	0.7827	0	11	5	8.35	11.46133
D. villosus	perch	0.7827	0	11	6	8.33	6.199042
D. villosus	perch	0.7827	0	11	7	8.3	20.06166
D. villosus	perch	0.7827	0	11	8	8.26	13.30077
D. villosus	perch	0.7827	0	11	9	8.24	6.07663
D. villosus	perch	0.7827	0	11	10	8.23	14.16143
D. villosus	perch	0.7827	0	11	11	8.18	15.67998
D. villosus	perch	0.7827	0	11	12	8.12	15.7444
D. villosus	perch	0.7827	0	11	13	8.04	10.68211
D. villosus	perch	0.7827	0	11	14	8.01	13.73621
D. villosus	perch	0.7827	0	11	15	7.98	16.02001
D. villosus	perch	0.7827	0	11	16	7.89	9.860082
D. villosus	perch	0.7827	0	11	17	7.86	21.16338
D. villosus	perch	0.7827	0	11	18	7.82	15.4775
D. villosus	perch	0.7827	0	11	19	7.77	10.37864
D. villosus	perch	0.7827	0	11	20	7.8	12.12678
D. villosus	perch	0.7827	0	11	21	7.8	12.45579
D. villosus	perch	0.7827	0	11	22	7.75	9.121473
D. villosus	perch	0.7827	0	11	23	7.53	14.97841
D. villosus	perch	0.7827	0	11	24	7.39	7.638371
D. villosus	perch	0.7827	0	11	25	7.53	11.04662
D. villosus	perch	0.7827	0	11	26	7.6	16.73486
D. villosus	perch	0.7827	0	11	27	7.57	10.24351
D. villosus	perch	0.7827	0	11	28	7.63	19.2218
D. villosus	perch	0.7827	0	11	29	7.66	18.23422
D. villosus	perch	0.7827	0	11	30	7.65	11.60582
D. villosus	perch	0.8072	0	12	1	8.53	5.220254
D. villosus	perch	0.8072	0	12	2	8.51	6.420539
D. villosus	perch	0.8072	0	12	3	8.48	6.959928
D. villosus	perch	0.8072	0	12	4	8.45	7.46041
D. villosus	perch	0.8072	0	12	5	8.41	7.43993
D. villosus	perch	0.8072	0	12	6	8.38	4.999588
D. villosus	perch	0.8072	0	12	7	8.33	5.981147
D. villosus	perch	0.8072	0	12	8	8.32	6.217839
D. villosus	perch	0.8072	0	12	9	8.3	5.24109
D. villosus	perch	0.8072	0	12	10	8.25	6.019707
D. villosus	perch	0.8072	0	12	11	8.23	8.719917
D. villosus	perch	0.8072	0	12	12	8.19	3.241166
D. villosus	perch	0.8072	0	12	13	8.18	5.301388
D. villosus	perch	0.8072	0	12	14	8.17	7.459991
D. villosus	perch	0.8072	0	12	15	8.16	10.29996
D. villosus	perch	0.8072	0	12	16	8.16	8.260343
D. villosus	perch	0.8072	0	12	17	8.12	12.21947
D. villosus	perch	0.8072	0	12	18	8.08	8.920285

Gammarid species	treatment	weight [g]	pre-exposure time	trial	time [min.]	oxygen level [mg/l]	activity
D. villosus	perch	0.8072	0	12	19	8.08	15.28483
D. villosus	perch	0.8072	0	12	20	8.06	19.3586
D. villosus	perch	0.8072	0	12	21	8.05	12.66
D. villosus	perch	0.8072	0	12	22	8.03	9.279907
D. villosus	perch	0.8072	0	12	23	8.03	7.581417
D. villosus	perch	0.8072	0	12	24	8.03	8.838351
D. villosus	perch	0.8072	0	12	25	7.97	10.40188
D. villosus	perch	0.8072	0	12	26	7.93	9.820019
D. villosus	perch	0.8072	0	12	27	7.89	3.640805
D. villosus	perch	0.8072	0	12	28	7.86	7.699389
D. villosus	perch	0.8072	0	12	29	7.82	3.481587
D. villosus	perch	0.8072	0	12	30	7.81	3.339968
D. villosus	perch	0.7888	0	14	1	8.62	6.839877
D. villosus	perch	0.7888	0	14	2	8.55	6.220661
D. villosus	perch	0.7888	0	14	3	8.51	9.258716
D. villosus	perch	0.7888	0	14	4	8.49	6.300775
D. villosus	perch	0.7888	0	14	5	8.43	11.93952
D. villosus	perch	0.7888	0	14	6	8.4	6.340546
D. villosus	perch	0.7888	0	14	7	8.36	7.879448
D. villosus	perch	0.7888	0	14	8	8.31	5.200009
D. villosus	perch	0.7888	0	14	9	8.27	10.09996
D. villosus	perch	0.7888	0	14	10	8.25	1.919261
D. villosus	perch	0.7888	0	14	11	8.21	9.699899
D. villosus	perch	0.7888	0	14	12	8.18	6.499933
D. villosus	perch	0.7888	0	14	13	8.15	14.40369
D. villosus	perch	0.7888	0	14	14	8.06	4.856115
D. villosus	perch	0.7888	0	14	15	8.01	5.541028
D. villosus	perch	0.7888	0	14	16	7.99	3.699962
D. villosus	perch	0.7888	0	14	17	7.99	0.902392
D. villosus	perch	0.7888	0	14	18	7.99	8.719975
D. villosus	perch	0.7888	0	14	19	7.99	3.178708
D. villosus	perch	0.7888	0	14	20	7.96	3.059968
D. villosus	perch	0.7888	0	14	21	7.97	5.419944
D. villosus	perch	0.7888	0	14	22	7.94	4.92295
D. villosus	perch	0.7888	0	14	23	7.91	4.378516
D. villosus	perch	0.7888	0	14	24	7.87	3.700023
D. villosus	perch	0.7888	0	14	25	7.86	6.219935
D. villosus	perch	0.7888	0	14	26	7.84	10.6799
D. villosus	perch	0.7888	0	14	27	7.81	4.45996
D. villosus	perch	0.7888	0	14	28	7.8	3.92182
D. villosus	perch	0.7888	0	14	29	7.79	7.347608
D. villosus	perch	0.7888	0	14	30	7.76	7.258195
D. villosus	perch	0.7644	0	15	1	8.42	5.799768
D. villosus	perch	0.7644	0	15	2	8.41	4.820252
D. villosus	perch	0.7644	0	15	3	8.38	4.380315
D. villosus	perch	0.7644	0	15	4	8.33	11.22036
D. villosus	perch	0.7644	0	15	5	8.27	9.179072
D. villosus	perch	0.7644	0	15	6	8.23	6.779933

Gammarid species	treatment	weight [g]	pre-exposure time	trial	time [min.]	oxygen level [mg/l]	activity
D. villosus	perch	0.7644	0	15	7	8.17	9.721101
D. villosus	perch	0.7644	0	15	8	8.14	10.34068
D. villosus	perch	0.7644	0	15	9	8.1	6.177961
D. villosus	perch	0.7644	0	15	10	8.07	7.09993
D. villosus	perch	0.7644	0	15	11	8.03	9.580756
D. villosus	perch	0.7644	0	15	12	8.02	6.679998
D. villosus	perch	0.7644	0	15	13	8	4.799052
D. villosus	perch	0.7644	0	15	14	7.92	6.099939
D. villosus	perch	0.7644	0	15	15	7.88	8.880993
D. villosus	perch	0.7644	0	15	16	7.8	9.263403
D. villosus	perch	0.7644	0	15	17	7.64	7.036509
D. villosus	perch	0.7644	0	15	18	7.63	5.37995
D. villosus	perch	0.7644	0	15	19	7.58	9.979901
D. villosus	perch	0.7644	0	15	20	7.57	9.461286
D. villosus	perch	0.7644	0	15	21	7.59	6.438554
D. villosus	perch	0.7644	0	15	22	7.6	5.39995
D. villosus	perch	0.7644	0	15	23	7.58	9.784574
D. villosus	perch	0.7644	0	15	24	7.28	12.69842
D. villosus	perch	0.7644	0	15	25	7.03	8.018358
D. villosus	perch	0.7644	0	15	26	6.98	10.69822
D. villosus	perch	0.7644	0	15	27	7.04	8.943514
D. villosus	perch	0.7644	0	15	28	6.8	8.021906
D. villosus	perch	0.7644	0	15	29	6.76	6.856273
D. villosus	perch	0.7644	0	15	30	6.74	12.35796
D. villosus	perch	0.8644	0	16	1	8.43	11.54109
D. villosus	perch	0.8644	0	16	2	8.4	4.459298
D. villosus	perch	0.8644	0	16	3	8.35	4.920014
D. villosus	perch	0.8644	0	16	4	8.3	12.30204
D. villosus	perch	0.8644	0	16	5	8.22	14.81877
D. villosus	perch	0.8644	0	16	6	8.09	7.278492
D. villosus	perch	0.8644	0	16	7	8.06	6.142348
D. villosus	perch	0.8644	0	16	8	7.97	20.7774
D. villosus	perch	0.8644	0	16	9	7.53	1.922141
D. villosus	perch	0.8644	0	16	10	7.71	17.21923
D. villosus	perch	0.8644	0	16	11	7.81	6.920052
D. villosus	perch	0.8644	0	16	12	7.74	5.919163
D. villosus	perch	0.8644	0	16	13	7.71	3.920981
D. villosus	perch	0.8644	0	16	14	7.73	5.639044
D. villosus	perch	0.8644	0	16	15	7.68	3.340027
D. villosus	perch	0.8644	0	16	16	7.85	3.898881
D. villosus	perch	0.8644	0	16	17	7.79	7.601125
D. villosus	perch	0.8644	0	16	18	7.67	3.841283
D. villosus	perch	0.8644	0	16	19	7.7	5.157431
D. villosus	perch	0.8644	0	16	20	7.66	8.519913
D. villosus	perch	0.8644	0	16	21	7.69	11.29988
D. villosus	perch	0.8644	0	16	22	7.68	9.141409
D. villosus	perch	0.8644	0	16	23	7.77	15.78148
D. villosus	perch	0.8644	0	16	24	7.7	15.83672

Gammarid species	treatment	weight [g]	pre-exposure time	trial	time [min.]	oxygen level [mg/l]	activity
D. villosus	perch	0.8644	0	16	25	7.59	9.688306
D. villosus	perch	0.8644	0	16	26	7.6	12.13659
D. villosus	perch	0.8644	0	16	27	7.49	10.53996
D. villosus	perch	0.8644	0	16	28	7.41	5.565588
D. villosus	perch	0.8644	0	16	29	7.44	13.99818
D. villosus	perch	0.8644	0	16	30	7.51	17.75605
D. villosus	perch	0.76	0	18	1	8.5	5.179827
D. villosus	perch	0.76	0	18	2	8.47	3.399726
D. villosus	perch	0.76	0	18	3	8.45	7.439924
D. villosus	perch	0.76	0	18	4	8.41	3.00039
D. villosus	perch	0.76	0	18	5	8.39	8.51998
D. villosus	perch	0.76	0	18	6	8.36	4.239478
D. villosus	perch	0.76	0	18	7	8.34	7.000531
D. villosus	perch	0.76	0	18	8	8.3	5.55994
D. villosus	perch	0.76	0	18	9	8.29	1.280708
D. villosus	perch	0.76	0	18	10	8.27	4.140022
D. villosus	perch	0.76	0	18	11	8.26	3.400026
D. villosus	perch	0.76	0	18	12	8.25	3.199132
D. villosus	perch	0.76	0	18	13	8.23	5.599938
D. villosus	perch	0.76	0	18	14	8.19	1.342027
D. villosus	perch	0.76	0	18	15	8.16	4.658934
D. villosus	perch	0.76	0	18	16	8.16	4.421101
D. villosus	perch	0.76	0	18	17	8.13	2.818836
D. villosus	perch	0.76	0	18	18	8.12	6.181195
D. villosus	perch	0.76	0	18	19	8.12	4.021339
D. villosus	perch	0.76	0	18	20	8.1	4.59731
D. villosus	perch	0.76	0	18	21	8.09	6.081386
D. villosus	perch	0.76	0	18	22	8.07	3.198529
D. villosus	perch	0.76	0	18	23	8.05	2.599974
D. villosus	perch	0.76	0	18	24	7.99	5.219945
D. villosus	perch	0.76	0	18	25	7.97	2.701657
D. villosus	perch	0.76	0	18	26	7.96	6.239998
D. villosus	perch	0.76	0	18	27	7.93	7.479928
D. villosus	perch	0.76	0	18	28	7.93	6.159936
D. villosus	perch	0.76	0	18	29	7.9	10.10565
D. villosus	perch	0.76	0	18	30	7.88	9.660085
D. villosus	perch	0.7714	0	19	1	8.65	7.520164
D. villosus	perch	0.7714	0	19	2	8.59	17.89941
D. villosus	perch	0.7714	0	19	3	8.54	10.88026
D. villosus	perch	0.7714	0	19	4	8.52	12.91995
D. villosus	perch	0.7714	0	19	5	8.47	10.05996
D. villosus	perch	0.7714	0	19	6	8.44	4.260017
D. villosus	perch	0.7714	0	19	7	8.42	5.87941
D. villosus	perch	0.7714	0	19	8	8.38	14.66249
D. villosus	perch	0.7714	0	19	9	8.35	11.29725
D. villosus	perch	0.7714	0	19	10	8.34	7.601478
D. villosus	perch	0.7714	0	19	11	8.32	14.76081
D. villosus	perch	0.7714	0	19	12	8.3	14.88183

Gammarid species	treatment	weight [g]	pre-exposure time	trial	time [min.]	oxygen level [mg/l]	activity
D. villosus	perch	0.7714	0	19	13	8.27	9.63733
D. villosus	perch	0.7714	0	19	14	8.26	14.01896
D. villosus	perch	0.7714	0	19	15	8.23	9.903199
D. villosus	perch	0.7714	0	19	16	8.21	9.576676
D. villosus	perch	0.7714	0	19	17	8.21	7.019926
D. villosus	perch	0.7714	0	19	18	8.19	14.43986
D. villosus	perch	0.7714	0	19	19	8.17	14.5625
D. villosus	perch	0.7714	0	19	20	8.15	10.30554
D. villosus	perch	0.7714	0	19	21	8.14	11.25731
D. villosus	perch	0.7714	0	19	22	8.1	13.99853
D. villosus	perch	0.7714	0	19	23	8.07	12.14157
D. villosus	perch	0.7714	0	19	24	8.06	10.61847
D. villosus	perch	0.7714	0	19	25	8.05	12.43832
D. villosus	perch	0.7714	0	19	26	8.03	11.75995
D. villosus	perch	0.7714	0	19	27	8	9.008965
D. villosus	perch	0.7714	0	19	28	7.99	12.09281
D. villosus	perch	0.7714	0	19	29	7.97	11.64192
D. villosus	perch	0.7714	0	19	30	7.88	16.92595
D. villosus	perch	0.714	0	20	1	8.75	4.919952
D. villosus	perch	0.714	0	20	2	8.7	10.38051
D. villosus	perch	0.714	0	20	3	8.66	4.579361
D. villosus	perch	0.714	0	20	4	8.63	5.480789
D. villosus	perch	0.714	0	20	5	8.6	11.68049
D. villosus	perch	0.714	0	20	6	8.56	10.099
D. villosus	perch	0.714	0	20	7	8.56	3.539425
D. villosus	perch	0.714	0	20	8	8.53	3.220628
D. villosus	perch	0.714	0	20	9	8.52	5.47995
D. villosus	perch	0.714	0	20	10	8.5	3.564644
D. villosus	perch	0.714	0	20	11	8.47	8.8577
D. villosus	perch	0.714	0	20	12	8.44	4.378276
D. villosus	perch	0.714	0	20	13	8.43	7.083763
D. villosus	perch	0.714	0	20	14	8.4	11.43604
D. villosus	perch	0.714	0	20	15	8.38	2.96105
D. villosus	perch	0.714	0	20	16	8.37	5.858862
D. villosus	perch	0.714	0	20	17	8.35	8.281117
D. villosus	perch	0.714	0	20	18	8.34	4.720013
D. villosus	perch	0.714	0	20	19	8.31	18.04121
D. villosus	perch	0.714	0	20	20	8.2	14.71997
D. villosus	perch	0.714	0	20	21	8.18	3.417206
D. villosus	perch	0.714	0	20	22	8.15	7.199923
D. villosus	perch	0.714	0	20	23	8.15	2.663093
D. villosus	perch	0.714	0	20	24	8.16	10.42164
D. villosus	perch	0.714	0	20	25	8.16	5.378447
D. villosus	perch	0.714	0	20	26	8.18	13.3765
D. villosus	perch	0.714	0	20	27	8.15	1.519985
D. villosus	perch	0.714	0	20	28	8.15	6.621792
D. villosus	perch	0.714	0	20	29	8.15	3.061891
D. villosus	perch	0.714	0	20	30	8.13	2.658054

Gammarid species	treatment	weight [g]	pre-exposure time	trial	time [min.]	oxygen level [mg/l]	activity
D. villosus	perch	0.7055	0	21	1	8.7	7.079812
D. villosus	perch	0.7055	0	21	2	8.68	3.240028
D. villosus	perch	0.7055	0	21	3	8.64	2.601473
D. villosus	perch	0.7055	0	21	4	8.62	3.360986
D. villosus	perch	0.7055	0	21	5	8.48	5.798798
D. villosus	perch	0.7055	0	21	6	8.41	2.319557
D. villosus	perch	0.7055	0	21	7	8.39	3.180568
D. villosus	perch	0.7055	0	21	8	8.4	12.08061
D. villosus	perch	0.7055	0	21	9	8.37	4.899292
D. villosus	perch	0.7055	0	21	10	8.36	0.519995
D. villosus	perch	0.7055	0	21	11	8.36	4.901633
D. villosus	perch	0.7055	0	21	12	8.35	3.819184
D. villosus	perch	0.7055	0	21	13	8.33	5.643845
D. villosus	perch	0.7055	0	21	14	8.31	6.996151
D. villosus	perch	0.7055	0	21	15	8.31	11.67887
D. villosus	perch	0.7055	0	21	16	8.29	6.563354
D. villosus	perch	0.7055	0	21	17	8.29	4.638815
D. villosus	perch	0.7055	0	21	18	8.25	10.70242
D. villosus	perch	0.7055	0	21	19	8.24	6.120059
D. villosus	perch	0.7055	0	21	20	8.21	7.199992
D. villosus	perch	0.7055	0	21	21	8.2	5.898559
D. villosus	perch	0.7055	0	21	22	8.2	6.067439
D. villosus	perch	0.7055	0	21	23	8.19	7.178554
D. villosus	perch	0.7055	0	21	24	8.18	6.878436
D. villosus	perch	0.7055	0	21	25	8.16	3.340026
D. villosus	perch	0.7055	0	21	26	8.13	12.22169
D. villosus	perch	0.7055	0	21	27	8.09	6.578257
D. villosus	perch	0.7055	0	21	28	8.07	5.881804
D. villosus	perch	0.7055	0	21	29	8.05	6.818074
D. villosus	perch	0.7055	0	21	30	8.03	5.763904
D. villosus	perch	0.6565	0	22	1	8.66	4.220499
D. villosus	perch	0.6565	0	22	2	8.6	19.40028
D. villosus	perch	0.6565	0	22	3	8.55	11.95927
D. villosus	perch	0.6565	0	22	4	8.51	1.261247
D. villosus	perch	0.6565	0	22	5	8.47	1.699142
D. villosus	perch	0.6565	0	22	6	8.46	1.99998
D. villosus	perch	0.6565	0	22	7	8.44	0.419996
D. villosus	perch	0.6565	0	22	8	8.42	8.540576
D. villosus	perch	0.6565	0	22	9	8.4	2.559316
D. villosus	perch	0.6565	0	22	10	8.33	0.939991
D. villosus	perch	0.6565	0	22	11	8.25	6.999928
D. villosus	perch	0.6565	0	22	12	8.14	6.019947
D. villosus	perch	0.6565	0	22	13	8.14	2.420939
D. villosus	perch	0.6565	0	22	14	8.05	3.420028
D. villosus	perch	0.6565	0	22	15	8.05	6.478912
D. villosus	perch	0.6565	0	22	16	8.14	0.899991
D. villosus	perch	0.6565	0	22	17	8.17	1.799983
D. villosus	perch	0.6565	0	22	18	8.16	4.919953

Gammarid species	treatment	weight [g]	pre-exposure time	trial	time [min.]	oxygen level [mg/l]	activity
D. villosus	perch	0.6565	0	22	19	8.17	6.779929
D. villosus	perch	0.6565	0	22	20	7.46	0.239998
D. villosus	perch	0.6565	0	22	21	6.98	9.519889
D. villosus	perch	0.6565	0	22	22	7.23	5.37995
D. villosus	perch	0.6565	0	22	23	7.42	1.99998
D. villosus	perch	0.6565	0	22	24	7.49	6.088037
D. villosus	perch	0.6565	0	22	25	7.73	4.311857
D. villosus	perch	0.6565	0	22	26	7.84	3.701703
D. villosus	perch	0.6565	0	22	27	7.9	4.583614
D. villosus	perch	0.6565	0	22	28	7.92	3.354568
D. villosus	perch	0.6565	0	22	29	7.94	1.99998
D. villosus	perch	0.6565	0	22	30	7.96	5.939945
D. villosus	perch	0.8429	0	23	1	8.51	13.77951
D. villosus	perch	0.8429	0	23	2	8.47	3.120568
D. villosus	perch	0.8429	0	23	3	8.42	7.460045
D. villosus	perch	0.8429	0	23	4	8.38	8.200041
D. villosus	perch	0.8429	0	23	5	8.35	5.340547
D. villosus	perch	0.8429	0	23	6	8.31	8.998474
D. villosus	perch	0.8429	0	23	7	8.3	5.139951
D. villosus	perch	0.8429	0	23	8	8.27	10.9212
D. villosus	perch	0.8429	0	23	9	8.24	8.720038
D. villosus	perch	0.8429	0	23	10	8.2	6.059221
D. villosus	perch	0.8429	0	23	11	8.19	8.141597
D. villosus	perch	0.8429	0	23	12	8.17	7.621842
D. villosus	perch	0.8429	0	23	13	8.14	12.76012
D. villosus	perch	0.8429	0	23	14	8.12	7.579088
D. villosus	perch	0.8429	0	23	15	8.11	9.897864
D. villosus	perch	0.8429	0	23	16	8.06	13.06329
D. villosus	perch	0.8429	0	23	17	8.04	8.097697
D. villosus	perch	0.8429	0	23	18	8.01	9.944993
D. villosus	perch	0.8429	0	23	19	8	11.03756
D. villosus	perch	0.8429	0	23	20	7.99	15.48141
D. villosus	perch	0.8429	0	23	21	7.97	16.60151
D. villosus	perch	0.8429	0	23	22	7.93	11.16156
D. villosus	perch	0.8429	0	23	23	7.93	7.638544
D. villosus	perch	0.8429	0	23	24	7.91	6.380058
D. villosus	perch	0.8429	0	23	25	7.88	8.261718
D. villosus	perch	0.8429	0	23	26	7.85	13.56004
D. villosus	perch	0.8429	0	23	27	7.83	13.38365
D. villosus	perch	0.8429	0	23	28	7.81	7.578312
D. villosus	perch	0.8429	0	23	29	7.79	7.722027
D. villosus	perch	0.8429	0	23	30	7.77	4.192281
D. villosus	perch	0.5457	0	24	1	8.63	2.260218
D. villosus	perch	0.5457	0	24	2	8.58	2.500275
D. villosus	perch	0.5457	0	24	3	8.56	17.26132
D. villosus	perch	0.5457	0	24	4	8.53	5.458148
D. villosus	perch	0.5457	0	24	5	8.48	5.181383
D. villosus	perch	0.5457	0	24	6	8.42	4.198522

Gammarid species	treatment	weight [g]	pre-exposure time	trial	time [min.]	oxygen level [mg/l]	activity
D. villosus	perch	0.5457	0	24	7	8.35	1.079989
D. villosus	perch	0.5457	0	24	8	8.19	2.800632
D. villosus	perch	0.5457	0	24	9	8.11	7.282149
D. villosus	perch	0.5457	0	24	10	8.09	9.697742
D. villosus	perch	0.5457	0	24	11	8.07	5.121633
D. villosus	perch	0.5457	0	24	12	8.05	2.460095
D. villosus	perch	0.5457	0	24	13	8.06	3.060089
D. villosus	perch	0.5457	0	24	14	8.07	6.324073
D. villosus	perch	0.5457	0	24	15	8.08	6.494837
D. villosus	perch	0.5457	0	24	16	8.09	2.619973
D. villosus	perch	0.5457	0	24	17	8.09	0.979991
D. villosus	perch	0.5457	0	24	18	8.12	5.181211
D. villosus	perch	0.5457	0	24	19	8.14	7.063949
D. villosus	perch	0.5457	0	24	20	8.14	8.556019
D. villosus	perch	0.5457	0	24	21	8.13	6.88287
D. villosus	perch	0.5457	0	24	22	8.13	6.357116
D. villosus	perch	0.5457	0	24	23	8.11	3.778463
D. villosus	perch	0.5457	0	24	24	8.09	3.461585
D. villosus	perch	0.5457	0	24	25	8.11	11.71993
D. villosus	perch	0.5457	0	24	26	8.09	3.238289
D. villosus	perch	0.5457	0	24	27	8.11	10.54171
D. villosus	perch	0.5457	0	24	28	8.07	11.23808
D. villosus	perch	0.5457	0	24	29	8.05	14.65137
D. villosus	perch	0.5457	0	24	30	8.04	5.170347
D. villosus	perch	0.721	0	26	1	8.59	12.4588
D. villosus	perch	0.721	0	26	2	8.55	4.439955
D. villosus	perch	0.721	0	26	3	8.5	6.119947
D. villosus	perch	0.721	0	26	4	8.47	0.159999
D. villosus	perch	0.721	0	26	5	8.46	9.562304
D. villosus	perch	0.721	0	26	6	8.39	16.75905
D. villosus	perch	0.721	0	26	7	8.37	5.178872
D. villosus	perch	0.721	0	26	8	8.36	9.599905
D. villosus	perch	0.721	0	26	9	8.34	7.479931
D. villosus	perch	0.721	0	26	10	8.31	4.800734
D. villosus	perch	0.721	0	26	11	8.29	2.359196
D. villosus	perch	0.721	0	26	12	8.27	6.699936
D. villosus	perch	0.721	0	26	13	8.24	10.39989
D. villosus	perch	0.721	0	26	14	8.22	2.019979
D. villosus	perch	0.721	0	26	15	8.19	2.319978
D. villosus	perch	0.721	0	26	16	8.19	8.783339
D. villosus	perch	0.721	0	26	17	8.17	10.31763
D. villosus	perch	0.721	0	26	18	8.18	0.499996
D. villosus	perch	0.721	0	26	19	8.15	9.361228
D. villosus	perch	0.721	0	26	20	8.06	1.05867
D. villosus	perch	0.721	0	26	21	8.06	7.761367
D. villosus	perch	0.721	0	26	22	8.02	2.358538
D. villosus	perch	0.721	0	26	23	7.93	2.699973
D. villosus	perch	0.721	0	26	24	7.81	1.839982

Gammarid species	treatment	weight [g]	pre-exposure time	trial	time [min.]	oxygen level [mg/l]	activity
D. villosus	perch	0.721	0	26	25	7.69	4.821632
D. villosus	perch	0.721	0	26	26	7.71	2.478295
D. villosus	perch	0.721	0	26	27	7.72	9.9617
D. villosus	perch	0.721	0	26	28	7.63	2.998169
D. villosus	perch	0.721	0	26	29	7.61	2.539975
D. villosus	perch	0.721	0	26	30	7.52	2.043941
D. villosus	no predator	0.5659	0	27	1	8.88	1.499985
D. villosus	no predator	0.5659	0	27	2	8.88	1.400287
D. villosus	no predator	0.5659	0	27	3	8.86	2.579675
D. villosus	no predator	0.5659	0	27	4	8.85	3.979961
D. villosus	no predator	0.5659	0	27	5	8.84	0.121439
D. villosus	no predator	0.5659	0	27	6	8.83	2.278538
D. villosus	no predator	0.5659	0	27	7	8.8	11.6011
D. villosus	no predator	0.5659	0	27	8	8.75	9.698701
D. villosus	no predator	0.5659	0	27	9	8.74	1.659985
D. villosus	no predator	0.5659	0	27	10	8.73	2.02154
D. villosus	no predator	0.5659	0	27	11	8.72	1.758422
D. villosus	no predator	0.5659	0	27	12	8.7	1.199989
D. villosus	no predator	0.5659	0	27	13	8.67	0.819993
D. villosus	no predator	0.5659	0	27	14	8.66	1.141009
D. villosus	no predator	0.5659	0	27	15	8.66	2.95997
D. villosus	no predator	0.5659	0	27	16	8.64	1.97998
D. villosus	no predator	0.5659	0	27	17	8.62	0.839992
D. villosus	no predator	0.5659	0	27	18	8.63	2.01998
D. villosus	no predator	0.5659	0	27	19	8.6	2.439975
D. villosus	no predator	0.5659	0	27	20	8.59	1.699983
D. villosus	no predator	0.5659	0	27	21	8.57	4.701393
D. villosus	no predator	0.5659	0	27	22	8.52	2.600033
D. villosus	no predator	0.5659	0	27	23	8.51	5.118452
D. villosus	no predator	0.5659	0	27	24	8.49	2.339977
D. villosus	no predator	0.5659	0	27	25	8.48	5.601632
D. villosus	no predator	0.5659	0	27	26	8.45	6.199996
D. villosus	no predator	0.5659	0	27	27	8.47	2.518234
D. villosus	no predator	0.5659	0	27	28	8.44	2.125558
D. villosus	no predator	0.5659	0	27	29	8.42	1.516265
D. villosus	no predator	0.5659	0	27	30	8.42	5.681927
D. villosus	no predator	0.605	0	28	1	8.87	2.220217
D. villosus	no predator	0.605	0	28	2	8.87	0.839752
D. villosus	no predator	0.605	0	28	3	8.83	1.339986
D. villosus	no predator	0.605	0	28	4	8.77	9.840325
D. villosus	no predator	0.605	0	28	5	8.74	6.040001
D. villosus	no predator	0.605	0	28	6	8.69	2.079499
D. villosus	no predator	0.605	0	28	7	8.68	2.379976
D. villosus	no predator	0.605	0	28	8	8.68	0.899992
D. villosus	no predator	0.605	0	28	9	8.68	0.959991
D. villosus	no predator	0.605	0	28	10	8.66	2.359976
D. villosus	no predator	0.605	0	28	11	8.65	0.89999
D. villosus	no predator	0.605	0	28	12	8.64	2.439974

Gammarid species	treatment	weight [g]	pre-exposure time	trial	time [min.]	oxygen level [mg/l]	activity
D. villosus	no predator	0.605	0	28	13	8.64	0.399996
D. villosus	no predator	0.605	0	28	14	8.63	0.359996
D. villosus	no predator	0.605	0	28	15	8.63	0.759993
D. villosus	no predator	0.605	0	28	16	8.61	5.243366
D. villosus	no predator	0.605	0	28	17	8.58	1.677703
D. villosus	no predator	0.605	0	28	18	8.58	0.699993
D. villosus	no predator	0.605	0	28	19	8.56	2.519975
D. villosus	no predator	0.605	0	28	20	8.54	1.399986
D. villosus	no predator	0.605	0	28	21	8.55	2.341418
D. villosus	no predator	0.605	0	28	22	8.53	1.05855
D. villosus	no predator	0.605	0	28	23	8.51	1.139989
D. villosus	no predator	0.605	0	28	24	8.5	2.03998
D. villosus	no predator	0.605	0	28	25	8.5	19.2499
D. villosus	no predator	0.605	0	28	26	8.48	13.71146
D. villosus	no predator	0.605	0	28	27	8.45	0.199998
D. villosus	no predator	0.605	0	28	28	8.45	10.0399
D. villosus	no predator	0.605	0	28	29	8.43	1.339987
D. villosus	no predator	0.605	0	28	30	8.43	2.761951
D. villosus	no predator	0.662	0	29	1	8.87	13.86058
D. villosus	no predator	0.662	0	29	2	8.84	16.85934
D. villosus	no predator	0.662	0	29	3	8.82	7.801357
D. villosus	no predator	0.662	0	29	4	8.8	17.56008
D. villosus	no predator	0.662	0	29	5	8.78	9.540144
D. villosus	no predator	0.662	0	29	6	8.76	12.37905
D. villosus	no predator	0.662	0	29	7	8.76	17.32108
D. villosus	no predator	0.662	0	29	8	8.72	5.558807
D. villosus	no predator	0.662	0	29	9	8.7	13.98206
D. villosus	no predator	0.662	0	29	10	8.68	8.937758
D. villosus	no predator	0.662	0	29	11	8.66	10.6599
D. villosus	no predator	0.662	0	29	12	8.67	13.78076
D. villosus	no predator	0.662	0	29	13	8.64	5.960962
D. villosus	no predator	0.662	0	29	14	8.62	17.36187
D. villosus	no predator	0.662	0	29	15	8.61	10.32104
D. villosus	no predator	0.662	0	29	16	8.58	15.46225
D. villosus	no predator	0.662	0	29	17	8.57	18.38125
D. villosus	no predator	0.662	0	29	18	8.55	17.5763
D. villosus	no predator	0.662	0	29	19	8.52	5.818681
D. villosus	no predator	0.662	0	29	20	8.51	10.54543
D. villosus	no predator	0.662	0	29	21	8.48	12.73578
D. villosus	no predator	0.662	0	29	22	8.48	26.62729
D. villosus	no predator	0.662	0	29	23	8.46	20.5308
D. villosus	no predator	0.662	0	29	24	8.44	17.14306
D. villosus	no predator	0.662	0	29	25	8.42	13.67831
D. villosus	no predator	0.662	0	29	26	8.4	17.7433
D. villosus	no predator	0.662	0	29	27	8.37	9.498225
D. villosus	no predator	0.662	0	29	28	8.35	11.25809
D. villosus	no predator	0.662	0	29	29	8.32	11.74758
D. villosus	no predator	0.662	0	29	30	8.31	8.776138

Gammarid species	treatment	weight [g]	pre-exposure time	trial	time [min.]	oxygen level [mg/l]	activity
D. villosus	no predator	0.5427	0	30	1	8.83	1.379987
D. villosus	no predator	0.5427	0	30	2	8.8	1.299987
D. villosus	no predator	0.5427	0	30	3	8.78	7.53992
D. villosus	no predator	0.5427	0	30	4	8.76	1.05999
D. villosus	no predator	0.5427	0	30	5	8.75	5.05995
D. villosus	no predator	0.5427	0	30	6	8.75	0.759993
D. villosus	no predator	0.5427	0	30	7	8.74	2.499975
D. villosus	no predator	0.5427	0	30	8	8.71	1.601305
D. villosus	no predator	0.5427	0	30	9	8.7	5.078629
D. villosus	no predator	0.5427	0	30	10	8.7	8.482251
D. villosus	no predator	0.5427	0	30	11	8.68	6.877595
D. villosus	no predator	0.5427	0	30	12	8.68	0.800893
D. villosus	no predator	0.5427	0	30	13	8.66	5.921921
D. villosus	no predator	0.5427	0	30	14	8.67	3.777082
D. villosus	no predator	0.5427	0	30	15	8.65	2.561055
D. villosus	no predator	0.5427	0	30	16	8.63	3.680023
D. villosus	no predator	0.5427	0	30	17	8.64	11.96235
D. villosus	no predator	0.5427	0	30	18	8.62	2.716372
D. villosus	no predator	0.5427	0	30	19	8.6	2.283936
D. villosus	no predator	0.5427	0	30	20	8.58	3.178708
D. villosus	no predator	0.5427	0	30	21	8.58	2.120039
D. villosus	no predator	0.5427	0	30	22	8.55	4.918512
D. villosus	no predator	0.5427	0	30	23	8.52	3.519966
D. villosus	no predator	0.5427	0	30	24	8.51	8.779905
D. villosus	no predator	0.5427	0	30	25	8.51	12.16492
D. villosus	no predator	0.5427	0	30	26	8.5	5.776582
D. villosus	no predator	0.5427	0	30	27	8.49	12.21989
D. villosus	no predator	0.5427	0	30	28	8.48	4.739954
D. villosus	no predator	0.5427	0	30	29	8.45	0.919991
D. villosus	no predator	0.5427	0	30	30	8.44	2.619975
D. villosus	no predator	0.5369	0	31	1	7.82	2.899972
D. villosus	no predator	0.5369	0	31	2	8	3.680265
D. villosus	no predator	0.5369	0	31	3	8.21	1.139689
D. villosus	no predator	0.5369	0	31	4	8.33	0.419996
D. villosus	no predator	0.5369	0	31	5	8.41	0.02
D. villosus	no predator	0.5369	0	31	6	8.21	1.580524
D. villosus	no predator	0.5369	0	31	7	7.83	2.439976
D. villosus	no predator	0.5369	0	31	8	7.93	2.159979
D. villosus	no predator	0.5369	0	31	9	7.66	1.199988
D. villosus	no predator	0.5369	0	31	10	7.88	0
D. villosus	no predator	0.5369	0	31	11	7.95	1.319986
D. villosus	no predator	0.5369	0	31	12	8.09	0.159998
D. villosus	no predator	0.5369	0	31	13	7.8	0.02
D. villosus	no predator	0.5369	0	31	14	7.73	1.499985
D. villosus	no predator	0.5369	0	31	15	7.5	1.079989
D. villosus	no predator	0.5369	0	31	16	7.65	0.99999
D. villosus	no predator	0.5369	0	31	17	7.57	0.899991
D. villosus	no predator	0.5369	0	31	18	7.83	0.559994

Gammarid species	treatment	weight [g]	pre-exposure time	trial	time [min.]	oxygen level [mg/l]	activity
D. villosus	no predator	0.5369	0	31	19	8.04	2.219978
D. villosus	no predator	0.5369	0	31	20	8.2	0
D. villosus	no predator	0.5369	0	31	21	8.25	1.881421
D. villosus	no predator	0.5369	0	31	22	7.93	2.504476
D. villosus	no predator	0.5369	0	31	23	7.99	3.135469
D. villosus	no predator	0.5369	0	31	24	7.87	4.479956
D. villosus	no predator	0.5369	0	31	25	7.38	0.439995
D. villosus	no predator	0.5369	0	31	26	7.17	3.679963
D. villosus	no predator	0.5369	0	31	27	7.43	1.239988
D. villosus	no predator	0.5369	0	31	28	7.51	4.88181
D. villosus	no predator	0.5369	0	31	29	7.63	2.238118
D. villosus	no predator	0.5369	0	31	30	7.77	2.099979
D. villosus	no predator	0.6584	0	32	1	8.81	9.000885
D. villosus	no predator	0.6584	0	32	2	8.8	6.878976
D. villosus	no predator	0.6584	0	32	3	8.77	4.35996
D. villosus	no predator	0.6584	0	32	4	8.76	8.920334
D. villosus	no predator	0.6584	0	32	5	8.72	3.159548
D. villosus	no predator	0.6584	0	32	6	8.71	7.241013
D. villosus	no predator	0.6584	0	32	7	8.69	7.018851
D. villosus	no predator	0.6584	0	32	8	8.68	2.500635
D. villosus	no predator	0.6584	0	32	9	8.68	7.019999
D. villosus	no predator	0.6584	0	32	10	8.65	3.439244
D. villosus	no predator	0.6584	0	32	11	8.64	4.244159
D. villosus	no predator	0.6584	0	32	12	8.61	5.81659
D. villosus	no predator	0.6584	0	32	13	8.58	3.559965
D. villosus	no predator	0.6584	0	32	14	8.57	4.240979
D. villosus	no predator	0.6584	0	32	15	8.56	5.681085
D. villosus	no predator	0.6584	0	32	16	8.55	6.498915
D. villosus	no predator	0.6584	0	32	17	8.52	9.364708
D. villosus	no predator	0.6584	0	32	18	8.51	12.23509
D. villosus	no predator	0.6584	0	32	19	8.49	8.202562
D. villosus	no predator	0.6584	0	32	20	8.49	10.65725
D. villosus	no predator	0.6584	0	32	21	8.46	12.4242
D. villosus	no predator	0.6584	0	32	22	8.44	4.638573
D. villosus	no predator	0.6584	0	32	23	8.42	7.421552
D. villosus	no predator	0.6584	0	32	24	8.4	5.300008
D. villosus	no predator	0.6584	0	32	25	8.38	5.061691
D. villosus	no predator	0.6584	0	32	26	8.36	15.33649
D. villosus	no predator	0.6584	0	32	27	8.37	5.679948
D. villosus	no predator	0.6584	0	32	28	8.34	6.321799
D. villosus	no predator	0.6584	0	32	29	8.31	7.64382
D. villosus	no predator	0.6584	0	32	30	8.3	8.776122
D. villosus	no predator	0.6869	0	33	1	8.83	6.739817
D. villosus	no predator	0.6869	0	33	2	8.8	2.820574
D. villosus	no predator	0.6869	0	33	3	8.77	9.219612
D. villosus	no predator	0.6869	0	33	4	8.74	4.980788
D. villosus	no predator	0.6869	0	33	5	8.7	6.499097
D. villosus	no predator	0.6869	0	33	6	8.67	3.840501

Gammarid species	treatment	weight [g]	pre-exposure time	trial	time [min.]	oxygen level [mg/l]	activity
D. villosus	no predator	0.6869	0	33	7	8.65	4.560015
D. villosus	no predator	0.6869	0	33	8	8.63	3.159968
D. villosus	no predator	0.6869	0	33	9	8.63	9.839898
D. villosus	no predator	0.6869	0	33	10	8.61	2.659973
D. villosus	no predator	0.6869	0	33	11	8.58	4.819955
D. villosus	no predator	0.6869	0	33	12	8.58	2.859972
D. villosus	no predator	0.6869	0	33	13	8.57	3.359964
D. villosus	no predator	0.6869	0	33	14	8.55	0.941011
D. villosus	no predator	0.6869	0	33	15	8.53	6.678912
D. villosus	no predator	0.6869	0	33	16	8.5	4.839953
D. villosus	no predator	0.6869	0	33	17	8.49	4.141159
D. villosus	no predator	0.6869	0	33	18	8.48	3.739963
D. villosus	no predator	0.6869	0	33	19	8.45	7.341253
D. villosus	no predator	0.6869	0	33	20	8.43	3.538646
D. villosus	no predator	0.6869	0	33	21	8.42	5.619939
D. villosus	no predator	0.6869	0	33	22	8.39	2.661475
D. villosus	no predator	0.6869	0	33	23	8.37	2.96003
D. villosus	no predator	0.6869	0	33	24	8.35	5.340002
D. villosus	no predator	0.6869	0	33	25	8.35	10.34164
D. villosus	no predator	0.6869	0	33	26	8.33	10.95653
D. villosus	no predator	0.6869	0	33	27	8.31	4.899947
D. villosus	no predator	0.6869	0	33	28	8.3	5.859949
D. villosus	no predator	0.6869	0	33	29	8.29	2.099979
D. villosus	no predator	0.6869	0	33	30	8.26	3.99996
D. villosus	no predator	0.818	0	34	1	8.56	7.499922
D. villosus	no predator	0.818	0	34	2	8.29	2.159981
D. villosus	no predator	0.818	0	34	3	8.48	2.1007
D. villosus	no predator	0.818	0	34	4	8.44	3.020089
D. villosus	no predator	0.818	0	34	5	8.49	4.859592
D. villosus	no predator	0.818	0	34	6	8.52	3.499489
D. villosus	no predator	0.818	0	34	7	8.54	2.340577
D. villosus	no predator	0.818	0	34	8	8.44	2.619374
D. villosus	no predator	0.818	0	34	9	8.17	5.259953
D. villosus	no predator	0.818	0	34	10	8.19	8.802259
D. villosus	no predator	0.818	0	34	11	8.26	7.677579
D. villosus	no predator	0.818	0	34	12	8.23	7.319925
D. villosus	no predator	0.818	0	34	13	8.17	3.579964
D. villosus	no predator	0.818	0	34	14	8.03	2.880992
D. villosus	no predator	0.818	0	34	15	8.05	8.138903
D. villosus	no predator	0.818	0	34	16	8.04	4.799961
D. villosus	no predator	0.818	0	34	17	7.97	5.602344
D. villosus	no predator	0.818	0	34	18	8.14	6.637528
D. villosus	no predator	0.818	0	34	19	8.34	10.54517
D. villosus	no predator	0.818	0	34	20	8.34	7.881537
D. villosus	no predator	0.818	0	34	21	8.34	11.63299
D. villosus	no predator	0.818	0	34	22	8.34	10.70139
D. villosus	no predator	0.818	0	34	23	8.33	8.938411
D. villosus	no predator	0.818	0	34	24	8.33	10.2399

Gammarid species	treatment	weight [g]	pre-exposure time	trial	time [min.]	oxygen level [mg/l]	activity
D. villosus	no predator	0.818	0	34	25	8.29	10.2399
D. villosus	no predator	0.818	0	34	26	8.28	4.859956
D. villosus	no predator	0.818	0	34	27	8.27	9.107107
D. villosus	no predator	0.818	0	34	28	8.23	13.67639
D. villosus	no predator	0.818	0	34	29	8.22	6.856211
D. villosus	no predator	0.818	0	34	30	8.19	4.079959
D. villosus	no predator	0.7752	0	35	1	8.7	4.000199
D. villosus	no predator	0.7752	0	35	2	8.68	8.30058
D. villosus	no predator	0.7752	0	35	3	8.65	14.47896
D. villosus	no predator	0.7752	0	35	4	8.65	4.381638
D. villosus	no predator	0.7752	0	35	5	8.63	10.43918
D. villosus	no predator	0.7752	0	35	6	8.62	13.37944
D. villosus	no predator	0.7752	0	35	7	8.6	5.720607
D. villosus	no predator	0.7752	0	35	8	8.57	6.579398
D. villosus	no predator	0.7752	0	35	9	8.56	6.280662
D. villosus	no predator	0.7752	0	35	10	8.51	11.18229
D. villosus	no predator	0.7752	0	35	11	8.49	7.818489
D. villosus	no predator	0.7752	0	35	12	8.45	8.300939
D. villosus	no predator	0.7752	0	35	13	8.43	15.68193
D. villosus	no predator	0.7752	0	35	14	8.42	14.93914
D. villosus	no predator	0.7752	0	35	15	8.4	5.796942
D. villosus	no predator	0.7752	0	35	16	8.38	14.32105
D. villosus	no predator	0.7752	0	35	17	8.36	14.54358
D. villosus	no predator	0.7752	0	35	18	8.35	13.47639
D. villosus	no predator	0.7752	0	35	19	8.32	6.798735
D. villosus	no predator	0.7752	0	35	20	8.32	9.878586
D. villosus	no predator	0.7752	0	35	21	8.3	6.701368
D. villosus	no predator	0.7752	0	35	22	8.28	10.05995
D. villosus	no predator	0.7752	0	35	23	8.27	9.741458
D. villosus	no predator	0.7752	0	35	24	8.24	20.48471
D. villosus	no predator	0.7752	0	35	25	8.2	6.21346
D. villosus	no predator	0.7752	0	35	26	8.19	9.126868
D. villosus	no predator	0.7752	0	35	27	8.18	9.496553
D. villosus	no predator	0.7752	0	35	28	8.15	11.98181
D. villosus	no predator	0.7752	0	35	29	8.12	12.91615
D. villosus	no predator	0.7752	0	35	30	8.1	9.259912
D. villosus	no predator	0.642	0	36	1	8.49	6.960174
D. villosus	no predator	0.642	0	36	2	8.57	1.799983
D. villosus	no predator	0.642	0	36	3	8.65	3.279968
D. villosus	no predator	0.642	0	36	4	8.7	1.441246
D. villosus	no predator	0.642	0	36	5	8.72	8.799613
D. villosus	no predator	0.642	0	36	6	8.65	1.679503
D. villosus	no predator	0.642	0	36	7	8.59	3.799961
D. villosus	no predator	0.642	0	36	8	8.49	6.059938
D. villosus	no predator	0.642	0	36	9	8.18	2.119981
D. villosus	no predator	0.642	0	36	10	8.19	3.21997
D. villosus	no predator	0.642	0	36	11	8.29	2.140819
D. villosus	no predator	0.642	0	36	12	8.39	3.899962

Gammarid species	treatment	weight [g]	pre-exposure time	trial	time [min.]	oxygen level [mg/l]	activity
D. villosus	no predator	0.642	0	36	13	8.49	0.539994
D. villosus	no predator	0.642	0	36	14	8.46	3.042011
D. villosus	no predator	0.642	0	36	15	8.44	2.94009
D. villosus	no predator	0.642	0	36	16	8.4	5.737782
D. villosus	no predator	0.642	0	36	17	8.41	4.12236
D. villosus	no predator	0.642	0	36	18	8.27	4.263799
D. villosus	no predator	0.642	0	36	19	8.21	3.574925
D. villosus	no predator	0.642	0	36	20	8.27	2.379976
D. villosus	no predator	0.642	0	36	21	8.21	2.561415
D. villosus	no predator	0.642	0	36	22	8.18	1.078549
D. villosus	no predator	0.642	0	36	23	8.08	3.94152
D. villosus	no predator	0.642	0	36	24	8.17	4.341637
D. villosus	no predator	0.642	0	36	25	8.25	17.00162
D. villosus	no predator	0.642	0	36	26	8.28	9.354876
D. villosus	no predator	0.642	0	36	27	8.22	7.739916
D. villosus	no predator	0.642	0	36	28	8.2	6.259941
D. villosus	no predator	0.642	0	36	29	8.18	3.185728
D. villosus	no predator	0.642	0	36	30	8.18	11.41605
D. villosus	no predator	0.6956	0	37	1	8.49	6.360417
D. villosus	no predator	0.6956	0	37	2	8.36	2.200398
D. villosus	no predator	0.6956	0	37	3	8.4	1.579384
D. villosus	no predator	0.6956	0	37	4	8.42	1.299986
D. villosus	no predator	0.6956	0	37	5	8.36	7.219935
D. villosus	no predator	0.6956	0	37	6	8.2	1.659984
D. villosus	no predator	0.6956	0	37	7	8.26	1.519985
D. villosus	no predator	0.6956	0	37	8	8.29	1.239988
D. villosus	no predator	0.6956	0	37	9	8.31	1.459985
D. villosus	no predator	0.6956	0	37	10	8.32	0.439995
D. villosus	no predator	0.6956	0	37	11	8.3	2.739973
D. villosus	no predator	0.6956	0	37	12	8.32	2.159978
D. villosus	no predator	0.6956	0	37	13	8.31	1.159988
D. villosus	no predator	0.6956	0	37	14	8.29	6.800949
D. villosus	no predator	0.6956	0	37	15	8.29	5.218925
D. villosus	no predator	0.6956	0	37	16	8.27	4.123385
D. villosus	no predator	0.6956	0	37	17	8.25	13.30365
D. villosus	no predator	0.6956	0	37	18	8.25	10.3527
D. villosus	no predator	0.6956	0	37	19	8.23	1.922621
D. villosus	no predator	0.6956	0	37	20	8.23	2.278718
D. villosus	no predator	0.6956	0	37	21	8.23	4.664278
D. villosus	no predator	0.6956	0	37	22	8.21	6.93561
D. villosus	no predator	0.6956	0	37	23	8.19	3.899962
D. villosus	no predator	0.6956	0	37	24	8.18	6.659935
D. villosus	no predator	0.6956	0	37	25	8.14	0.379997
D. villosus	no predator	0.6956	0	37	26	8.14	3.64344
D. villosus	no predator	0.6956	0	37	27	8.12	3.216487
D. villosus	no predator	0.6956	0	37	28	8.11	0.139999
D. villosus	no predator	0.6956	0	37	29	8.11	0.779992
D. villosus	no predator	0.6956	0	37	30	8.09	1.201968

Gammarid species	treatment	weight [g]	pre-exposure time	trial	time [min.]	oxygen level [mg/l]	activity
D. villosus	no predator	0.6141	0	38	1	8.6	2.659794
D. villosus	no predator	0.6141	0	38	2	8.57	4.540557
D. villosus	no predator	0.6141	0	38	3	8.57	7.600048
D. villosus	no predator	0.6141	0	38	4	8.54	17.30037
D. villosus	no predator	0.6141	0	38	5	8.51	19.60093
D. villosus	no predator	0.6141	0	38	6	8.49	21.27792
D. villosus	no predator	0.6141	0	38	7	8.45	13.44293
D. villosus	no predator	0.6141	0	38	8	8.42	10.49755
D. villosus	no predator	0.6141	0	38	9	8.42	2.980031
D. villosus	no predator	0.6141	0	38	10	8.4	4.35924
D. villosus	no predator	0.6141	0	38	11	8.4	5.279944
D. villosus	no predator	0.6141	0	38	12	8.39	6.999933
D. villosus	no predator	0.6141	0	38	13	8.38	9.24375
D. villosus	no predator	0.6141	0	38	14	8.36	16.45702
D. villosus	no predator	0.6141	0	38	15	8.35	5.14001
D. villosus	no predator	0.6141	0	38	16	8.34	5.518868
D. villosus	no predator	0.6141	0	38	17	8.29	4.962351
D. villosus	no predator	0.6141	0	38	18	8.26	3.118771
D. villosus	no predator	0.6141	0	38	19	8.23	2.401297
D. villosus	no predator	0.6141	0	38	20	8.23	4.839953
D. villosus	no predator	0.6141	0	38	21	8.22	3.104288
D. villosus	no predator	0.6141	0	38	22	8.2	14.34448
D. villosus	no predator	0.6141	0	38	23	8.19	7.797101
D. villosus	no predator	0.6141	0	38	24	8.18	6.756873
D. villosus	no predator	0.6141	0	38	25	8.16	5.826724
D. villosus	no predator	0.6141	0	38	26	8.15	4.963672
D. villosus	no predator	0.6141	0	38	27	8.13	10.33486
D. villosus	no predator	0.6141	0	38	28	8.15	4.674553
D. villosus	no predator	0.6141	0	38	29	8.14	3.199968
D. villosus	no predator	0.6141	0	38	30	8.12	8.61992
D. villosus	no predator	0.7222	0	39	1	8.59	6.019935
D. villosus	no predator	0.7222	0	39	2	8.56	6.019939
D. villosus	no predator	0.7222	0	39	3	8.53	1.701423
D. villosus	no predator	0.7222	0	39	4	8.51	6.498921
D. villosus	no predator	0.7222	0	39	5	8.5	6.319518
D. villosus	no predator	0.7222	0	39	6	8.48	4.841029
D. villosus	no predator	0.7222	0	39	7	8.48	5.119955
D. villosus	no predator	0.7222	0	39	8	8.46	6.401256
D. villosus	no predator	0.7222	0	39	9	8.44	12.70139
D. villosus	no predator	0.7222	0	39	10	8.42	3.458525
D. villosus	no predator	0.7222	0	39	11	8.4	2.119979
D. villosus	no predator	0.7222	0	39	12	8.37	4.12086
D. villosus	no predator	0.7222	0	39	13	8.36	5.84282
D. villosus	no predator	0.7222	0	39	14	8.35	6.037064
D. villosus	no predator	0.7222	0	39	15	8.33	3.501043
D. villosus	no predator	0.7222	0	39	16	8.31	11.02332
D. villosus	no predator	0.7222	0	39	17	8.3	5.17767
D. villosus	no predator	0.7222	0	39	18	8.28	1.221249

Gammarid species	treatment	weight [g]	pre-exposure time	trial	time [min.]	oxygen level [mg/l]	activity
D. villosus	no predator	0.7222	0	39	19	8.26	9.881287
D. villosus	no predator	0.7222	0	39	20	8.25	2.761473
D. villosus	no predator	0.7222	0	39	21	8.23	5.735801
D. villosus	no predator	0.7222	0	39	22	8.23	5.461446
D. villosus	no predator	0.7222	0	39	23	8.21	7.41999
D. villosus	no predator	0.7222	0	39	24	8.2	5.978379
D. villosus	no predator	0.7222	0	39	25	8.18	6.899933
D. villosus	no predator	0.7222	0	39	26	8.16	7.665146
D. villosus	no predator	0.7222	0	39	27	8.14	8.854687
D. villosus	no predator	0.7222	0	39	28	8.14	4.36368
D. villosus	no predator	0.7222	0	39	29	8.14	9.176197
D. villosus	no predator	0.7222	0	39	30	8.13	3.761942
D. villosus	no predator	0.575	0	40	1	8.47	11.15977
D. villosus	no predator	0.575	0	40	2	8.48	8.979682
D. villosus	no predator	0.575	0	40	3	8.44	5.539954
D. villosus	no predator	0.575	0	40	4	8.45	12.28198
D. villosus	no predator	0.575	0	40	5	8.43	13.47823
D. villosus	no predator	0.575	0	40	6	8.43	1.319506
D. villosus	no predator	0.575	0	40	7	8.41	2.919974
D. villosus	no predator	0.575	0	40	8	8.4	1.780642
D. villosus	no predator	0.575	0	40	9	8.39	0.539335
D. villosus	no predator	0.575	0	40	10	8.38	4.739954
D. villosus	no predator	0.575	0	40	11	8.35	2.679973
D. villosus	no predator	0.575	0	40	12	8.32	1.781783
D. villosus	no predator	0.575	0	40	13	8.32	3.778163
D. villosus	no predator	0.575	0	40	14	8.3	4.09996
D. villosus	no predator	0.575	0	40	15	8.3	2.459974
D. villosus	no predator	0.575	0	40	16	8.3	4.659955
D. villosus	no predator	0.575	0	40	17	8.28	5.464749
D. villosus	no predator	0.575	0	40	18	8.25	8.636389
D. villosus	no predator	0.575	0	40	19	8.24	5.218691
D. villosus	no predator	0.575	0	40	20	8.24	6.182707
D. villosus	no predator	0.575	0	40	21	8.22	5.637183
D. villosus	no predator	0.575	0	40	22	8.21	1.521485
D. villosus	no predator	0.575	0	40	23	8.22	5.698445
D. villosus	no predator	0.575	0	40	24	8.2	8.139916
D. villosus	no predator	0.575	0	40	25	8.2	6.241614
D. villosus	no predator	0.575	0	40	26	8.18	3.358289
D. villosus	no predator	0.575	0	40	27	8.17	5.547146
D. villosus	no predator	0.575	0	40	28	8.16	3.912761
D. villosus	no predator	0.575	0	40	29	8.12	2.519974
D. villosus	no predator	0.575	0	40	30	8.11	9.939902
D. villosus	no predator	0.7961	0	41	1	8.48	1.400226
D. villosus	no predator	0.7961	0	41	2	8.45	2.359736
D. villosus	no predator	0.7961	0	41	3	8.45	3.359967
D. villosus	no predator	0.7961	0	41	4	8.43	2.841231
D. villosus	no predator	0.7961	0	41	5	8.41	14.79859
D. villosus	no predator	0.7961	0	41	6	8.38	4.019964

Gammarid species	treatment	weight [g]	pre-exposure time	trial	time [min.]	oxygen level [mg/l]	activity
D. villosus	no predator	0.7961	0	41	7	8.34	3.039969
D. villosus	no predator	0.7961	0	41	8	8.32	2.88129
D. villosus	no predator	0.7961	0	41	9	8.3	5.299289
D. villosus	no predator	0.7961	0	41	10	8.3	4.501519
D. villosus	no predator	0.7961	0	41	11	8.29	4.818393
D. villosus	no predator	0.7961	0	41	12	8.27	2.139979
D. villosus	no predator	0.7961	0	41	13	8.26	2.379977
D. villosus	no predator	0.7961	0	41	14	8.24	2.242017
D. villosus	no predator	0.7961	0	41	15	8.22	8.739978
D. villosus	no predator	0.7961	0	41	16	8.21	0.718913
D. villosus	no predator	0.7961	0	41	17	8.18	7.781132
D. villosus	no predator	0.7961	0	41	18	8.15	2.378776
D. villosus	no predator	0.7961	0	41	19	8.14	3.261289
D. villosus	no predator	0.7961	0	41	20	8.14	1.03867
D. villosus	no predator	0.7961	0	41	21	8.1	4.561395
D. villosus	no predator	0.7961	0	41	22	8.07	3.021531
D. villosus	no predator	0.7961	0	41	23	8.02	4.036959
D. villosus	no predator	0.7961	0	41	24	8.04	0.599994
D. villosus	no predator	0.7961	0	41	25	8.02	10.57989
D. villosus	no predator	0.7961	0	41	26	8	1.361726
D. villosus	no predator	0.7961	0	41	27	7.99	1.759983
D. villosus	no predator	0.7961	0	41	28	7.98	1.739983
D. villosus	no predator	0.7961	0	41	29	7.96	1.363826
D. villosus	no predator	0.7961	0	41	30	7.95	3.436126
D. villosus	no predator	0.649	0	42	1	8.51	9.680628
D. villosus	no predator	0.649	0	42	2	8.47	10.23918
D. villosus	no predator	0.649	0	42	3	8.45	7.139937
D. villosus	no predator	0.649	0	42	4	8.4	7.700348
D. villosus	no predator	0.649	0	42	5	8.41	9.07997
D. villosus	no predator	0.649	0	42	6	8.39	3.241108
D. villosus	no predator	0.649	0	42	7	8.36	13.57937
D. villosus	no predator	0.649	0	42	8	8.35	10.53929
D. villosus	no predator	0.649	0	42	9	8.33	6.679933
D. villosus	no predator	0.649	0	42	10	8.3	4.741511
D. villosus	no predator	0.649	0	42	11	8.28	4.178399
D. villosus	no predator	0.649	0	42	12	8.26	5.321747
D. villosus	no predator	0.649	0	42	13	8.24	3.439126
D. villosus	no predator	0.649	0	42	14	8.24	4.920012
D. villosus	no predator	0.649	0	42	15	8.2	7.321064
D. villosus	no predator	0.649	0	42	16	8.19	2.55782
D. villosus	no predator	0.649	0	42	17	8.18	5.781142
D. villosus	no predator	0.649	0	42	18	8.16	5.240002
D. villosus	no predator	0.649	0	42	19	8.15	4.378695
D. villosus	no predator	0.649	0	42	20	8.13	9.419898
D. villosus	no predator	0.649	0	42	21	8.11	2.279976
D. villosus	no predator	0.649	0	42	22	8.1	0.639993
D. villosus	no predator	0.649	0	42	23	8.09	1.599985
D. villosus	no predator	0.649	0	42	24	8.06	1.141609

Gammarid species	treatment	weight [g]	pre-exposure time	trial	time [min.]	oxygen level [mg/l]	activity
D. villosus	no predator	0.649	0	42	25	8.05	4.33834
D. villosus	no predator	0.649	0	42	26	8.03	2.159979
D. villosus	no predator	0.649	0	42	27	8.01	0.279997
D. villosus	no predator	0.649	0	42	28	8	2.919971
D. villosus	no predator	0.649	0	42	29	7.98	0.93999
D. villosus	no predator	0.649	0	42	30	7.98	2.94393
D. villosus	no predator	0.5817	0	43	1	8.53	3.09943
D. villosus	no predator	0.5817	0	43	2	8.49	2.139977
D. villosus	no predator	0.5817	0	43	3	8.46	1.779983
D. villosus	no predator	0.5817	0	43	4	8.42	0
D. villosus	no predator	0.5817	0	43	5	8.4	1.339988
D. villosus	no predator	0.5817	0	43	6	8.39	3.861041
D. villosus	no predator	0.5817	0	43	7	8.37	2.678893
D. villosus	no predator	0.5817	0	43	8	8.37	0.719992
D. villosus	no predator	0.5817	0	43	9	8.35	2.319976
D. villosus	no predator	0.5817	0	43	10	8.33	6.75994
D. villosus	no predator	0.5817	0	43	11	8.31	0.799992
D. villosus	no predator	0.5817	0	43	12	8.29	1.499985
D. villosus	no predator	0.5817	0	43	13	8.28	1.459985
D. villosus	no predator	0.5817	0	43	14	8.27	0.379996
D. villosus	no predator	0.5817	0	43	15	8.26	2.142139
D. villosus	no predator	0.5817	0	43	16	8.24	0.99783
D. villosus	no predator	0.5817	0	43	17	8.23	0.919992
D. villosus	no predator	0.5817	0	43	18	8.2	0.259997
D. villosus	no predator	0.5817	0	43	19	8.19	3.059969
D. villosus	no predator	0.5817	0	43	20	8.17	1.919981
D. villosus	no predator	0.5817	0	43	21	8.15	6.06139
D. villosus	no predator	0.5817	0	43	22	8.12	2.138539
D. villosus	no predator	0.5817	0	43	23	8.1	1.359986
D. villosus	no predator	0.5817	0	43	24	8.1	2.219979
D. villosus	no predator	0.5817	0	43	25	8.08	8.661593
D. villosus	no predator	0.5817	0	43	26	8.07	4.819954
D. villosus	no predator	0.5817	0	43	27	8.04	0.759992
D. villosus	no predator	0.5817	0	43	28	8.04	2.901832
D. villosus	no predator	0.5817	0	43	29	8.02	1.958121
D. villosus	no predator	0.5817	0	43	30	8	2.221958
D. villosus	no predator	0.5899	0	44	1	8.47	1.260226
D. villosus	no predator	0.5899	0	44	2	8.44	2.13974
D. villosus	no predator	0.5899	0	44	3	8.42	1.419986
D. villosus	no predator	0.5899	0	44	4	8.41	2.220398
D. villosus	no predator	0.5899	0	44	5	8.41	3.519545
D. villosus	no predator	0.5899	0	44	6	8.39	1.900521
D. villosus	no predator	0.5899	0	44	7	8.37	0.299457
D. villosus	no predator	0.5899	0	44	8	8.36	1.239988
D. villosus	no predator	0.5899	0	44	9	8.33	3.342128
D. villosus	no predator	0.5899	0	44	10	8.29	5.497785
D. villosus	no predator	0.5899	0	44	11	8.3	4.981627
D. villosus	no predator	0.5899	0	44	12	8.29	3.320927

Gammarid species	treatment	weight [g]	pre-exposure time	trial	time [min.]	oxygen level [mg/l]	activity
D. villosus	no predator	0.5899	0	44	13	8.25	6.63994
D. villosus	no predator	0.5899	0	44	14	8.24	1.419986
D. villosus	no predator	0.5899	0	44	15	8.22	3.619963
D. villosus	no predator	0.5899	0	44	16	8.21	2.339977
D. villosus	no predator	0.5899	0	44	17	8.19	4.381157
D. villosus	no predator	0.5899	0	44	18	8.17	7.258725
D. villosus	no predator	0.5899	0	44	19	8.18	1.159988
D. villosus	no predator	0.5899	0	44	20	8.18	8.648192
D. villosus	no predator	0.5899	0	44	21	8.16	11.09732
D. villosus	no predator	0.5899	0	44	22	8.15	17.86002
D. villosus	no predator	0.5899	0	44	23	8.13	4.655456
D. villosus	no predator	0.5899	0	44	24	8.09	0.759992
D. villosus	no predator	0.5899	0	44	25	8.09	1.559984
D. villosus	no predator	0.5899	0	44	26	8.08	1.599984
D. villosus	no predator	0.5899	0	44	27	8.05	7.708925
D. villosus	no predator	0.5899	0	44	28	8.01	7.430926
D. villosus	no predator	0.5899	0	44	29	8	2.321896
D. villosus	no predator	0.5899	0	44	30	7.99	5.659945
G. jazdzewski	no predator	0.1519	0	48	1	8.61	19.86063
G. jazdzewski	no predator	0.1519	0	48	2	8.62	19.94011
G. jazdzewski	no predator	0.1519	0	48	3	8.64	20.45974
G. jazdzewski	no predator	0.1519	0	48	4	8.64	22.72169
G. jazdzewski	no predator	0.1519	0	48	5	8.64	22.69826
G. jazdzewski	no predator	0.1519	0	48	6	8.64	19.1816
G. jazdzewski	no predator	0.1519	0	48	7	8.64	23.65945
G. jazdzewski	no predator	0.1519	0	48	8	8.66	28.08194
G. jazdzewski	no predator	0.1519	0	48	9	8.66	24.35933
G. jazdzewski	no predator	0.1519	0	48	10	8.66	24.12
G. jazdzewski	no predator	0.1519	0	48	11	8.65	24.65914
G. jazdzewski	no predator	0.1519	0	48	12	8.64	23.07996
G. jazdzewski	no predator	0.1519	0	48	13	8.63	23.08092
G. jazdzewski	no predator	0.1519	0	48	14	8.63	18.32108
G. jazdzewski	no predator	0.1519	0	48	15	8.62	20.23793
G. jazdzewski	no predator	0.1519	0	48	16	8.6	16.84115
G. jazdzewski	no predator	0.1519	0	48	17	8.59	16.19768
G. jazdzewski	no predator	0.1519	0	48	18	8.59	15.70248
G. jazdzewski	no predator	0.1519	0	48	19	8.6	16.63875
G. jazdzewski	no predator	0.1519	0	48	20	8.6	22.34267
G. jazdzewski	no predator	0.1519	0	48	21	8.59	24.03999
G. jazdzewski	no predator	0.1519	0	48	22	8.58	23.66002
G. jazdzewski	no predator	0.1519	0	48	23	8.59	26.89686
G. jazdzewski	no predator	0.1519	0	48	24	8.57	26.02634
G. jazdzewski	no predator	0.1519	0	48	25	8.57	26.05844
G. jazdzewski	no predator	0.1519	0	48	26	8.57	25.16179
G. jazdzewski	no predator	0.1519	0	48	27	8.59	25.71831
G. jazdzewski	no predator	0.1519	0	48	28	8.58	24.15821
G. jazdzewski	no predator	0.1519	0	48	29	8.57	20.74573
G. jazdzewski	no predator	0.1519	0	48	30	8.57	24.99418

Gammarid species	treatment	weight [g]	pre-exposure time	trial	time [min.]	oxygen level [mg/l]	activity
G. jazdzewski	no predator	0.1289	0	49	1	8.48	39.00135
G. jazdzewski	no predator	0.1289	0	49	2	8.48	44.06026
G. jazdzewski	no predator	0.1289	0	49	3	8.47	43.51927
G. jazdzewski	no predator	0.1289	0	49	4	8.46	41.32074
G. jazdzewski	no predator	0.1289	0	49	5	8.48	37.44005
G. jazdzewski	no predator	0.1289	0	49	6	8.49	39.5195
G. jazdzewski	no predator	0.1289	0	49	7	8.47	43.73931
G. jazdzewski	no predator	0.1289	0	49	8	8.47	40.07924
G. jazdzewski	no predator	0.1289	0	49	9	8.45	41.26271
G. jazdzewski	no predator	0.1289	0	49	10	8.46	43.86069
G. jazdzewski	no predator	0.1289	0	49	11	8.46	40.98155
G. jazdzewski	no predator	0.1289	0	49	12	8.44	39.00002
G. jazdzewski	no predator	0.1289	0	49	13	8.44	38.98194
G. jazdzewski	no predator	0.1289	0	49	14	8.44	38.2981
G. jazdzewski	no predator	0.1289	0	49	15	8.43	44.22105
G. jazdzewski	no predator	0.1289	0	49	16	8.41	44.38002
G. jazdzewski	no predator	0.1289	0	49	17	8.4	45.49996
G. jazdzewski	no predator	0.1289	0	49	18	8.4	46.92114
G. jazdzewski	no predator	0.1289	0	49	19	8.4	42.05603
G. jazdzewski	no predator	0.1289	0	49	20	8.39	40.13977
G. jazdzewski	no predator	0.1289	0	49	21	8.41	42.66695
G. jazdzewski	no predator	0.1289	0	49	22	8.39	41.64006
G. jazdzewski	no predator	0.1289	0	49	23	8.39	40.79697
G. jazdzewski	no predator	0.1289	0	49	24	8.38	38.74322
G. jazdzewski	no predator	0.1289	0	49	25	8.37	41.71504
G. jazdzewski	no predator	0.1289	0	49	26	8.37	42.77031
G. jazdzewski	no predator	0.1289	0	49	27	8.36	41.61304
G. jazdzewski	no predator	0.1289	0	49	28	8.33	41.28187
G. jazdzewski	no predator	0.1289	0	49	29	8.34	39.62003
G. jazdzewski	no predator	0.1289	0	49	30	8.35	39.85604
G. jazdzewski	no predator	0.1153	0	50	1	8.52	39.68032
G. jazdzewski	no predator	0.1153	0	50	2	8.5	40.60026
G. jazdzewski	no predator	0.1153	0	50	3	8.47	40.35893
G. jazdzewski	no predator	0.1153	0	50	4	8.46	39.00152
G. jazdzewski	no predator	0.1153	0	50	5	8.46	40.01852
G. jazdzewski	no predator	0.1153	0	50	6	8.44	33.86147
G. jazdzewski	no predator	0.1153	0	50	7	8.43	37.20114
G. jazdzewski	no predator	0.1153	0	50	8	8.44	39.80002
G. jazdzewski	no predator	0.1153	0	50	9	8.41	38.65992
G. jazdzewski	no predator	0.1153	0	50	10	8.4	39.2207
G. jazdzewski	no predator	0.1153	0	50	11	8.39	36.44251
G. jazdzewski	no predator	0.1153	0	50	12	8.37	36.15923
G. jazdzewski	no predator	0.1153	0	50	13	8.38	36.56096
G. jazdzewski	no predator	0.1153	0	50	14	8.37	37.53902
G. jazdzewski	no predator	0.1153	0	50	15	8.36	39.10308
G. jazdzewski	no predator	0.1153	0	50	16	8.36	40.14117
G. jazdzewski	no predator	0.1153	0	50	17	8.35	40.45408
G. jazdzewski	no predator	0.1153	0	50	18	8.34	35.66612

Gammarid species	treatment	weight [g]	pre-exposure time	trial	time [min.]	oxygen level [mg/l]	activity
G. jazdzewski	no predator	0.1153	0	50	19	8.33	37.19747
G. jazdzewski	no predator	0.1153	0	50	20	8.31	38.43999
G. jazdzewski	no predator	0.1153	0	50	21	8.31	36.65855
G. jazdzewski	no predator	0.1153	0	50	22	8.32	39.7369
G. jazdzewski	no predator	0.1153	0	50	23	8.3	36.84604
G. jazdzewski	no predator	0.1153	0	50	24	8.31	38.7584
G. jazdzewski	no predator	0.1153	0	50	25	8.31	38.7983
G. jazdzewski	no predator	0.1153	0	50	26	8.3	40.1486
G. jazdzewski	no predator	0.1153	0	50	27	8.31	35.19839
G. jazdzewski	no predator	0.1153	0	50	28	8.3	40.50176
G. jazdzewski	no predator	0.1153	0	50	29	8.29	41.60187
G. jazdzewski	no predator	0.1153	0	50	30	8.29	36.5561
G. jazdzewski	no predator	0.2237	0	51	1	8.41	34.08075
G. jazdzewski	no predator	0.2237	0	51	2	8.38	29.86005
G. jazdzewski	no predator	0.2237	0	51	3	8.37	31.26004
G. jazdzewski	no predator	0.2237	0	51	4	8.36	29.49873
G. jazdzewski	no predator	0.2237	0	51	5	8.36	33.18026
G. jazdzewski	no predator	0.2237	0	51	6	8.34	29.04044
G. jazdzewski	no predator	0.2237	0	51	7	8.33	29.28108
G. jazdzewski	no predator	0.2237	0	51	8	8.33	34.03996
G. jazdzewski	no predator	0.2237	0	51	9	8.31	40.07991
G. jazdzewski	no predator	0.2237	0	51	10	8.3	35.13917
G. jazdzewski	no predator	0.2237	0	51	11	8.3	30.74246
G. jazdzewski	no predator	0.2237	0	51	12	8.28	32.93831
G. jazdzewski	no predator	0.2237	0	51	13	8.29	34.77899
G. jazdzewski	no predator	0.2237	0	51	14	8.28	36.12091
G. jazdzewski	no predator	0.2237	0	51	15	8.26	32.52212
G. jazdzewski	no predator	0.2237	0	51	16	8.26	32.05784
G. jazdzewski	no predator	0.2237	0	51	17	8.26	33.98236
G. jazdzewski	no predator	0.2237	0	51	18	8.24	40.70121
G. jazdzewski	no predator	0.2237	0	51	19	8.24	39.98002
G. jazdzewski	no predator	0.2237	0	51	20	8.22	39.15452
G. jazdzewski	no predator	0.2237	0	51	21	8.21	35.90702
G. jazdzewski	no predator	0.2237	0	51	22	8.18	32.79853
G. jazdzewski	no predator	0.2237	0	51	23	8.18	36.20152
G. jazdzewski	no predator	0.2237	0	51	24	8.18	26.52005
G. jazdzewski	no predator	0.2237	0	51	25	8.18	27.5617
G. jazdzewski	no predator	0.2237	0	51	26	8.16	30.05658
G. jazdzewski	no predator	0.2237	0	51	27	8.14	29.64535
G. jazdzewski	no predator	0.2237	0	51	28	8.14	33.29265
G. jazdzewski	no predator	0.2237	0	51	29	8.11	29.52557
G. jazdzewski	no predator	0.2237	0	51	30	8.11	31.32592
G. jazdzewski	no predator	0.2464	0	52	1	8.48	45.7998
G. jazdzewski	no predator	0.2464	0	52	2	8.47	33.71977
G. jazdzewski	no predator	0.2464	0	52	3	8.45	36.62102
G. jazdzewski	no predator	0.2464	0	52	4	8.43	36.9801
G. jazdzewski	no predator	0.2464	0	52	5	8.43	44.59955
G. jazdzewski	no predator	0.2464	0	52	6	8.43	42.13994

Gammarid species	treatment	weight [g]	pre-exposure time	trial	time [min.]	oxygen level [mg/l]	activity
G. jazdzewski	no predator	0.2464	0	52	7	8.41	46.5793
G. jazdzewski	no predator	0.2464	0	52	8	8.39	44.02116
G. jazdzewski	no predator	0.2464	0	52	9	8.39	38.81861
G. jazdzewski	no predator	0.2464	0	52	10	8.36	41.66144
G. jazdzewski	no predator	0.2464	0	52	11	8.36	38.25915
G. jazdzewski	no predator	0.2464	0	52	12	8.35	40.4826
G. jazdzewski	no predator	0.2464	0	52	13	8.32	43.45998
G. jazdzewski	no predator	0.2464	0	52	14	8.32	42.99795
G. jazdzewski	no predator	0.2464	0	52	15	8.3	44.34526
G. jazdzewski	no predator	0.2464	0	52	16	8.29	41.97905
G. jazdzewski	no predator	0.2464	0	52	17	8.29	44.83882
G. jazdzewski	no predator	0.2464	0	52	18	8.27	41.45867
G. jazdzewski	no predator	0.2464	0	52	19	8.26	41.52118
G. jazdzewski	no predator	0.2464	0	52	20	8.25	38.73722
G. jazdzewski	no predator	0.2464	0	52	21	8.24	41.86264
G. jazdzewski	no predator	0.2464	0	52	22	8.22	41.75989
G. jazdzewski	no predator	0.2464	0	52	23	8.22	37.11992
G. jazdzewski	no predator	0.2464	0	52	24	8.21	38.12153
G. jazdzewski	no predator	0.2464	0	52	25	8.19	34.60332
G. jazdzewski	no predator	0.2464	0	52	26	8.18	41.91825
G. jazdzewski	no predator	0.2464	0	52	27	8.17	36.5564
G. jazdzewski	no predator	0.2464	0	52	28	8.17	40.36726
G. jazdzewski	no predator	0.2464	0	52	29	8.14	35.5763
G. jazdzewski	no predator	0.2464	0	52	30	8.14	36.91802
G. jazdzewski	no predator	0.2062	0	53	1	8.44	34.39983
G. jazdzewski	no predator	0.2062	0	53	2	8.43	42.99962
G. jazdzewski	no predator	0.2062	0	53	3	8.42	41.24128
G. jazdzewski	no predator	0.2062	0	53	4	8.43	37.99968
G. jazdzewski	no predator	0.2062	0	53	5	8.42	42.08
G. jazdzewski	no predator	0.2062	0	53	6	8.42	39.15949
G. jazdzewski	no predator	0.2062	0	53	7	8.41	37.39939
G. jazdzewski	no predator	0.2062	0	53	8	8.41	34.92061
G. jazdzewski	no predator	0.2062	0	53	9	8.39	40.57852
G. jazdzewski	no predator	0.2062	0	53	10	8.4	33.03992
G. jazdzewski	no predator	0.2062	0	53	11	8.38	35.6024
G. jazdzewski	no predator	0.2062	0	53	12	8.39	36.93998
G. jazdzewski	no predator	0.2062	0	53	13	8.37	35.78097
G. jazdzewski	no predator	0.2062	0	53	14	8.36	33.7411
G. jazdzewski	no predator	0.2062	0	53	15	8.36	38.71902
G. jazdzewski	no predator	0.2062	0	53	16	8.34	37.36005
G. jazdzewski	no predator	0.2062	0	53	17	8.34	35.60007
G. jazdzewski	no predator	0.2062	0	53	18	8.34	34.81376
G. jazdzewski	no predator	0.2062	0	53	19	8.32	31.58509
G. jazdzewski	no predator	0.2062	0	53	20	8.32	36.41999
G. jazdzewski	no predator	0.2062	0	53	21	8.3	33.08292
G. jazdzewski	no predator	0.2062	0	53	22	8.29	35.28307
G. jazdzewski	no predator	0.2062	0	53	23	8.28	35.89394
G. jazdzewski	no predator	0.2062	0	53	24	8.3	31.51832

Gammarid species	treatment	weight [g]	pre-exposure time	trial	time [min.]	oxygen level [mg/l]	activity
G. jazdzewski	no predator	0.2062	0	53	25	8.27	34.70984
G. jazdzewski	no predator	0.2062	0	53	26	8.27	35.35659
G. jazdzewski	no predator	0.2062	0	53	27	8.25	34.72175
G. jazdzewski	no predator	0.2062	0	53	28	8.27	39.22368
G. jazdzewski	no predator	0.2062	0	53	29	8.24	34.78204
G. jazdzewski	no predator	0.2062	0	53	30	8.23	39.63617
G. jazdzewski	no predator	0.1936	0	54	1	8.41	20.3989
G. jazdzewski	no predator	0.1936	0	54	2	8.39	13.72047
G. jazdzewski	no predator	0.1936	0	54	3	8.39	20.17957
G. jazdzewski	no predator	0.1936	0	54	4	8.37	17.86072
G. jazdzewski	no predator	0.1936	0	54	5	8.38	17.06091
G. jazdzewski	no predator	0.1936	0	54	6	8.37	16.85954
G. jazdzewski	no predator	0.1936	0	54	7	8.36	16.85941
G. jazdzewski	no predator	0.1936	0	54	8	8.36	21.0025
G. jazdzewski	no predator	0.1936	0	54	9	8.33	20.05938
G. jazdzewski	no predator	0.1936	0	54	10	8.33	28.08147
G. jazdzewski	no predator	0.1936	0	54	11	8.32	22.80086
G. jazdzewski	no predator	0.1936	0	54	12	8.33	16.78012
G. jazdzewski	no predator	0.1936	0	54	13	8.3	18.41917
G. jazdzewski	no predator	0.1936	0	54	14	8.31	25.08094
G. jazdzewski	no predator	0.1936	0	54	15	8.32	20.9211
G. jazdzewski	no predator	0.1936	0	54	16	8.31	18.25784
G. jazdzewski	no predator	0.1936	0	54	17	8.31	26.66232
G. jazdzewski	no predator	0.1936	0	54	18	8.3	26.78004
G. jazdzewski	no predator	0.1936	0	54	19	8.28	26.14005
G. jazdzewski	no predator	0.1936	0	54	20	8.28	26.8255
G. jazdzewski	no predator	0.1936	0	54	21	8.26	22.83161
G. jazdzewski	no predator	0.1936	0	54	22	8.27	21.14742
G. jazdzewski	no predator	0.1936	0	54	23	8.25	25.53546
G. jazdzewski	no predator	0.1936	0	54	24	8.23	23.90162
G. jazdzewski	no predator	0.1936	0	54	25	8.22	26.30165
G. jazdzewski	no predator	0.1936	0	54	26	8.21	32.47649
G. jazdzewski	no predator	0.1936	0	54	27	8.19	25.86531
G. jazdzewski	no predator	0.1936	0	54	28	8.19	29.55076
G. jazdzewski	no predator	0.1936	0	54	29	8.19	27.2094
G. jazdzewski	no predator	0.1936	0	54	30	8.18	29.25216
G. jazdzewski	no predator	0.2481	0	55	1	8.35	35.83982
G. jazdzewski	no predator	0.2481	0	55	2	8.36	37.61998
G. jazdzewski	no predator	0.2481	0	55	3	8.36	44.04065
G. jazdzewski	no predator	0.2481	0	55	4	8.38	39.57923
G. jazdzewski	no predator	0.2481	0	55	5	8.37	39.9599
G. jazdzewski	no predator	0.2481	0	55	6	8.37	32.24053
G. jazdzewski	no predator	0.2481	0	55	7	8.36	37.63986
G. jazdzewski	no predator	0.2481	0	55	8	8.34	37.74052
G. jazdzewski	no predator	0.2481	0	55	9	8.32	37.57987
G. jazdzewski	no predator	0.2481	0	55	10	8.32	39.54298
G. jazdzewski	no predator	0.2481	0	55	11	8.32	32.53926
G. jazdzewski	no predator	0.2481	0	55	12	8.31	37.30172

Gammarid species	treatment	weight [g]	pre-exposure time	trial	time [min.]	oxygen level [mg/l]	activity
G. jazdzewski	no predator	0.2481	0	55	13	8.3	39.25907
G. jazdzewski	no predator	0.2481	0	55	14	8.28	36.27999
G. jazdzewski	no predator	0.2481	0	55	15	8.28	40.72209
G. jazdzewski	no predator	0.2481	0	55	16	8.28	40.03665
G. jazdzewski	no predator	0.2481	0	55	17	8.27	39.52352
G. jazdzewski	no predator	0.2481	0	55	18	8.26	40.6375
G. jazdzewski	no predator	0.2481	0	55	19	8.22	37.8252
G. jazdzewski	no predator	0.2481	0	55	20	8.23	42.95734
G. jazdzewski	no predator	0.2481	0	55	21	8.23	40.76146
G. jazdzewski	no predator	0.2481	0	55	22	8.2	41.43999
G. jazdzewski	no predator	0.2481	0	55	23	8.21	40.9815
G. jazdzewski	no predator	0.2481	0	55	24	8.21	45.35998
G. jazdzewski	no predator	0.2481	0	55	25	8.17	46.94156
G. jazdzewski	no predator	0.2481	0	55	26	8.15	42.36
G. jazdzewski	no predator	0.2481	0	55	27	8.14	42.02001
G. jazdzewski	no predator	0.2481	0	55	28	8.15	41.36368
G. jazdzewski	no predator	0.2481	0	55	29	8.13	41.86199
G. jazdzewski	no predator	0.2481	0	55	30	8.13	43.57813
G. jazdzewski	no predator	0.1654	0	56	1	8.51	10.78001
G. jazdzewski	no predator	0.1654	0	56	2	8.51	14.20058
G. jazdzewski	no predator	0.1654	0	56	3	8.5	13.35902
G. jazdzewski	no predator	0.1654	0	56	4	8.5	15.32068
G. jazdzewski	no predator	0.1654	0	56	5	8.48	10.87906
G. jazdzewski	no predator	0.1654	0	56	6	8.49	12.74041
G. jazdzewski	no predator	0.1654	0	56	7	8.48	13.28173
G. jazdzewski	no predator	0.1654	0	56	8	8.48	12.75748
G. jazdzewski	no predator	0.1654	0	56	9	8.47	17.28127
G. jazdzewski	no predator	0.1654	0	56	10	8.47	22.08068
G. jazdzewski	no predator	0.1654	0	56	11	8.46	21.82165
G. jazdzewski	no predator	0.1654	0	56	12	8.46	30.91908
G. jazdzewski	no predator	0.1654	0	56	13	8.45	31.2037
G. jazdzewski	no predator	0.1654	0	56	14	8.45	29.84115
G. jazdzewski	no predator	0.1654	0	56	15	8.45	36.3579
G. jazdzewski	no predator	0.1654	0	56	16	8.44	31.04342
G. jazdzewski	no predator	0.1654	0	56	17	8.43	30.76017
G. jazdzewski	no predator	0.1654	0	56	18	8.44	34.50007
G. jazdzewski	no predator	0.1654	0	56	19	8.43	33.04008
G. jazdzewski	no predator	0.1654	0	56	20	8.42	31.07735
G. jazdzewski	no predator	0.1654	0	56	21	8.41	31.36717
G. jazdzewski	no predator	0.1654	0	56	22	8.38	32.93428
G. jazdzewski	no predator	0.1654	0	56	23	8.41	35.49689
G. jazdzewski	no predator	0.1654	0	56	24	8.38	27.19997
G. jazdzewski	no predator	0.1654	0	56	25	8.36	27.35829
G. jazdzewski	no predator	0.1654	0	56	26	8.35	33.35983
G. jazdzewski	no predator	0.1654	0	56	27	8.34	27.6399
G. jazdzewski	no predator	0.1654	0	56	28	8.34	29.52362
G. jazdzewski	no predator	0.1654	0	56	29	8.34	30.61615
G. jazdzewski	no predator	0.1654	0	56	30	8.34	31.02184

Gammarid species	treatment	weight [g]	pre-exposure time	trial	time [min.]	oxygen level [mg/l]	activity
G. jazdzewski	no predator	0.2238	0	57	1	8.41	21.36003
G. jazdzewski	no predator	0.2238	0	57	2	8.38	19.45976
G. jazdzewski	no predator	0.2238	0	57	3	8.36	24.50029
G. jazdzewski	no predator	0.2238	0	57	4	8.35	25.56163
G. jazdzewski	no predator	0.2238	0	57	5	8.37	23.47819
G. jazdzewski	no predator	0.2238	0	57	6	8.36	20.321
G. jazdzewski	no predator	0.2238	0	57	7	8.29	21.77882
G. jazdzewski	no predator	0.2238	0	57	8	8.33	26.44382
G. jazdzewski	no predator	0.2238	0	57	9	8.35	28.67725
G. jazdzewski	no predator	0.2238	0	57	10	8.32	24.46306
G. jazdzewski	no predator	0.2238	0	57	11	8.31	27.21763
G. jazdzewski	no predator	0.2238	0	57	12	8.29	29.88078
G. jazdzewski	no predator	0.2238	0	57	13	8.29	26.55709
G. jazdzewski	no predator	0.2238	0	57	14	8.28	30.02485
G. jazdzewski	no predator	0.2238	0	57	15	8.27	25.43688
G. jazdzewski	no predator	0.2238	0	57	16	8.25	23.65995
G. jazdzewski	no predator	0.2238	0	57	17	8.24	23.12116
G. jazdzewski	no predator	0.2238	0	57	18	8.24	22.94499
G. jazdzewski	no predator	0.2238	0	57	19	8.23	19.44004
G. jazdzewski	no predator	0.2238	0	57	20	8.22	18.28005
G. jazdzewski	no predator	0.2238	0	57	21	8.21	27.34283
G. jazdzewski	no predator	0.2238	0	57	22	8.19	20.77565
G. jazdzewski	no predator	0.2238	0	57	23	8.18	23.04152
G. jazdzewski	no predator	0.2238	0	57	24	8.17	23.81677
G. jazdzewski	no predator	0.2238	0	57	25	8.14	28.02321
G. jazdzewski	no predator	0.2238	0	57	26	8.12	28.53995
G. jazdzewski	no predator	0.2238	0	57	27	8.11	22.67822
G. jazdzewski	no predator	0.2238	0	57	28	8.11	25.38916
G. jazdzewski	no predator	0.2238	0	57	29	8.04	29.17815
G. jazdzewski	no predator	0.2238	0	57	30	8.03	27.83408
G. jazdzewski	no predator	0.1823	0	58	1	8.4	40.75977
G. jazdzewski	no predator	0.1823	0	58	2	8.37	38.31998
G. jazdzewski	no predator	0.1823	0	58	3	8.36	35.57966
G. jazdzewski	no predator	0.1823	0	58	4	8.36	41.14032
G. jazdzewski	no predator	0.1823	0	58	5	8.36	39.40093
G. jazdzewski	no predator	0.1823	0	58	6	8.34	41.26001
G. jazdzewski	no predator	0.1823	0	58	7	8.33	41.88059
G. jazdzewski	no predator	0.1823	0	58	8	8.32	39.02069
G. jazdzewski	no predator	0.1823	0	58	9	8.32	39.99937
G. jazdzewski	no predator	0.1823	0	58	10	8.3	38.0023
G. jazdzewski	no predator	0.1823	0	58	11	8.3	44.65841
G. jazdzewski	no predator	0.1823	0	58	12	8.28	44.59999
G. jazdzewski	no predator	0.1823	0	58	13	8.25	43.48088
G. jazdzewski	no predator	0.1823	0	58	14	8.25	40.24098
G. jazdzewski	no predator	0.1823	0	58	15	8.23	37.97998
G. jazdzewski	no predator	0.1823	0	58	16	8.21	40.40109
G. jazdzewski	no predator	0.1823	0	58	17	8.21	33.53883
G. jazdzewski	no predator	0.1823	0	58	18	8.2	40.06361

Gammarid species	treatment	weight [g]	pre-exposure time	trial	time [min.]	oxygen level [mg/l]	activity
G. jazdzewski	no predator	0.1823	0	58	19	8.2	36.52268
G. jazdzewski	no predator	0.1823	0	58	20	8.21	44.0228
G. jazdzewski	no predator	0.1823	0	58	21	8.18	40.63587
G. jazdzewski	no predator	0.1823	0	58	22	8.19	36.98154
G. jazdzewski	no predator	0.1823	0	58	23	8.17	37.14012
G. jazdzewski	no predator	0.1823	0	58	24	8.16	38.76003
G. jazdzewski	no predator	0.1823	0	58	25	8.17	42.63664
G. jazdzewski	no predator	0.1823	0	58	26	8.17	40.82337
G. jazdzewski	no predator	0.1823	0	58	27	8.13	38.47643
G. jazdzewski	no predator	0.1823	0	58	28	8.14	40.04548
G. jazdzewski	no predator	0.1823	0	58	29	8.14	41.29623
G. jazdzewski	no predator	0.1823	0	58	30	8.14	35.79998
G. jazdzewski	no predator	0.2581	0	59	1	8.4	39.40052
G. jazdzewski	no predator	0.2581	0	59	2	8.38	41.79916
G. jazdzewski	no predator	0.2581	0	59	3	8.36	38.06028
G. jazdzewski	no predator	0.2581	0	59	4	8.34	31.74005
G. jazdzewski	no predator	0.2581	0	59	5	8.33	42.53899
G. jazdzewski	no predator	0.2581	0	59	6	8.33	33.82152
G. jazdzewski	no predator	0.2581	0	59	7	8.3	36.63947
G. jazdzewski	no predator	0.2581	0	59	8	8.29	37.96185
G. jazdzewski	no predator	0.2581	0	59	9	8.27	34.4993
G. jazdzewski	no predator	0.2581	0	59	10	8.27	35.57916
G. jazdzewski	no predator	0.2581	0	59	11	8.25	34.60242
G. jazdzewski	no predator	0.2581	0	59	12	8.23	38.85907
G. jazdzewski	no predator	0.2581	0	59	13	8.23	37.20088
G. jazdzewski	no predator	0.2581	0	59	14	8.22	37.33693
G. jazdzewski	no predator	0.2581	0	59	15	8.2	40.32296
G. jazdzewski	no predator	0.2581	0	59	16	8.21	41.21874
G. jazdzewski	no predator	0.2581	0	59	17	8.21	42.3434
G. jazdzewski	no predator	0.2581	0	59	18	8.19	41.44115
G. jazdzewski	no predator	0.2581	0	59	19	8.19	35.97868
G. jazdzewski	no predator	0.2581	0	59	20	8.18	34.8241
G. jazdzewski	no predator	0.2581	0	59	21	8.16	37.12142
G. jazdzewski	no predator	0.2581	0	59	22	8.16	34.09708
G. jazdzewski	no predator	0.2581	0	59	23	8.15	36.09837
G. jazdzewski	no predator	0.2581	0	59	24	8.14	36.68149
G. jazdzewski	no predator	0.2581	0	59	25	8.12	32.98502
G. jazdzewski	no predator	0.2581	0	59	26	8.12	28.35498
G. jazdzewski	no predator	0.2581	0	59	27	8.1	30.62
G. jazdzewski	no predator	0.2581	0	59	28	8.1	34.2218
G. jazdzewski	no predator	0.2581	0	59	29	8.09	32.11999
G. jazdzewski	no predator	0.2581	0	59	30	8.07	31.16196
G. jazdzewski	no predator	0.2165	0	60	1	8.39	36.6403
G. jazdzewski	no predator	0.2165	0	60	2	8.38	38.28058
G. jazdzewski	no predator	0.2165	0	60	3	8.36	35.20048
G. jazdzewski	no predator	0.2165	0	60	4	8.36	36.88018
G. jazdzewski	no predator	0.2165	0	60	5	8.37	41.11917
G. jazdzewski	no predator	0.2165	0	60	6	8.35	41.09893

Gammarid species	treatment	weight [g]	pre-exposure time	trial	time [min.]	oxygen level [mg/l]	activity
G. jazdzewski	no predator	0.2165	0	60	7	8.34	38.59993
G. jazdzewski	no predator	0.2165	0	60	8	8.33	35.70188
G. jazdzewski	no predator	0.2165	0	60	9	8.32	34.37791
G. jazdzewski	no predator	0.2165	0	60	10	8.34	36.63986
G. jazdzewski	no predator	0.2165	0	60	11	8.32	36.7424
G. jazdzewski	no predator	0.2165	0	60	12	8.3	39.72177
G. jazdzewski	no predator	0.2165	0	60	13	8.27	35.91916
G. jazdzewski	no predator	0.2165	0	60	14	8.25	36.43901
G. jazdzewski	no predator	0.2165	0	60	15	8.22	39.37997
G. jazdzewski	no predator	0.2165	0	60	16	8.2	37.17772
G. jazdzewski	no predator	0.2165	0	60	17	8.19	39.06337
G. jazdzewski	no predator	0.2165	0	60	18	8.18	36.8463
G. jazdzewski	no predator	0.2165	0	60	19	8.15	39.69887
G. jazdzewski	no predator	0.2165	0	60	20	8.15	39.79602
G. jazdzewski	no predator	0.2165	0	60	21	8.14	39.71995
G. jazdzewski	no predator	0.2165	0	60	22	8.14	38.99847
G. jazdzewski	no predator	0.2165	0	60	23	8.12	31.34466
G. jazdzewski	no predator	0.2165	0	60	24	8.11	35.47527
G. jazdzewski	no predator	0.2165	0	60	25	8.07	34.88164
G. jazdzewski	no predator	0.2165	0	60	26	8.05	35.38342
G. jazdzewski	no predator	0.2165	0	60	27	8.04	36.85998
G. jazdzewski	no predator	0.2165	0	60	28	8.04	30.91448
G. jazdzewski	no predator	0.2165	0	60	29	8.03	36.36749
G. jazdzewski	no predator	0.2165	0	60	30	8.01	38.68195
G. jazdzewski	no predator	0.2028	0	61	1	8.33	19.9804
G. jazdzewski	no predator	0.2028	0	61	2	8.3	29.98049
G. jazdzewski	no predator	0.2028	0	61	3	8.28	20.62034
G. jazdzewski	no predator	0.2028	0	61	4	8.28	21.6792
G. jazdzewski	no predator	0.2028	0	61	5	8.26	21.24135
G. jazdzewski	no predator	0.2028	0	61	6	8.23	27.95947
G. jazdzewski	no predator	0.2028	0	61	7	8.22	21.24003
G. jazdzewski	no predator	0.2028	0	61	8	8.21	17.74009
G. jazdzewski	no predator	0.2028	0	61	9	8.22	19.33933
G. jazdzewski	no predator	0.2028	0	61	10	8.21	19.40071
G. jazdzewski	no predator	0.2028	0	61	11	8.23	24.59992
G. jazdzewski	no predator	0.2028	0	61	12	8.22	20.91996
G. jazdzewski	no predator	0.2028	0	61	13	8.21	19.20095
G. jazdzewski	no predator	0.2028	0	61	14	8.17	22.95901
G. jazdzewski	no predator	0.2028	0	61	15	8.16	16.84422
G. jazdzewski	no predator	0.2028	0	61	16	8.15	15.65895
G. jazdzewski	no predator	0.2028	0	61	17	8.16	21.52355
G. jazdzewski	no predator	0.2028	0	61	18	8.16	22.27384
G. jazdzewski	no predator	0.2028	0	61	19	8.17	22.96506
G. jazdzewski	no predator	0.2028	0	61	20	8.18	23.18001
G. jazdzewski	no predator	0.2028	0	61	21	8.14	19.7986
G. jazdzewski	no predator	0.2028	0	61	22	8.13	17.57851
G. jazdzewski	no predator	0.2028	0	61	23	8.11	23.743
G. jazdzewski	no predator	0.2028	0	61	24	8.11	19.21843

Gammarid species	treatment	weight [g]	pre-exposure time	trial	time [min.]	oxygen level [mg/l]	activity
G. jazdzewski	no predator	0.2028	0	61	25	8.09	19.78335
G. jazdzewski	no predator	0.2028	0	61	26	8.07	19.3601
G. jazdzewski	no predator	0.2028	0	61	27	8.05	22.07649
G. jazdzewski	no predator	0.2028	0	61	28	8.04	23.02553
G. jazdzewski	no predator	0.2028	0	61	29	8.04	19.94863
G. jazdzewski	no predator	0.2028	0	61	30	8.05	19.58771
G. jazdzewski	no predator	0.2156	0	62	1	8.43	14.29956
G. jazdzewski	no predator	0.2156	0	62	2	8.39	9.540278
G. jazdzewski	no predator	0.2156	0	62	3	8.4	8.379309
G. jazdzewski	no predator	0.2156	0	62	4	8.36	12.2003
G. jazdzewski	no predator	0.2156	0	62	5	8.37	10.56139
G. jazdzewski	no predator	0.2156	0	62	6	8.36	13.61903
G. jazdzewski	no predator	0.2156	0	62	7	8.39	17.88114
G. jazdzewski	no predator	0.2156	0	62	8	8.37	15.7587
G. jazdzewski	no predator	0.2156	0	62	9	8.38	18.92269
G. jazdzewski	no predator	0.2156	0	62	10	8.36	15.62159
G. jazdzewski	no predator	0.2156	0	62	11	8.31	12.81682
G. jazdzewski	no predator	0.2156	0	62	12	8.31	15.39991
G. jazdzewski	no predator	0.2156	0	62	13	8.29	18.09988
G. jazdzewski	no predator	0.2156	0	62	14	8.3	16.7799
G. jazdzewski	no predator	0.2156	0	62	15	8.27	13.70316
G. jazdzewski	no predator	0.2156	0	62	16	8.28	16.13667
G. jazdzewski	no predator	0.2156	0	62	17	8.27	17.92349
G. jazdzewski	no predator	0.2156	0	62	18	8.27	19.76005
G. jazdzewski	no predator	0.2156	0	62	19	8.25	22.2187
G. jazdzewski	no predator	0.2156	0	62	20	8.25	16.34278
G. jazdzewski	no predator	0.2156	0	62	21	8.23	21.32002
G. jazdzewski	no predator	0.2156	0	62	22	8.25	19.18299
G. jazdzewski	no predator	0.2156	0	62	23	8.25	12.99705
G. jazdzewski	no predator	0.2156	0	62	24	8.24	14.58165
G. jazdzewski	no predator	0.2156	0	62	25	8.25	11.45838
G. jazdzewski	no predator	0.2156	0	62	26	8.23	13.83998
G. jazdzewski	no predator	0.2156	0	62	27	8.21	13.61999
G. jazdzewski	no predator	0.2156	0	62	28	8.17	12.74185
G. jazdzewski	no predator	0.2156	0	62	29	8.17	17.97994
G. jazdzewski	no predator	0.2156	0	62	30	8.17	14.0279
G. jazdzewski	no predator	0.1896	0	63	1	8.32	30.04047
G. jazdzewski	no predator	0.1896	0	63	2	8.33	29.80037
G. jazdzewski	no predator	0.1896	0	63	3	8.33	28.15906
G. jazdzewski	no predator	0.1896	0	63	4	8.32	31.32119
G. jazdzewski	no predator	0.1896	0	63	5	8.32	24.36018
G. jazdzewski	no predator	0.1896	0	63	6	8.31	28.01797
G. jazdzewski	no predator	0.1896	0	63	7	8.3	27.09991
G. jazdzewski	no predator	0.1896	0	63	8	8.3	29.68055
G. jazdzewski	no predator	0.1896	0	63	9	8.29	25.29927
G. jazdzewski	no predator	0.1896	0	63	10	8.27	27.04149
G. jazdzewski	no predator	0.1896	0	63	11	8.28	27.58002
G. jazdzewski	no predator	0.1896	0	63	12	8.26	23.32006

Gammarid species	treatment	weight [g]	pre-exposure time	trial	time [min.]	oxygen level [mg/l]	activity
G. jazdzewski	no predator	0.1896	0	63	13	8.26	25.17716
G. jazdzewski	no predator	0.1896	0	63	14	8.24	28.34085
G. jazdzewski	no predator	0.1896	0	63	15	8.24	20.96322
G. jazdzewski	no predator	0.1896	0	63	16	8.23	24.1567
G. jazdzewski	no predator	0.1896	0	63	17	8.22	20.06591
G. jazdzewski	no predator	0.1896	0	63	18	8.23	25.57513
G. jazdzewski	no predator	0.1896	0	63	19	8.21	26.69985
G. jazdzewski	no predator	0.1896	0	63	20	8.2	26.70399
G. jazdzewski	no predator	0.1896	0	63	21	8.2	26.55715
G. jazdzewski	no predator	0.1896	0	63	22	8.19	22.28296
G. jazdzewski	no predator	0.1896	0	63	23	8.17	27.33533
G. jazdzewski	no predator	0.1896	0	63	24	8.17	28.82138
G. jazdzewski	no predator	0.1896	0	63	25	8.16	28.24488
G. jazdzewski	no predator	0.1896	0	63	26	8.15	26.33829
G. jazdzewski	no predator	0.1896	0	63	27	8.15	31.62165
G. jazdzewski	no predator	0.1896	0	63	28	8.14	31.64353
G. jazdzewski	no predator	0.1896	0	63	29	8.13	27.80762
G. jazdzewski	no predator	0.1896	0	63	30	8.12	28.41811
G. jazdzewski	no predator	0.2062	0	64	1	8.37	37.61874
G. jazdzewski	no predator	0.2062	0	64	2	8.36	35.90073
G. jazdzewski	no predator	0.2062	0	64	3	8.37	31.25933
G. jazdzewski	no predator	0.2062	0	64	4	8.39	29.81994
G. jazdzewski	no predator	0.2062	0	64	5	8.37	34.66079
G. jazdzewski	no predator	0.2062	0	64	6	8.36	31.43891
G. jazdzewski	no predator	0.2062	0	64	7	8.36	33.12045
G. jazdzewski	no predator	0.2062	0	64	8	8.34	32.68057
G. jazdzewski	no predator	0.2062	0	64	9	8.33	29.69928
G. jazdzewski	no predator	0.2062	0	64	10	8.33	26.45997
G. jazdzewski	no predator	0.2062	0	64	11	8.32	29.53995
G. jazdzewski	no predator	0.2062	0	64	12	8.33	26.40177
G. jazdzewski	no predator	0.2062	0	64	13	8.32	27.77911
G. jazdzewski	no predator	0.2062	0	64	14	8.33	29.71798
G. jazdzewski	no predator	0.2062	0	64	15	8.35	26.55991
G. jazdzewski	no predator	0.2062	0	64	16	8.34	30.13645
G. jazdzewski	no predator	0.2062	0	64	17	8.34	23.62336
G. jazdzewski	no predator	0.2062	0	64	18	8.32	25.97993
G. jazdzewski	no predator	0.2062	0	64	19	8.32	22.66391
G. jazdzewski	no predator	0.2062	0	64	20	8.32	18.67742
G. jazdzewski	no predator	0.2062	0	64	21	8.29	25.16
G. jazdzewski	no predator	0.2062	0	64	22	8.28	20.82441
G. jazdzewski	no predator	0.2062	0	64	23	8.27	23.89849
G. jazdzewski	no predator	0.2062	0	64	24	8.28	21.52
G. jazdzewski	no predator	0.2062	0	64	25	8.25	25.63663
G. jazdzewski	no predator	0.2062	0	64	26	8.25	26.58682
G. jazdzewski	no predator	0.2062	0	64	27	8.26	24.29832
G. jazdzewski	no predator	0.2062	0	64	28	8.26	25.47818
G. jazdzewski	no predator	0.2062	0	64	29	8.27	27.81798
G. jazdzewski	no predator	0.2062	0	64	30	8.24	24.50779

Gammarid species	treatment	weight [g]	pre-exposure time	trial	time [min.]	oxygen level [mg/l]	activity
G. jazdzewski	perch	0.3554	0	65	1	8.84	4.719777
G. jazdzewski	perch	0.3554	0	65	2	8.85	5.779946
G. jazdzewski	perch	0.3554	0	65	3	8.83	2.340336
G. jazdzewski	perch	0.3554	0	65	4	8.83	6.139997
G. jazdzewski	perch	0.3554	0	65	5	8.79	6.239521
G. jazdzewski	perch	0.3554	0	65	6	8.78	6.060477
G. jazdzewski	perch	0.3554	0	65	7	8.78	4.959411
G. jazdzewski	perch	0.3554	0	65	8	8.78	7.581251
G. jazdzewski	perch	0.3554	0	65	9	8.75	8.518592
G. jazdzewski	perch	0.3554	0	65	10	8.75	7.999922
G. jazdzewski	perch	0.3554	0	65	11	8.74	9.342422
G. jazdzewski	perch	0.3554	0	65	12	8.73	10.75829
G. jazdzewski	perch	0.3554	0	65	13	8.72	10.49995
G. jazdzewski	perch	0.3554	0	65	14	8.71	10.342
G. jazdzewski	perch	0.3554	0	65	15	8.69	10.37787
G. jazdzewski	perch	0.3554	0	65	16	8.68	9.543329
G. jazdzewski	perch	0.3554	0	65	17	8.68	11.32242
G. jazdzewski	perch	0.3554	0	65	18	8.65	17.03745
G. jazdzewski	perch	0.3554	0	65	19	8.65	10.08122
G. jazdzewski	perch	0.3554	0	65	20	8.65	14.82266
G. jazdzewski	perch	0.3554	0	65	21	8.62	11.34294
G. jazdzewski	perch	0.3554	0	65	22	8.62	11.37563
G. jazdzewski	perch	0.3554	0	65	23	8.59	12.48151
G. jazdzewski	perch	0.3554	0	65	24	8.56	9.736789
G. jazdzewski	perch	0.3554	0	65	25	8.58	9.921586
G. jazdzewski	perch	0.3554	0	65	26	8.57	12.19993
G. jazdzewski	perch	0.3554	0	65	27	8.54	15.7781
G. jazdzewski	perch	0.3554	0	65	28	8.54	16.50355
G. jazdzewski	perch	0.3554	0	65	29	8.54	12.14001
G. jazdzewski	perch	0.3554	0	65	30	8.51	14.38592
G. jazdzewski	perch	0.3081	0	66	1	8.87	17.30031
G. jazdzewski	perch	0.3081	0	66	2	8.83	21.61991
G. jazdzewski	perch	0.3081	0	66	3	8.81	18.04137
G. jazdzewski	perch	0.3081	0	66	4	8.81	22.50008
G. jazdzewski	perch	0.3081	0	66	5	8.79	23.82053
G. jazdzewski	perch	0.3081	0	66	6	8.77	24.78003
G. jazdzewski	perch	0.3081	0	66	7	8.77	22.73828
G. jazdzewski	perch	0.3081	0	66	8	8.74	21.70387
G. jazdzewski	perch	0.3081	0	66	9	8.75	26.27788
G. jazdzewski	perch	0.3081	0	66	10	8.73	27.14298
G. jazdzewski	perch	0.3081	0	66	11	8.72	26.71758
G. jazdzewski	perch	0.3081	0	66	12	8.72	26.14441
G. jazdzewski	perch	0.3081	0	66	13	8.72	25.79445
G. jazdzewski	perch	0.3081	0	66	14	8.69	27.68392
G. jazdzewski	perch	0.3081	0	66	15	8.68	23.63892
G. jazdzewski	perch	0.3081	0	66	16	8.67	24.70449
G. jazdzewski	perch	0.3081	0	66	17	8.66	24.73771
G. jazdzewski	perch	0.3081	0	66	18	8.64	27.31745

Gammarid species	treatment	weight [g]	pre-exposure time	trial	time [min.]	oxygen level [mg/l]	activity
G. jazdzewski	perch	0.3081	0	66	19	8.65	24.3825
G. jazdzewski	perch	0.3081	0	66	20	8.65	27.10411
G. jazdzewski	perch	0.3081	0	66	21	8.61	26.99727
G. jazdzewski	perch	0.3081	0	66	22	8.61	26.88453
G. jazdzewski	perch	0.3081	0	66	23	8.58	28.52013
G. jazdzewski	perch	0.3081	0	66	24	8.58	26.15369
G. jazdzewski	perch	0.3081	0	66	25	8.57	24.95993
G. jazdzewski	perch	0.3081	0	66	26	8.57	29.0199
G. jazdzewski	perch	0.3081	0	66	27	8.56	26.56525
G. jazdzewski	perch	0.3081	0	66	28	8.56	24.72006
G. jazdzewski	perch	0.3081	0	66	29	8.54	22.45241
G. jazdzewski	perch	0.3081	0	66	30	8.53	23.38971
G. jazdzewski	perch	0.3686	0	67	1	8.88	22.45971
G. jazdzewski	perch	0.3686	0	67	2	8.87	21.66079
G. jazdzewski	perch	0.3686	0	67	3	8.84	26.61998
G. jazdzewski	perch	0.3686	0	67	4	8.81	25.63997
G. jazdzewski	perch	0.3686	0	67	5	8.78	27.21803
G. jazdzewski	perch	0.3686	0	67	6	8.77	30.46238
G. jazdzewski	perch	0.3686	0	67	7	8.74	26.05939
G. jazdzewski	perch	0.3686	0	67	8	8.71	28.13986
G. jazdzewski	perch	0.3686	0	67	9	8.72	29.18055
G. jazdzewski	perch	0.3686	0	67	10	8.7	29.36066
G. jazdzewski	perch	0.3686	0	67	11	8.69	36.40155
G. jazdzewski	perch	0.3686	0	67	12	8.67	28.08277
G. jazdzewski	perch	0.3686	0	67	13	8.66	29.83636
G. jazdzewski	perch	0.3686	0	67	14	8.65	27.22099
G. jazdzewski	perch	0.3686	0	67	15	8.62	29.52
G. jazdzewski	perch	0.3686	0	67	16	8.61	29.66
G. jazdzewski	perch	0.3686	0	67	17	8.6	35.12233
G. jazdzewski	perch	0.3686	0	67	18	8.59	35.83628
G. jazdzewski	perch	0.3686	0	67	19	8.57	25.23867
G. jazdzewski	perch	0.3686	0	67	20	8.57	31.40677
G. jazdzewski	perch	0.3686	0	67	21	8.55	31.73727
G. jazdzewski	perch	0.3686	0	67	22	8.55	32.04298
G. jazdzewski	perch	0.3686	0	67	23	8.52	41.0384
G. jazdzewski	perch	0.3686	0	67	24	8.5	34.39994
G. jazdzewski	perch	0.3686	0	67	25	8.47	34.86331
G. jazdzewski	perch	0.3686	0	67	26	8.47	36.22173
G. jazdzewski	perch	0.3686	0	67	27	8.45	32.02191
G. jazdzewski	perch	0.3686	0	67	28	8.44	37.31638
G. jazdzewski	perch	0.3686	0	67	29	8.43	35.80372
G. jazdzewski	perch	0.3686	0	67	30	8.41	32.75607
G. jazdzewski	perch	0.2684	0	68	1	8.91	29.0194
G. jazdzewski	perch	0.2684	0	68	2	8.91	23.6006
G. jazdzewski	perch	0.2684	0	68	3	8.88	28.72079
G. jazdzewski	perch	0.2684	0	68	4	8.86	29.83928
G. jazdzewski	perch	0.2684	0	68	5	8.85	31.97901
G. jazdzewski	perch	0.2684	0	68	6	8.85	30.75879

Gammarid species	treatment	weight [g]	pre-exposure time	trial	time [min.]	oxygen level [mg/l]	activity
G. jazdzewski	perch	0.2684	0	68	7	8.83	34.08331
G. jazdzewski	perch	0.2684	0	68	8	8.81	33.21744
G. jazdzewski	perch	0.2684	0	68	9	8.79	32.83984
G. jazdzewski	perch	0.2684	0	68	10	8.8	33.19985
G. jazdzewski	perch	0.2684	0	68	11	8.77	37.98226
G. jazdzewski	perch	0.2684	0	68	12	8.78	33.3453
G. jazdzewski	perch	0.2684	0	68	13	8.75	32.91734
G. jazdzewski	perch	0.2684	0	68	14	8.75	36.00204
G. jazdzewski	perch	0.2684	0	68	15	8.73	35.10222
G. jazdzewski	perch	0.2684	0	68	16	8.72	32.18016
G. jazdzewski	perch	0.2684	0	68	17	8.73	35.92001
G. jazdzewski	perch	0.2684	0	68	18	8.71	36.36623
G. jazdzewski	perch	0.2684	0	68	19	8.7	36.97628
G. jazdzewski	perch	0.2684	0	68	20	8.68	35.67729
G. jazdzewski	perch	0.2684	0	68	21	8.67	37.43987
G. jazdzewski	perch	0.2684	0	68	22	8.66	35.24137
G. jazdzewski	perch	0.2684	0	68	23	8.64	33.7599
G. jazdzewski	perch	0.2684	0	68	24	8.64	37.63987
G. jazdzewski	perch	0.2684	0	68	25	8.63	39.06986
G. jazdzewski	perch	0.2684	0	68	26	8.63	40.60007
G. jazdzewski	perch	0.2684	0	68	27	8.61	37.25471
G. jazdzewski	perch	0.2684	0	68	28	8.6	37.04365
G. jazdzewski	perch	0.2684	0	68	29	8.59	33.58391
G. jazdzewski	perch	0.2684	0	68	30	8.57	33.71822
G. jazdzewski	perch	0.3813	0	69	1	8.91	21.10045
G. jazdzewski	perch	0.3813	0	69	2	8.87	21.87977
G. jazdzewski	perch	0.3813	0	69	3	8.86	19.23969
G. jazdzewski	perch	0.3813	0	69	4	8.83	19.80125
G. jazdzewski	perch	0.3813	0	69	5	8.82	21.65857
G. jazdzewski	perch	0.3813	0	69	6	8.8	24.4809
G. jazdzewski	perch	0.3813	0	69	7	8.78	22.51993
G. jazdzewski	perch	0.3813	0	69	8	8.78	27.2599
G. jazdzewski	perch	0.3813	0	69	9	8.75	28.14273
G. jazdzewski	perch	0.3813	0	69	10	8.75	27.09774
G. jazdzewski	perch	0.3813	0	69	11	8.73	31.32316
G. jazdzewski	perch	0.3813	0	69	12	8.71	31.85823
G. jazdzewski	perch	0.3813	0	69	13	8.67	31.27993
G. jazdzewski	perch	0.3813	0	69	14	8.67	33.06196
G. jazdzewski	perch	0.3813	0	69	15	8.67	35.68109
G. jazdzewski	perch	0.3813	0	69	16	8.64	29.26014
G. jazdzewski	perch	0.3813	0	69	17	8.64	39.43996
G. jazdzewski	perch	0.3813	0	69	18	8.64	35.39748
G. jazdzewski	perch	0.3813	0	69	19	8.61	27.8839
G. jazdzewski	perch	0.3813	0	69	20	8.63	28.54015
G. jazdzewski	perch	0.3813	0	69	21	8.61	29.79723
G. jazdzewski	perch	0.3813	0	69	22	8.6	29.04289
G. jazdzewski	perch	0.3813	0	69	23	8.59	25.80153
G. jazdzewski	perch	0.3813	0	69	24	8.59	25.71842

Gammarid species	treatment	weight [g]	pre-exposure time	trial	time [min.]	oxygen level [mg/l]	activity
G. jazdzewski	perch	0.3813	0	69	25	8.58	26.0633
G. jazdzewski	perch	0.3813	0	69	26	8.56	29.32691
G. jazdzewski	perch	0.3813	0	69	27	8.54	29.63652
G. jazdzewski	perch	0.3813	0	69	28	8.55	27.21631
G. jazdzewski	perch	0.3813	0	69	29	8.53	25.77796
G. jazdzewski	perch	0.3813	0	69	30	8.5	29.78175
G. jazdzewski	perch	0.3294	0	70	1	8.82	29.02079
G. jazdzewski	perch	0.3294	0	70	2	8.84	36.2
G. jazdzewski	perch	0.3294	0	70	3	8.81	33.69967
G. jazdzewski	perch	0.3294	0	70	4	8.8	37.36076
G. jazdzewski	perch	0.3294	0	70	5	8.79	32.13817
G. jazdzewski	perch	0.3294	0	70	6	8.77	37.34196
G. jazdzewski	perch	0.3294	0	70	7	8.77	36.37946
G. jazdzewski	perch	0.3294	0	70	8	8.75	38.09998
G. jazdzewski	perch	0.3294	0	70	9	8.76	39.60139
G. jazdzewski	perch	0.3294	0	70	10	8.73	39.6193
G. jazdzewski	perch	0.3294	0	70	11	8.73	39.51834
G. jazdzewski	perch	0.3294	0	70	12	8.73	38.02442
G. jazdzewski	perch	0.3294	0	70	13	8.7	43.35536
G. jazdzewski	perch	0.3294	0	70	14	8.69	42.16191
G. jazdzewski	perch	0.3294	0	70	15	8.68	41.29246
G. jazdzewski	perch	0.3294	0	70	16	8.66	40.38531
G. jazdzewski	perch	0.3294	0	70	17	8.65	37.39874
G. jazdzewski	perch	0.3294	0	70	18	8.64	37.7386
G. jazdzewski	perch	0.3294	0	70	19	8.63	36.68378
G. jazdzewski	perch	0.3294	0	70	20	8.6	34.30272
G. jazdzewski	perch	0.3294	0	70	21	8.6	30.55869
G. jazdzewski	perch	0.3294	0	70	22	8.58	37.78147
G. jazdzewski	perch	0.3294	0	70	23	8.58	40.83059
G. jazdzewski	perch	0.3294	0	70	24	8.57	41.24769
G. jazdzewski	perch	0.3294	0	70	25	8.57	41.10991
G. jazdzewski	perch	0.3294	0	70	26	8.55	39.48965
G. jazdzewski	perch	0.3294	0	70	27	8.54	38.99051
G. jazdzewski	perch	0.3294	0	70	28	8.52	36.87082
G. jazdzewski	perch	0.3294	0	70	29	8.52	41.23322
G. jazdzewski	perch	0.3294	0	70	30	8.51	44.65025
G. jazdzewski	perch	0.3442	0	71	1	8.82	23.81988
G. jazdzewski	perch	0.3442	0	71	2	8.83	29.50102
G. jazdzewski	perch	0.3442	0	71	3	8.81	24.4401
G. jazdzewski	perch	0.3442	0	71	4	8.79	26.78008
G. jazdzewski	perch	0.3442	0	71	5	8.78	31.24053
G. jazdzewski	perch	0.3442	0	71	6	8.76	26.118
G. jazdzewski	perch	0.3442	0	71	7	8.74	26.84111
G. jazdzewski	perch	0.3442	0	71	8	8.76	28.46002
G. jazdzewski	perch	0.3442	0	71	9	8.74	28.98001
G. jazdzewski	perch	0.3442	0	71	10	8.73	23.75999
G. jazdzewski	perch	0.3442	0	71	11	8.71	33.5199
G. jazdzewski	perch	0.3442	0	71	12	8.71	26.9026

Gammarid species	treatment	weight [g]	pre-exposure time	trial	time [min.]	oxygen level [mg/l]	activity
G. jazdzewski	perch	0.3442	0	71	13	8.71	30.35718
G. jazdzewski	perch	0.3442	0	71	14	8.69	34.28291
G. jazdzewski	perch	0.3442	0	71	15	8.67	37.45884
G. jazdzewski	perch	0.3442	0	71	16	8.66	35.40103
G. jazdzewski	perch	0.3442	0	71	17	8.65	31.48239
G. jazdzewski	perch	0.3442	0	71	18	8.62	35.39755
G. jazdzewski	perch	0.3442	0	71	19	8.61	35.53994
G. jazdzewski	perch	0.3442	0	71	20	8.6	32.82135
G. jazdzewski	perch	0.3442	0	71	21	8.59	29.53429
G. jazdzewski	perch	0.3442	0	71	22	8.57	33.18578
G. jazdzewski	perch	0.3442	0	71	23	8.57	30.84162
G. jazdzewski	perch	0.3442	0	71	24	8.57	30.06648
G. jazdzewski	perch	0.3442	0	71	25	8.56	34.3151
G. jazdzewski	perch	0.3442	0	71	26	8.53	25.18354
G. jazdzewski	perch	0.3442	0	71	27	8.52	29.81831
G. jazdzewski	perch	0.3442	0	71	28	8.5	30.38
G. jazdzewski	perch	0.3442	0	71	29	8.5	33.04381
G. jazdzewski	perch	0.3442	0	71	30	8.48	26.65421
G. jazdzewski	perch	0.2522	0	72	1	8.83	24.11977
G. jazdzewski	perch	0.2522	0	72	2	8.8	23.69994
G. jazdzewski	perch	0.2522	0	72	3	8.79	26.42029
G. jazdzewski	perch	0.2522	0	72	4	8.79	27.70068
G. jazdzewski	perch	0.2522	0	72	5	8.78	25.26094
G. jazdzewski	perch	0.2522	0	72	6	8.78	25.2185
G. jazdzewski	perch	0.2522	0	72	7	8.76	29.18349
G. jazdzewski	perch	0.2522	0	72	8	8.76	35.17955
G. jazdzewski	perch	0.2522	0	72	9	8.76	32.77728
G. jazdzewski	perch	0.2522	0	72	10	8.76	36.28293
G. jazdzewski	perch	0.2522	0	72	11	8.76	39.20255
G. jazdzewski	perch	0.2522	0	72	12	8.74	39.6757
G. jazdzewski	perch	0.2522	0	72	13	8.74	34.88558
G. jazdzewski	perch	0.2522	0	72	14	8.73	38.31607
G. jazdzewski	perch	0.2522	0	72	15	8.73	34.81672
G. jazdzewski	perch	0.2522	0	72	16	8.72	35.1066
G. jazdzewski	perch	0.2522	0	72	17	8.7	37.31531
G. jazdzewski	perch	0.2522	0	72	18	8.69	38.88728
G. jazdzewski	perch	0.2522	0	72	19	8.67	37.60142
G. jazdzewski	perch	0.2522	0	72	20	8.66	38.65589
G. jazdzewski	perch	0.2522	0	72	21	8.66	37.02557
G. jazdzewski	perch	0.2522	0	72	22	8.66	40.46451
G. jazdzewski	perch	0.2522	0	72	23	8.65	38.09861
G. jazdzewski	perch	0.2522	0	72	24	8.64	38.86171
G. jazdzewski	perch	0.2522	0	72	25	8.64	38.4001
G. jazdzewski	perch	0.2522	0	72	26	8.63	42.62005
G. jazdzewski	perch	0.2522	0	72	27	8.61	44.86183
G. jazdzewski	perch	0.2522	0	72	28	8.6	40.17457
G. jazdzewski	perch	0.2522	0	72	29	8.6	38.48758
G. jazdzewski	perch	0.2522	0	72	30	8.58	40.76014

Gammarid species	treatment	weight [g]	pre-exposure time	trial	time [min.]	oxygen level [mg/l]	activity
G. jazdzewski	perch	0.2561	0	73	1	8.87	31.5602
G. jazdzewski	perch	0.2561	0	73	2	8.87	26.0193
G. jazdzewski	perch	0.2561	0	73	3	8.86	26.96015
G. jazdzewski	perch	0.2561	0	73	4	8.86	28.92062
G. jazdzewski	perch	0.2561	0	73	5	8.84	22.77996
G. jazdzewski	perch	0.2561	0	73	6	8.85	26.78151
G. jazdzewski	perch	0.2561	0	73	7	8.84	27.70009
G. jazdzewski	perch	0.2561	0	73	8	8.8	29.52193
G. jazdzewski	perch	0.2561	0	73	9	8.8	33.44141
G. jazdzewski	perch	0.2561	0	73	10	8.83	30.16078
G. jazdzewski	perch	0.2561	0	73	11	8.83	30.38007
G. jazdzewski	perch	0.2561	0	73	12	8.8	30.90006
G. jazdzewski	perch	0.2561	0	73	13	8.78	26.93818
G. jazdzewski	perch	0.2561	0	73	14	8.69	28.84097
G. jazdzewski	perch	0.2561	0	73	15	8.68	34.24096
G. jazdzewski	perch	0.2561	0	73	16	8.66	33.52446
G. jazdzewski	perch	0.2561	0	73	17	8.62	33.55774
G. jazdzewski	perch	0.2561	0	73	18	8.7	30.23375
G. jazdzewski	perch	0.2561	0	73	19	8.71	24.68247
G. jazdzewski	perch	0.2561	0	73	20	8.7	27.43985
G. jazdzewski	perch	0.2561	0	73	21	8.66	28.20993
G. jazdzewski	perch	0.2561	0	73	22	8.63	29.65125
G. jazdzewski	perch	0.2561	0	73	23	8.6	31.24141
G. jazdzewski	perch	0.2561	0	73	24	8.61	32.78302
G. jazdzewski	perch	0.2561	0	73	25	8.61	31.57825
G. jazdzewski	perch	0.2561	0	73	26	8.58	30.82678
G. jazdzewski	perch	0.2561	0	73	27	8.59	30.25826
G. jazdzewski	perch	0.2561	0	73	28	8.57	28.7836
G. jazdzewski	perch	0.2561	0	73	29	8.56	28.71426
G. jazdzewski	perch	0.2561	0	73	30	8.61	30.44179
G. jazdzewski	perch	0.3318	0	74	1	8.84	26.99961
G. jazdzewski	perch	0.3318	0	74	2	8.82	23.8606
G. jazdzewski	perch	0.3318	0	74	3	8.81	28.30045
G. jazdzewski	perch	0.3318	0	74	4	8.79	39.50082
G. jazdzewski	perch	0.3318	0	74	5	8.76	26.31877
G. jazdzewski	perch	0.3318	0	74	6	8.75	26.4795
G. jazdzewski	perch	0.3318	0	74	7	8.73	28.87996
G. jazdzewski	perch	0.3318	0	74	8	8.71	27.68128
G. jazdzewski	perch	0.3318	0	74	9	8.7	31.14007
G. jazdzewski	perch	0.3318	0	74	10	8.67	30.79927
G. jazdzewski	perch	0.3318	0	74	11	8.65	30.06082
G. jazdzewski	perch	0.3318	0	74	12	8.64	29.11556
G. jazdzewski	perch	0.3318	0	74	13	8.63	28.56746
G. jazdzewski	perch	0.3318	0	74	14	8.63	24.01315
G. jazdzewski	perch	0.3318	0	74	15	8.62	26.06635
G. jazdzewski	perch	0.3318	0	74	16	8.62	29.47677
G. jazdzewski	perch	0.3318	0	74	17	8.61	26.26117
G. jazdzewski	perch	0.3318	0	74	18	8.6	28.62249

Gammarid species	treatment	weight [g]	pre-exposure time	trial	time [min.]	oxygen level [mg/l]	activity
G. jazdzewski	perch	0.3318	0	74	19	8.57	28.91875
G. jazdzewski	perch	0.3318	0	74	20	8.57	28.71995
G. jazdzewski	perch	0.3318	0	74	21	8.55	29.14566
G. jazdzewski	perch	0.3318	0	74	22	8.54	32.5171
G. jazdzewski	perch	0.3318	0	74	23	8.53	28.57846
G. jazdzewski	perch	0.3318	0	74	24	8.51	23.62325
G. jazdzewski	perch	0.3318	0	74	25	8.51	26.79674
G. jazdzewski	perch	0.3318	0	74	26	8.5	27.75996
G. jazdzewski	perch	0.3318	0	74	27	8.49	28.85996
G. jazdzewski	perch	0.3318	0	74	28	8.48	23.51814
G. jazdzewski	perch	0.3318	0	74	29	8.46	21.3638
G. jazdzewski	perch	0.3318	0	74	30	8.45	27.13409
G. jazdzewski	perch	0.3033	0	75	1	8.81	8.87974
G. jazdzewski	perch	0.3033	0	75	2	8.81	8.100229
G. jazdzewski	perch	0.3033	0	75	3	8.8	10.3214
G. jazdzewski	perch	0.3033	0	75	4	8.79	11.14019
G. jazdzewski	perch	0.3033	0	75	5	8.8	15.18063
G. jazdzewski	perch	0.3033	0	75	6	8.78	17.1191
G. jazdzewski	perch	0.3033	0	75	7	8.77	16.30122
G. jazdzewski	perch	0.3033	0	75	8	8.75	25.44005
G. jazdzewski	perch	0.3033	0	75	9	8.73	25.46149
G. jazdzewski	perch	0.3033	0	75	10	8.72	22.19631
G. jazdzewski	perch	0.3033	0	75	11	8.71	24.40072
G. jazdzewski	perch	0.3033	0	75	12	8.68	25.64165
G. jazdzewski	perch	0.3033	0	75	13	8.68	21.82001
G. jazdzewski	perch	0.3033	0	75	14	8.68	23.84306
G. jazdzewski	perch	0.3033	0	75	15	8.66	25.85584
G. jazdzewski	perch	0.3033	0	75	16	8.62	25.44106
G. jazdzewski	perch	0.3033	0	75	17	8.62	27.09999
G. jazdzewski	perch	0.3033	0	75	18	8.62	25.04
G. jazdzewski	perch	0.3033	0	75	19	8.61	23.64391
G. jazdzewski	perch	0.3033	0	75	20	8.6	27.48007
G. jazdzewski	perch	0.3033	0	75	21	8.59	24.65572
G. jazdzewski	perch	0.3033	0	75	22	8.58	29.20432
G. jazdzewski	perch	0.3033	0	75	23	8.56	26.83534
G. jazdzewski	perch	0.3033	0	75	24	8.55	27.60144
G. jazdzewski	perch	0.3033	0	75	25	8.53	26.01824
G. jazdzewski	perch	0.3033	0	75	26	8.5	23.16683
G. jazdzewski	perch	0.3033	0	75	27	8.51	27.12358
G. jazdzewski	perch	0.3033	0	75	28	8.49	29.27451
G. jazdzewski	perch	0.3033	0	75	29	8.48	24.2361
G. jazdzewski	perch	0.3033	0	75	30	8.49	28.76372
G. jazdzewski	perch	0.3668	0	76	1	8.84	19.18005
G. jazdzewski	perch	0.3668	0	76	2	8.81	23.12042
G. jazdzewski	perch	0.3668	0	76	3	8.8	24.86065
G. jazdzewski	perch	0.3668	0	76	4	8.79	26.85963
G. jazdzewski	perch	0.3668	0	76	5	8.79	23.30049
G. jazdzewski	perch	0.3668	0	76	6	8.77	23.23845

Gammarid species	treatment	weight [g]	pre-exposure time	trial	time [min.]	oxygen level [mg/l]	activity
G. jazdzewski	perch	0.3668	0	76	7	8.75	28.12162
G. jazdzewski	perch	0.3668	0	76	8	8.72	24.24004
G. jazdzewski	perch	0.3668	0	76	9	8.71	26.10284
G. jazdzewski	perch	0.3668	0	76	10	8.7	30.35627
G. jazdzewski	perch	0.3668	0	76	11	8.68	26.14077
G. jazdzewski	perch	0.3668	0	76	12	8.66	28.90438
G. jazdzewski	perch	0.3668	0	76	13	8.64	33.4163
G. jazdzewski	perch	0.3668	0	76	14	8.62	28.73995
G. jazdzewski	perch	0.3668	0	76	15	8.6	31.33877
G. jazdzewski	perch	0.3668	0	76	16	8.6	28.24554
G. jazdzewski	perch	0.3668	0	76	17	8.59	31.79885
G. jazdzewski	perch	0.3668	0	76	18	8.57	29.89876
G. jazdzewski	perch	0.3668	0	76	19	8.56	24.17859
G. jazdzewski	perch	0.3668	0	76	20	8.54	32.36392
G. jazdzewski	perch	0.3668	0	76	21	8.54	30.31999
G. jazdzewski	perch	0.3668	0	76	22	8.53	27.10304
G. jazdzewski	perch	0.3668	0	76	23	8.51	25.87386
G. jazdzewski	perch	0.3668	0	76	24	8.49	26.46797
G. jazdzewski	perch	0.3668	0	76	25	8.49	29.40344
G. jazdzewski	perch	0.3668	0	76	26	8.47	32.10971
G. jazdzewski	perch	0.3668	0	76	27	8.46	26.79245
G. jazdzewski	perch	0.3668	0	76	28	8.47	31.329
G. jazdzewski	perch	0.3668	0	76	29	8.45	32.32365
G. jazdzewski	perch	0.3668	0	76	30	8.42	28.52385
G. jazdzewski	perch	0.3025	0	77	1	8.88	21.02027
G. jazdzewski	perch	0.3025	0	77	2	8.87	18.19976
G. jazdzewski	perch	0.3025	0	77	3	8.84	19.96069
G. jazdzewski	perch	0.3025	0	77	4	8.82	23.99919
G. jazdzewski	perch	0.3025	0	77	5	8.8	27.65991
G. jazdzewski	perch	0.3025	0	77	6	8.78	22.14158
G. jazdzewski	perch	0.3025	0	77	7	8.78	24.09952
G. jazdzewski	perch	0.3025	0	77	8	8.76	29.75933
G. jazdzewski	perch	0.3025	0	77	9	8.75	29.34061
G. jazdzewski	perch	0.3025	0	77	10	8.74	31.96065
G. jazdzewski	perch	0.3025	0	77	11	8.73	30.20077
G. jazdzewski	perch	0.3025	0	77	12	8.72	29.86089
G. jazdzewski	perch	0.3025	0	77	13	8.71	23.37629
G. jazdzewski	perch	0.3025	0	77	14	8.69	28.0988
G. jazdzewski	perch	0.3025	0	77	15	8.68	20.32418
G. jazdzewski	perch	0.3025	0	77	16	8.67	24.96113
G. jazdzewski	perch	0.3025	0	77	17	8.66	27.43764
G. jazdzewski	perch	0.3025	0	77	18	8.63	30.56491
G. jazdzewski	perch	0.3025	0	77	19	8.64	23.73484
G. jazdzewski	perch	0.3025	0	77	20	8.62	25.02677
G. jazdzewski	perch	0.3025	0	77	21	8.62	27.97433
G. jazdzewski	perch	0.3025	0	77	22	8.6	25.38137
G. jazdzewski	perch	0.3025	0	77	23	8.59	27.62771
G. jazdzewski	perch	0.3025	0	77	24	8.57	24.27531

Gammarid species	treatment	weight [g]	pre-exposure time	trial	time [min.]	oxygen level [mg/l]	activity
G. jazdzewski	perch	0.3025	0	77	25	8.55	32.00664
G. jazdzewski	perch	0.3025	0	77	26	8.55	32.26002
G. jazdzewski	perch	0.3025	0	77	27	8.55	31.47824
G. jazdzewski	perch	0.3025	0	77	28	8.51	25.48004
G. jazdzewski	perch	0.3025	0	77	29	8.5	22.27816
G. jazdzewski	perch	0.3025	0	77	30	8.48	28.26193
G. jazdzewski	perch	0.2409	0	78	1	8.83	24.22065
G. jazdzewski	perch	0.2409	0	78	2	8.83	25.23982
G. jazdzewski	perch	0.2409	0	78	3	8.83	26.76104
G. jazdzewski	perch	0.2409	0	78	4	8.82	29.59887
G. jazdzewski	perch	0.2409	0	78	5	8.83	34.73989
G. jazdzewski	perch	0.2409	0	78	6	8.81	30.78203
G. jazdzewski	perch	0.2409	0	78	7	8.81	27.45954
G. jazdzewski	perch	0.2409	0	78	8	8.78	30.90204
G. jazdzewski	perch	0.2409	0	78	9	8.77	28.41942
G. jazdzewski	perch	0.2409	0	78	10	8.77	33.08003
G. jazdzewski	perch	0.2409	0	78	11	8.76	35.8
G. jazdzewski	perch	0.2409	0	78	12	8.75	33.30003
G. jazdzewski	perch	0.2409	0	78	13	8.73	33.94193
G. jazdzewski	perch	0.2409	0	78	14	8.75	36.314
G. jazdzewski	perch	0.2409	0	78	15	8.72	30.90949
G. jazdzewski	perch	0.2409	0	78	16	8.7	35.09683
G. jazdzewski	perch	0.2409	0	78	17	8.7	35.06245
G. jazdzewski	perch	0.2409	0	78	18	8.7	33.77889
G. jazdzewski	perch	0.2409	0	78	19	8.67	35.73341
G. jazdzewski	perch	0.2409	0	78	20	8.68	29.80389
G. jazdzewski	perch	0.2409	0	78	21	8.66	30.55993
G. jazdzewski	perch	0.2409	0	78	22	8.65	36.96137
G. jazdzewski	perch	0.2409	0	78	23	8.63	31.28779
G. jazdzewski	perch	0.2409	0	78	24	8.63	35.13379
G. jazdzewski	perch	0.2409	0	78	25	8.63	38.125
G. jazdzewski	perch	0.2409	0	78	26	8.6	33.81318
G. jazdzewski	perch	0.2409	0	78	27	8.59	39.97804
G. jazdzewski	perch	0.2409	0	78	28	8.61	33.02915
G. jazdzewski	perch	0.2409	0	78	29	8.57	34.62009
G. jazdzewski	perch	0.2409	0	78	30	8.56	36.73801
G. jazdzewski	perch	0.282	0	80	1	8.8	29.02056
G. jazdzewski	perch	0.282	0	80	2	8.78	25.64063
G. jazdzewski	perch	0.282	0	80	3	8.8	34.08009
G. jazdzewski	perch	0.282	0	80	4	8.79	30.79881
G. jazdzewski	perch	0.282	0	80	5	8.77	25.4004
G. jazdzewski	perch	0.282	0	80	6	8.77	26.70207
G. jazdzewski	perch	0.282	0	80	7	8.76	27.94073
G. jazdzewski	perch	0.282	0	80	8	8.74	31.77751
G. jazdzewski	perch	0.282	0	80	9	8.75	29.84203
G. jazdzewski	perch	0.282	0	80	10	8.73	32.36233
G. jazdzewski	perch	0.282	0	80	11	8.71	32.71596
G. jazdzewski	perch	0.282	0	80	12	8.7	32.97985

Gammarid species	treatment	weight [g]	pre-exposure time	trial	time [min.]	oxygen level [mg/l]	activity
G. jazdzewski	perch	0.282	0	80	13	8.7	33.62465
G. jazdzewski	perch	0.282	0	80	14	8.69	36.67604
G. jazdzewski	perch	0.282	0	80	15	8.68	32.802
G. jazdzewski	perch	0.282	0	80	16	8.67	30.88
G. jazdzewski	perch	0.282	0	80	17	8.65	36.71634
G. jazdzewski	perch	0.282	0	80	18	8.63	31.80225
G. jazdzewski	perch	0.282	0	80	19	8.63	30.76779
G. jazdzewski	perch	0.282	0	80	20	8.59	33.03463
G. jazdzewski	perch	0.282	0	80	21	8.59	29.56282
G. jazdzewski	perch	0.282	0	80	22	8.58	37.24149
G. jazdzewski	perch	0.282	0	80	23	8.57	32.99542
G. jazdzewski	perch	0.282	0	80	24	8.57	34.48476
G. jazdzewski	perch	0.282	0	80	25	8.55	35.46001
G. jazdzewski	perch	0.282	0	80	26	8.55	37.5652
G. jazdzewski	perch	0.282	0	80	27	8.53	32.37121
G. jazdzewski	perch	0.282	0	80	28	8.53	34.32176
G. jazdzewski	perch	0.282	0	80	29	8.51	30.68767
G. jazdzewski	perch	0.282	0	80	30	8.48	33.81817
G. jazdzewski	perch	0.2702	0	81	1	8.88	26.72034
G. jazdzewski	perch	0.2702	0	81	2	8.84	29.34032
G. jazdzewski	perch	0.2702	0	81	3	8.82	29.49971
G. jazdzewski	perch	0.2702	0	81	4	8.81	36.78076
G. jazdzewski	perch	0.2702	0	81	5	8.79	31.31998
G. jazdzewski	perch	0.2702	0	81	6	8.79	35.28042
G. jazdzewski	perch	0.2702	0	81	7	8.77	28.0018
G. jazdzewski	perch	0.2702	0	81	8	8.77	34.18013
G. jazdzewski	perch	0.2702	0	81	9	8.76	29.85802
G. jazdzewski	perch	0.2702	0	81	10	8.74	33.32075
G. jazdzewski	perch	0.2702	0	81	11	8.72	35.88002
G. jazdzewski	perch	0.2702	0	81	12	8.74	34.57821
G. jazdzewski	perch	0.2702	0	81	13	8.7	33.68471
G. jazdzewski	perch	0.2702	0	81	14	8.7	36.01814
G. jazdzewski	perch	0.2702	0	81	15	8.67	27.33907
G. jazdzewski	perch	0.2702	0	81	16	8.67	34.63888
G. jazdzewski	perch	0.2702	0	81	17	8.65	36.92222
G. jazdzewski	perch	0.2702	0	81	18	8.64	28.40001
G. jazdzewski	perch	0.2702	0	81	19	8.63	37.18257
G. jazdzewski	perch	0.2702	0	81	20	8.64	30.71873
G. jazdzewski	perch	0.2702	0	81	21	8.61	34.7557
G. jazdzewski	perch	0.2702	0	81	22	8.61	32.38129
G. jazdzewski	perch	0.2702	0	81	23	8.6	40.1844
G. jazdzewski	perch	0.2702	0	81	24	8.58	33.20633
G. jazdzewski	perch	0.2702	0	81	25	8.58	30.31171
G. jazdzewski	perch	0.2702	0	81	26	8.57	28.6468
G. jazdzewski	perch	0.2702	0	81	27	8.54	29.69287
G. jazdzewski	perch	0.2702	0	81	28	8.53	31.28723
G. jazdzewski	perch	0.2702	0	81	29	8.52	27.39433
G. jazdzewski	perch	0.2702	0	81	30	8.52	32.48776

Gammarid species	treatment	weight [g]	pre-exposure time	trial	time [min.]	oxygen level [mg/l]	activity
G. jazdzewski	perch	0.3003	0	82	1	8.87	15.57955
G. jazdzewski	perch	0.3003	0	82	2	8.84	18.10108
G. jazdzewski	perch	0.3003	0	82	3	8.83	20.80105
G. jazdzewski	perch	0.3003	0	82	4	8.8	19.0992
G. jazdzewski	perch	0.3003	0	82	5	8.78	27.04171
G. jazdzewski	perch	0.3003	0	82	6	8.76	17.95958
G. jazdzewski	perch	0.3003	0	82	7	8.76	22.98181
G. jazdzewski	perch	0.3003	0	82	8	8.76	19.27958
G. jazdzewski	perch	0.3003	0	82	9	8.73	19.51938
G. jazdzewski	perch	0.3003	0	82	10	8.72	19.78082
G. jazdzewski	perch	0.3003	0	82	11	8.7	21.05757
G. jazdzewski	perch	0.3003	0	82	12	8.68	26.12166
G. jazdzewski	perch	0.3003	0	82	13	8.67	25.94088
G. jazdzewski	perch	0.3003	0	82	14	8.67	27.082
G. jazdzewski	perch	0.3003	0	82	15	8.65	30.67573
G. jazdzewski	perch	0.3003	0	82	16	8.64	24.49988
G. jazdzewski	perch	0.3003	0	82	17	8.63	25.03989
G. jazdzewski	perch	0.3003	0	82	18	8.63	27.74615
G. jazdzewski	perch	0.3003	0	82	19	8.61	29.49616
G. jazdzewski	perch	0.3003	0	82	20	8.6	26.49999
G. jazdzewski	perch	0.3003	0	82	21	8.58	22.22002
G. jazdzewski	perch	0.3003	0	82	22	8.58	30.25994
G. jazdzewski	perch	0.3003	0	82	23	8.57	31.30148
G. jazdzewski	perch	0.3003	0	82	24	8.56	28.58162
G. jazdzewski	perch	0.3003	0	82	25	8.55	29.15838
G. jazdzewski	perch	0.3003	0	82	26	8.54	37.52508
G. jazdzewski	perch	0.3003	0	82	27	8.53	28.47648
G. jazdzewski	perch	0.3003	0	82	28	8.51	31.24177
G. jazdzewski	perch	0.3003	0	82	29	8.49	30.03999
G. jazdzewski	perch	0.3003	0	82	30	8.48	27.99602
G. jazdzewski	perch	0.2633	0	83	1	8.82	20.55932
G. jazdzewski	perch	0.2633	0	83	2	8.81	23.80103
G. jazdzewski	perch	0.2633	0	83	3	8.81	22.44007
G. jazdzewski	perch	0.2633	0	83	4	8.81	26.59835
G. jazdzewski	perch	0.2633	0	83	5	8.79	31.94214
G. jazdzewski	perch	0.2633	0	83	6	8.79	29.55791
G. jazdzewski	perch	0.2633	0	83	7	8.79	35.6245
G. jazdzewski	perch	0.2633	0	83	8	8.78	34.34008
G. jazdzewski	perch	0.2633	0	83	9	8.77	36.48006
G. jazdzewski	perch	0.2633	0	83	10	8.75	35.47923
G. jazdzewski	perch	0.2633	0	83	11	8.74	33.62411
G. jazdzewski	perch	0.2633	0	83	12	8.72	37.6792
G. jazdzewski	perch	0.2633	0	83	13	8.71	34.92009
G. jazdzewski	perch	0.2633	0	83	14	8.7	46.382
G. jazdzewski	perch	0.2633	0	83	15	8.7	41.96011
G. jazdzewski	perch	0.2633	0	83	16	8.68	43.81896
G. jazdzewski	perch	0.2633	0	83	17	8.68	35.78127
G. jazdzewski	perch	0.2633	0	83	18	8.66	34.72391

Gammarid species	treatment	weight [g]	pre-exposure time	trial	time [min.]	oxygen level [mg/l]	activity
G. jazdzewski	perch	0.2633	0	83	19	8.63	31.55369
G. jazdzewski	perch	0.2633	0	83	20	8.62	28.9441
G. jazdzewski	perch	0.2633	0	83	21	8.62	30.51435
G. jazdzewski	perch	0.2633	0	83	22	8.59	37.7688
G. jazdzewski	perch	0.2633	0	83	23	8.6	42.94154
G. jazdzewski	perch	0.2633	0	83	24	8.59	34.19204
G. jazdzewski	perch	0.2633	0	83	25	8.58	35.46653
G. jazdzewski	perch	0.2633	0	83	26	8.54	33.53481
G. jazdzewski	perch	0.2633	0	83	27	8.54	36.88696
G. jazdzewski	perch	0.2633	0	83	28	8.5	36.9544
G. jazdzewski	perch	0.2633	0	83	29	8.5	33.23326
G. jazdzewski	perch	0.2633	0	83	30	8.51	40.56827
G. jazdzewski	perch	0.2654	0	84	1	8.81	16.11961
G. jazdzewski	perch	0.2654	0	84	2	8.79	12.0006
G. jazdzewski	perch	0.2654	0	84	3	8.75	15.47901
G. jazdzewski	perch	0.2654	0	84	4	8.75	16.44031
G. jazdzewski	perch	0.2654	0	84	5	8.75	20.74136
G. jazdzewski	perch	0.2654	0	84	6	8.73	28.24157
G. jazdzewski	perch	0.2654	0	84	7	8.75	27.23836
G. jazdzewski	perch	0.2654	0	84	8	8.75	29.60257
G. jazdzewski	perch	0.2654	0	84	9	8.73	26.45877
G. jazdzewski	perch	0.2654	0	84	10	8.72	24.23934
G. jazdzewski	perch	0.2654	0	84	11	8.71	25.49921
G. jazdzewski	perch	0.2654	0	84	12	8.68	24.04265
G. jazdzewski	perch	0.2654	0	84	13	8.67	27.79913
G. jazdzewski	perch	0.2654	0	84	14	8.67	24.15904
G. jazdzewski	perch	0.2654	0	84	15	8.65	23.90217
G. jazdzewski	perch	0.2654	0	84	16	8.66	29.02228
G. jazdzewski	perch	0.2654	0	84	17	8.66	34.43641
G. jazdzewski	perch	0.2654	0	84	18	8.64	28.64492
G. jazdzewski	perch	0.2654	0	84	19	8.63	28.57486
G. jazdzewski	perch	0.2654	0	84	20	8.63	24.32132
G. jazdzewski	perch	0.2654	0	84	21	8.62	29.49844
G. jazdzewski	perch	0.2654	0	84	22	8.6	24.96138
G. jazdzewski	perch	0.2654	0	84	23	8.59	21.91996
G. jazdzewski	perch	0.2654	0	84	24	8.58	22.91834
G. jazdzewski	perch	0.2654	0	84	25	8.57	25.60657
G. jazdzewski	perch	0.2654	0	84	26	8.55	24.35831
G. jazdzewski	perch	0.2654	0	84	27	8.54	19.22364
G. jazdzewski	perch	0.2654	0	84	28	8.54	26.88003
G. jazdzewski	perch	0.2654	0	84	29	8.53	23.62001
G. jazdzewski	perch	0.2654	0	84	30	8.51	28.65997
D. villosus	no predator	0.7755	1	91	1	8.55	11.54012
D. villosus	no predator	0.7755	1	91	2	8.48	25.74028
D. villosus	no predator	0.7755	1	91	3	8.33	11.5394
D. villosus	no predator	0.7755	1	91	4	8.35	10.92001
D. villosus	no predator	0.7755	1	91	5	8.23	10.85905
D. villosus	no predator	0.7755	1	91	6	8.28	17.16143

Gammarid species	treatment	weight [g]	pre-exposure time	trial	time [min.]	oxygen level [mg/l]	activity
D. villosus	no predator	0.7755	1	91	7	8.1	14.11825
D. villosus	no predator	0.7755	1	91	8	8.08	6.382571
D. villosus	no predator	0.7755	1	91	9	8.15	16.87935
D. villosus	no predator	0.7755	1	91	10	8.15	20.72075
D. villosus	no predator	0.7755	1	91	11	7.9	11.41844
D. villosus	no predator	0.7755	1	91	12	7.45	17.13993
D. villosus	no predator	0.7755	1	91	13	7.54	10.36098
D. villosus	no predator	0.7755	1	91	14	7.75	10.63804
D. villosus	no predator	0.7755	1	91	15	7.79	12.86318
D. villosus	no predator	0.7755	1	91	16	7.73	13.88238
D. villosus	no predator	0.7755	1	91	17	7.61	15.87541
D. villosus	no predator	0.7755	1	91	18	7.54	12.49875
D. villosus	no predator	0.7755	1	91	19	7.74	18.16384
D. villosus	no predator	0.7755	1	91	20	7.65	19.95589
D. villosus	no predator	0.7755	1	91	21	7.64	16.59845
D. villosus	no predator	0.7755	1	91	22	7.45	17.84732
D. villosus	no predator	0.7755	1	91	23	7.42	19.20007
D. villosus	no predator	0.7755	1	91	24	7.49	13.35842
D. villosus	no predator	0.7755	1	91	25	7.32	9.91666
D. villosus	no predator	0.7755	1	91	26	7.24	13.2816
D. villosus	no predator	0.7755	1	91	27	6.89	15.70345
D. villosus	no predator	0.7755	1	91	28	6.74	10.41297
D. villosus	no predator	0.7755	1	91	29	6.88	19.22877
D. villosus	no predator	0.7755	1	91	30	6.62	22.02386
D. villosus	no predator	0.5959	1	92	1	8.84	14.87913
D. villosus	no predator	0.5959	1	92	2	8.83	10.86019
D. villosus	no predator	0.5959	1	92	3	8.8	11.71994
D. villosus	no predator	0.5959	1	92	4	8.78	11.92078
D. villosus	no predator	0.5959	1	92	5	8.75	16.08003
D. villosus	no predator	0.5959	1	92	6	8.74	16.78055
D. villosus	no predator	0.5959	1	92	7	8.7	15.62008
D. villosus	no predator	0.5959	1	92	8	8.69	12.72
D. villosus	no predator	0.5959	1	92	9	8.66	7.440769
D. villosus	no predator	0.5959	1	92	10	8.6	17.19924
D. villosus	no predator	0.5959	1	92	11	8.55	12.88082
D. villosus	no predator	0.5959	1	92	12	8.45	20.19907
D. villosus	no predator	0.5959	1	92	13	8.27	13.48
D. villosus	no predator	0.5959	1	92	14	8.33	18.96094
D. villosus	no predator	0.5959	1	92	15	8.31	13.77788
D. villosus	no predator	0.5959	1	92	16	8.27	14.91992
D. villosus	no predator	0.5959	1	92	17	8.31	12.00234
D. villosus	no predator	0.5959	1	92	18	8.29	11.00134
D. villosus	no predator	0.5959	1	92	19	8.3	19.47873
D. villosus	no predator	0.5959	1	92	20	8.23	18.10277
D. villosus	no predator	0.5959	1	92	21	8.19	17.47294
D. villosus	no predator	0.5959	1	92	22	8.16	16.31985
D. villosus	no predator	0.5959	1	92	23	8.19	15.10454
D. villosus	no predator	0.5959	1	92	24	8.19	22.07995

Gammarid species	treatment	weight [g]	pre-exposure time	trial	time [min.]	oxygen level [mg/l]	activity
D. villosus	no predator	0.5959	1	92	25	8.12	12.65832
D. villosus	no predator	0.5959	1	92	26	8.14	16.62163
D. villosus	no predator	0.5959	1	92	27	8.16	19.19813
D. villosus	no predator	0.5959	1	92	28	8.14	13.46737
D. villosus	no predator	0.5959	1	92	29	8.14	13.9144
D. villosus	no predator	0.5959	1	92	30	7.92	17.9859
D. villosus	no predator	0.7358	1	93	1	8.47	2.780512
D. villosus	no predator	0.7358	1	93	2	8.45	8.700701
D. villosus	no predator	0.7358	1	93	3	8.43	9.058766
D. villosus	no predator	0.7358	1	93	4	8.38	10.31995
D. villosus	no predator	0.7358	1	93	5	8.37	8.479981
D. villosus	no predator	0.7358	1	93	6	8.35	14.601
D. villosus	no predator	0.7358	1	93	7	8.33	9.600098
D. villosus	no predator	0.7358	1	93	8	8.33	8.959367
D. villosus	no predator	0.7358	1	93	9	8.3	10.06067
D. villosus	no predator	0.7358	1	93	10	8.28	10.76158
D. villosus	no predator	0.7358	1	93	11	8.26	14.87841
D. villosus	no predator	0.7358	1	93	12	8.24	12.5418
D. villosus	no predator	0.7358	1	93	13	8.2	13.79716
D. villosus	no predator	0.7358	1	93	14	8.18	14.17985
D. villosus	no predator	0.7358	1	93	15	8.17	12.02312
D. villosus	no predator	0.7358	1	93	16	8.16	9.922361
D. villosus	no predator	0.7358	1	93	17	8.13	11.83898
D. villosus	no predator	0.7358	1	93	18	8.13	13.37999
D. villosus	no predator	0.7358	1	93	19	8.09	12.06125
D. villosus	no predator	0.7358	1	93	20	8.07	4.618696
D. villosus	no predator	0.7358	1	93	21	8.07	5.904325
D. villosus	no predator	0.7358	1	93	22	8.03	13.7986
D. villosus	no predator	0.7358	1	93	23	8.02	13.62005
D. villosus	no predator	0.7358	1	93	24	8	11.05846
D. villosus	no predator	0.7358	1	93	25	7.98	5.856704
D. villosus	no predator	0.7358	1	93	26	7.96	12.28335
D. villosus	no predator	0.7358	1	93	27	7.94	10.41821
D. villosus	no predator	0.7358	1	93	28	7.92	6.078139
D. villosus	no predator	0.7358	1	93	29	7.92	5.923784
D. villosus	no predator	0.7358	1	93	30	7.88	9.543985
D. villosus	no predator	0.5665	1	94	1	8.79	9.480442
D. villosus	no predator	0.5665	1	94	2	8.76	11.29976
D. villosus	no predator	0.5665	1	94	3	8.73	9.559668
D. villosus	no predator	0.5665	1	94	4	8.68	16.5411
D. villosus	no predator	0.5665	1	94	5	8.6	15.18002
D. villosus	no predator	0.5665	1	94	6	8.6	17.79946
D. villosus	no predator	0.5665	1	94	7	8.59	16.3799
D. villosus	no predator	0.5665	1	94	8	8.58	9.882611
D. villosus	no predator	0.5665	1	94	9	8.57	17.87795
D. villosus	no predator	0.5665	1	94	10	8.54	16.20224
D. villosus	no predator	0.5665	1	94	11	8.52	17.57922
D. villosus	no predator	0.5665	1	94	12	8.5	16.43731

Gammarid species	treatment	weight [g]	pre-exposure time	trial	time [min.]	oxygen level [mg/l]	activity
D. villosus	no predator	0.5665	1	94	13	8.5	15.82464
D. villosus	no predator	0.5665	1	94	14	8.48	18.71605
D. villosus	no predator	0.5665	1	94	15	8.46	17.56096
D. villosus	no predator	0.5665	1	94	16	8.43	16.57996
D. villosus	no predator	0.5665	1	94	17	8.41	17.34356
D. villosus	no predator	0.5665	1	94	18	8.38	15.24262
D. villosus	no predator	0.5665	1	94	19	8.37	23.07742
D. villosus	no predator	0.5665	1	94	20	8.36	21.27581
D. villosus	no predator	0.5665	1	94	21	8.33	17.48703
D. villosus	no predator	0.5665	1	94	22	8.31	11.62011
D. villosus	no predator	0.5665	1	94	23	8.29	17.29851
D. villosus	no predator	0.5665	1	94	24	8.27	19.81999
D. villosus	no predator	0.5665	1	94	25	8.23	16.31666
D. villosus	no predator	0.5665	1	94	26	8.2	12.59994
D. villosus	no predator	0.5665	1	94	27	8.19	12.70174
D. villosus	no predator	0.5665	1	94	28	8.17	16.60553
D. villosus	no predator	0.5665	1	94	29	8.15	20.77816
D. villosus	no predator	0.5665	1	94	30	8.13	24.39803
D. villosus	no predator	0.6328	1	95	1	8.76	17.50067
D. villosus	no predator	0.6328	1	95	2	8.76	11.45869
D. villosus	no predator	0.6328	1	95	3	8.75	6.840656
D. villosus	no predator	0.6328	1	95	4	8.74	12.11999
D. villosus	no predator	0.6328	1	95	5	8.72	14.0419
D. villosus	no predator	0.6328	1	95	6	8.7	18.14017
D. villosus	no predator	0.6328	1	95	7	8.69	14.81895
D. villosus	no predator	0.6328	1	95	8	8.69	11.73935
D. villosus	no predator	0.6328	1	95	9	8.67	8.080692
D. villosus	no predator	0.6328	1	95	10	8.65	9.020813
D. villosus	no predator	0.6328	1	95	11	8.63	16.01918
D. villosus	no predator	0.6328	1	95	12	8.58	12.81999
D. villosus	no predator	0.6328	1	95	13	8.46	11.9
D. villosus	no predator	0.6328	1	95	14	8.44	10.56098
D. villosus	no predator	0.6328	1	95	15	8.44	6.999991
D. villosus	no predator	0.6328	1	95	16	8.44	15.62218
D. villosus	no predator	0.6328	1	95	17	8.46	17.3176
D. villosus	no predator	0.6328	1	95	18	8.44	16.99987
D. villosus	no predator	0.6328	1	95	19	8.42	12.32655
D. villosus	no predator	0.6328	1	95	20	8.42	19.07879
D. villosus	no predator	0.6328	1	95	21	8.42	14.15433
D. villosus	no predator	0.6328	1	95	22	8.38	13.21987
D. villosus	no predator	0.6328	1	95	23	8.36	14.12611
D. villosus	no predator	0.6328	1	95	24	8.34	14.99361
D. villosus	no predator	0.6328	1	95	25	8.33	11.40494
D. villosus	no predator	0.6328	1	95	26	8.33	14.74178
D. villosus	no predator	0.6328	1	95	27	8.31	14.2001
D. villosus	no predator	0.6328	1	95	28	8.3	15.04182
D. villosus	no predator	0.6328	1	95	29	8.29	15.33811
D. villosus	no predator	0.6328	1	95	30	8.28	15.17798

Gammarid species	treatment	weight [g]	pre-exposure time	trial	time [min.]	oxygen level [mg/l]	activity
D. villosus	no predator	0.7735	1	96	1	8.64	9.639904
D. villosus	no predator	0.7735	1	96	2	8.61	5.659944
D. villosus	no predator	0.7735	1	96	3	8.61	4.180318
D. villosus	no predator	0.7735	1	96	4	8.6	4.879596
D. villosus	no predator	0.7735	1	96	5	8.58	2.319976
D. villosus	no predator	0.7735	1	96	6	8.57	7.981
D. villosus	no predator	0.7735	1	96	7	8.56	9.339427
D. villosus	no predator	0.7735	1	96	8	8.55	5.281988
D. villosus	no predator	0.7735	1	96	9	8.54	5.800123
D. villosus	no predator	0.7735	1	96	10	8.53	10.92009
D. villosus	no predator	0.7735	1	96	11	8.43	9.99757
D. villosus	no predator	0.7735	1	96	12	8.4	5.859942
D. villosus	no predator	0.7735	1	96	13	8.4	9.719908
D. villosus	no predator	0.7735	1	96	14	8.33	3.222008
D. villosus	no predator	0.7735	1	96	15	8.35	4.457915
D. villosus	no predator	0.7735	1	96	16	8.39	1.799981
D. villosus	no predator	0.7735	1	96	17	8.39	0.519995
D. villosus	no predator	0.7735	1	96	18	8.32	3.019969
D. villosus	no predator	0.7735	1	96	19	8.26	3.579968
D. villosus	no predator	0.7735	1	96	20	8.24	4.641336
D. villosus	no predator	0.7735	1	96	21	8.23	5.401452
D. villosus	no predator	0.7735	1	96	22	8.22	4.900072
D. villosus	no predator	0.7735	1	96	23	8.21	5.321628
D. villosus	no predator	0.7735	1	96	24	8.21	6.316818
D. villosus	no predator	0.7735	1	96	25	8.21	0.87999
D. villosus	no predator	0.7735	1	96	26	8.21	12.44163
D. villosus	no predator	0.7735	1	96	27	8.2	11.21995
D. villosus	no predator	0.7735	1	96	28	8.2	6.741854
D. villosus	no predator	0.7735	1	96	29	8.19	5.358145
D. villosus	no predator	0.7735	1	96	30	8.17	9.461941
D. villosus	no predator	0.5669	1	97	1	8.44	4.000202
D. villosus	no predator	0.5669	1	97	2	8.41	5.659709
D. villosus	no predator	0.5669	1	97	3	8.41	5.180669
D. villosus	no predator	0.5669	1	97	4	8.38	7.500043
D. villosus	no predator	0.5669	1	97	5	8.36	4.439118
D. villosus	no predator	0.5669	1	97	6	8.35	2.399975
D. villosus	no predator	0.5669	1	97	7	8.34	4.19996
D. villosus	no predator	0.5669	1	97	8	8.33	7.840584
D. villosus	no predator	0.5669	1	97	9	8.31	4.419296
D. villosus	no predator	0.5669	1	97	10	8.29	3.839965
D. villosus	no predator	0.5669	1	97	11	8.27	2.660813
D. villosus	no predator	0.5669	1	97	12	8.26	4.519114
D. villosus	no predator	0.5669	1	97	13	8.14	3.05997
D. villosus	no predator	0.5669	1	97	14	8.09	0.879992
D. villosus	no predator	0.5669	1	97	15	8.07	3.479965
D. villosus	no predator	0.5669	1	97	16	8.06	3.379967
D. villosus	no predator	0.5669	1	97	17	8.08	1.03999
D. villosus	no predator	0.5669	1	97	18	8.08	2.279976

Gammarid species	treatment	weight [g]	pre-exposure time	trial	time [min.]	oxygen level [mg/l]	activity
D. villosus	no predator	0.5669	1	97	19	8.08	2.03998
D. villosus	no predator	0.5669	1	97	20	8.09	2.219978
D. villosus	no predator	0.5669	1	97	21	8.1	2.139981
D. villosus	no predator	0.5669	1	97	22	8.1	3.439963
D. villosus	no predator	0.5669	1	97	23	8.1	0.119999
D. villosus	no predator	0.5669	1	97	24	8.1	2.561595
D. villosus	no predator	0.5669	1	97	25	8.09	1.098369
D. villosus	no predator	0.5669	1	97	26	8.08	1.699983
D. villosus	no predator	0.5669	1	97	27	8.09	2.359977
D. villosus	no predator	0.5669	1	97	28	8.07	1.439985
D. villosus	no predator	0.5669	1	97	29	8.05	0.95999
D. villosus	no predator	0.5669	1	97	30	8.05	3.13997
D. villosus	no predator	0.6986	1	98	1	8.62	4.140198
D. villosus	no predator	0.6986	1	98	2	8.61	6.339943
D. villosus	no predator	0.6986	1	98	3	8.6	4.000324
D. villosus	no predator	0.6986	1	98	4	8.56	8.799556
D. villosus	no predator	0.6986	1	98	5	8.55	9.281821
D. villosus	no predator	0.6986	1	98	6	8.54	14.20047
D. villosus	no predator	0.6986	1	98	7	8.5	7.398849
D. villosus	no predator	0.6986	1	98	8	8.48	6.60125
D. villosus	no predator	0.6986	1	98	9	8.46	11.39923
D. villosus	no predator	0.6986	1	98	10	8.45	2.90153
D. villosus	no predator	0.6986	1	98	11	8.43	4.798384
D. villosus	no predator	0.6986	1	98	12	8.41	8.121712
D. villosus	no predator	0.6986	1	98	13	8.39	8.198126
D. villosus	no predator	0.6986	1	98	14	8.38	10.22194
D. villosus	no predator	0.6986	1	98	15	8.36	11.48433
D. villosus	no predator	0.6986	1	98	16	8.34	10.17797
D. villosus	no predator	0.6986	1	98	17	8.32	10.07648
D. villosus	no predator	0.6986	1	98	18	8.31	7.163717
D. villosus	no predator	0.6986	1	98	19	8.28	9.860077
D. villosus	no predator	0.6986	1	98	20	8.27	11.4587
D. villosus	no predator	0.6986	1	98	21	8.24	12.04144
D. villosus	no predator	0.6986	1	98	22	8.22	8.320102
D. villosus	no predator	0.6986	1	98	23	8.21	14.54466
D. villosus	no predator	0.6986	1	98	24	8.19	10.19528
D. villosus	no predator	0.6986	1	98	25	8.17	7.605031
D. villosus	no predator	0.6986	1	98	26	8.14	14.47836
D. villosus	no predator	0.6986	1	98	27	8.09	11.43821
D. villosus	no predator	0.6986	1	98	28	8.07	16.58549
D. villosus	no predator	0.6986	1	98	29	8.07	15.19241
D. villosus	no predator	0.6986	1	98	30	8.05	13.12187
D. villosus	no predator	0.6554	1	99	1	8.5	7.539934
D. villosus	no predator	0.6554	1	99	2	8.49	5.279951
D. villosus	no predator	0.6554	1	99	3	8.47	3.700323
D. villosus	no predator	0.6554	1	99	4	8.45	5.49959
D. villosus	no predator	0.6554	1	99	5	8.43	6.419932
D. villosus	no predator	0.6554	1	99	6	8.42	5.999943

Gammarid species	treatment	weight [g]	pre-exposure time	trial	time [min.]	oxygen level [mg/l]	activity
D. villosus	no predator	0.6554	1	99	7	8.39	8.000521
D. villosus	no predator	0.6554	1	99	8	8.37	5.479349
D. villosus	no predator	0.6554	1	99	9	8.36	9.861349
D. villosus	no predator	0.6554	1	99	10	8.32	8.820815
D. villosus	no predator	0.6554	1	99	11	8.31	8.360108
D. villosus	no predator	0.6554	1	99	12	8.32	10.17918
D. villosus	no predator	0.6554	1	99	13	8.28	10.80097
D. villosus	no predator	0.6554	1	99	14	8.28	9.680028
D. villosus	no predator	0.6554	1	99	15	8.26	7.53999
D. villosus	no predator	0.6554	1	99	16	8.26	6.841129
D. villosus	no predator	0.6554	1	99	17	8.24	8.657637
D. villosus	no predator	0.6554	1	99	18	8.23	8.784955
D. villosus	no predator	0.6554	1	99	19	8.21	9.997499
D. villosus	no predator	0.6554	1	99	20	8.23	10.19727
D. villosus	no predator	0.6554	1	99	21	8.2	12.55988
D. villosus	no predator	0.6554	1	99	22	8.14	9.005905
D. villosus	no predator	0.6554	1	99	23	8.11	10.77858
D. villosus	no predator	0.6554	1	99	24	8.12	9.338416
D. villosus	no predator	0.6554	1	99	25	8.11	8.1383
D. villosus	no predator	0.6554	1	99	26	8.12	12.14162
D. villosus	no predator	0.6554	1	99	27	8.09	8.361794
D. villosus	no predator	0.6554	1	99	28	8.11	11.20176
D. villosus	no predator	0.6554	1	99	29	8.09	11.84188
D. villosus	no predator	0.6554	1	99	30	8.08	13.32197
D. villosus	no predator	0.6264	1	100	1	8.59	7.600048
D. villosus	no predator	0.6264	1	100	2	8.58	4.660015
D. villosus	no predator	0.6264	1	100	3	8.56	9.300328
D. villosus	no predator	0.6264	1	100	4	8.54	9.660814
D. villosus	no predator	0.6264	1	100	5	8.52	11.08001
D. villosus	no predator	0.6264	1	100	6	8.5	3.799482
D. villosus	no predator	0.6264	1	100	7	8.47	10.0011
D. villosus	no predator	0.6264	1	100	8	8.47	10.30062
D. villosus	no predator	0.6264	1	100	9	8.45	12.50145
D. villosus	no predator	0.6264	1	100	10	8.44	8.8386
D. villosus	no predator	0.6264	1	100	11	8.43	10.60332
D. villosus	no predator	0.6264	1	100	12	8.41	9.776541
D. villosus	no predator	0.6264	1	100	13	8.39	3.819964
D. villosus	no predator	0.6264	1	100	14	8.38	8.400937
D. villosus	no predator	0.6264	1	100	15	8.38	3.119969
D. villosus	no predator	0.6264	1	100	16	8.36	7.921067
D. villosus	no predator	0.6264	1	100	17	8.33	5.738804
D. villosus	no predator	0.6264	1	100	18	8.31	3.839964
D. villosus	no predator	0.6264	1	100	19	8.29	7.399932
D. villosus	no predator	0.6264	1	100	20	8.27	3.841342
D. villosus	no predator	0.6264	1	100	21	8.24	8.001422
D. villosus	no predator	0.6264	1	100	22	8.2	11.6445
D. villosus	no predator	0.6264	1	100	23	8.19	6.473996
D. villosus	no predator	0.6264	1	100	24	8.15	6.363236

Gammarid species	treatment	weight [g]	pre-exposure time	trial	time [min.]	oxygen level [mg/l]	activity
D. villosus	no predator	0.6264	1	100	25	8.13	9.015052
D. villosus	no predator	0.6264	1	100	26	8.11	10.14687
D. villosus	no predator	0.6264	1	100	27	8.08	9.656547
D. villosus	no predator	0.6264	1	100	28	8.06	5.658204
D. villosus	no predator	0.6264	1	100	29	8.05	7.798065
D. villosus	no predator	0.6264	1	100	30	8.04	8.189819
D. villosus	no predator	0.4409	1	101	1	8.62	15.65931
D. villosus	no predator	0.4409	1	101	2	8.6	11.98048
D. villosus	no predator	0.4409	1	101	3	8.58	13.03964
D. villosus	no predator	0.4409	1	101	4	8.56	4.699594
D. villosus	no predator	0.4409	1	101	5	8.55	2.920931
D. villosus	no predator	0.4409	1	101	6	8.54	4.39953
D. villosus	no predator	0.4409	1	101	7	8.52	1.460045
D. villosus	no predator	0.4409	1	101	8	8.52	1.479386
D. villosus	no predator	0.4409	1	101	9	8.5	2.699973
D. villosus	no predator	0.4409	1	101	10	8.49	1.339986
D. villosus	no predator	0.4409	1	101	11	8.47	12.21988
D. villosus	no predator	0.4409	1	101	12	8.46	1.559985
D. villosus	no predator	0.4409	1	101	13	8.44	3.880916
D. villosus	no predator	0.4409	1	101	14	8.42	9.579909
D. villosus	no predator	0.4409	1	101	15	8.42	7.702078
D. villosus	no predator	0.4409	1	101	16	8.42	7.161188
D. villosus	no predator	0.4409	1	101	17	8.4	7.156508
D. villosus	no predator	0.4409	1	101	18	8.39	6.601199
D. villosus	no predator	0.4409	1	101	19	8.34	8.498651
D. villosus	no predator	0.4409	1	101	20	8.31	11.80266
D. villosus	no predator	0.4409	1	101	21	8.3	8.778586
D. villosus	no predator	0.4409	1	101	22	8.29	5.27851
D. villosus	no predator	0.4409	1	101	23	8.29	10.12302
D. villosus	no predator	0.4409	1	101	24	8.29	7.41999
D. villosus	no predator	0.4409	1	101	25	8.29	9.479971
D. villosus	no predator	0.4409	1	101	26	8.27	5.481752
D. villosus	no predator	0.4409	1	101	27	8.27	7.661849
D. villosus	no predator	0.4409	1	101	28	8.27	9.158231
D. villosus	no predator	0.4409	1	101	29	8.26	11.23808
D. villosus	no predator	0.4409	1	101	30	8.25	7.319922
D. villosus	no predator	0.5286	1	102	1	8.08	7.720533
D. villosus	no predator	0.5286	1	102	2	8.13	5.738984
D. villosus	no predator	0.5286	1	102	3	8.17	4.700678
D. villosus	no predator	0.5286	1	102	4	8.16	11.59958
D. villosus	no predator	0.5286	1	102	5	8.16	6.381858
D. villosus	no predator	0.5286	1	102	6	8.18	8.658001
D. villosus	no predator	0.5286	1	102	7	8.19	9.422313
D. villosus	no predator	0.5286	1	102	8	8.22	10.9588
D. villosus	no predator	0.5286	1	102	9	8.23	10.47859
D. villosus	no predator	0.5286	1	102	10	8.24	13.86066
D. villosus	no predator	0.5286	1	102	11	8.24	7.039137
D. villosus	no predator	0.5286	1	102	12	8.22	5.280848

Gammarid species	treatment	weight [g]	pre-exposure time	trial	time [min.]	oxygen level [mg/l]	activity
D. villosus	no predator	0.5286	1	102	13	8.1	6.479041
D. villosus	no predator	0.5286	1	102	14	8.02	4.699949
D. villosus	no predator	0.5286	1	102	15	7.96	4.939961
D. villosus	no predator	0.5286	1	102	16	7.93	6.465629
D. villosus	no predator	0.5286	1	102	17	7.92	10.15421
D. villosus	no predator	0.5286	1	102	18	7.98	10.93989
D. villosus	no predator	0.5286	1	102	19	8.02	6.021259
D. villosus	no predator	0.5286	1	102	20	8.06	6.764131
D. villosus	no predator	0.5286	1	102	21	8.07	6.798669
D. villosus	no predator	0.5286	1	102	22	8.08	7.857035
D. villosus	no predator	0.5286	1	102	23	8.09	3.719962
D. villosus	no predator	0.5286	1	102	24	8.12	5.341572
D. villosus	no predator	0.5286	1	102	25	8.13	3.898342
D. villosus	no predator	0.5286	1	102	26	8.13	5.339951
D. villosus	no predator	0.5286	1	102	27	8.13	8.961719
D. villosus	no predator	0.5286	1	102	28	8.13	5.698146
D. villosus	no predator	0.5286	1	102	29	8.14	7.379925
D. villosus	no predator	0.5286	1	102	30	8.12	2.319978
D. villosus	perch	0.9554	1	109	1	8.62	7.419926
D. villosus	perch	0.9554	1	109	2	8.6	15.52074
D. villosus	perch	0.9554	1	109	3	8.58	10.08152
D. villosus	perch	0.9554	1	109	4	8.57	21.65972
D. villosus	perch	0.9554	1	109	5	8.55	13.83914
D. villosus	perch	0.9554	1	109	6	8.54	14.71891
D. villosus	perch	0.9554	1	109	7	8.5	11.2223
D. villosus	perch	0.9554	1	109	8	8.49	16.35941
D. villosus	perch	0.9554	1	109	9	8.48	15.47787
D. villosus	perch	0.9554	1	109	10	8.47	13.72377
D. villosus	perch	0.9554	1	109	11	8.46	10.53768
D. villosus	perch	0.9554	1	109	12	8.44	8.81997
D. villosus	perch	0.9554	1	109	13	8.41	12.39988
D. villosus	perch	0.9554	1	109	14	8.39	11.92194
D. villosus	perch	0.9554	1	109	15	8.37	13.17987
D. villosus	perch	0.9554	1	109	16	8.35	16.28096
D. villosus	perch	0.9554	1	109	17	8.33	12.49871
D. villosus	perch	0.9554	1	109	18	8.31	12.54238
D. villosus	perch	0.9554	1	109	19	8.25	10.61868
D. villosus	perch	0.9554	1	109	20	8.17	10.53858
D. villosus	perch	0.9554	1	109	21	8.17	7.139922
D. villosus	perch	0.9554	1	109	22	8.15	10.18289
D. villosus	perch	0.9554	1	109	23	8	15.90145
D. villosus	perch	0.9554	1	109	24	7.71	8.476801
D. villosus	perch	0.9554	1	109	25	7.88	8.521595
D. villosus	perch	0.9554	1	109	26	7.95	5.698265
D. villosus	perch	0.9554	1	109	27	7.99	10.1453
D. villosus	perch	0.9554	1	109	28	7.98	11.63448
D. villosus	perch	0.9554	1	109	29	8.01	13.06756
D. villosus	perch	0.9554	1	109	30	8.01	8.63817

Gammarid species	treatment	weight [g]	pre-exposure time	trial	time [min.]	oxygen level [mg/l]	activity
D. villosus	perch	0.8284	1	110	1	8.55	7.319924
D. villosus	perch	0.8284	1	110	2	8.49	9.560804
D. villosus	perch	0.8284	1	110	3	8.45	2.479376
D. villosus	perch	0.8284	1	110	4	8.08	9.601172
D. villosus	perch	0.8284	1	110	5	7.92	6.378684
D. villosus	perch	0.8284	1	110	6	7.9	4.660498
D. villosus	perch	0.8284	1	110	7	7.9	6.96053
D. villosus	perch	0.8284	1	110	8	7.99	5.179947
D. villosus	perch	0.8284	1	110	9	7.99	9.40063
D. villosus	perch	0.8284	1	110	10	8.13	5.619226
D. villosus	perch	0.8284	1	110	11	8.18	7.760769
D. villosus	perch	0.8284	1	110	12	8.26	2.579976
D. villosus	perch	0.8284	1	110	13	8.28	4.979955
D. villosus	perch	0.8284	1	110	14	8.27	3.179968
D. villosus	perch	0.8284	1	110	15	8.29	8.220993
D. villosus	perch	0.8284	1	110	16	8.29	8.139983
D. villosus	perch	0.8284	1	110	17	8.28	8.461116
D. villosus	perch	0.8284	1	110	18	8.27	2.619974
D. villosus	perch	0.8284	1	110	19	8.25	6.301253
D. villosus	perch	0.8284	1	110	20	8.24	8.321355
D. villosus	perch	0.8284	1	110	21	8.21	7.984307
D. villosus	perch	0.8284	1	110	22	8.21	6.623184
D. villosus	perch	0.8284	1	110	23	8.2	13.13244
D. villosus	perch	0.8284	1	110	24	8.19	14.52153
D. villosus	perch	0.8284	1	110	25	8.17	6.201733
D. villosus	perch	0.8284	1	110	26	8.17	9.880083
D. villosus	perch	0.8284	1	110	27	8.13	6.660054
D. villosus	perch	0.8284	1	110	28	8.13	7.476326
D. villosus	perch	0.8284	1	110	29	8.11	10.0799
D. villosus	perch	0.8284	1	110	30	8.1	7.021907
D. villosus	perch	0.7814	1	111	1	8.68	4.759954
D. villosus	perch	0.7814	1	111	2	8.59	4.221158
D. villosus	perch	0.7814	1	111	3	8.29	3.679064
D. villosus	perch	0.7814	1	111	4	7.07	1.679984
D. villosus	perch	0.7814	1	111	5	6.23	5.28091
D. villosus	perch	0.7814	1	111	6	6.8	4.259002
D. villosus	perch	0.7814	1	111	7	6.99	3.379962
D. villosus	perch	0.7814	1	111	8	7.17	4.560614
D. villosus	perch	0.7814	1	111	9	7.41	2.499319
D. villosus	perch	0.7814	1	111	10	7.34	3.941521
D. villosus	perch	0.7814	1	111	11	7.41	6.238381
D. villosus	perch	0.7814	1	111	12	7.71	3.10357
D. villosus	perch	0.7814	1	111	13	7.59	9.240149
D. villosus	perch	0.7814	1	111	14	7.56	7.25711
D. villosus	perch	0.7814	1	111	15	7.15	8.098905
D. villosus	perch	0.7814	1	111	16	7.14	3.762242
D. villosus	perch	0.7814	1	111	17	7.42	4.498875
D. villosus	perch	0.7814	1	111	18	7.38	4.520013

Gammarid species	treatment	weight [g]	pre-exposure time	trial	time [min.]	oxygen level [mg/l]	activity
D. villosus	perch	0.7814	1	111	19	7.06	3.478709
D. villosus	perch	0.7814	1	111	20	7.01	3.739965
D. villosus	perch	0.7814	1	111	21	6.86	3.622846
D. villosus	perch	0.7814	1	111	22	6.81	2.559974
D. villosus	perch	0.7814	1	111	23	6.83	5.183066
D. villosus	perch	0.7814	1	111	24	7.08	5.56007
D. villosus	perch	0.7814	1	111	25	7.21	7.336681
D. villosus	perch	0.7814	1	111	26	6.89	2.521714
D. villosus	perch	0.7814	1	111	27	6.97	8.981785
D. villosus	perch	0.7814	1	111	28	6.98	4.94007
D. villosus	perch	0.7814	1	111	29	6.55	3.13817
D. villosus	perch	0.7814	1	111	30	6.21	9.011864
D. villosus	perch	0.7821	1	112	1	8.67	7.079988
D. villosus	perch	0.7821	1	112	2	8.63	13.34084
D. villosus	perch	0.7821	1	112	3	8.62	11.29905
D. villosus	perch	0.7821	1	112	4	8.6	13.97992
D. villosus	perch	0.7821	1	112	5	8.58	11.39947
D. villosus	perch	0.7821	1	112	6	8.49	7.059933
D. villosus	perch	0.7821	1	112	7	8.2	5.680545
D. villosus	perch	0.7821	1	112	8	8.2	11.21988
D. villosus	perch	0.7821	1	112	9	8.26	13.24131
D. villosus	perch	0.7821	1	112	10	8.26	9.182372
D. villosus	perch	0.7821	1	112	11	8.3	16.09594
D. villosus	perch	0.7821	1	112	12	8.29	11.38259
D. villosus	perch	0.7821	1	112	13	8.28	16.36476
D. villosus	perch	0.7821	1	112	14	8.31	16.71714
D. villosus	perch	0.7821	1	112	15	8.29	11.73899
D. villosus	perch	0.7821	1	112	16	8.26	12.16343
D. villosus	perch	0.7821	1	112	17	8.25	13.79416
D. villosus	perch	0.7821	1	112	18	8.24	19.74359
D. villosus	perch	0.7821	1	112	19	8.23	11.25611
D. villosus	perch	0.7821	1	112	20	8.21	13.66538
D. villosus	perch	0.7821	1	112	21	8.19	16.00145
D. villosus	perch	0.7821	1	112	22	8.17	18.1215
D. villosus	perch	0.7821	1	112	23	8.1	12.63694
D. villosus	perch	0.7821	1	112	24	8.1	17.41828
D. villosus	perch	0.7821	1	112	25	8.07	12.1049
D. villosus	perch	0.7821	1	112	26	8.05	15.2948
D. villosus	perch	0.7821	1	112	27	7.96	16.34166
D. villosus	perch	0.7821	1	112	28	7.97	15.78549
D. villosus	perch	0.7821	1	112	29	7.96	17.5343
D. villosus	perch	0.7821	1	112	30	7.95	17.10378
D. villosus	perch	0.5806	1	113	1	8.66	2.220218
D. villosus	perch	0.5806	1	113	2	8.62	1.88094
D. villosus	perch	0.5806	1	113	3	8.59	3.05907
D. villosus	perch	0.5806	1	113	4	8.6	2.761232
D. villosus	perch	0.5806	1	113	5	8.59	3.219187
D. villosus	perch	0.5806	1	113	6	8.57	4.759473

Gammarid species	treatment	weight [g]	pre-exposure time	trial	time [min.]	oxygen level [mg/l]	activity
D. villosus	perch	0.5806	1	113	7	8.56	3.660563
D. villosus	perch	0.5806	1	113	8	8.54	4.020023
D. villosus	perch	0.5806	1	113	9	8.51	6.560717
D. villosus	perch	0.5806	1	113	10	8.51	4.259297
D. villosus	perch	0.5806	1	113	11	8.48	4.521696
D. villosus	perch	0.5806	1	113	12	8.47	5.37833
D. villosus	perch	0.5806	1	113	13	8.46	6.399029
D. villosus	perch	0.5806	1	113	14	8.43	3.402006
D. villosus	perch	0.5806	1	113	15	8.44	4.658936
D. villosus	perch	0.5806	1	113	16	8.44	1.819981
D. villosus	perch	0.5806	1	113	17	8.42	4.399959
D. villosus	perch	0.5806	1	113	18	8.41	6.539934
D. villosus	perch	0.5806	1	113	19	8.37	4.919956
D. villosus	perch	0.5806	1	113	20	8.39	5.739941
D. villosus	perch	0.5806	1	113	21	8.38	4.682833
D. villosus	perch	0.5806	1	113	22	8.35	3.697084
D. villosus	perch	0.5806	1	113	23	8.33	4.163079
D. villosus	perch	0.5806	1	113	24	8.34	8.000051
D. villosus	perch	0.5806	1	113	25	8.31	7.258373
D. villosus	perch	0.5806	1	113	26	8.29	4.805173
D. villosus	perch	0.5806	1	113	27	8.26	5.720126
D. villosus	perch	0.5806	1	113	28	8.24	8.980095
D. villosus	perch	0.5806	1	113	29	8.23	6.903948
D. villosus	perch	0.5806	1	113	30	8.22	9.396257
D. villosus	perch	0.6616	1	114	1	8.33	17.09983
D. villosus	perch	0.6616	1	114	2	8.37	5.899945
D. villosus	perch	0.6616	1	114	3	8.31	1.880701
D. villosus	perch	0.6616	1	114	4	8.16	3.999239
D. villosus	perch	0.6616	1	114	5	8.12	2.719974
D. villosus	perch	0.6616	1	114	6	8.21	1.699983
D. villosus	perch	0.6616	1	114	7	8.28	0
D. villosus	perch	0.6616	1	114	8	8.31	1.639984
D. villosus	perch	0.6616	1	114	9	8.36	0.179998
D. villosus	perch	0.6616	1	114	10	8.39	0.399995
D. villosus	perch	0.6616	1	114	11	8.4	0.499995
D. villosus	perch	0.6616	1	114	12	8.43	1.459984
D. villosus	perch	0.6616	1	114	13	8.43	0.339996
D. villosus	perch	0.6616	1	114	14	8.44	0.619993
D. villosus	perch	0.6616	1	114	15	8.46	0.399996
D. villosus	perch	0.6616	1	114	16	8.47	0.199998
D. villosus	perch	0.6616	1	114	17	8.47	0.04
D. villosus	perch	0.6616	1	114	18	8.48	0.359996
D. villosus	perch	0.6616	1	114	19	8.47	1.09999
D. villosus	perch	0.6616	1	114	20	8.46	0.459995
D. villosus	perch	0.6616	1	114	21	8.43	0
D. villosus	perch	0.6616	1	114	22	8.43	0.361496
D. villosus	perch	0.6616	1	114	23	8.43	0.058499
D. villosus	perch	0.6616	1	114	24	8.43	0.259998

Gammarid species	treatment	weight [g]	pre-exposure time	trial	time [min.]	oxygen level [mg/l]	activity
D. villosus	perch	0.6616	1	114	25	8.42	0.179998
D. villosus	perch	0.6616	1	114	26	8.43	0.979989
D. villosus	perch	0.6616	1	114	27	8.44	0.759992
D. villosus	perch	0.6616	1	114	28	8.44	0.399996
D. villosus	perch	0.6616	1	114	29	8.43	0.179999
D. villosus	perch	0.6616	1	114	30	8.43	0
D. villosus	perch	0.5127	1	115	1	6.35	4.77995
D. villosus	perch	0.5127	1	115	2	6.26	9.819903
D. villosus	perch	0.5127	1	115	3	6.17	6.759943
D. villosus	perch	0.5127	1	115	4	6.12	4.480795
D. villosus	perch	0.5127	1	115	5	6.07	3.480087
D. villosus	perch	0.5127	1	115	6	6.01	10.19894
D. villosus	perch	0.5127	1	115	7	5.98	6.679934
D. villosus	perch	0.5127	1	115	8	5.95	6.099935
D. villosus	perch	0.5127	1	115	9	5.92	8.56063
D. villosus	perch	0.5127	1	115	10	5.9	9.839197
D. villosus	perch	0.5127	1	115	11	5.88	6.919938
D. villosus	perch	0.5127	1	115	12	5.86	9.922607
D. villosus	perch	0.5127	1	115	13	5.83	12.37814
D. villosus	perch	0.5127	1	115	14	5.31	12.06096
D. villosus	perch	0.5127	1	115	15	4.97	7.197893
D. villosus	perch	0.5127	1	115	16	4.66	8.279917
D. villosus	perch	0.5127	1	115	17	4.13	5.602345
D. villosus	perch	0.5127	1	115	18	3.66	5.677543
D. villosus	perch	0.5127	1	115	19	3.45	13.66383
D. villosus	perch	0.5127	1	115	20	3.47	4.715992
D. villosus	perch	0.5127	1	115	21	3.82	7.764241
D. villosus	perch	0.5127	1	115	22	4.08	9.737081
D. villosus	perch	0.5127	1	115	23	4.39	7.163107
D. villosus	perch	0.5127	1	115	24	4.46	9.920027
D. villosus	perch	0.5127	1	115	25	4.58	6.781673
D. villosus	perch	0.5127	1	115	26	4.83	11.35826
D. villosus	perch	0.5127	1	115	27	5.05	7.499928
D. villosus	perch	0.5127	1	115	28	5.06	6.899938
D. villosus	perch	0.5127	1	115	29	5.05	12.22948
D. villosus	perch	0.5127	1	115	30	5.02	9.652232
D. villosus	perch	0.7549	1	116	1	8.31	1.279987
D. villosus	perch	0.7549	1	116	2	8.32	0.099999
D. villosus	perch	0.7549	1	116	3	8.29	1.179988
D. villosus	perch	0.7549	1	116	4	8.29	0.219998
D. villosus	perch	0.7549	1	116	5	8.28	1.159988
D. villosus	perch	0.7549	1	116	6	8.3	1.239988
D. villosus	perch	0.7549	1	116	7	8.28	0.499995
D. villosus	perch	0.7549	1	116	8	8.29	1.039989
D. villosus	perch	0.7549	1	116	9	8.26	0.479995
D. villosus	perch	0.7549	1	116	10	8.27	0.519995
D. villosus	perch	0.7549	1	116	11	8.26	0.499994
D. villosus	perch	0.7549	1	116	12	8.27	0.659994

Gammarid species	treatment	weight [g]	pre-exposure time	trial	time [min.]	oxygen level [mg/l]	activity
D. villosus	perch	0.7549	1	116	13	8.25	0.239998
D. villosus	perch	0.7549	1	116	14	8.25	0.879991
D. villosus	perch	0.7549	1	116	15	8.23	0.93999
D. villosus	perch	0.7549	1	116	16	8.24	1.479985
D. villosus	perch	0.7549	1	116	17	8.25	1.761182
D. villosus	perch	0.7549	1	116	18	8.25	1.07879
D. villosus	perch	0.7549	1	116	19	8.2	3.561287
D. villosus	perch	0.7549	1	116	20	8.18	2.718653
D. villosus	perch	0.7549	1	116	21	8.13	0.661434
D. villosus	perch	0.7549	1	116	22	8.11	1.881482
D. villosus	perch	0.7549	1	116	23	8.09	2.978471
D. villosus	perch	0.7549	1	116	24	8.07	0.781612
D. villosus	perch	0.7549	1	116	25	8.07	0.699992
D. villosus	perch	0.7549	1	116	26	8.04	2.699974
D. villosus	perch	0.7549	1	116	27	8.03	5.181748
D. villosus	perch	0.7549	1	116	28	8.01	1.158188
D. villosus	perch	0.7549	1	116	29	7.99	1.579984
D. villosus	perch	0.7549	1	116	30	7.96	1.399987
D. villosus	perch	0.4717	1	117	1	8.28	0.239998
D. villosus	perch	0.4717	1	117	2	8.25	0.300297
D. villosus	perch	0.4717	1	117	3	8.25	0.239698
D. villosus	perch	0.4717	1	117	4	8.22	1.619984
D. villosus	perch	0.4717	1	117	5	8.2	0.159999
D. villosus	perch	0.4717	1	117	6	8.18	0.820532
D. villosus	perch	0.4717	1	117	7	8.16	0.199998
D. villosus	perch	0.4717	1	117	8	8.15	0.259997
D. villosus	perch	0.4717	1	117	9	8.14	0.119999
D. villosus	perch	0.4717	1	117	10	8.13	0.199998
D. villosus	perch	0.4717	1	117	11	8.11	0.02
D. villosus	perch	0.4717	1	117	12	8.1	0.499994
D. villosus	perch	0.4717	1	117	13	8.1	0
D. villosus	perch	0.4717	1	117	14	8.1	0.02
D. villosus	perch	0.4717	1	117	15	8.09	0.079999
D. villosus	perch	0.4717	1	117	16	8.09	1.01999
D. villosus	perch	0.4717	1	117	17	8.08	2.319974
D. villosus	perch	0.4717	1	117	18	8.06	0.159998
D. villosus	perch	0.4717	1	117	19	8.07	0.299997
D. villosus	perch	0.4717	1	117	20	8.07	0.079999
D. villosus	perch	0.4717	1	117	21	8.06	0.659994
D. villosus	perch	0.4717	1	117	22	8.07	0.04
D. villosus	perch	0.4717	1	117	23	8.05	0
D. villosus	perch	0.4717	1	117	24	8.04	0.439995
D. villosus	perch	0.4717	1	117	25	8.03	0.02
D. villosus	perch	0.4717	1	117	26	8.02	0.319996
D. villosus	perch	0.4717	1	117	27	8.03	5.319948
D. villosus	perch	0.4717	1	117	28	8.02	2.93997
D. villosus	perch	0.4717	1	117	29	8	0.359997
D. villosus	perch	0.4717	1	117	30	8.01	1.159988

Gammarid species	treatment	weight [g]	pre-exposure time	trial	time [min.]	oxygen level [mg/l]	activity
D. villosus	perch	0.5513	1	118	1	8.43	3.499971
D. villosus	perch	0.5513	1	118	2	8.41	5.479948
D. villosus	perch	0.5513	1	118	3	8.38	2.981051
D. villosus	perch	0.5513	1	118	4	8.33	2.619314
D. villosus	perch	0.5513	1	118	5	8.23	1.600464
D. villosus	perch	0.5513	1	118	6	8.19	3.139972
D. villosus	perch	0.5513	1	118	7	8.14	0.95999
D. villosus	perch	0.5513	1	118	8	8.09	1.079989
D. villosus	perch	0.5513	1	118	9	8.03	0.579994
D. villosus	perch	0.5513	1	118	10	7.97	0.739993
D. villosus	perch	0.5513	1	118	11	7.92	3.379966
D. villosus	perch	0.5513	1	118	12	7.92	5.359951
D. villosus	perch	0.5513	1	118	13	7.97	4.184758
D. villosus	perch	0.5513	1	118	14	8	8.195115
D. villosus	perch	0.5513	1	118	15	8.02	3.721044
D. villosus	perch	0.5513	1	118	16	8.02	4.01888
D. villosus	perch	0.5513	1	118	17	8	2.601174
D. villosus	perch	0.5513	1	118	18	8.01	1.198788
D. villosus	perch	0.5513	1	118	19	8	3.279969
D. villosus	perch	0.5513	1	118	20	8.01	4.321338
D. villosus	perch	0.5513	1	118	21	8.01	4.680013
D. villosus	perch	0.5513	1	118	22	7.99	3.523025
D. villosus	perch	0.5513	1	118	23	8.03	7.898485
D. villosus	perch	0.5513	1	118	24	8.05	4.0184
D. villosus	perch	0.5513	1	118	25	8.01	2.919975
D. villosus	perch	0.5513	1	118	26	7.96	0.779992
D. villosus	perch	0.5513	1	118	27	7.95	1.319987
D. villosus	perch	0.5513	1	118	28	7.96	6.305521
D. villosus	perch	0.5513	1	118	29	7.96	9.854321
D. villosus	perch	0.5513	1	118	30	7.96	4.96589
D. villosus	perch	0.5346	1	119	1	8.22	5.339768
D. villosus	perch	0.5346	1	119	2	8.18	1.89998
D. villosus	perch	0.5346	1	119	3	8.16	5.199952
D. villosus	perch	0.5346	1	119	4	8.14	5.319941
D. villosus	perch	0.5346	1	119	5	8.11	3.140449
D. villosus	perch	0.5346	1	119	6	8.1	4.920495
D. villosus	perch	0.5346	1	119	7	8.08	3.480028
D. villosus	perch	0.5346	1	119	8	8.05	5.780663
D. villosus	perch	0.5346	1	119	9	8.03	1.699383
D. villosus	perch	0.5346	1	119	10	7.99	4.762353
D. villosus	perch	0.5346	1	119	11	7.97	6.518498
D. villosus	perch	0.5346	1	119	12	7.95	4.780076
D. villosus	perch	0.5346	1	119	13	7.95	6.759099
D. villosus	perch	0.5346	1	119	14	7.93	3.740023
D. villosus	perch	0.5346	1	119	15	7.92	6.398925
D. villosus	perch	0.5346	1	119	16	7.89	3.541105
D. villosus	perch	0.5346	1	119	17	7.9	4.678813
D. villosus	perch	0.5346	1	119	18	7.9	5.663724

Gammarid species	treatment	weight [g]	pre-exposure time	trial	time [min.]	oxygen level [mg/l]	activity
D. villosus	perch	0.5346	1	119	19	7.87	3.016191
D. villosus	perch	0.5346	1	119	20	7.78	6.179939
D. villosus	perch	0.5346	1	119	21	7.68	0.739992
D. villosus	perch	0.5346	1	119	22	7.63	4.559956
D. villosus	perch	0.5346	1	119	23	7.6	3.559967
D. villosus	perch	0.5346	1	119	24	7.58	0.94323
D. villosus	perch	0.5346	1	119	25	7.57	6.558377
D. villosus	perch	0.5346	1	119	26	7.65	1.8383
D. villosus	perch	0.5346	1	119	27	7.66	2.17998
D. villosus	perch	0.5346	1	119	28	7.64	3.339967
D. villosus	perch	0.5346	1	119	29	7.64	7.663757
D. villosus	perch	0.5346	1	119	30	7.63	2.356137
D. villosus	perch	0.5506	1	120	1	8.16	3.859961
D. villosus	perch	0.5506	1	120	2	8.13	8.45992
D. villosus	perch	0.5506	1	120	3	8.11	1.740703
D. villosus	perch	0.5506	1	120	4	8.07	0.499695
D. villosus	perch	0.5506	1	120	5	8.06	1.819562
D. villosus	perch	0.5506	1	120	6	8.05	1.399985
D. villosus	perch	0.5506	1	120	7	8.02	1.619983
D. villosus	perch	0.5506	1	120	8	8.01	0.939991
D. villosus	perch	0.5506	1	120	9	8	0.719992
D. villosus	perch	0.5506	1	120	10	7.98	1.259987
D. villosus	perch	0.5506	1	120	11	7.97	2.500815
D. villosus	perch	0.5506	1	120	12	7.96	1.919142
D. villosus	perch	0.5506	1	120	13	7.94	0.359997
D. villosus	perch	0.5506	1	120	14	7.93	2.859972
D. villosus	perch	0.5506	1	120	15	7.91	2.139978
D. villosus	perch	0.5506	1	120	16	7.89	0.299997
D. villosus	perch	0.5506	1	120	17	7.86	1.359987
D. villosus	perch	0.5506	1	120	18	7.84	1.401246
D. villosus	perch	0.5506	1	120	19	7.84	4.95995
D. villosus	perch	0.5506	1	120	20	7.81	3.139968
D. villosus	perch	0.5506	1	120	21	7.8	2.519974
D. villosus	perch	0.5506	1	120	22	7.78	0.459996
D. villosus	perch	0.5506	1	120	23	7.76	0.279997
D. villosus	perch	0.5506	1	120	24	7.75	0.261617
D. villosus	perch	0.5506	1	120	25	7.76	0.238378
D. villosus	perch	0.5506	1	120	26	7.75	0.099999
D. villosus	perch	0.5506	1	120	27	7.74	1.579984
D. villosus	perch	0.5506	1	120	28	7.71	1.459986
D. villosus	perch	0.5506	1	120	29	7.7	0.479995
D. villosus	perch	0.5506	1	120	30	7.69	0.359996
G. jazdzewski	no predator	0.2399	1	129	1	8.92	10.08093
G. jazdzewski	no predator	0.2399	1	129	2	8.9	15.73955
G. jazdzewski	no predator	0.2399	1	129	3	8.89	9.780074
G. jazdzewski	no predator	0.2399	1	129	4	8.89	16.06127
G. jazdzewski	no predator	0.2399	1	129	5	8.88	16.8378
G. jazdzewski	no predator	0.2399	1	129	6	8.87	12.66103

Gammarid species	treatment	weight [g]	pre-exposure time	trial	time [min.]	oxygen level [mg/l]	activity
G. jazdzewski	no predator	0.2399	1	129	7	8.88	15.08002
G. jazdzewski	no predator	0.2399	1	129	8	8.89	21.33997
G. jazdzewski	no predator	0.2399	1	129	9	8.88	23.14139
G. jazdzewski	no predator	0.2399	1	129	10	8.87	21.58008
G. jazdzewski	no predator	0.2399	1	129	11	8.86	19.87842
G. jazdzewski	no predator	0.2399	1	129	12	8.88	16.88
G. jazdzewski	no predator	0.2399	1	129	13	8.86	25.42088
G. jazdzewski	no predator	0.2399	1	129	14	8.85	17.97899
G. jazdzewski	no predator	0.2399	1	129	15	8.85	15.66428
G. jazdzewski	no predator	0.2399	1	129	16	8.83	22.39444
G. jazdzewski	no predator	0.2399	1	129	17	8.81	24.01982
G. jazdzewski	no predator	0.2399	1	129	18	8.8	22.1423
G. jazdzewski	no predator	0.2399	1	129	19	8.79	28.09983
G. jazdzewski	no predator	0.2399	1	129	20	8.79	21.5999
G. jazdzewski	no predator	0.2399	1	129	21	8.77	21.8799
G. jazdzewski	no predator	0.2399	1	129	22	8.76	19.74435
G. jazdzewski	no predator	0.2399	1	129	23	8.78	18.0385
G. jazdzewski	no predator	0.2399	1	129	24	8.76	20.27672
G. jazdzewski	no predator	0.2399	1	129	25	8.75	23.58655
G. jazdzewski	no predator	0.2399	1	129	26	8.74	27.40004
G. jazdzewski	no predator	0.2399	1	129	27	8.73	24.83467
G. jazdzewski	no predator	0.2399	1	129	28	8.72	19.42172
G. jazdzewski	no predator	0.2399	1	129	29	8.71	20.67992
G. jazdzewski	no predator	0.2399	1	129	30	8.71	22.92583
G. jazdzewski	no predator	0.249	1	130	1	8.8	23.64001
G. jazdzewski	no predator	0.249	1	130	2	8.81	22.11954
G. jazdzewski	no predator	0.249	1	130	3	8.82	20.4604
G. jazdzewski	no predator	0.249	1	130	4	8.84	26.39963
G. jazdzewski	no predator	0.249	1	130	5	8.85	23.27904
G. jazdzewski	no predator	0.249	1	130	6	8.84	28.82246
G. jazdzewski	no predator	0.249	1	130	7	8.84	29.77826
G. jazdzewski	no predator	0.249	1	130	8	8.83	29.37922
G. jazdzewski	no predator	0.249	1	130	9	8.82	21.64201
G. jazdzewski	no predator	0.249	1	130	10	8.82	24.37922
G. jazdzewski	no predator	0.249	1	130	11	8.83	26.33991
G. jazdzewski	no predator	0.249	1	130	12	8.84	24.09904
G. jazdzewski	no predator	0.249	1	130	13	8.82	21.91989
G. jazdzewski	no predator	0.249	1	130	14	8.81	22.62398
G. jazdzewski	no predator	0.249	1	130	15	8.8	25.99901
G. jazdzewski	no predator	0.249	1	130	16	8.79	26.90345
G. jazdzewski	no predator	0.249	1	130	17	8.8	29.57898
G. jazdzewski	no predator	0.249	1	130	18	8.78	21.81768
G. jazdzewski	no predator	0.249	1	130	19	8.77	14.75751
G. jazdzewski	no predator	0.249	1	130	20	8.76	22.47996
G. jazdzewski	no predator	0.249	1	130	21	8.76	19.65992
G. jazdzewski	no predator	0.249	1	130	22	8.76	21.73841
G. jazdzewski	no predator	0.249	1	130	23	8.74	19.8783
G. jazdzewski	no predator	0.249	1	130	24	8.74	16.1047

Gammarid species	treatment	weight [g]	pre-exposure time	trial	time [min.]	oxygen level [mg/l]	activity
G. jazdzewski	no predator	0.249	1	130	25	8.74	18.06835
G. jazdzewski	no predator	0.249	1	130	26	8.72	18.37663
G. jazdzewski	no predator	0.249	1	130	27	8.71	16.96001
G. jazdzewski	no predator	0.249	1	130	28	8.72	13.58004
G. jazdzewski	no predator	0.249	1	130	29	8.71	14.18388
G. jazdzewski	no predator	0.249	1	130	30	8.7	11.55815
G. jazdzewski	no predator	0.2555	1	131	1	8.85	23.38026
G. jazdzewski	no predator	0.2555	1	131	2	8.85	32.05996
G. jazdzewski	no predator	0.2555	1	131	3	8.84	27.61967
G. jazdzewski	no predator	0.2555	1	131	4	8.83	20.22003
G. jazdzewski	no predator	0.2555	1	131	5	8.82	31.75993
G. jazdzewski	no predator	0.2555	1	131	6	8.82	28.51941
G. jazdzewski	no predator	0.2555	1	131	7	8.82	32.55926
G. jazdzewski	no predator	0.2555	1	131	8	8.8	38.30173
G. jazdzewski	no predator	0.2555	1	131	9	8.81	33.47925
G. jazdzewski	no predator	0.2555	1	131	10	8.81	28.38068
G. jazdzewski	no predator	0.2555	1	131	11	8.8	32.0033
G. jazdzewski	no predator	0.2555	1	131	12	8.8	31.19565
G. jazdzewski	no predator	0.2555	1	131	13	8.77	26.62088
G. jazdzewski	no predator	0.2555	1	131	14	8.77	29.84095
G. jazdzewski	no predator	0.2555	1	131	15	8.76	28.9021
G. jazdzewski	no predator	0.2555	1	131	16	8.75	31.76005
G. jazdzewski	no predator	0.2555	1	131	17	8.75	34.86235
G. jazdzewski	no predator	0.2555	1	131	18	8.74	29.68012
G. jazdzewski	no predator	0.2555	1	131	19	8.72	23.75357
G. jazdzewski	no predator	0.2555	1	131	20	8.72	18.43995
G. jazdzewski	no predator	0.2555	1	131	21	8.71	24.13983
G. jazdzewski	no predator	0.2555	1	131	22	8.7	28.60426
G. jazdzewski	no predator	0.2555	1	131	23	8.71	21.5216
G. jazdzewski	no predator	0.2555	1	131	24	8.69	23.71846
G. jazdzewski	no predator	0.2555	1	131	25	8.69	25.35662
G. jazdzewski	no predator	0.2555	1	131	26	8.68	20.59991
G. jazdzewski	no predator	0.2555	1	131	27	8.66	32.68879
G. jazdzewski	no predator	0.2555	1	131	28	8.66	30.07455
G. jazdzewski	no predator	0.2555	1	131	29	8.64	20.64196
G. jazdzewski	no predator	0.2555	1	131	30	8.64	26.49807
G. jazdzewski	no predator	0.3204	1	132	1	8.86	22.81984
G. jazdzewski	no predator	0.3204	1	132	2	8.86	23.20032
G. jazdzewski	no predator	0.3204	1	132	3	8.86	27.66039
G. jazdzewski	no predator	0.3204	1	132	4	8.85	31.12005
G. jazdzewski	no predator	0.3204	1	132	5	8.83	21.80008
G. jazdzewski	no predator	0.3204	1	132	6	8.82	26.2595
G. jazdzewski	no predator	0.3204	1	132	7	8.8	20.57942
G. jazdzewski	no predator	0.3204	1	132	8	8.81	24.02254
G. jazdzewski	no predator	0.3204	1	132	9	8.79	27.36069
G. jazdzewski	no predator	0.3204	1	132	10	8.79	21.96074
G. jazdzewski	no predator	0.3204	1	132	11	8.78	28.64164
G. jazdzewski	no predator	0.3204	1	132	12	8.76	23.84103

Gammarid species	treatment	weight [g]	pre-exposure time	trial	time [min.]	oxygen level [mg/l]	activity
G. jazdzewski	no predator	0.3204	1	132	13	8.75	25.79536
G. jazdzewski	no predator	0.3204	1	132	14	8.73	23.86292
G. jazdzewski	no predator	0.3204	1	132	15	8.72	18.33785
G. jazdzewski	no predator	0.3204	1	132	16	8.72	21.60442
G. jazdzewski	no predator	0.3204	1	132	17	8.7	18.7801
G. jazdzewski	no predator	0.3204	1	132	18	8.68	20.61632
G. jazdzewski	no predator	0.3204	1	132	19	8.69	20.11986
G. jazdzewski	no predator	0.3204	1	132	20	8.69	18.20264
G. jazdzewski	no predator	0.3204	1	132	21	8.68	24.59994
G. jazdzewski	no predator	0.3204	1	132	22	8.68	15.45998
G. jazdzewski	no predator	0.3204	1	132	23	8.66	24.78455
G. jazdzewski	no predator	0.3204	1	132	24	8.65	28.34483
G. jazdzewski	no predator	0.3204	1	132	25	8.64	25.93681
G. jazdzewski	no predator	0.3204	1	132	26	8.62	28.09656
G. jazdzewski	no predator	0.3204	1	132	27	8.61	27.89629
G. jazdzewski	no predator	0.3204	1	132	28	8.61	27.16352
G. jazdzewski	no predator	0.3204	1	132	29	8.6	32.18173
G. jazdzewski	no predator	0.3204	1	132	30	8.6	22.22781
G. jazdzewski	no predator	0.2432	1	133	1	8.75	18.87974
G. jazdzewski	no predator	0.2432	1	133	2	8.76	11.44085
G. jazdzewski	no predator	0.2432	1	133	3	8.78	17.73971
G. jazdzewski	no predator	0.2432	1	133	4	8.77	20.66082
G. jazdzewski	no predator	0.2432	1	133	5	8.77	20.71955
G. jazdzewski	no predator	0.2432	1	133	6	8.77	12.34005
G. jazdzewski	no predator	0.2432	1	133	7	8.78	17.6582
G. jazdzewski	no predator	0.2432	1	133	8	8.77	11.88187
G. jazdzewski	no predator	0.2432	1	133	9	8.77	12.11862
G. jazdzewski	no predator	0.2432	1	133	10	8.76	11.08152
G. jazdzewski	no predator	0.2432	1	133	11	8.76	17.4591
G. jazdzewski	no predator	0.2432	1	133	12	8.75	9.299072
G. jazdzewski	no predator	0.2432	1	133	13	8.74	7.261843
G. jazdzewski	no predator	0.2432	1	133	14	8.74	4.841096
G. jazdzewski	no predator	0.2432	1	133	15	8.73	6.937955
G. jazdzewski	no predator	0.2432	1	133	16	8.74	7.078855
G. jazdzewski	no predator	0.2432	1	133	17	8.73	7.399932
G. jazdzewski	no predator	0.2432	1	133	18	8.74	8.523698
G. jazdzewski	no predator	0.2432	1	133	19	8.72	7.838785
G. jazdzewski	no predator	0.2432	1	133	20	8.72	7.961426
G. jazdzewski	no predator	0.2432	1	133	21	8.71	9.838571
G. jazdzewski	no predator	0.2432	1	133	22	8.7	8.739971
G. jazdzewski	no predator	0.2432	1	133	23	8.68	13.91993
G. jazdzewski	no predator	0.2432	1	133	24	8.69	8.524837
G. jazdzewski	no predator	0.2432	1	133	25	8.68	10.50008
G. jazdzewski	no predator	0.2432	1	133	26	8.69	13.27651
G. jazdzewski	no predator	0.2432	1	133	27	8.66	11.80528
G. jazdzewski	no predator	0.2432	1	133	28	8.66	17.91442
G. jazdzewski	no predator	0.2432	1	133	29	8.65	15.76177
G. jazdzewski	no predator	0.2432	1	133	30	8.65	17.68384

Gammarid species	treatment	weight [g]	pre-exposure time	trial	time [min.]	oxygen level [mg/l]	activity
G. jazdzewski	no predator	0.1963	1	134	1	8.76	31.74006
G. jazdzewski	no predator	0.1963	1	134	2	8.76	33.84045
G. jazdzewski	no predator	0.1963	1	134	3	8.76	39.23956
G. jazdzewski	no predator	0.1963	1	134	4	8.77	36.6207
G. jazdzewski	no predator	0.1963	1	134	5	8.75	31.02048
G. jazdzewski	no predator	0.1963	1	134	6	8.76	37.33946
G. jazdzewski	no predator	0.1963	1	134	7	8.74	28.95821
G. jazdzewski	no predator	0.1963	1	134	8	8.74	26.28316
G. jazdzewski	no predator	0.1963	1	134	9	8.74	34.74009
G. jazdzewski	no predator	0.1963	1	134	10	8.72	31.99931
G. jazdzewski	no predator	0.1963	1	134	11	8.73	28.35756
G. jazdzewski	no predator	0.1963	1	134	12	8.71	30.14438
G. jazdzewski	no predator	0.1963	1	134	13	8.68	28.55732
G. jazdzewski	no predator	0.1963	1	134	14	8.7	28.98193
G. jazdzewski	no predator	0.1963	1	134	15	8.7	37.18208
G. jazdzewski	no predator	0.1963	1	134	16	8.68	36.57777
G. jazdzewski	no predator	0.1963	1	134	17	8.67	26.34238
G. jazdzewski	no predator	0.1963	1	134	18	8.67	34.16127
G. jazdzewski	no predator	0.1963	1	134	19	8.66	26.8948
G. jazdzewski	no predator	0.1963	1	134	20	8.66	28.72811
G. jazdzewski	no predator	0.1963	1	134	21	8.65	32.68159
G. jazdzewski	no predator	0.1963	1	134	22	8.63	31.79722
G. jazdzewski	no predator	0.1963	1	134	23	8.63	32.02159
G. jazdzewski	no predator	0.1963	1	134	24	8.63	33.70329
G. jazdzewski	no predator	0.1963	1	134	25	8.61	25.69687
G. jazdzewski	no predator	0.1963	1	134	26	8.62	21.7784
G. jazdzewski	no predator	0.1963	1	134	27	8.61	26.46184
G. jazdzewski	no predator	0.1963	1	134	28	8.59	19.59272
G. jazdzewski	no predator	0.1963	1	134	29	8.59	9.700035
G. jazdzewski	no predator	0.1963	1	134	30	8.59	17.83795
G. jazdzewski	no predator	0.2605	1	135	1	8.91	17.35977
G. jazdzewski	no predator	0.2605	1	135	2	8.9	18.41965
G. jazdzewski	no predator	0.2605	1	135	3	8.9	26.52089
G. jazdzewski	no predator	0.2605	1	135	4	8.91	26.74165
G. jazdzewski	no predator	0.2605	1	135	5	8.9	27.75683
G. jazdzewski	no predator	0.2605	1	135	6	8.89	26.98195
G. jazdzewski	no predator	0.2605	1	135	7	8.9	20.57828
G. jazdzewski	no predator	0.2605	1	135	8	8.87	21.35925
G. jazdzewski	no predator	0.2605	1	135	9	8.87	12.7021
G. jazdzewski	no predator	0.2605	1	135	10	8.87	13.97931
G. jazdzewski	no predator	0.2605	1	135	11	8.86	16.0017
G. jazdzewski	no predator	0.2605	1	135	12	8.85	20.47739
G. jazdzewski	no predator	0.2605	1	135	13	8.85	24.40371
G. jazdzewski	no predator	0.2605	1	135	14	8.85	22.95803
G. jazdzewski	no predator	0.2605	1	135	15	8.84	27.602
G. jazdzewski	no predator	0.2605	1	135	16	8.82	29.18331
G. jazdzewski	no predator	0.2605	1	135	17	8.81	30.22247
G. jazdzewski	no predator	0.2605	1	135	18	8.8	34.93888

Gammarid species	treatment	weight [g]	pre-exposure time	trial	time [min.]	oxygen level [mg/l]	activity
G. jazdzewski	no predator	0.2605	1	135	19	8.8	32.55745
G. jazdzewski	no predator	0.2605	1	135	20	8.79	30.81999
G. jazdzewski	no predator	0.2605	1	135	21	8.77	37.41704
G. jazdzewski	no predator	0.2605	1	135	22	8.77	34.04285
G. jazdzewski	no predator	0.2605	1	135	23	8.76	31.96152
G. jazdzewski	no predator	0.2605	1	135	24	8.74	29.01358
G. jazdzewski	no predator	0.2605	1	135	25	8.73	29.54823
G. jazdzewski	no predator	0.2605	1	135	26	8.7	28.53313
G. jazdzewski	no predator	0.2605	1	135	27	8.72	26.96705
G. jazdzewski	no predator	0.2605	1	135	28	8.71	28.31636
G. jazdzewski	no predator	0.2605	1	135	29	8.71	31.298
G. jazdzewski	no predator	0.2605	1	135	30	8.69	29.89592
G. jazdzewski	no predator	0.2137	1	136	1	8.87	29.9397
G. jazdzewski	no predator	0.2137	1	136	2	8.86	23.02024
G. jazdzewski	no predator	0.2137	1	136	3	8.86	26.17991
G. jazdzewski	no predator	0.2137	1	136	4	8.84	26.7616
G. jazdzewski	no predator	0.2137	1	136	5	8.84	25.0197
G. jazdzewski	no predator	0.2137	1	136	6	8.86	24.71795
G. jazdzewski	no predator	0.2137	1	136	7	8.86	24.08168
G. jazdzewski	no predator	0.2137	1	136	8	8.88	17.33748
G. jazdzewski	no predator	0.2137	1	136	9	8.89	17.44277
G. jazdzewski	no predator	0.2137	1	136	10	8.88	20.98081
G. jazdzewski	no predator	0.2137	1	136	11	8.88	25.52089
G. jazdzewski	no predator	0.2137	1	136	12	8.86	21.08099
G. jazdzewski	no predator	0.2137	1	136	13	8.87	22.01625
G. jazdzewski	no predator	0.2137	1	136	14	8.88	13.84401
G. jazdzewski	no predator	0.2137	1	136	15	8.85	20.72111
G. jazdzewski	no predator	0.2137	1	136	16	8.86	18.87892
G. jazdzewski	no predator	0.2137	1	136	17	8.84	13.46245
G. jazdzewski	no predator	0.2137	1	136	18	8.82	17.07761
G. jazdzewski	no predator	0.2137	1	136	19	8.82	21.00393
G. jazdzewski	no predator	0.2137	1	136	20	8.83	17.27599
G. jazdzewski	no predator	0.2137	1	136	21	8.83	17.15852
G. jazdzewski	no predator	0.2137	1	136	22	8.8	16.2844
G. jazdzewski	no predator	0.2137	1	136	23	8.81	19.76459
G. jazdzewski	no predator	0.2137	1	136	24	8.79	24.88328
G. jazdzewski	no predator	0.2137	1	136	25	8.8	29.85507
G. jazdzewski	no predator	0.2137	1	136	26	8.78	25.70346
G. jazdzewski	no predator	0.2137	1	136	27	8.78	27.98002
G. jazdzewski	no predator	0.2137	1	136	28	8.79	26.57445
G. jazdzewski	no predator	0.2137	1	136	29	8.76	23.98372
G. jazdzewski	no predator	0.2137	1	136	30	8.78	28.07996
G. jazdzewski	no predator	0.2655	1	137	1	8.83	27.63997
G. jazdzewski	no predator	0.2655	1	137	2	8.85	20.90033
G. jazdzewski	no predator	0.2655	1	137	3	8.84	32.88034
G. jazdzewski	no predator	0.2655	1	137	4	8.83	25.35843
G. jazdzewski	no predator	0.2655	1	137	5	8.84	29.98126
G. jazdzewski	no predator	0.2655	1	137	6	8.84	26.45895

Gammarid species	treatment	weight [g]	pre-exposure time	trial	time [min.]	oxygen level [mg/l]	activity
G. jazdzewski	no predator	0.2655	1	137	7	8.84	25.88051
G. jazdzewski	no predator	0.2655	1	137	8	8.82	31.66252
G. jazdzewski	no predator	0.2655	1	137	9	8.82	39.87929
G. jazdzewski	no predator	0.2655	1	137	10	8.82	35.44079
G. jazdzewski	no predator	0.2655	1	137	11	8.8	33.83923
G. jazdzewski	no predator	0.2655	1	137	12	8.8	31.26089
G. jazdzewski	no predator	0.2655	1	137	13	8.79	36.43903
G. jazdzewski	no predator	0.2655	1	137	14	8.78	31.15997
G. jazdzewski	no predator	0.2655	1	137	15	8.79	29.94216
G. jazdzewski	no predator	0.2655	1	137	16	8.76	26.71217
G. jazdzewski	no predator	0.2655	1	137	17	8.75	23.04937
G. jazdzewski	no predator	0.2655	1	137	18	8.74	27.25643
G. jazdzewski	no predator	0.2655	1	137	19	8.74	23.89742
G. jazdzewski	no predator	0.2655	1	137	20	8.74	30.66537
G. jazdzewski	no predator	0.2655	1	137	21	8.72	37.31859
G. jazdzewski	no predator	0.2655	1	137	22	8.72	33.95703
G. jazdzewski	no predator	0.2655	1	137	23	8.72	23.34464
G. jazdzewski	no predator	0.2655	1	137	24	8.71	32.81997
G. jazdzewski	no predator	0.2655	1	137	25	8.7	28.25828
G. jazdzewski	no predator	0.2655	1	137	26	8.69	28.38513
G. jazdzewski	no predator	0.2655	1	137	27	8.68	29.06188
G. jazdzewski	no predator	0.2655	1	137	28	8.67	24.24384
G. jazdzewski	no predator	0.2655	1	137	29	8.66	35.14011
G. jazdzewski	no predator	0.2655	1	137	30	8.65	35.63814
G. jazdzewski	no predator	0.2158	1	138	1	8.85	20.8598
G. jazdzewski	no predator	0.2158	1	138	2	8.84	23.82023
G. jazdzewski	no predator	0.2158	1	138	3	8.83	19.81968
G. jazdzewski	no predator	0.2158	1	138	4	8.84	26.0799
G. jazdzewski	no predator	0.2158	1	138	5	8.82	24.3218
G. jazdzewski	no predator	0.2158	1	138	6	8.81	27.46009
G. jazdzewski	no predator	0.2158	1	138	7	8.8	33.01884
G. jazdzewski	no predator	0.2158	1	138	8	8.79	28.38127
G. jazdzewski	no predator	0.2158	1	138	9	8.79	32.60075
G. jazdzewski	no predator	0.2158	1	138	10	8.78	25.18004
G. jazdzewski	no predator	0.2158	1	138	11	8.76	28.92336
G. jazdzewski	no predator	0.2158	1	138	12	8.75	41.59909
G. jazdzewski	no predator	0.2158	1	138	13	8.75	38.95996
G. jazdzewski	no predator	0.2158	1	138	14	8.74	30.68108
G. jazdzewski	no predator	0.2158	1	138	15	8.73	34.63893
G. jazdzewski	no predator	0.2158	1	138	16	8.72	29.21652
G. jazdzewski	no predator	0.2158	1	138	17	8.7	20.68586
G. jazdzewski	no predator	0.2158	1	138	18	8.71	26.33757
G. jazdzewski	no predator	0.2158	1	138	19	8.7	23.44133
G. jazdzewski	no predator	0.2158	1	138	20	8.7	20.69733
G. jazdzewski	no predator	0.2158	1	138	21	8.68	22.36283
G. jazdzewski	no predator	0.2158	1	138	22	8.68	17.12013
G. jazdzewski	no predator	0.2158	1	138	23	8.68	18.4585
G. jazdzewski	no predator	0.2158	1	138	24	8.66	18.06163

Gammarid species	treatment	weight [g]	pre-exposure time	trial	time [min.]	oxygen level [mg/l]	activity
G. jazdzewski	no predator	0.2158	1	138	25	8.66	16.98344
G. jazdzewski	no predator	0.2158	1	138	26	8.64	22.89491
G. jazdzewski	no predator	0.2158	1	138	27	8.63	18.44361
G. jazdzewski	no predator	0.2158	1	138	28	8.63	20.80008
G. jazdzewski	no predator	0.2158	1	138	29	8.63	24.67621
G. jazdzewski	no predator	0.2158	1	138	30	8.6	20.71798
G. jazdzewski	no predator	0.2182	1	139	1	8.67	22.07949
G. jazdzewski	no predator	0.2182	1	139	2	8.68	16.97989
G. jazdzewski	no predator	0.2182	1	139	3	8.68	15.54061
G. jazdzewski	no predator	0.2182	1	139	4	8.69	18.25993
G. jazdzewski	no predator	0.2182	1	139	5	8.69	11.71952
G. jazdzewski	no predator	0.2182	1	139	6	8.7	22.18038
G. jazdzewski	no predator	0.2182	1	139	7	8.7	20.47931
G. jazdzewski	no predator	0.2182	1	139	8	8.72	19.10252
G. jazdzewski	no predator	0.2182	1	139	9	8.72	20.73867
G. jazdzewski	no predator	0.2182	1	139	10	8.7	18.85922
G. jazdzewski	no predator	0.2182	1	139	11	8.7	16.5799
G. jazdzewski	no predator	0.2182	1	139	12	8.69	21.50255
G. jazdzewski	no predator	0.2182	1	139	13	8.69	22.6209
G. jazdzewski	no predator	0.2182	1	139	14	8.68	17.45803
G. jazdzewski	no predator	0.2182	1	139	15	8.67	22.74421
G. jazdzewski	no predator	0.2182	1	139	16	8.68	18.35899
G. jazdzewski	no predator	0.2182	1	139	17	8.66	23.68001
G. jazdzewski	no predator	0.2182	1	139	18	8.67	21.74506
G. jazdzewski	no predator	0.2182	1	139	19	8.65	21.63626
G. jazdzewski	no predator	0.2182	1	139	20	8.64	15.01865
G. jazdzewski	no predator	0.2182	1	139	21	8.63	23.62984
G. jazdzewski	no predator	0.2182	1	139	22	8.61	20.57273
G. jazdzewski	no predator	0.2182	1	139	23	8.59	10.48313
G. jazdzewski	no predator	0.2182	1	139	24	8.58	10.85365
G. jazdzewski	no predator	0.2182	1	139	25	8.59	9.864941
G. jazdzewski	no predator	0.2182	1	139	26	8.59	16.90169
G. jazdzewski	no predator	0.2182	1	139	27	8.59	13.07464
G. jazdzewski	no predator	0.2182	1	139	28	8.58	12.54359
G. jazdzewski	no predator	0.2182	1	139	29	8.58	15.42189
G. jazdzewski	no predator	0.2182	1	139	30	8.59	11.97809
G. jazdzewski	no predator	0.2577	1	140	1	8.73	32.01957
G. jazdzewski	no predator	0.2577	1	140	2	8.74	30.78167
G. jazdzewski	no predator	0.2577	1	140	3	8.71	36.43947
G. jazdzewski	no predator	0.2577	1	140	4	8.71	44.17955
G. jazdzewski	no predator	0.2577	1	140	5	8.71	34.80234
G. jazdzewski	no predator	0.2577	1	140	6	8.71	38.63963
G. jazdzewski	no predator	0.2577	1	140	7	8.71	38.47891
G. jazdzewski	no predator	0.2577	1	140	8	8.7	31.36129
G. jazdzewski	no predator	0.2577	1	140	9	8.7	31.25933
G. jazdzewski	no predator	0.2577	1	140	10	8.68	24.82078
G. jazdzewski	no predator	0.2577	1	140	11	8.66	29.84085
G. jazdzewski	no predator	0.2577	1	140	12	8.67	30.49915

Gammarid species	treatment	weight [g]	pre-exposure time	trial	time [min.]	oxygen level [mg/l]	activity
G. jazdzewski	no predator	0.2577	1	140	13	8.64	31.13897
G. jazdzewski	no predator	0.2577	1	140	14	8.63	24.06607
G. jazdzewski	no predator	0.2577	1	140	15	8.63	27.57703
G. jazdzewski	no predator	0.2577	1	140	16	8.62	35.2233
G. jazdzewski	no predator	0.2577	1	140	17	8.6	34.31887
G. jazdzewski	no predator	0.2577	1	140	18	8.59	23.85754
G. jazdzewski	no predator	0.2577	1	140	19	8.59	24.10509
G. jazdzewski	no predator	0.2577	1	140	20	8.58	24.43592
G. jazdzewski	no predator	0.2577	1	140	21	8.58	18.04283
G. jazdzewski	no predator	0.2577	1	140	22	8.56	23.78294
G. jazdzewski	no predator	0.2577	1	140	23	8.56	20.25699
G. jazdzewski	no predator	0.2577	1	140	24	8.55	15.22321
G. jazdzewski	no predator	0.2577	1	140	25	8.53	15.34176
G. jazdzewski	no predator	0.2577	1	140	26	8.53	5.033291
G. jazdzewski	no predator	0.2577	1	140	27	8.52	2.078238
G. jazdzewski	no predator	0.2577	1	140	28	8.5	0.999989
G. jazdzewski	no predator	0.2577	1	140	29	8.48	1.339987
G. jazdzewski	no predator	0.2577	1	140	30	8.49	0.359997
G. jazdzewski	perch	0.245	1	149	1	8.86	36.94035
G. jazdzewski	perch	0.245	1	149	2	8.84	29.48039
G. jazdzewski	perch	0.245	1	149	3	8.85	28.41834
G. jazdzewski	perch	0.245	1	149	4	8.85	33.32141
G. jazdzewski	perch	0.245	1	149	5	8.84	31.52035
G. jazdzewski	perch	0.245	1	149	6	8.84	32.38094
G. jazdzewski	perch	0.245	1	149	7	8.82	30.07999
G. jazdzewski	perch	0.245	1	149	8	8.81	28.59996
G. jazdzewski	perch	0.245	1	149	9	8.81	26.52494
G. jazdzewski	perch	0.245	1	149	10	8.8	29.81556
G. jazdzewski	perch	0.245	1	149	11	8.79	35.10484
G. jazdzewski	perch	0.245	1	149	12	8.79	27.67937
G. jazdzewski	perch	0.245	1	149	13	8.78	37.64013
G. jazdzewski	perch	0.245	1	149	14	8.77	24.93309
G. jazdzewski	perch	0.245	1	149	15	8.76	24.82197
G. jazdzewski	perch	0.245	1	149	16	8.75	23.73989
G. jazdzewski	perch	0.245	1	149	17	8.75	19.18113
G. jazdzewski	perch	0.245	1	149	18	8.74	30.6786
G. jazdzewski	perch	0.245	1	149	19	8.75	21.50122
G. jazdzewski	perch	0.245	1	149	20	8.72	32.01849
G. jazdzewski	perch	0.245	1	149	21	8.73	30.86269
G. jazdzewski	perch	0.245	1	149	22	8.71	33.32292
G. jazdzewski	perch	0.245	1	149	23	8.72	30.82305
G. jazdzewski	perch	0.245	1	149	24	8.71	34.91995
G. jazdzewski	perch	0.245	1	149	25	8.7	37.59657
G. jazdzewski	perch	0.245	1	149	26	8.7	32.94333
G. jazdzewski	perch	0.245	1	149	27	8.69	30.30179
G. jazdzewski	perch	0.245	1	149	28	8.68	31.88378
G. jazdzewski	perch	0.245	1	149	29	8.7	31.80208
G. jazdzewski	perch	0.245	1	149	30	8.67	33.08833

Gammarid species	treatment	weight [g]	pre-exposure time	trial	time [min.]	oxygen level [mg/l]	activity
G. jazdzewski	perch	0.3371	1	150	1	8.79	15.97979
G. jazdzewski	perch	0.3371	1	150	2	8.79	14.70051
G. jazdzewski	perch	0.3371	1	150	3	8.8	16.16039
G. jazdzewski	perch	0.3371	1	150	4	8.79	15.81967
G. jazdzewski	perch	0.3371	1	150	5	8.8	17.97952
G. jazdzewski	perch	0.3371	1	150	6	8.8	11.01946
G. jazdzewski	perch	0.3371	1	150	7	8.8	14.8011
G. jazdzewski	perch	0.3371	1	150	8	8.79	8.100699
G. jazdzewski	perch	0.3371	1	150	9	8.79	17.39994
G. jazdzewski	perch	0.3371	1	150	10	8.77	8.219259
G. jazdzewski	perch	0.3371	1	150	11	8.78	7.399132
G. jazdzewski	perch	0.3371	1	150	12	8.77	12.60168
G. jazdzewski	perch	0.3371	1	150	13	8.77	7.921905
G. jazdzewski	perch	0.3371	1	150	14	8.77	9.299076
G. jazdzewski	perch	0.3371	1	150	15	8.75	10.68205
G. jazdzewski	perch	0.3371	1	150	16	8.75	6.661142
G. jazdzewski	perch	0.3371	1	150	17	8.75	9.36003
G. jazdzewski	perch	0.3371	1	150	18	8.74	4.297559
G. jazdzewski	perch	0.3371	1	150	19	8.74	4.07996
G. jazdzewski	perch	0.3371	1	150	20	8.73	4.48134
G. jazdzewski	perch	0.3371	1	150	21	8.71	2.642914
G. jazdzewski	perch	0.3371	1	150	22	8.7	4.037137
G. jazdzewski	perch	0.3371	1	150	23	8.7	1.03999
G. jazdzewski	perch	0.3371	1	150	24	8.67	1.919981
G. jazdzewski	perch	0.3371	1	150	25	8.68	0.919991
G. jazdzewski	perch	0.3371	1	150	26	8.67	2.159983
G. jazdzewski	perch	0.3371	1	150	27	8.67	1.359986
G. jazdzewski	perch	0.3371	1	150	28	8.66	1.221848
G. jazdzewski	perch	0.3371	1	150	29	8.66	1.278127
G. jazdzewski	perch	0.3371	1	150	30	8.67	3.039976
G. jazdzewski	perch	0.2712	1	151	1	8.85	16.83995
G. jazdzewski	perch	0.2712	1	151	2	8.84	17.70024
G. jazdzewski	perch	0.2712	1	151	3	8.83	12.22001
G. jazdzewski	perch	0.2712	1	151	4	8.82	16.61996
G. jazdzewski	perch	0.2712	1	151	5	8.81	16.50037
G. jazdzewski	perch	0.2712	1	151	6	8.8	17.42049
G. jazdzewski	perch	0.2712	1	151	7	8.81	19.09998
G. jazdzewski	perch	0.2712	1	151	8	8.82	18.65928
G. jazdzewski	perch	0.2712	1	151	9	8.81	18.20059
G. jazdzewski	perch	0.2712	1	151	10	8.78	18.75915
G. jazdzewski	perch	0.2712	1	151	11	8.79	20.20237
G. jazdzewski	perch	0.2712	1	151	12	8.79	16.64008
G. jazdzewski	perch	0.2712	1	151	13	8.78	12.45626
G. jazdzewski	perch	0.2712	1	151	14	8.74	15.02292
G. jazdzewski	perch	0.2712	1	151	15	8.74	13.70005
G. jazdzewski	perch	0.2712	1	151	16	8.71	13.58003
G. jazdzewski	perch	0.2712	1	151	17	8.69	15.11755
G. jazdzewski	perch	0.2712	1	151	18	8.67	16.96362

Gammarid species	treatment	weight [g]	pre-exposure time	trial	time [min.]	oxygen level [mg/l]	activity
3. jazdzewski	perch	0.2712	1	151	19	8.69	18.14
3. jazdzewski	perch	0.2712	1	151	20	8.69	26.26407
3. jazdzewski	perch	0.2712	1	151	21	8.67	23.35431
3. jazdzewski	perch	0.2712	1	151	22	8.68	19.02437
3. jazdzewski	perch	0.2712	1	151	23	8.7	27.59841
3. jazdzewski	perch	0.2712	1	151	24	8.68	24.94797
3. jazdzewski	perch	0.2712	1	151	25	8.66	20.05512
3. jazdzewski	perch	0.2712	1	151	26	8.68	17.26174
3. jazdzewski	perch	0.2712	1	151	27	8.64	14.4965
3. jazdzewski	perch	0.2712	1	151	28	8.66	19.36544
3. jazdzewski	perch	0.2712	1	151	29	8.65	14.42201
3. jazdzewski	perch	0.2712	1	151	30	8.65	14.61025
3. jazdzewski	perch	0.2513	1	152	1	8.85	29.46018
3. jazdzewski	perch	0.2513	1	152	2	8.83	25.52035
3. jazdzewski	perch	0.2513	1	152	3	8.85	27.86007
3. jazdzewski	perch	0.2513	1	152	4	8.85	32.77959
3. jazdzewski	perch	0.2513	1	152	5	8.85	34.76043
3. jazdzewski	perch	0.2513	1	152	6	8.84	40.16159
3. jazdzewski	perch	0.2513	1	152	7	8.83	35.55779
3. jazdzewski	perch	0.2513	1	152	8	8.81	29.88132
3. jazdzewski	perch	0.2513	1	152	9	8.81	27.03938
3. jazdzewski	perch	0.2513	1	152	10	8.8	28.78001
3. jazdzewski	perch	0.2513	1	152	11	8.79	28.68248
3. jazdzewski	perch	0.2513	1	152	12	8.78	27.79924
3. jazdzewski	perch	0.2513	1	152	13	8.78	25.24203
3. jazdzewski	perch	0.2513	1	152	14	8.77	34.54109
3. jazdzewski	perch	0.2513	1	152	15	8.75	32.33692
3. jazdzewski	perch	0.2513	1	152	16	8.75	36.76563
3. jazdzewski	perch	0.2513	1	152	17	8.74	35.59298
3. jazdzewski	perch	0.2513	1	152	18	8.72	32.70483
3. jazdzewski	perch	0.2513	1	152	19	8.72	30.70268
3. jazdzewski	perch	0.2513	1	152	20	8.7	30.01327
3. jazdzewski	perch	0.2513	1	152	21	8.71	19.28139
3. jazdzewski	perch	0.2513	1	152	22	8.69	23.73695
3. jazdzewski	perch	0.2513	1	152	23	8.7	17.99989
3. jazdzewski	perch	0.2513	1	152	24	8.68	16.68638
3. jazdzewski	perch	0.2513	1	152	25	8.67	18.15676
3. jazdzewski	perch	0.2513	1	152	26	8.65	17.04344
3. jazdzewski	perch	0.2513	1	152	27	8.65	24.0764
3. jazdzewski	perch	0.2513	1	152	28	8.64	17.93989
3. jazdzewski	perch	0.2513	1	152	29	8.62	14.93992
3. jazdzewski	perch	0.2513	1	152	30	8.62	9.983922
3. jazdzewski	perch	0.298	1	153	1	8.81	23.08036
3. jazdzewski	perch	0.298	1	153	2	8.81	24.64023
3. jazdzewski	perch	0.298	1	153	3	8.8	24.11855
3. jazdzewski	perch	0.298	1	153	4	8.78	16.92235
3. jazdzewski	perch	0.298	1	153	5	8.75	16.39917
3. jazdzewski	perch	0.298	1	153	6	8.77	14.30113

Gammarid species	treatment	weight [g]	pre-exposure time	trial	time [min.]	oxygen level [mg/l]	activity
G. jazdzewski	perch	0.298	1	153	7	8.76	22.54181
G. jazdzewski	perch	0.298	1	153	8	8.75	25.95811
G. jazdzewski	perch	0.298	1	153	9	8.75	26.38202
G. jazdzewski	perch	0.298	1	153	10	8.74	23.97851
G. jazdzewski	perch	0.298	1	153	11	8.75	19.25993
G. jazdzewski	perch	0.298	1	153	12	8.71	20.62075
G. jazdzewski	perch	0.298	1	153	13	8.69	16.98188
G. jazdzewski	perch	0.298	1	153	14	8.6	17.00003
G. jazdzewski	perch	0.298	1	153	15	8.55	23.73887
G. jazdzewski	perch	0.298	1	153	16	8.58	24.33762
G. jazdzewski	perch	0.298	1	153	17	8.59	17.22464
G. jazdzewski	perch	0.298	1	153	18	8.61	17.50006
G. jazdzewski	perch	0.298	1	153	19	8.57	16.29744
G. jazdzewski	perch	0.298	1	153	20	8.48	19.36131
G. jazdzewski	perch	0.298	1	153	21	7.94	10.63857
G. jazdzewski	perch	0.298	1	153	22	8.22	11.37988
G. jazdzewski	perch	0.298	1	153	23	7.99	16.97983
G. jazdzewski	perch	0.298	1	153	24	8.11	15.36146
G. jazdzewski	perch	0.298	1	153	25	7.8	16.84492
G. jazdzewski	perch	0.298	1	153	26	8.1	9.996597
G. jazdzewski	perch	0.298	1	153	27	8.02	6.561796
G. jazdzewski	perch	0.298	1	153	28	7.92	1.91824
G. jazdzewski	perch	0.298	1	153	29	8.11	3.638105
G. jazdzewski	perch	0.298	1	153	30	8.15	2.599973
G. jazdzewski	perch	0.2493	1	154	1	8.81	38.41981
G. jazdzewski	perch	0.2493	1	154	2	8.8	34.2397
G. jazdzewski	perch	0.2493	1	154	3	8.79	34.47851
G. jazdzewski	perch	0.2493	1	154	4	8.79	33.10265
G. jazdzewski	perch	0.2493	1	154	5	8.76	40.51911
G. jazdzewski	perch	0.2493	1	154	6	8.76	35.64197
G. jazdzewski	perch	0.2493	1	154	7	8.76	38.64118
G. jazdzewski	perch	0.2493	1	154	8	8.76	37.52011
G. jazdzewski	perch	0.2493	1	154	9	8.73	33.1187
G. jazdzewski	perch	0.2493	1	154	10	8.73	35.1208
G. jazdzewski	perch	0.2493	1	154	11	8.73	39.73751
G. jazdzewski	perch	0.2493	1	154	12	8.71	30.97981
G. jazdzewski	perch	0.2493	1	154	13	8.69	27.36368
G. jazdzewski	perch	0.2493	1	154	14	8.69	27.78111
G. jazdzewski	perch	0.2493	1	154	15	8.68	29.98006
G. jazdzewski	perch	0.2493	1	154	16	8.68	28.70122
G. jazdzewski	perch	0.2493	1	154	17	8.66	35.59766
G. jazdzewski	perch	0.2493	1	154	18	8.68	33.1625
G. jazdzewski	perch	0.2493	1	154	19	8.65	27.12013
G. jazdzewski	perch	0.2493	1	154	20	8.64	26.35741
G. jazdzewski	perch	0.2493	1	154	21	8.65	29.98287
G. jazdzewski	perch	0.2493	1	154	22	8.63	35.00444
G. jazdzewski	perch	0.2493	1	154	23	8.62	32.91702
G. jazdzewski	perch	0.2493	1	154	24	8.61	30.81518

Gammarid species	treatment	weight [g]	pre-exposure time	trial	time [min.]	oxygen level [mg/l]	activity
G. jazdzewski	perch	0.2493	1	154	25	8.62	30.3866
G. jazdzewski	perch	0.2493	1	154	26	8.59	36.69309
G. jazdzewski	perch	0.2493	1	154	27	8.58	42.78696
G. jazdzewski	perch	0.2493	1	154	28	8.58	25.94899
G. jazdzewski	perch	0.2493	1	154	29	8.57	33.80932
G. jazdzewski	perch	0.2493	1	154	30	8.56	33.57606
G. jazdzewski	perch	0.244	1	155	1	8.81	23.01898
G. jazdzewski	perch	0.244	1	155	2	8.81	20.92141
G. jazdzewski	perch	0.244	1	155	3	8.79	18.77808
G. jazdzewski	perch	0.244	1	155	4	8.78	16.36076
G. jazdzewski	perch	0.244	1	155	5	8.79	24.27992
G. jazdzewski	perch	0.244	1	155	6	8.79	18.54054
G. jazdzewski	perch	0.244	1	155	7	8.77	22.53941
G. jazdzewski	perch	0.244	1	155	8	8.76	22.3793
G. jazdzewski	perch	0.244	1	155	9	8.75	23.00421
G. jazdzewski	perch	0.244	1	155	10	8.77	34.64002
G. jazdzewski	perch	0.244	1	155	11	8.75	27.32002
G. jazdzewski	perch	0.244	1	155	12	8.76	21.09919
G. jazdzewski	perch	0.244	1	155	13	8.75	22.96289
G. jazdzewski	perch	0.244	1	155	14	8.75	23.33712
G. jazdzewski	perch	0.244	1	155	15	8.72	21.44111
G. jazdzewski	perch	0.244	1	155	16	8.72	28.44109
G. jazdzewski	perch	0.244	1	155	17	8.71	33.00116
G. jazdzewski	perch	0.244	1	155	18	8.72	25.85632
G. jazdzewski	perch	0.244	1	155	19	8.69	27.14254
G. jazdzewski	perch	0.244	1	155	20	8.71	22.51732
G. jazdzewski	perch	0.244	1	155	21	8.67	22.7985
G. jazdzewski	perch	0.244	1	155	22	8.6	18.08144
G. jazdzewski	perch	0.244	1	155	23	8.6	17.52469
G. jazdzewski	perch	0.244	1	155	24	8.53	10.0121
G. jazdzewski	perch	0.244	1	155	25	8.44	10.8483
G. jazdzewski	perch	0.244	1	155	26	8.44	12.87321
G. jazdzewski	perch	0.244	1	155	27	8.38	14.89991
G. jazdzewski	perch	0.244	1	155	28	8.35	15.26177
G. jazdzewski	perch	0.244	1	155	29	8.35	14.0819
G. jazdzewski	perch	0.244	1	155	30	8.41	19.92189
G. jazdzewski	perch	0.222	1	156	1	8.78	26.12004
G. jazdzewski	perch	0.222	1	156	2	8.75	28.24045
G. jazdzewski	perch	0.222	1	156	3	8.75	28.25997
G. jazdzewski	perch	0.222	1	156	4	8.78	27.3604
G. jazdzewski	perch	0.222	1	156	5	8.79	27.18002
G. jazdzewski	perch	0.222	1	156	6	8.78	27.69894
G. jazdzewski	perch	0.222	1	156	7	8.77	34.60284
G. jazdzewski	perch	0.222	1	156	8	8.79	37.27873
G. jazdzewski	perch	0.222	1	156	9	8.77	32.75998
G. jazdzewski	perch	0.222	1	156	10	8.77	34.9623
G. jazdzewski	perch	0.222	1	156	11	8.76	31.61343
G. jazdzewski	perch	0.222	1	156	12	8.74	23.24248

Gammarid species	treatment	weight [g]	pre-exposure time	trial	time [min.]	oxygen level [mg/l]	activity
G. jazdzewski	perch	0.222	1	156	13	8.73	28.27991
G. jazdzewski	perch	0.222	1	156	14	8.73	22.66098
G. jazdzewski	perch	0.222	1	156	15	8.72	34.90312
G. jazdzewski	perch	0.222	1	156	16	8.71	36.74118
G. jazdzewski	perch	0.222	1	156	17	8.7	32.47897
G. jazdzewski	perch	0.222	1	156	18	8.71	34.97628
G. jazdzewski	perch	0.222	1	156	19	8.68	27.29997
G. jazdzewski	perch	0.222	1	156	20	8.69	25.3386
G. jazdzewski	perch	0.222	1	156	21	8.68	30.14563
G. jazdzewski	perch	0.222	1	156	22	8.67	30.64962
G. jazdzewski	perch	0.222	1	156	23	8.67	30.81217
G. jazdzewski	perch	0.222	1	156	24	8.65	28.70884
G. jazdzewski	perch	0.222	1	156	25	8.67	29.94816
G. jazdzewski	perch	0.222	1	156	26	8.64	29.25484
G. jazdzewski	perch	0.222	1	156	27	8.64	24.50712
G. jazdzewski	perch	0.222	1	156	28	8.65	31.17454
G. jazdzewski	perch	0.222	1	156	29	8.64	33.54177
G. jazdzewski	perch	0.222	1	156	30	8.62	28.84392
G. jazdzewski	perch	0.2754	1	157	1	8.73	21.68014
G. jazdzewski	perch	0.2754	1	157	2	8.72	24.93964
G. jazdzewski	perch	0.2754	1	157	3	8.73	21.18135
G. jazdzewski	perch	0.2754	1	157	4	8.74	21.40051
G. jazdzewski	perch	0.2754	1	157	5	8.74	27.42009
G. jazdzewski	perch	0.2754	1	157	6	8.74	16.37912
G. jazdzewski	perch	0.2754	1	157	7	8.74	25.61877
G. jazdzewski	perch	0.2754	1	157	8	8.73	25.60183
G. jazdzewski	perch	0.2754	1	157	9	8.72	25.73994
G. jazdzewski	perch	0.2754	1	157	10	8.71	20.53919
G. jazdzewski	perch	0.2754	1	157	11	8.72	28.2832
G. jazdzewski	perch	0.2754	1	157	12	8.71	31.81913
G. jazdzewski	perch	0.2754	1	157	13	8.68	29.92283
G. jazdzewski	perch	0.2754	1	157	14	8.69	24.09713
G. jazdzewski	perch	0.2754	1	157	15	8.67	20.10113
G. jazdzewski	perch	0.2754	1	157	16	8.66	26.49661
G. jazdzewski	perch	0.2754	1	157	17	8.65	20.7671
G. jazdzewski	perch	0.2754	1	157	18	8.65	23.47394
G. jazdzewski	perch	0.2754	1	157	19	8.63	22.51733
G. jazdzewski	perch	0.2754	1	157	20	8.61	20.36262
G. jazdzewski	perch	0.2754	1	157	21	8.6	19.16142
G. jazdzewski	perch	0.2754	1	157	22	8.61	20.12003
G. jazdzewski	perch	0.2754	1	157	23	8.6	16.15853
G. jazdzewski	perch	0.2754	1	157	24	8.58	13.97841
G. jazdzewski	perch	0.2754	1	157	25	8.57	12.68167
G. jazdzewski	perch	0.2754	1	157	26	8.58	16.03828
G. jazdzewski	perch	0.2754	1	157	27	8.57	19.29634
G. jazdzewski	perch	0.2754	1	157	28	8.58	18.36354
G. jazdzewski	perch	0.2754	1	157	29	8.56	19.92185
G. jazdzewski	perch	0.2754	1	157	30	8.54	20.12191

Gammarid species	treatment	weight [g]	pre-exposure time	trial	time [min.]	oxygen level [mg/l]	activity
G. jazdzewski	perch	0.2577	1	158	1	8.71	31.31981
G. jazdzewski	perch	0.2577	1	158	2	8.71	33.14087
G. jazdzewski	perch	0.2577	1	158	3	8.71	40.24009
G. jazdzewski	perch	0.2577	1	158	4	8.7	35.15887
G. jazdzewski	perch	0.2577	1	158	5	8.68	34.30039
G. jazdzewski	perch	0.2577	1	158	6	8.66	31.51998
G. jazdzewski	perch	0.2577	1	158	7	8.68	28.80061
G. jazdzewski	perch	0.2577	1	158	8	8.67	31.08004
G. jazdzewski	perch	0.2577	1	158	9	8.68	35.46001
G. jazdzewski	perch	0.2577	1	158	10	8.67	34.66078
G. jazdzewski	perch	0.2577	1	158	11	8.64	27.61924
G. jazdzewski	perch	0.2577	1	158	12	8.64	25.97913
G. jazdzewski	perch	0.2577	1	158	13	8.63	28.48091
G. jazdzewski	perch	0.2577	1	158	14	8.63	29.51899
G. jazdzewski	perch	0.2577	1	158	15	8.63	29.17994
G. jazdzewski	perch	0.2577	1	158	16	8.63	24.90107
G. jazdzewski	perch	0.2577	1	158	17	8.61	25.22118
G. jazdzewski	perch	0.2577	1	158	18	8.59	25.69999
G. jazdzewski	perch	0.2577	1	158	19	8.59	20.16268
G. jazdzewski	perch	0.2577	1	158	20	8.58	21.21462
G. jazdzewski	perch	0.2577	1	158	21	8.58	19.00137
G. jazdzewski	perch	0.2577	1	158	22	8.59	15.44002
G. jazdzewski	perch	0.2577	1	158	23	8.57	19.45842
G. jazdzewski	perch	0.2577	1	158	24	8.56	24.08312
G. jazdzewski	perch	0.2577	1	158	25	8.56	20.74172
G. jazdzewski	perch	0.2577	1	158	26	8.54	30.39993
G. jazdzewski	perch	0.2577	1	158	27	8.55	20.60004
G. jazdzewski	perch	0.2577	1	158	28	8.54	24.21815
G. jazdzewski	perch	0.2577	1	158	29	8.51	19.08193
G. jazdzewski	perch	0.2577	1	158	30	8.52	27.52393
G. jazdzewski	perch	0.2775	1	159	1	8.7	14.65997
G. jazdzewski	perch	0.2775	1	159	2	8.72	16.09968
G. jazdzewski	perch	0.2775	1	159	3	8.71	18.30059
G. jazdzewski	perch	0.2775	1	159	4	8.72	18.99957
G. jazdzewski	perch	0.2775	1	159	5	8.72	17.77995
G. jazdzewski	perch	0.2775	1	159	6	8.74	21.781
G. jazdzewski	perch	0.2775	1	159	7	8.74	26.13992
G. jazdzewski	perch	0.2775	1	159	8	8.73	20.47998
G. jazdzewski	perch	0.2775	1	159	9	8.73	19.22072
G. jazdzewski	perch	0.2775	1	159	10	8.71	24.21916
G. jazdzewski	perch	0.2775	1	159	11	8.71	19.76329
G. jazdzewski	perch	0.2775	1	159	12	8.71	17.4957
G. jazdzewski	perch	0.2775	1	159	13	8.69	19.02083
G. jazdzewski	perch	0.2775	1	159	14	8.69	22.0999
G. jazdzewski	perch	0.2775	1	159	15	8.66	16.38428
G. jazdzewski	perch	0.2775	1	159	16	8.65	18.75904
G. jazdzewski	perch	0.2775	1	159	17	8.64	30.61879
G. jazdzewski	perch	0.2775	1	159	18	8.63	26.27747

Gammarid species	treatment	weight [g]	pre-exposure time	trial	time [min.]	oxygen level [mg/l]	activity
G. jazdzewski	perch	0.2775	1	159	19	8.63	15.38128
G. jazdzewski	perch	0.2775	1	159	20	8.61	16.92824
G. jazdzewski	perch	0.2775	1	159	21	8.6	18.72022
G. jazdzewski	perch	0.2775	1	159	22	8.6	20.71266
G. jazdzewski	perch	0.2775	1	159	23	8.59	14.8999
G. jazdzewski	perch	0.2775	1	159	24	8.58	22.0847
G. jazdzewski	perch	0.2775	1	159	25	8.58	20.30004
G. jazdzewski	perch	0.2775	1	159	26	8.57	18.89825
G. jazdzewski	perch	0.2775	1	159	27	8.55	19.54531
G. jazdzewski	perch	0.2775	1	159	28	8.53	27.16191
G. jazdzewski	perch	0.2775	1	159	29	8.53	15.41444
G. jazdzewski	perch	0.2775	1	159	30	8.52	9.754141
G. jazdzewski	perch	0.2661	1	160	1	8.68	22.9603
G. jazdzewski	perch	0.2661	1	160	2	8.68	27.25944
G. jazdzewski	perch	0.2661	1	160	3	8.67	19.35963
G. jazdzewski	perch	0.2661	1	160	4	8.69	19.12078
G. jazdzewski	perch	0.2661	1	160	5	8.69	19.96005
G. jazdzewski	perch	0.2661	1	160	6	8.68	21.5605
G. jazdzewski	perch	0.2661	1	160	7	8.67	31.15993
G. jazdzewski	perch	0.2661	1	160	8	8.67	27.68326
G. jazdzewski	perch	0.2661	1	160	9	8.67	30.23802
G. jazdzewski	perch	0.2661	1	160	10	8.66	31.60078
G. jazdzewski	perch	0.2661	1	160	11	8.66	31.38082
G. jazdzewski	perch	0.2661	1	160	12	8.66	31.36004
G. jazdzewski	perch	0.2661	1	160	13	8.66	31.70093
G. jazdzewski	perch	0.2661	1	160	14	8.65	30.78005
G. jazdzewski	perch	0.2661	1	160	15	8.64	30.2422
G. jazdzewski	perch	0.2661	1	160	16	8.64	32.84236
G. jazdzewski	perch	0.2661	1	160	17	8.63	31.71651
G. jazdzewski	perch	0.2661	1	160	18	8.62	28.06368
G. jazdzewski	perch	0.2661	1	160	19	8.6	29.27869
G. jazdzewski	perch	0.2661	1	160	20	8.59	26.16406
G. jazdzewski	perch	0.2661	1	160	21	8.6	31.03574
G. jazdzewski	perch	0.2661	1	160	22	8.59	31.46287
G. jazdzewski	perch	0.2661	1	160	23	8.56	21.43694
G. jazdzewski	perch	0.2661	1	160	24	8.55	26.15831
G. jazdzewski	perch	0.2661	1	160	25	8.57	24.04324
G. jazdzewski	perch	0.2661	1	160	26	8.56	26.71823
G. jazdzewski	perch	0.2661	1	160	27	8.54	25.14172
G. jazdzewski	perch	0.2661	1	160	28	8.53	26.0437
G. jazdzewski	perch	0.2661	1	160	29	8.54	33.76194
G. jazdzewski	perch	0.2661	1	160	30	8.5	24.76012
D. villosus	no predator	0.5056	7	161	1	8.75	13.47987
D. villosus	no predator	0.5056	7	161	2	8.74	10.86139
D. villosus	no predator	0.5056	7	161	3	8.73	15.07937
D. villosus	no predator	0.5056	7	161	4	8.71	11.72085
D. villosus	no predator	0.5056	7	161	5	8.7	14.00059
D. villosus	no predator	0.5056	7	161	6	8.68	16.87905

Gammarid species	treatment	weight [g]	pre-exposure time	trial	time [min.]	oxygen level [mg/l]	activity
D. villosus	no predator	0.5056	7	161	7	8.67	14.33884
D. villosus	no predator	0.5056	7	161	8	8.66	2.480035
D. villosus	no predator	0.5056	7	161	9	8.65	18.96053
D. villosus	no predator	0.5056	7	161	10	8.62	4.560796
D. villosus	no predator	0.5056	7	161	11	8.61	12.38079
D. villosus	no predator	0.5056	7	161	12	8.61	4.601872
D. villosus	no predator	0.5056	7	161	13	8.59	14.15627
D. villosus	no predator	0.5056	7	161	14	8.57	9.579906
D. villosus	no predator	0.5056	7	161	15	8.55	6.722099
D. villosus	no predator	0.5056	7	161	16	8.52	7.039984
D. villosus	no predator	0.5056	7	161	17	8.5	6.559935
D. villosus	no predator	0.5056	7	161	18	8.48	10.46116
D. villosus	no predator	0.5056	7	161	19	8.47	5.258691
D. villosus	no predator	0.5056	7	161	20	8.47	11.64265
D. villosus	no predator	0.5056	7	161	21	8.44	8.597146
D. villosus	no predator	0.5056	7	161	22	8.42	11.05989
D. villosus	no predator	0.5056	7	161	23	8.41	4.319956
D. villosus	no predator	0.5056	7	161	24	8.41	5.339951
D. villosus	no predator	0.5056	7	161	25	8.41	5.626661
D. villosus	no predator	0.5056	7	161	26	8.37	8.834875
D. villosus	no predator	0.5056	7	161	27	8.33	6.585332
D. villosus	no predator	0.5056	7	161	28	8.33	9.474495
D. villosus	no predator	0.5056	7	161	29	8.32	2.301897
D. villosus	no predator	0.5056	7	161	30	8.3	5.881991
D. villosus	no predator	0.4707	7	162	1	8.86	9.640388
D. villosus	no predator	0.4707	7	162	2	8.81	16.04049
D. villosus	no predator	0.4707	7	162	3	8.77	9.259368
D. villosus	no predator	0.4707	7	162	4	8.74	10.73996
D. villosus	no predator	0.4707	7	162	5	8.71	13.18042
D. villosus	no predator	0.4707	7	162	6	8.68	13.40107
D. villosus	no predator	0.4707	7	162	7	8.66	12.6783
D. villosus	no predator	0.4707	7	162	8	8.65	8.339978
D. villosus	no predator	0.4707	7	162	9	8.64	8.89997
D. villosus	no predator	0.4707	7	162	10	8.61	12.48151
D. villosus	no predator	0.4707	7	162	11	8.59	11.83838
D. villosus	no predator	0.4707	7	162	12	8.58	8.060886
D. villosus	no predator	0.4707	7	162	13	8.57	9.783863
D. villosus	no predator	0.4707	7	162	14	8.56	12.71713
D. villosus	no predator	0.4707	7	162	15	8.53	9.820031
D. villosus	no predator	0.4707	7	162	16	8.53	8.960027
D. villosus	no predator	0.4707	7	162	17	8.53	13.46114
D. villosus	no predator	0.4707	7	162	18	8.51	13.56238
D. villosus	no predator	0.4707	7	162	19	8.51	9.080035
D. villosus	no predator	0.4707	7	162	20	8.53	10.6214
D. villosus	no predator	0.4707	7	162	21	8.5	11.69575
D. villosus	no predator	0.4707	7	162	22	8.13	9.439906
D. villosus	no predator	0.4707	7	162	23	7.94	8.324598
D. villosus	no predator	0.4707	7	162	24	7.94	12.09682

Gammarid species	treatment	weight [g]	pre-exposure time	trial	time [min.]	oxygen level [mg/l]	activity
D. villosus	no predator	0.4707	7	162	25	8.14	3.258347
D. villosus	no predator	0.4707	7	162	26	8.09	7.219928
D. villosus	no predator	0.4707	7	162	27	8.02	6.139942
D. villosus	no predator	0.4707	7	162	28	7.74	7.521789
D. villosus	no predator	0.4707	7	162	29	7.93	8.338051
D. villosus	no predator	0.4707	7	162	30	8.07	7.389827
D. villosus	no predator	0.5604	7	163	1	8.79	7.339873
D. villosus	no predator	0.5604	7	163	2	8.76	6.479454
D. villosus	no predator	0.5604	7	163	3	8.77	1.960343
D. villosus	no predator	0.5604	7	163	4	8.75	7.419988
D. villosus	no predator	0.5604	7	163	5	8.71	9.980444
D. villosus	no predator	0.5604	7	163	6	8.71	12.8
D. villosus	no predator	0.5604	7	163	7	8.67	10.00117
D. villosus	no predator	0.5604	7	163	8	8.67	11.01875
D. villosus	no predator	0.5604	7	163	9	8.65	9.560677
D. villosus	no predator	0.5604	7	163	10	8.63	11.04235
D. villosus	no predator	0.5604	7	163	11	8.61	17.0576
D. villosus	no predator	0.5604	7	163	12	8.59	8.660035
D. villosus	no predator	0.5604	7	163	13	8.57	6.900057
D. villosus	no predator	0.5604	7	163	14	8.55	11.14204
D. villosus	no predator	0.5604	7	163	15	8.53	7.535855
D. villosus	no predator	0.5604	7	163	16	8.5	6.739935
D. villosus	no predator	0.5604	7	163	17	8.5	6.179941
D. villosus	no predator	0.5604	7	163	18	8.49	9.523692
D. villosus	no predator	0.5604	7	163	19	8.49	10.90265
D. villosus	no predator	0.5604	7	163	20	8.47	12.06138
D. villosus	no predator	0.5604	7	163	21	8.45	11.81575
D. villosus	no predator	0.5604	7	163	22	8.43	14.86585
D. villosus	no predator	0.5604	7	163	23	8.42	14.55698
D. villosus	no predator	0.5604	7	163	24	8.42	15.66152
D. villosus	no predator	0.5604	7	163	25	8.4	15.76165
D. villosus	no predator	0.5604	7	163	26	8.4	12.33658
D. villosus	no predator	0.5604	7	163	27	8.36	15.3399
D. villosus	no predator	0.5604	7	163	28	8.33	13.20179
D. villosus	no predator	0.5604	7	163	29	8.31	9.618104
D. villosus	no predator	0.5604	7	163	30	8.29	12.35796
D. villosus	no predator	0.6466	7	164	1	8.84	16.62128
D. villosus	no predator	0.6466	7	164	2	8.81	16.19865
D. villosus	no predator	0.6466	7	164	3	8.77	12.16096
D. villosus	no predator	0.6466	7	164	4	8.73	9.418826
D. villosus	no predator	0.6466	7	164	5	8.72	13.10132
D. villosus	no predator	0.6466	7	164	6	8.7	12.8795
D. villosus	no predator	0.6466	7	164	7	8.67	10.35882
D. villosus	no predator	0.6466	7	164	8	8.64	6.699936
D. villosus	no predator	0.6466	7	164	9	8.61	9.161355
D. villosus	no predator	0.6466	7	164	10	8.6	7.160826
D. villosus	no predator	0.6466	7	164	11	8.58	10.55925
D. villosus	no predator	0.6466	7	164	12	8.57	9.199134

Gammarid species	treatment	weight [g]	pre-exposure time	trial	time [min.]	oxygen level [mg/l]	activity
D. villosus	no predator	0.6466	7	164	13	8.53	8.182798
D. villosus	no predator	0.6466	7	164	14	8.54	7.358003
D. villosus	no predator	0.6466	7	164	15	8.53	11.16098
D. villosus	no predator	0.6466	7	164	16	8.49	9.302185
D. villosus	no predator	0.6466	7	164	17	8.48	9.737631
D. villosus	no predator	0.6466	7	164	18	8.48	7.361185
D. villosus	no predator	0.6466	7	164	19	8.44	4.621334
D. villosus	no predator	0.6466	7	164	20	8.42	2.418715
D. villosus	no predator	0.6466	7	164	21	8.4	4.938571
D. villosus	no predator	0.6466	7	164	22	8.38	9.439908
D. villosus	no predator	0.6466	7	164	23	8.38	3.299967
D. villosus	no predator	0.6466	7	164	24	8.35	3.801582
D. villosus	no predator	0.6466	7	164	25	8.33	7.101673
D. villosus	no predator	0.6466	7	164	26	8.31	10.82698
D. villosus	no predator	0.6466	7	164	27	8.29	7.913022
D. villosus	no predator	0.6466	7	164	28	8.29	9.423683
D. villosus	no predator	0.6466	7	164	29	8.29	8.094342
D. villosus	no predator	0.6466	7	164	30	8.27	19.14177
D. villosus	no predator	0.5662	7	165	1	8.9	7.860162
D. villosus	no predator	0.5662	7	165	2	8.87	18.52018
D. villosus	no predator	0.5662	7	165	3	8.84	20.74129
D. villosus	no predator	0.5662	7	165	4	8.81	19.19957
D. villosus	no predator	0.5662	7	165	5	8.77	15.46095
D. villosus	no predator	0.5662	7	165	6	8.75	21.59941
D. villosus	no predator	0.5662	7	165	7	8.72	17.43993
D. villosus	no predator	0.5662	7	165	8	8.7	13.11999
D. villosus	no predator	0.5662	7	165	9	8.67	10.53929
D. villosus	no predator	0.5662	7	165	10	8.66	14.16065
D. villosus	no predator	0.5662	7	165	11	8.63	13.78244
D. villosus	no predator	0.5662	7	165	12	8.61	10.09744
D. villosus	no predator	0.5662	7	165	13	8.58	11.60092
D. villosus	no predator	0.5662	7	165	14	8.59	12.25898
D. villosus	no predator	0.5662	7	165	15	8.58	8.978894
D. villosus	no predator	0.5662	7	165	16	8.55	10.78218
D. villosus	no predator	0.5662	7	165	17	8.55	10.99995
D. villosus	no predator	0.5662	7	165	18	8.52	5.718748
D. villosus	no predator	0.5662	7	165	19	8.5	9.179913
D. villosus	no predator	0.5662	7	165	20	8.49	11.52127
D. villosus	no predator	0.5662	7	165	21	8.48	10.47996
D. villosus	no predator	0.5662	7	165	22	8.46	10.30296
D. villosus	no predator	0.5662	7	165	23	8.45	9.340024
D. villosus	no predator	0.5662	7	165	24	8.42	7.360053
D. villosus	no predator	0.5662	7	165	25	8.42	4.136719
D. villosus	no predator	0.5662	7	165	26	8.4	8.319924
D. villosus	no predator	0.5662	7	165	27	8.38	6.521736
D. villosus	no predator	0.5662	7	165	28	8.35	7.739925
D. villosus	no predator	0.5662	7	165	29	8.35	6.159935
D. villosus	no predator	0.5662	7	165	30	8.33	3.96194

Gammarid species	treatment	weight [g]	pre-exposure time	trial	time [min.]	oxygen level [mg/l]	activity
D. villosus	no predator	0.6026	7	166	1	8.94	4.339597
D. villosus	no predator	0.6026	7	166	2	8.89	7.540228
D. villosus	no predator	0.6026	7	166	3	8.88	2.479675
D. villosus	no predator	0.6026	7	166	4	8.84	7.079941
D. villosus	no predator	0.6026	7	166	5	8.81	2.139979
D. villosus	no predator	0.6026	7	166	6	8.78	1.279987
D. villosus	no predator	0.6026	7	166	7	8.75	0.839991
D. villosus	no predator	0.6026	7	166	8	8.73	3.599961
D. villosus	no predator	0.6026	7	166	9	8.71	0.919991
D. villosus	no predator	0.6026	7	166	10	8.69	0.419996
D. villosus	no predator	0.6026	7	166	11	8.68	0.379996
D. villosus	no predator	0.6026	7	166	12	8.65	4.379958
D. villosus	no predator	0.6026	7	166	13	8.63	0.079999
D. villosus	no predator	0.6026	7	166	14	8.62	0.379996
D. villosus	no predator	0.6026	7	166	15	8.59	3.479965
D. villosus	no predator	0.6026	7	166	16	8.55	1.581124
D. villosus	no predator	0.6026	7	166	17	8.54	6.744737
D. villosus	no predator	0.6026	7	166	18	8.52	5.796404
D. villosus	no predator	0.6026	7	166	19	8.52	5.999942
D. villosus	no predator	0.6026	7	166	20	8.51	0.659994
D. villosus	no predator	0.6026	7	166	21	8.49	0.519995
D. villosus	no predator	0.6026	7	166	22	8.47	9.579908
D. villosus	no predator	0.6026	7	166	23	8.45	5.081508
D. villosus	no predator	0.6026	7	166	24	8.43	1.398426
D. villosus	no predator	0.6026	7	166	25	8.41	1.99998
D. villosus	no predator	0.6026	7	166	26	8.4	2.819972
D. villosus	no predator	0.6026	7	166	27	8.37	0.059999
D. villosus	no predator	0.6026	7	166	28	8.34	1.459985
D. villosus	no predator	0.6026	7	166	29	8.32	0
D. villosus	no predator	0.6026	7	166	30	8.32	1.279986
D. villosus	no predator	0.588	7	167	1	8.76	8.159731
D. villosus	no predator	0.588	7	167	2	8.74	5.301149
D. villosus	no predator	0.588	7	167	3	8.73	21.63858
D. villosus	no predator	0.588	7	167	4	8.71	6.439933
D. villosus	no predator	0.588	7	167	5	8.68	3.940442
D. villosus	no predator	0.588	7	167	6	8.64	3.459489
D. villosus	no predator	0.588	7	167	7	8.61	7.179928
D. villosus	no predator	0.588	7	167	8	8.58	1.520645
D. villosus	no predator	0.588	7	167	9	8.56	9.362122
D. villosus	no predator	0.588	7	167	10	8.56	12.497
D. villosus	no predator	0.588	7	167	11	8.54	2.17998
D. villosus	no predator	0.588	7	167	12	8.51	0.699993
D. villosus	no predator	0.588	7	167	13	8.5	3.319967
D. villosus	no predator	0.588	7	167	14	8.48	2.139978
D. villosus	no predator	0.588	7	167	15	8.46	2.801053
D. villosus	no predator	0.588	7	167	16	8.45	3.618884
D. villosus	no predator	0.588	7	167	17	8.43	1.901181
D. villosus	no predator	0.588	7	167	18	8.41	4.83875

Gammarid species	treatment	weight [g]	pre-exposure time	trial	time [min.]	oxygen level [mg/l]	activity
D. villosus	no predator	0.588	7	167	19	8.38	1.159988
D. villosus	no predator	0.588	7	167	20	8.39	9.619905
D. villosus	no predator	0.588	7	167	21	8.39	6.739924
D. villosus	no predator	0.588	7	167	22	8.37	10.13991
D. villosus	no predator	0.588	7	167	23	8.35	5.341508
D. villosus	no predator	0.588	7	167	24	8.36	8.078361
D. villosus	no predator	0.588	7	167	25	8.34	2.659973
D. villosus	no predator	0.588	7	167	26	8.32	6.043424
D. villosus	no predator	0.588	7	167	27	8.31	7.656453
D. villosus	no predator	0.588	7	167	28	8.3	5.259945
D. villosus	no predator	0.588	7	167	29	8.28	5.421866
D. villosus	no predator	0.588	7	167	30	8.27	6.33802
D. villosus	no predator	0.6168	7	168	1	8.68	9.720019
D. villosus	no predator	0.6168	7	168	2	8.65	5.940359
D. villosus	no predator	0.6168	7	168	3	8.63	10.079
D. villosus	no predator	0.6168	7	168	4	8.61	5.440364
D. villosus	no predator	0.6168	7	168	5	8.59	6.400953
D. villosus	no predator	0.6168	7	168	6	8.56	5.899458
D. villosus	no predator	0.6168	7	168	7	8.54	4.639952
D. villosus	no predator	0.6168	7	168	8	8.52	4.740612
D. villosus	no predator	0.6168	7	168	9	8.49	9.962781
D. villosus	no predator	0.6168	7	168	10	8.46	6.397061
D. villosus	no predator	0.6168	7	168	11	8.45	9.582427
D. villosus	no predator	0.6168	7	168	12	8.43	9.420089
D. villosus	no predator	0.6168	7	168	13	8.41	6.741012
D. villosus	no predator	0.6168	7	168	14	8.39	9.198055
D. villosus	no predator	0.6168	7	168	15	8.38	7.018908
D. villosus	no predator	0.6168	7	168	16	8.38	6.859929
D. villosus	no predator	0.6168	7	168	17	8.34	8.424724
D. villosus	no predator	0.6168	7	168	18	8.32	7.458793
D. villosus	no predator	0.6168	7	168	19	8.31	3.918704
D. villosus	no predator	0.6168	7	168	20	8.32	2.639973
D. villosus	no predator	0.6168	7	168	21	8.32	6.182817
D. villosus	no predator	0.6168	7	168	22	8.33	5.657065
D. villosus	no predator	0.6168	7	168	23	8.31	3.03997
D. villosus	no predator	0.6168	7	168	24	8.32	6.639936
D. villosus	no predator	0.6168	7	168	25	8.3	1.439985
D. villosus	no predator	0.6168	7	168	26	8.29	1.699982
D. villosus	no predator	0.6168	7	168	27	8.29	4.139954
D. villosus	no predator	0.6168	7	168	28	8.28	0.899991
D. villosus	no predator	0.6168	7	168	29	8.27	0.739991
D. villosus	no predator	0.6168	7	168	30	8.27	0.439995
D. villosus	perch	0.5153	7	173	1	8.47	9.979891
D. villosus	perch	0.5153	7	173	2	8.45	15.88074
D. villosus	perch	0.5153	7	173	3	8.43	18.21892
D. villosus	perch	0.5153	7	173	4	8.41	3.281227
D. villosus	perch	0.5153	7	173	5	8.4	15.33906
D. villosus	perch	0.5153	7	173	6	8.37	11.21995

Gammarid species	treatment	weight [g]	pre-exposure time	trial	time [min.]	oxygen level [mg/l]	activity
D. villosus	perch	0.5153	7	173	7	8.37	5.679949
D. villosus	perch	0.5153	7	173	8	8.39	7.301904
D. villosus	perch	0.5153	7	173	9	8.35	6.518668
D. villosus	perch	0.5153	7	173	10	8.31	15.50068
D. villosus	perch	0.5153	7	173	11	8.3	10.63834
D. villosus	perch	0.5153	7	173	12	8.28	9.24531
D. villosus	perch	0.5153	7	173	13	8.26	10.03733
D. villosus	perch	0.5153	7	173	14	8.26	7.858006
D. villosus	perch	0.5153	7	173	15	8.24	3.482125
D. villosus	perch	0.5153	7	173	16	8.24	7.438839
D. villosus	perch	0.5153	7	173	17	8.22	3.399967
D. villosus	perch	0.5153	7	173	18	8.21	13.60238
D. villosus	perch	0.5153	7	173	19	8.21	4.557439
D. villosus	perch	0.5153	7	173	20	8.16	12.33989
D. villosus	perch	0.5153	7	173	21	8.16	5.379945
D. villosus	perch	0.5153	7	173	22	8.15	3.739964
D. villosus	perch	0.5153	7	173	23	8.13	1.98622
D. villosus	perch	0.5153	7	173	24	8.12	5.280127
D. villosus	perch	0.5153	7	173	25	8.12	1.555124
D. villosus	perch	0.5153	7	173	26	8.11	3.419964
D. villosus	perch	0.5153	7	173	27	8.09	6.619937
D. villosus	perch	0.5153	7	173	28	8.08	2.319976
D. villosus	perch	0.5153	7	173	29	8.01	1.179988
D. villosus	perch	0.5153	7	173	30	7.76	0.779992
D. villosus	perch	0.4578	7	174	1	8.56	13.92064
D. villosus	perch	0.4578	7	174	2	8.53	7.798963
D. villosus	perch	0.4578	7	174	3	8.52	3.299963
D. villosus	perch	0.4578	7	174	4	8.47	2.960811
D. villosus	perch	0.4578	7	174	5	8.45	0.639153
D. villosus	perch	0.4578	7	174	6	8.42	0.499995
D. villosus	perch	0.4578	7	174	7	8.4	1.219988
D. villosus	perch	0.4578	7	174	8	8.4	0
D. villosus	perch	0.4578	7	174	9	8.38	0.119999
D. villosus	perch	0.4578	7	174	10	8.36	0.199998
D. villosus	perch	0.4578	7	174	11	8.35	0.399996
D. villosus	perch	0.4578	7	174	12	8.34	0
D. villosus	perch	0.4578	7	174	13	8.32	0.339997
D. villosus	perch	0.4578	7	174	14	8.32	0.119999
D. villosus	perch	0.4578	7	174	15	8.31	0.279997
D. villosus	perch	0.4578	7	174	16	8.31	0.359996
D. villosus	perch	0.4578	7	174	17	8.3	0.119999
D. villosus	perch	0.4578	7	174	18	8.31	0.099999
D. villosus	perch	0.4578	7	174	19	8.3	0.379996
D. villosus	perch	0.4578	7	174	20	8.31	0
D. villosus	perch	0.4578	7	174	21	8.31	0.139999
D. villosus	perch	0.4578	7	174	22	8.3	0.359996
D. villosus	perch	0.4578	7	174	23	8.32	0.219998
D. villosus	perch	0.4578	7	174	24	8.32	0.079999

Gammarid species	treatment	weight [g]	pre-exposure time	trial	time [min.]	oxygen level [mg/l]	activity
D. villosus	perch	0.4578	7	174	25	8.32	0.219997
D. villosus	perch	0.4578	7	174	26	8.32	0.199998
D. villosus	perch	0.4578	7	174	27	8.32	0.201798
D. villosus	perch	0.4578	7	174	28	8.32	0.118199
D. villosus	perch	0.4578	7	174	29	8.33	0.02
D. villosus	perch	0.4578	7	174	30	8.34	0.239998
D. villosus	perch	0.5064	7	175	1	8.52	4.200681
D. villosus	perch	0.5064	7	175	2	8.49	7.759809
D. villosus	perch	0.5064	7	175	3	8.47	2.300457
D. villosus	perch	0.5064	7	175	4	8.43	2.519315
D. villosus	perch	0.5064	7	175	5	8.4	3.219548
D. villosus	perch	0.5064	7	175	6	8.39	1.919982
D. villosus	perch	0.5064	7	175	7	8.34	0.059999
D. villosus	perch	0.5064	7	175	8	8.32	3.779968
D. villosus	perch	0.5064	7	175	9	8.29	0.779993
D. villosus	perch	0.5064	7	175	10	8.26	3.419967
D. villosus	perch	0.5064	7	175	11	8.24	0.619993
D. villosus	perch	0.5064	7	175	12	8.09	2.420877
D. villosus	perch	0.5064	7	175	13	8.06	0.499095
D. villosus	perch	0.5064	7	175	14	8.07	4.719956
D. villosus	perch	0.5064	7	175	15	8.08	3.99996
D. villosus	perch	0.5064	7	175	16	8.08	0.659994
D. villosus	perch	0.5064	7	175	17	8.08	3.599964
D. villosus	perch	0.5064	7	175	18	8.06	4.079963
D. villosus	perch	0.5064	7	175	19	8.07	2.721293
D. villosus	perch	0.5064	7	175	20	8.05	0.238677
D. villosus	perch	0.5064	7	175	21	8.03	6.339937
D. villosus	perch	0.5064	7	175	22	8.01	3.999959
D. villosus	perch	0.5064	7	175	23	7.95	0.02
D. villosus	perch	0.5064	7	175	24	7.86	3.319967
D. villosus	perch	0.5064	7	175	25	7.7	0.599994
D. villosus	perch	0.5064	7	175	26	7.6	0.199998
D. villosus	perch	0.5064	7	175	27	7.55	1.279988
D. villosus	perch	0.5064	7	175	28	7.61	0.239998
D. villosus	perch	0.5064	7	175	29	7.62	0.479995
D. villosus	perch	0.5064	7	175	30	7.6	1.779982
D. villosus	perch	0.354	7	176	1	8.47	4.740069
D. villosus	perch	0.354	7	176	2	8.44	4.800017
D. villosus	perch	0.354	7	176	3	8.41	5.819646
D. villosus	perch	0.354	7	176	4	8.39	1.839981
D. villosus	perch	0.354	7	176	5	8.37	7.200883
D. villosus	perch	0.354	7	176	6	8.35	4.999536
D. villosus	perch	0.354	7	176	7	8.33	4.560556
D. villosus	perch	0.354	7	176	8	8.34	4.279356
D. villosus	perch	0.354	7	176	9	8.32	9.162071
D. villosus	perch	0.354	7	176	10	8.31	5.177778
D. villosus	perch	0.354	7	176	11	8.31	3.759963
D. villosus	perch	0.354	7	176	12	8.3	7.160832

Gammarid species	treatment	weight [g]	pre-exposure time	trial	time [min.]	oxygen level [mg/l]	activity
D. villosus	perch	0.354	7	176	13	8.29	9.098999
D. villosus	perch	0.354	7	176	14	8.27	4.759956
D. villosus	perch	0.354	7	176	15	8.26	2.859971
D. villosus	perch	0.354	7	176	16	8.25	6.579937
D. villosus	perch	0.354	7	176	17	8.24	3.07997
D. villosus	perch	0.354	7	176	18	8.22	5.519947
D. villosus	perch	0.354	7	176	19	8.23	4.359958
D. villosus	perch	0.354	7	176	20	8.2	6.705462
D. villosus	perch	0.354	7	176	21	8.21	5.114428
D. villosus	perch	0.354	7	176	22	8.2	1.199988
D. villosus	perch	0.354	7	176	23	8.19	5.181516
D. villosus	perch	0.354	7	176	24	8.19	1.840042
D. villosus	perch	0.354	7	176	25	8.19	3.07835
D. villosus	perch	0.354	7	176	26	8.17	3.319966
D. villosus	perch	0.354	7	176	27	8.16	2.303576
D. villosus	perch	0.354	7	176	28	8.16	3.456364
D. villosus	perch	0.354	7	176	29	8.16	1.91998
D. villosus	perch	0.354	7	176	30	8.15	0.97999
D. villosus	perch	0.5192	7	177	1	8.4	1.279988
D. villosus	perch	0.5192	7	177	2	8.38	1.199989
D. villosus	perch	0.5192	7	177	3	8.37	1.759983
D. villosus	perch	0.5192	7	177	4	8.36	1.699983
D. villosus	perch	0.5192	7	177	5	8.36	1.261428
D. villosus	perch	0.5192	7	177	6	8.34	3.460685
D. villosus	perch	0.5192	7	177	7	8.33	6.578323
D. villosus	perch	0.5192	7	177	8	8.33	2.619975
D. villosus	perch	0.5192	7	177	9	8.31	8.999919
D. villosus	perch	0.5192	7	177	10	8.29	0.98233
D. villosus	perch	0.5192	7	177	11	8.28	8.317585
D. villosus	perch	0.5192	7	177	12	8.27	0.099999
D. villosus	perch	0.5192	7	177	13	8.25	1.199989
D. villosus	perch	0.5192	7	177	14	8.24	0.279997
D. villosus	perch	0.5192	7	177	15	8.24	3.419968
D. villosus	perch	0.5192	7	177	16	8.22	0.199998
D. villosus	perch	0.5192	7	177	17	8.22	0.379996
D. villosus	perch	0.5192	7	177	18	8.2	0.539994
D. villosus	perch	0.5192	7	177	19	8.2	0.239997
D. villosus	perch	0.5192	7	177	20	8.18	0.279997
D. villosus	perch	0.5192	7	177	21	8.16	0.339996
D. villosus	perch	0.5192	7	177	22	8.16	3.979961
D. villosus	perch	0.5192	7	177	23	8.16	0.259997
D. villosus	perch	0.5192	7	177	24	8.14	0.159998
D. villosus	perch	0.5192	7	177	25	8.14	0
D. villosus	perch	0.5192	7	177	26	8.14	0.219998
D. villosus	perch	0.5192	7	177	27	8.13	0.659993
D. villosus	perch	0.5192	7	177	28	8.12	0.339997
D. villosus	perch	0.5192	7	177	29	8.13	0.319997
D. villosus	perch	0.5192	7	177	30	8.14	0.059999

Gammarid species	treatment	weight [g]	pre-exposure time	trial	time [min.]	oxygen level [mg/l]	activity
D. villosus	perch	0.5307	7	178	1	8.49	0.299997
D. villosus	perch	0.5307	7	178	2	8.48	0
D. villosus	perch	0.5307	7	178	3	8.43	0.02
D. villosus	perch	0.5307	7	178	4	8.4	0.04
D. villosus	perch	0.5307	7	178	5	8.38	0.339997
D. villosus	perch	0.5307	7	178	6	8.37	2.260518
D. villosus	perch	0.5307	7	178	7	8.36	0.099999
D. villosus	perch	0.5307	7	178	8	8.36	8.059914
D. villosus	perch	0.5307	7	178	9	8.35	0.099999
D. villosus	perch	0.5307	7	178	10	8.33	1.919981
D. villosus	perch	0.5307	7	178	11	8.29	2.099979
D. villosus	perch	0.5307	7	178	12	8.28	0
D. villosus	perch	0.5307	7	178	13	8.26	0
D. villosus	perch	0.5307	7	178	14	8.25	0.099999
D. villosus	perch	0.5307	7	178	15	8.26	0.079999
D. villosus	perch	0.5307	7	178	16	8.24	3.519967
D. villosus	perch	0.5307	7	178	17	8.23	1.639983
D. villosus	perch	0.5307	7	178	18	8.23	0
D. villosus	perch	0.5307	7	178	19	8.22	0.219998
D. villosus	perch	0.5307	7	178	20	8.23	0.299997
D. villosus	perch	0.5307	7	178	21	8.22	0
D. villosus	perch	0.5307	7	178	22	8.23	1.919982
D. villosus	perch	0.5307	7	178	23	8.23	1.159988
D. villosus	perch	0.5307	7	178	24	8.22	0.399996
D. villosus	perch	0.5307	7	178	25	8.23	0
D. villosus	perch	0.5307	7	178	26	8.23	0.02
D. villosus	perch	0.5307	7	178	27	8.23	0
D. villosus	perch	0.5307	7	178	28	8.23	0
D. villosus	perch	0.5307	7	178	29	8.22	0
D. villosus	perch	0.5307	7	178	30	8.19	0
D. villosus	perch	0.5075	7	179	1	8.39	15.51938
D. villosus	perch	0.5075	7	179	2	8.36	5.999706
D. villosus	perch	0.5075	7	179	3	8.33	6.741731
D. villosus	perch	0.5075	7	179	4	8.24	12.2785
D. villosus	perch	0.5075	7	179	5	7.89	4.139539
D. villosus	perch	0.5075	7	179	6	7.8	5.721022
D. villosus	perch	0.5075	7	179	7	7.81	3.878884
D. villosus	perch	0.5075	7	179	8	7.72	4.659953
D. villosus	perch	0.5075	7	179	9	7.8	3.082129
D. villosus	perch	0.5075	7	179	10	7.82	5.237784
D. villosus	perch	0.5075	7	179	11	7.85	1.479985
D. villosus	perch	0.5075	7	179	12	7.88	8.261732
D. villosus	perch	0.5075	7	179	13	7.89	3.519126
D. villosus	perch	0.5075	7	179	14	7.92	5.999944
D. villosus	perch	0.5075	7	179	15	7.94	5.28103
D. villosus	perch	0.5075	7	179	16	7.97	2.418898
D. villosus	perch	0.5075	7	179	17	7.97	2.342375
D. villosus	perch	0.5075	7	179	18	7.97	2.277577

Gammarid species	treatment	weight [g]	pre-exposure time	trial	time [min.]	oxygen level [mg/l]	activity
D. villosus	perch	0.5075	7	179	19	7.97	2.179979
D. villosus	perch	0.5075	7	179	20	7.99	2.881351
D. villosus	perch	0.5075	7	179	21	7.98	5.939945
D. villosus	perch	0.5075	7	179	22	7.99	3.801462
D. villosus	perch	0.5075	7	179	23	7.97	2.96153
D. villosus	perch	0.5075	7	179	24	7.98	4.198396
D. villosus	perch	0.5075	7	179	25	7.96	6.219944
D. villosus	perch	0.5075	7	179	26	7.96	5.579946
D. villosus	perch	0.5075	7	179	27	7.95	2.421776
D. villosus	perch	0.5075	7	179	28	7.94	3.238172
D. villosus	perch	0.5075	7	179	29	7.93	3.721885
D. villosus	perch	0.5075	7	179	30	7.91	3.263987
D. villosus	perch	0.4324	7	180	1	8.39	0.759992
D. villosus	perch	0.4324	7	180	2	8.36	2.19998
D. villosus	perch	0.4324	7	180	3	8.32	2.499976
D. villosus	perch	0.4324	7	180	4	8.26	0.89999
D. villosus	perch	0.4324	7	180	5	8.21	0.459995
D. villosus	perch	0.4324	7	180	6	8.17	0.599994
D. villosus	perch	0.4324	7	180	7	8.09	3.699967
D. villosus	perch	0.4324	7	180	8	7.99	1.619984
D. villosus	perch	0.4324	7	180	9	7.9	1.9407
D. villosus	perch	0.4324	7	180	10	7.88	0.99999
D. villosus	perch	0.4324	7	180	11	7.92	0.519995
D. villosus	perch	0.4324	7	180	12	7.95	0.279997
D. villosus	perch	0.4324	7	180	13	7.98	1.079989
D. villosus	perch	0.4324	7	180	14	7.99	0.799991
D. villosus	perch	0.4324	7	180	15	7.97	0.419995
D. villosus	perch	0.4324	7	180	16	7.96	0.099999
D. villosus	perch	0.4324	7	180	17	7.96	0
D. villosus	perch	0.4324	7	180	18	7.96	0.02
D. villosus	perch	0.4324	7	180	19	7.97	0.239997
D. villosus	perch	0.4324	7	180	20	8.01	0.839992
D. villosus	perch	0.4324	7	180	21	8.05	0.93999
D. villosus	perch	0.4324	7	180	22	8.08	0.359996
D. villosus	perch	0.4324	7	180	23	8.09	2.079978
D. villosus	perch	0.4324	7	180	24	8.09	0.199998
D. villosus	perch	0.4324	7	180	25	8.1	0.759993
D. villosus	perch	0.4324	7	180	26	8.1	0.719993
D. villosus	perch	0.4324	7	180	27	8.07	0.599994
D. villosus	perch	0.4324	7	180	28	8.08	0
D. villosus	perch	0.4324	7	180	29	8.06	1.159988
D. villosus	perch	0.4324	7	180	30	8.06	0.699993
D. villosus	perch	0.5372	7	181	1	8.17	0.519995
D. villosus	perch	0.5372	7	181	2	8.13	3.220567
D. villosus	perch	0.5372	7	181	3	8.09	1.220048
D. villosus	perch	0.5372	7	181	4	8.06	1.399986
D. villosus	perch	0.5372	7	181	5	8.04	10.24133
D. villosus	perch	0.5372	7	181	6	8.02	12.45952

Gammarid species	treatment	weight [g]	pre-exposure time	trial	time [min.]	oxygen level [mg/l]	activity
D. villosus	perch	0.5372	7	181	7	7.99	1.9589
D. villosus	perch	0.5372	7	181	8	7.97	0.119999
D. villosus	perch	0.5372	7	181	9	7.95	2.782852
D. villosus	perch	0.5372	7	181	10	7.94	1.457824
D. villosus	perch	0.5372	7	181	11	7.93	5.082469
D. villosus	perch	0.5372	7	181	12	7.91	2.81829
D. villosus	perch	0.5372	7	181	13	7.9	6.839935
D. villosus	perch	0.5372	7	181	14	7.9	6.845033
D. villosus	perch	0.5372	7	181	15	7.9	2.894871
D. villosus	perch	0.5372	7	181	16	7.89	5.319948
D. villosus	perch	0.5372	7	181	17	7.87	0.939991
D. villosus	perch	0.5372	7	181	18	7.87	4.179961
D. villosus	perch	0.5372	7	181	19	7.85	2.539975
D. villosus	perch	0.5372	7	181	20	7.82	0.401376
D. villosus	perch	0.5372	7	181	21	7.8	1.05861
D. villosus	perch	0.5372	7	181	22	7.77	2.199978
D. villosus	perch	0.5372	7	181	23	7.76	0.199998
D. villosus	perch	0.5372	7	181	24	7.74	1.159988
D. villosus	perch	0.5372	7	181	25	7.71	0.961671
D. villosus	perch	0.5372	7	181	26	7.69	2.0983
D. villosus	perch	0.5372	7	181	27	7.68	2.599974
D. villosus	perch	0.5372	7	181	28	7.68	1.499985
D. villosus	perch	0.5372	7	181	29	7.65	4.183803
D. villosus	perch	0.5372	7	181	30	7.66	9.956054
D. villosus	perch	0.4518	7	182	1	8.07	12.03898
D. villosus	perch	0.4518	7	182	2	8.02	4.801151
D. villosus	perch	0.4518	7	182	3	7.97	4.598755
D. villosus	perch	0.4518	7	182	4	7.94	8.162441
D. villosus	perch	0.4518	7	182	5	7.9	3.417866
D. villosus	perch	0.4518	7	182	6	7.85	4.279958
D. villosus	perch	0.4518	7	182	7	7.84	0.239997
D. villosus	perch	0.4518	7	182	8	7.81	1.599984
D. villosus	perch	0.4518	7	182	9	7.8	6.099934
D. villosus	perch	0.4518	7	182	10	7.82	20.28057
D. villosus	perch	0.4518	7	182	11	7.8	1.299207
D. villosus	perch	0.4518	7	182	12	7.76	2.839972
D. villosus	perch	0.4518	7	182	13	7.74	0.579993
D. villosus	perch	0.4518	7	182	14	7.73	0.379996
D. villosus	perch	0.4518	7	182	15	7.72	3.279969
D. villosus	perch	0.4518	7	182	16	7.75	11.44559
D. villosus	perch	0.4518	7	182	17	7.71	2.534274
D. villosus	perch	0.4518	7	182	18	7.7	2.579976
D. villosus	perch	0.4518	7	182	19	7.66	0.279997
D. villosus	perch	0.4518	7	182	20	7.67	1.239988
D. villosus	perch	0.4518	7	182	21	7.65	2.259978
D. villosus	perch	0.4518	7	182	22	7.64	0.379996
D. villosus	perch	0.4518	7	182	23	7.59	2.704652
D. villosus	perch	0.4518	7	182	24	7.62	11.95683

Gammarid species	treatment	weight [g]	pre-exposure time	trial	time [min.]	oxygen level [mg/l]	activity
D. villosus	perch	0.4518	7	182	25	7.59	1.858361
D. villosus	perch	0.4518	7	182	26	7.56	2.919971
D. villosus	perch	0.4518	7	182	27	7.6	8.76892
D. villosus	perch	0.4518	7	182	28	7.57	5.070951
D. villosus	perch	0.4518	7	182	29	7.56	0.439995
D. villosus	perch	0.4518	7	182	30	7.56	6.325886
D. villosus	perch	0.4999	7	183	1	8.52	0.579994
D. villosus	perch	0.4999	7	183	2	8.47	9.461102
D. villosus	perch	0.4999	7	183	3	8.42	6.078742
D. villosus	perch	0.4999	7	183	4	8.4	3.959961
D. villosus	perch	0.4999	7	183	5	8.37	0.240958
D. villosus	perch	0.4999	7	183	6	8.34	2.979011
D. villosus	perch	0.4999	7	183	7	8.31	3.181768
D. villosus	perch	0.4999	7	183	8	8.3	9.643324
D. villosus	perch	0.4999	7	183	9	8.29	9.595286
D. villosus	perch	0.4999	7	183	10	8.27	13.20221
D. villosus	perch	0.4999	7	183	11	8.26	7.318426
D. villosus	perch	0.4999	7	183	12	8.25	10.86175
D. villosus	perch	0.4999	7	183	13	8.25	11.05905
D. villosus	perch	0.4999	7	183	14	8.23	2.800032
D. villosus	perch	0.4999	7	183	15	8.2	13.95883
D. villosus	perch	0.4999	7	183	16	8.2	12.44444
D. villosus	perch	0.4999	7	183	17	8.18	16.19888
D. villosus	perch	0.4999	7	183	18	8.15	9.84248
D. villosus	perch	0.4999	7	183	19	8.14	9.560035
D. villosus	perch	0.4999	7	183	20	8.12	8.179913
D. villosus	perch	0.4999	7	183	21	8.1	9.522786
D. villosus	perch	0.4999	7	183	22	8.07	4.958514
D. villosus	perch	0.4999	7	183	23	8.08	19.92449
D. villosus	perch	0.4999	7	183	24	8.06	14.27841
D. villosus	perch	0.4999	7	183	25	8.02	11.90169
D. villosus	perch	0.4999	7	183	26	8	4.736657
D. villosus	perch	0.4999	7	183	27	8	5.901747
D. villosus	perch	0.4999	7	183	28	7.99	9.138117
D. villosus	perch	0.4999	7	183	29	7.97	3.801885
D. villosus	perch	0.4999	7	183	30	7.96	5.779999
D. villosus	perch	0.5111	7	184	1	8.33	0.179998
D. villosus	perch	0.5111	7	184	2	8.29	0.760292
D. villosus	perch	0.5111	7	184	3	8.26	0.120359
D. villosus	perch	0.5111	7	184	4	8.23	2.479975
D. villosus	perch	0.5111	7	184	5	8.2	4.319962
D. villosus	perch	0.5111	7	184	6	8.17	1.901061
D. villosus	perch	0.5111	7	184	7	8.16	0.678913
D. villosus	perch	0.5111	7	184	8	8.13	0.859991
D. villosus	perch	0.5111	7	184	9	8.1	1.759982
D. villosus	perch	0.5111	7	184	10	8.1	0.159999
D. villosus	perch	0.5111	7	184	11	8.07	0.079999
D. villosus	perch	0.5111	7	184	12	8.03	0.539994

Gammarid species	treatment	weight [g]	pre-exposure time	trial	time [min.]	oxygen level [mg/l]	activity
D. villosus	perch	0.5111	7	184	13	8.02	0.779992
D. villosus	perch	0.5111	7	184	14	8	2.139979
D. villosus	perch	0.5111	7	184	15	7.97	2.699975
D. villosus	perch	0.5111	7	184	16	7.93	1.899981
D. villosus	perch	0.5111	7	184	17	7.92	0.779993
D. villosus	perch	0.5111	7	184	18	7.9	2.339977
D. villosus	perch	0.5111	7	184	19	7.89	0.939991
D. villosus	perch	0.5111	7	184	20	7.9	0.02
D. villosus	perch	0.5111	7	184	21	7.94	5.619956
D. villosus	perch	0.5111	7	184	22	7.94	1.359986
D. villosus	perch	0.5111	7	184	23	7.93	2.279977
D. villosus	perch	0.5111	7	184	24	7.92	3.883201
D. villosus	perch	0.5111	7	184	25	7.92	13.35663
D. villosus	perch	0.5111	7	184	26	7.9	8.019921
D. villosus	perch	0.5111	7	184	27	7.88	2.619974
D. villosus	perch	0.5111	7	184	28	7.87	5.299953
D. villosus	perch	0.5111	7	184	29	7.85	2.939972
D. villosus	perch	0.5111	7	184	30	7.84	0.579994
G. jazdzewski	no predator	0.2664	7	187	1	8.75	12.19975
G. jazdzewski	no predator	0.2664	7	187	2	8.74	10.95995
G. jazdzewski	no predator	0.2664	7	187	3	8.71	11.14032
G. jazdzewski	no predator	0.2664	7	187	4	8.71	7.259211
G. jazdzewski	no predator	0.2664	7	187	5	8.69	8.841355
G. jazdzewski	no predator	0.2664	7	187	6	8.67	3.679064
G. jazdzewski	no predator	0.2664	7	187	7	8.64	8.979978
G. jazdzewski	no predator	0.2664	7	187	8	8.64	6.319335
G. jazdzewski	no predator	0.2664	7	187	9	8.62	2.942132
G. jazdzewski	no predator	0.2664	7	187	10	8.6	4.417796
G. jazdzewski	no predator	0.2664	7	187	11	8.59	2.359976
G. jazdzewski	no predator	0.2664	7	187	12	8.58	2.579974
G. jazdzewski	no predator	0.2664	7	187	13	8.58	3.500926
G. jazdzewski	no predator	0.2664	7	187	14	8.67	6.098986
G. jazdzewski	no predator	0.2664	7	187	15	8.71	3.01997
G. jazdzewski	no predator	0.2664	7	187	16	8.71	5.021091
G. jazdzewski	no predator	0.2664	7	187	17	8.71	5.078816
G. jazdzewski	no predator	0.2664	7	187	18	8.7	2.299978
G. jazdzewski	no predator	0.2664	7	187	19	8.66	9.179916
G. jazdzewski	no predator	0.2664	7	187	20	8.63	7.242687
G. jazdzewski	no predator	0.2664	7	187	21	8.65	6.780049
G. jazdzewski	no predator	0.2664	7	187	22	8.6	5.900059
G. jazdzewski	no predator	0.2664	7	187	23	8.58	6.096936
G. jazdzewski	no predator	0.2664	7	187	24	8.56	3.143208
G. jazdzewski	no predator	0.2664	7	187	25	8.57	5.356708
G. jazdzewski	no predator	0.2664	7	187	26	8.56	3.05997
G. jazdzewski	no predator	0.2664	7	187	27	8.56	4.699956
G. jazdzewski	no predator	0.2664	7	187	28	8.55	4.481817
G. jazdzewski	no predator	0.2664	7	187	29	8.54	10.48187
G. jazdzewski	no predator	0.2664	7	187	30	8.53	8.003997

Gammarid species	treatment	weight [g]	pre-exposure time	trial	time [min.]	oxygen level [mg/l]	activity
G. jazdzewski	no predator	0.283	7	188	1	8.8	8.859912
G. jazdzewski	no predator	0.283	7	188	2	8.76	8.539917
G. jazdzewski	no predator	0.283	7	188	3	8.76	5.740307
G. jazdzewski	no predator	0.283	7	188	4	8.75	2.879971
G. jazdzewski	no predator	0.283	7	188	5	8.74	8.319927
G. jazdzewski	no predator	0.283	7	188	6	8.73	2.900512
G. jazdzewski	no predator	0.283	7	188	7	8.73	10.44116
G. jazdzewski	no predator	0.283	7	188	8	8.72	5.818142
G. jazdzewski	no predator	0.283	7	188	9	8.71	2.999971
G. jazdzewski	no predator	0.283	7	188	10	8.7	6.779933
G. jazdzewski	no predator	0.283	7	188	11	8.69	4.399956
G. jazdzewski	no predator	0.283	7	188	12	8.68	5.641744
G. jazdzewski	no predator	0.283	7	188	13	8.68	14.62094
G. jazdzewski	no predator	0.283	7	188	14	8.66	4.738034
G. jazdzewski	no predator	0.283	7	188	15	8.64	1.139989
G. jazdzewski	no predator	0.283	7	188	16	8.63	2.903391
G. jazdzewski	no predator	0.283	7	188	17	8.61	9.098826
G. jazdzewski	no predator	0.283	7	188	18	8.6	2.960031
G. jazdzewski	no predator	0.283	7	188	19	8.59	5.202592
G. jazdzewski	no predator	0.283	7	188	20	8.59	6.520054
G. jazdzewski	no predator	0.283	7	188	21	8.58	5.397189
G. jazdzewski	no predator	0.283	7	188	22	8.56	5.384447
G. jazdzewski	no predator	0.283	7	188	23	8.58	7.415433
G. jazdzewski	no predator	0.283	7	188	24	8.56	5.163189
G. jazdzewski	no predator	0.283	7	188	25	8.54	0.700053
G. jazdzewski	no predator	0.283	7	188	26	8.51	7.141733
G. jazdzewski	no predator	0.283	7	188	27	8.52	5.120072
G. jazdzewski	no predator	0.283	7	188	28	8.5	2.756375
G. jazdzewski	no predator	0.283	7	188	29	8.5	9.859898
G. jazdzewski	no predator	0.283	7	188	30	8.49	3.679966
G. jazdzewski	no predator	0.1246	7	189	1	8.66	0.299997
G. jazdzewski	no predator	0.1246	7	189	2	8.6	0.840292
G. jazdzewski	no predator	0.1246	7	189	3	8.57	2.460035
G. jazdzewski	no predator	0.1246	7	189	4	8.55	3.639606
G. jazdzewski	no predator	0.1246	7	189	5	8.52	2.740936
G. jazdzewski	no predator	0.1246	7	189	6	8.5	3.160087
G. jazdzewski	no predator	0.1246	7	189	7	8.48	3.559482
G. jazdzewski	no predator	0.1246	7	189	8	8.47	5.359346
G. jazdzewski	no predator	0.1246	7	189	9	8.45	0.140719
G. jazdzewski	no predator	0.1246	7	189	10	8.45	2.299973
G. jazdzewski	no predator	0.1246	7	189	11	8.44	1.199988
G. jazdzewski	no predator	0.1246	7	189	12	8.43	3.499964
G. jazdzewski	no predator	0.1246	7	189	13	8.41	3.31997
G. jazdzewski	no predator	0.1246	7	189	14	8.41	1.079989
G. jazdzewski	no predator	0.1246	7	189	15	8.41	2.759972
G. jazdzewski	no predator	0.1246	7	189	16	8.4	0.859991
G. jazdzewski	no predator	0.1246	7	189	17	8.38	3.662362
G. jazdzewski	no predator	0.1246	7	189	18	8.38	4.258759

Gammarid species	treatment	weight [g]	pre-exposure time	trial	time [min.]	oxygen level [mg/l]	activity
G. jazdzewski	no predator	0.1246	7	189	19	8.38	4.061279
G. jazdzewski	no predator	0.1246	7	189	20	8.37	2.46004
G. jazdzewski	no predator	0.1246	7	189	21	8.38	5.621444
G. jazdzewski	no predator	0.1246	7	189	22	8.38	2.364477
G. jazdzewski	no predator	0.1246	7	189	23	8.37	0.555495
G. jazdzewski	no predator	0.1246	7	189	24	8.36	1.401606
G. jazdzewski	no predator	0.1246	7	189	25	8.37	8.939968
G. jazdzewski	no predator	0.1246	7	189	26	8.38	3.01829
G. jazdzewski	no predator	0.1246	7	189	27	8.39	3.859965
G. jazdzewski	no predator	0.1246	7	189	28	8.39	6.739936
G. jazdzewski	no predator	0.1246	7	189	29	8.4	2.861894
G. jazdzewski	no predator	0.1246	7	189	30	8.39	0.398076
G. jazdzewski	no predator	0.1115	7	190	1	8.82	18.65976
G. jazdzewski	no predator	0.1115	7	190	2	8.78	17.40083
G. jazdzewski	no predator	0.1115	7	190	3	8.76	20.59901
G. jazdzewski	no predator	0.1115	7	190	4	8.73	10.91917
G. jazdzewski	no predator	0.1115	7	190	5	8.71	13.00179
G. jazdzewski	no predator	0.1115	7	190	6	8.69	18.85899
G. jazdzewski	no predator	0.1115	7	190	7	8.68	28.72222
G. jazdzewski	no predator	0.1115	7	190	8	8.67	27.65677
G. jazdzewski	no predator	0.1115	7	190	9	8.65	26.78123
G. jazdzewski	no predator	0.1115	7	190	10	8.66	23.2015
G. jazdzewski	no predator	0.1115	7	190	11	8.64	27.39919
G. jazdzewski	no predator	0.1115	7	190	12	8.62	25.67998
G. jazdzewski	no predator	0.1115	7	190	13	8.61	20.89907
G. jazdzewski	no predator	0.1115	7	190	14	8.58	18.81994
G. jazdzewski	no predator	0.1115	7	190	15	8.57	20.06101
G. jazdzewski	no predator	0.1115	7	190	16	8.56	13.96341
G. jazdzewski	no predator	0.1115	7	190	17	8.59	21.72129
G. jazdzewski	no predator	0.1115	7	190	18	8.62	23.53257
G. jazdzewski	no predator	0.1115	7	190	19	8.64	16.86513
G. jazdzewski	no predator	0.1115	7	190	20	8.65	23.82414
G. jazdzewski	no predator	0.1115	7	190	21	8.59	9.157449
G. jazdzewski	no predator	0.1115	7	190	22	8.58	22.47409
G. jazdzewski	no predator	0.1115	7	190	23	8.55	14.28302
G. jazdzewski	no predator	0.1115	7	190	24	8.54	14.55996
G. jazdzewski	no predator	0.1115	7	190	25	8.54	13.59824
G. jazdzewski	no predator	0.1115	7	190	26	8.54	5.263425
G. jazdzewski	no predator	0.1115	7	190	27	8.53	12.8236
G. jazdzewski	no predator	0.1115	7	190	28	8.54	11.07641
G. jazdzewski	no predator	0.1115	7	190	29	8.53	10.92002
G. jazdzewski	no predator	0.1115	7	190	30	8.5	13.62389
G. jazdzewski	no predator	0.1644	7	191	1	8.7	19.08016
G. jazdzewski	no predator	0.1644	7	191	2	8.7	16.34002
G. jazdzewski	no predator	0.1644	7	191	3	8.67	16.82038
G. jazdzewski	no predator	0.1644	7	191	4	8.64	17.74006
G. jazdzewski	no predator	0.1644	7	191	5	8.63	13.56005
G. jazdzewski	no predator	0.1644	7	191	6	8.62	18.48054

Gammarid species	treatment	weight [g]	pre-exposure time	trial	time [min.]	oxygen level [mg/l]	activity
G. jazdzewski	no predator	0.1644	7	191	7	8.63	14.0213
G. jazdzewski	no predator	0.1644	7	191	8	8.62	16.43623
G. jazdzewski	no predator	0.1644	7	191	9	8.59	15.60272
G. jazdzewski	no predator	0.1644	7	191	10	8.58	13.84166
G. jazdzewski	no predator	0.1644	7	191	11	8.56	19.67843
G. jazdzewski	no predator	0.1644	7	191	12	8.56	11.61916
G. jazdzewski	no predator	0.1644	7	191	13	8.56	14.519
G. jazdzewski	no predator	0.1644	7	191	14	8.56	11.91995
G. jazdzewski	no predator	0.1644	7	191	15	8.57	16.22206
G. jazdzewski	no predator	0.1644	7	191	16	8.53	14.74111
G. jazdzewski	no predator	0.1644	7	191	17	8.53	21.61997
G. jazdzewski	no predator	0.1644	7	191	18	8.54	25.78368
G. jazdzewski	no predator	0.1644	7	191	19	8.52	21.3348
G. jazdzewski	no predator	0.1644	7	191	20	8.52	15.25989
G. jazdzewski	no predator	0.1644	7	191	21	8.52	12.38563
G. jazdzewski	no predator	0.1644	7	191	22	8.5	18.8185
G. jazdzewski	no predator	0.1644	7	191	23	8.49	18.48462
G. jazdzewski	no predator	0.1644	7	191	24	8.48	15.37522
G. jazdzewski	no predator	0.1644	7	191	25	8.46	17.36493
G. jazdzewski	no predator	0.1644	7	191	26	8.44	22.36177
G. jazdzewski	no predator	0.1644	7	191	27	8.43	18.86012
G. jazdzewski	no predator	0.1644	7	191	28	8.41	21.9963
G. jazdzewski	no predator	0.1644	7	191	29	8.39	19.938
G. jazdzewski	no predator	0.1644	7	191	30	8.38	20.10382
G. jazdzewski	no predator	0.2889	7	196	1	9.16	22.49965
G. jazdzewski	no predator	0.2889	7	196	2	9.16	19.38076
G. jazdzewski	no predator	0.2889	7	196	3	9.17	24.39926
G. jazdzewski	no predator	0.2889	7	196	4	9.15	17.32037
G. jazdzewski	no predator	0.2889	7	196	5	9.16	15.09955
G. jazdzewski	no predator	0.2889	7	196	6	9.16	17.74048
G. jazdzewski	no predator	0.2889	7	196	7	9.15	11.41945
G. jazdzewski	no predator	0.2889	7	196	8	9.15	12.20198
G. jazdzewski	no predator	0.2889	7	196	9	9.15	11.7373
G. jazdzewski	no predator	0.2889	7	196	10	9.13	12.75914
G. jazdzewski	no predator	0.2889	7	196	11	9.13	5.119944
G. jazdzewski	no predator	0.2889	7	196	12	9.12	14.26076
G. jazdzewski	no predator	0.2889	7	196	13	9.12	14.21894
G. jazdzewski	no predator	0.2889	7	196	14	9.11	9.941948
G. jazdzewski	no predator	0.2889	7	196	15	9.1	6.158982
G. jazdzewski	no predator	0.2889	7	196	16	9.09	13.23993
G. jazdzewski	no predator	0.2889	7	196	17	9.1	13.23873
G. jazdzewski	no predator	0.2889	7	196	18	9.1	11.02493
G. jazdzewski	no predator	0.2889	7	196	19	9.09	13.38011
G. jazdzewski	no predator	0.2889	7	196	20	9.07	18.41867
G. jazdzewski	no predator	0.2889	7	196	21	9.09	13.75998
G. jazdzewski	no predator	0.2889	7	196	22	9.07	15.58139
G. jazdzewski	no predator	0.2889	7	196	23	9.07	16.08146
G. jazdzewski	no predator	0.2889	7	196	24	9.07	6.736808

Gammarid species	treatment	weight [g]	pre-exposure time	trial	time [min.]	oxygen level [mg/l]	activity
G. jazdzewski	no predator	0.2889	7	196	25	9.06	11.06493
G. jazdzewski	no predator	0.2889	7	196	26	9.05	11.29833
G. jazdzewski	no predator	0.2889	7	196	27	9.04	13.49819
G. jazdzewski	no predator	0.2889	7	196	28	9.05	16.61989
G. jazdzewski	no predator	0.2889	7	196	29	9.05	13.48185
G. jazdzewski	no predator	0.2889	7	196	30	9.03	10.26002
G. jazdzewski	no predator	0.2877	7	197	1	9.12	23.62006
G. jazdzewski	no predator	0.2877	7	197	2	9.12	16.95934
G. jazdzewski	no predator	0.2877	7	197	3	9.14	7.660292
G. jazdzewski	no predator	0.2877	7	197	4	9.14	5.080851
G. jazdzewski	no predator	0.2877	7	197	5	9.12	14.33907
G. jazdzewski	no predator	0.2877	7	197	6	9.13	14.49937
G. jazdzewski	no predator	0.2877	7	197	7	9.13	13.29987
G. jazdzewski	no predator	0.2877	7	197	8	9.14	17.22182
G. jazdzewski	no predator	0.2877	7	197	9	9.14	17.06145
G. jazdzewski	no predator	0.2877	7	197	10	9.12	13.34173
G. jazdzewski	no predator	0.2877	7	197	11	9.09	15.89684
G. jazdzewski	no predator	0.2877	7	197	12	9.11	9.039142
G. jazdzewski	no predator	0.2877	7	197	13	9.1	9.099976
G. jazdzewski	no predator	0.2877	7	197	14	9.1	14.77991
G. jazdzewski	no predator	0.2877	7	197	15	9.08	10.63887
G. jazdzewski	no predator	0.2877	7	197	16	9.08	7.899915
G. jazdzewski	no predator	0.2877	7	197	17	9.08	7.803522
G. jazdzewski	no predator	0.2877	7	197	18	9.08	12.89878
G. jazdzewski	no predator	0.2877	7	197	19	9.09	5.717419
G. jazdzewski	no predator	0.2877	7	197	20	9.06	6.479929
G. jazdzewski	no predator	0.2877	7	197	21	9.05	11.78132
G. jazdzewski	no predator	0.2877	7	197	22	9.07	9.662963
G. jazdzewski	no predator	0.2877	7	197	23	9.05	7.618546
G. jazdzewski	no predator	0.2877	7	197	24	9.04	1.419986
G. jazdzewski	no predator	0.2877	7	197	25	9.03	6.701614
G. jazdzewski	no predator	0.2877	7	197	26	9.02	10.60518
G. jazdzewski	no predator	0.2877	7	197	27	9.03	10.89294
G. jazdzewski	no predator	0.2877	7	197	28	9.03	2.839972
G. jazdzewski	no predator	0.2877	7	197	29	9.01	5.781864
G. jazdzewski	no predator	0.2877	7	197	30	9.01	6.43802
G. jazdzewski	no predator	0.1965	7	198	1	9.08	0.97999
G. jazdzewski	no predator	0.1965	7	198	2	9.08	3.240268
G. jazdzewski	no predator	0.1965	7	198	3	9.08	5.160365
G. jazdzewski	no predator	0.1965	7	198	4	9.09	5.560072
G. jazdzewski	no predator	0.1965	7	198	5	9.09	6.699582
G. jazdzewski	no predator	0.1965	7	198	6	9.08	3.461645
G. jazdzewski	no predator	0.1965	7	198	7	9.09	8.958348
G. jazdzewski	no predator	0.1965	7	198	8	9.08	12.25994
G. jazdzewski	no predator	0.1965	7	198	9	9.09	11.30067
G. jazdzewski	no predator	0.1965	7	198	10	9.09	1.879261
G. jazdzewski	no predator	0.1965	7	198	11	9.08	5.279951
G. jazdzewski	no predator	0.1965	7	198	12	9.09	2.539974

Gammarid species	treatment	weight [g]	pre-exposure time	trial	time [min.]	oxygen level [mg/l]	activity
G. jazdzewski	no predator	0.1965	7	198	13	9.08	2.340937
G. jazdzewski	no predator	0.1965	7	198	14	9.08	3.320023
G. jazdzewski	no predator	0.1965	7	198	15	9.1	3.042191
G. jazdzewski	no predator	0.1965	7	198	16	9.08	4.336717
G. jazdzewski	no predator	0.1965	7	198	17	9.11	3.621168
G. jazdzewski	no predator	0.1965	7	198	18	9.09	2.2013
G. jazdzewski	no predator	0.1965	7	198	19	9.09	3.797446
G. jazdzewski	no predator	0.1965	7	198	20	9.08	0.419996
G. jazdzewski	no predator	0.1965	7	198	21	9.08	2.159978
G. jazdzewski	no predator	0.1965	7	198	22	9.08	0.499995
G. jazdzewski	no predator	0.1965	7	198	23	9.08	1.699984
G. jazdzewski	no predator	0.1965	7	198	24	9.08	0.02
G. jazdzewski	no predator	0.1965	7	198	25	9.09	0.119999
G. jazdzewski	no predator	0.1965	7	198	26	9.1	0
G. jazdzewski	no predator	0.1965	7	198	27	9.07	0.119999
G. jazdzewski	no predator	0.1965	7	198	28	9.09	0.279997
G. jazdzewski	no predator	0.1965	7	198	29	9.08	0.059999
G. jazdzewski	no predator	0.1965	7	198	30	9.08	0
G. jazdzewski	no predator	0.2584	7	199	1	9.03	11.53917
G. jazdzewski	no predator	0.2584	7	199	2	9.08	9.819905
G. jazdzewski	no predator	0.2584	7	199	3	9.08	17.36091
G. jazdzewski	no predator	0.2584	7	199	4	9.08	13.09957
G. jazdzewski	no predator	0.2584	7	199	5	9.07	15.70039
G. jazdzewski	no predator	0.2584	7	199	6	9.06	17.63995
G. jazdzewski	no predator	0.2584	7	199	7	9.05	12.5606
G. jazdzewski	no predator	0.2584	7	199	8	9.06	17.11802
G. jazdzewski	no predator	0.2584	7	199	9	9.05	12.3806
G. jazdzewski	no predator	0.2584	7	199	10	9.05	12.44149
G. jazdzewski	no predator	0.2584	7	199	11	9.02	14.37836
G. jazdzewski	no predator	0.2584	7	199	12	9.02	8.620875
G. jazdzewski	no predator	0.2584	7	199	13	9.01	9.080039
G. jazdzewski	no predator	0.2584	7	199	14	9.03	10.89893
G. jazdzewski	no predator	0.2584	7	199	15	9.01	11.72205
G. jazdzewski	no predator	0.2584	7	199	16	8.99	24.96438
G. jazdzewski	no predator	0.2584	7	199	17	9	18.8753
G. jazdzewski	no predator	0.2584	7	199	18	8.98	13.36119
G. jazdzewski	no predator	0.2584	7	199	19	8.97	13.96262
G. jazdzewski	no predator	0.2584	7	199	20	8.96	9.317393
G. jazdzewski	no predator	0.2584	7	199	21	8.96	13.54143
G. jazdzewski	no predator	0.2584	7	199	22	8.94	10.15702
G. jazdzewski	no predator	0.2584	7	199	23	8.93	5.259949
G. jazdzewski	no predator	0.2584	7	199	24	8.92	6.999927
G. jazdzewski	no predator	0.2584	7	199	25	8.93	10.38326
G. jazdzewski	no predator	0.2584	7	199	26	8.92	5.298323
G. jazdzewski	no predator	0.2584	7	199	27	8.91	3.898222
G. jazdzewski	no predator	0.2584	7	199	28	8.92	5.519949
G. jazdzewski	no predator	0.2584	7	199	29	8.88	10.83989
G. jazdzewski	no predator	0.2584	7	199	30	8.88	15.15985

Gammarid species	treatment	weight [g]	pre-exposure time	trial	time [min.]	oxygen level [mg/l]	activity
G. jazdzewski	no predator	0.2534	7	200	1	9.11	26.70081
G. jazdzewski	no predator	0.2534	7	200	2	9.12	20.25957
G. jazdzewski	no predator	0.2534	7	200	3	9.13	18.42006
G. jazdzewski	no predator	0.2534	7	200	4	9.12	24.38042
G. jazdzewski	no predator	0.2534	7	200	5	9.13	23.72
G. jazdzewski	no predator	0.2534	7	200	6	9.11	23.62001
G. jazdzewski	no predator	0.2534	7	200	7	9.11	22.05883
G. jazdzewski	no predator	0.2534	7	200	8	9.11	19.79927
G. jazdzewski	no predator	0.2534	7	200	9	9.1	17.7806
G. jazdzewski	no predator	0.2534	7	200	10	9.1	11.07923
G. jazdzewski	no predator	0.2534	7	200	11	9.08	12.59993
G. jazdzewski	no predator	0.2534	7	200	12	9.08	14.94082
G. jazdzewski	no predator	0.2534	7	200	13	9.07	15.37901
G. jazdzewski	no predator	0.2534	7	200	14	9.07	8.338965
G. jazdzewski	no predator	0.2534	7	200	15	9.06	10.12099
G. jazdzewski	no predator	0.2534	7	200	16	9.05	10.61882
G. jazdzewski	no predator	0.2534	7	200	17	9.04	10.82349
G. jazdzewski	no predator	0.2534	7	200	18	9.04	6.679995
G. jazdzewski	no predator	0.2534	7	200	19	9.03	12.78125
G. jazdzewski	no predator	0.2534	7	200	20	9.02	8.778656
G. jazdzewski	no predator	0.2534	7	200	21	9.01	13.4257
G. jazdzewski	no predator	0.2534	7	200	22	9.01	11.49268
G. jazdzewski	no predator	0.2534	7	200	23	9.02	6.839932
G. jazdzewski	no predator	0.2534	7	200	24	8.99	7.119931
G. jazdzewski	no predator	0.2534	7	200	25	9.01	8.099925
G. jazdzewski	no predator	0.2534	7	200	26	9	5.281691
G. jazdzewski	no predator	0.2534	7	200	27	8.98	10.16176
G. jazdzewski	no predator	0.2534	7	200	28	8.99	5.001867
G. jazdzewski	no predator	0.2534	7	200	29	8.96	13.00384
G. jazdzewski	no predator	0.2534	7	200	30	8.96	14.82003
G. jazdzewski	perch	0.1286	7	201	1	8.66	2.299981
G. jazdzewski	perch	0.1286	7	201	2	8.63	0.519995
G. jazdzewski	perch	0.1286	7	201	3	8.62	0.799992
G. jazdzewski	perch	0.1286	7	201	4	8.61	0.159999
G. jazdzewski	perch	0.1286	7	201	5	8.59	0.159998
G. jazdzewski	perch	0.1286	7	201	6	8.58	0.319997
G. jazdzewski	perch	0.1286	7	201	7	8.57	0.04
G. jazdzewski	perch	0.1286	7	201	8	8.56	0
G. jazdzewski	perch	0.1286	7	201	9	8.57	0.079999
G. jazdzewski	perch	0.1286	7	201	10	8.55	0.95999
G. jazdzewski	perch	0.1286	7	201	11	8.54	0.339997
G. jazdzewski	perch	0.1286	7	201	12	8.53	1.119989
G. jazdzewski	perch	0.1286	7	201	13	8.53	0.159998
G. jazdzewski	perch	0.1286	7	201	14	8.52	0.02
G. jazdzewski	perch	0.1286	7	201	15	8.53	0
G. jazdzewski	perch	0.1286	7	201	16	8.51	0
G. jazdzewski	perch	0.1286	7	201	17	8.51	0.419996
G. jazdzewski	perch	0.1286	7	201	18	8.48	0.079999

Gammarid species	treatment	weight [g]	pre-exposure time	trial	time [min.]	oxygen level [mg/l]	activity
G. jazdzewski	perch	0.1286	7	201	19	8.47	0.579994
G. jazdzewski	perch	0.1286	7	201	20	8.47	0.04
G. jazdzewski	perch	0.1286	7	201	21	8.46	0.04
G. jazdzewski	perch	0.1286	7	201	22	8.44	0
G. jazdzewski	perch	0.1286	7	201	23	8.42	0
G. jazdzewski	perch	0.1286	7	201	24	8.4	0.099999
G. jazdzewski	perch	0.1286	7	201	25	8.38	0
G. jazdzewski	perch	0.1286	7	201	26	8.37	0.199998
G. jazdzewski	perch	0.1286	7	201	27	8.37	0.02
G. jazdzewski	perch	0.1286	7	201	28	8.36	0.379996
G. jazdzewski	perch	0.1286	7	201	29	8.35	0.079999
G. jazdzewski	perch	0.1286	7	201	30	8.35	0.379996
G. jazdzewski	perch	0.1613	7	202	1	8.47	2.039979
G. jazdzewski	perch	0.1613	7	202	2	8.45	1.499985
G. jazdzewski	perch	0.1613	7	202	3	8.46	3.10033
G. jazdzewski	perch	0.1613	7	202	4	8.46	5.199592
G. jazdzewski	perch	0.1613	7	202	5	8.45	1.519985
G. jazdzewski	perch	0.1613	7	202	6	8.45	0.91999
G. jazdzewski	perch	0.1613	7	202	7	8.45	0.139999
G. jazdzewski	perch	0.1613	7	202	8	8.43	0.179998
G. jazdzewski	perch	0.1613	7	202	9	8.43	0.339996
G. jazdzewski	perch	0.1613	7	202	10	8.42	0
G. jazdzewski	perch	0.1613	7	202	11	8.43	0.81999
G. jazdzewski	perch	0.1613	7	202	12	8.41	0.659993
G. jazdzewski	perch	0.1613	7	202	13	8.48	0.379996
G. jazdzewski	perch	0.1613	7	202	14	8.49	0.519995
G. jazdzewski	perch	0.1613	7	202	15	8.48	0.159998
G. jazdzewski	perch	0.1613	7	202	16	8.49	0.199998
G. jazdzewski	perch	0.1613	7	202	17	8.49	0
G. jazdzewski	perch	0.1613	7	202	18	8.47	0.079999
G. jazdzewski	perch	0.1613	7	202	19	8.48	0.179998
G. jazdzewski	perch	0.1613	7	202	20	8.46	0.139999
G. jazdzewski	perch	0.1613	7	202	21	8.44	1.639984
G. jazdzewski	perch	0.1613	7	202	22	8.39	0.04
G. jazdzewski	perch	0.1613	7	202	23	8.41	0.079999
G. jazdzewski	perch	0.1613	7	202	24	8.4	0
G. jazdzewski	perch	0.1613	7	202	25	8.41	0
G. jazdzewski	perch	0.1613	7	202	26	8.44	0.079999
G. jazdzewski	perch	0.1613	7	202	27	8.41	0.219998
G. jazdzewski	perch	0.1613	7	202	28	8.4	0
G. jazdzewski	perch	0.1613	7	202	29	8.37	0
G. jazdzewski	perch	0.1613	7	202	30	8.38	0
G. jazdzewski	perch	0.2709	7	203	1	8.27	0.759993
G. jazdzewski	perch	0.2709	7	203	2	8.3	1.619983
G. jazdzewski	perch	0.2709	7	203	3	8.28	0.599994
G. jazdzewski	perch	0.2709	7	203	4	8.26	3.799963
G. jazdzewski	perch	0.2709	7	203	5	8.27	1.400466
G. jazdzewski	perch	0.2709	7	203	6	8.25	1.339507

Gammarid species	treatment	weight [g]	pre-exposure time	trial	time [min.]	oxygen level [mg/l]	activity
G. jazdzewski	perch	0.2709	7	203	7	8.25	4.821754
G. jazdzewski	perch	0.2709	7	203	8	8.24	2.098178
G. jazdzewski	perch	0.2709	7	203	9	8.23	0.159999
G. jazdzewski	perch	0.2709	7	203	10	8.25	0.819992
G. jazdzewski	perch	0.2709	7	203	11	8.22	1.499985
G. jazdzewski	perch	0.2709	7	203	12	8.21	1.219988
G. jazdzewski	perch	0.2709	7	203	13	8.17	0.679993
G. jazdzewski	perch	0.2709	7	203	14	8.17	0.619994
G. jazdzewski	perch	0.2709	7	203	15	8.14	0.701073
G. jazdzewski	perch	0.2709	7	203	16	8.16	0.638914
G. jazdzewski	perch	0.2709	7	203	17	8.15	0.93999
G. jazdzewski	perch	0.2709	7	203	18	8.12	0.519994
G. jazdzewski	perch	0.2709	7	203	19	8.13	2.099979
G. jazdzewski	perch	0.2709	7	203	20	8.13	3.619962
G. jazdzewski	perch	0.2709	7	203	21	8.11	0.339996
G. jazdzewski	perch	0.2709	7	203	22	8.1	0
G. jazdzewski	perch	0.2709	7	203	23	8.1	0.04
G. jazdzewski	perch	0.2709	7	203	24	8.09	0.179998
G. jazdzewski	perch	0.2709	7	203	25	8.07	0.079999
G. jazdzewski	perch	0.2709	7	203	26	8.06	0.179998
G. jazdzewski	perch	0.2709	7	203	27	8.05	0.159998
G. jazdzewski	perch	0.2709	7	203	28	8.04	0.119999
G. jazdzewski	perch	0.2709	7	203	29	8.04	0.061919
G. jazdzewski	perch	0.2709	7	203	30	8.02	0.639994
G. jazdzewski	perch	0.1687	7	204	1	8.54	11.15982
G. jazdzewski	perch	0.1687	7	204	2	8.52	7.819745
G. jazdzewski	perch	0.1687	7	204	3	8.5	1.999981
G. jazdzewski	perch	0.1687	7	204	4	8.49	5.662048
G. jazdzewski	perch	0.1687	7	204	5	8.5	24.13958
G. jazdzewski	perch	0.1687	7	204	6	8.47	9.97852
G. jazdzewski	perch	0.1687	7	204	7	8.46	1.879981
G. jazdzewski	perch	0.1687	7	204	8	8.44	2.439975
G. jazdzewski	perch	0.1687	7	204	9	8.44	8.759917
G. jazdzewski	perch	0.1687	7	204	10	8.44	5.579946
G. jazdzewski	perch	0.1687	7	204	11	8.44	2.679974
G. jazdzewski	perch	0.1687	7	204	12	8.42	3.863561
G. jazdzewski	perch	0.1687	7	204	13	8.41	11.02002
G. jazdzewski	perch	0.1687	7	204	14	8.41	10.319
G. jazdzewski	perch	0.1687	7	204	15	8.4	9.302058
G. jazdzewski	perch	0.1687	7	204	16	8.4	5.977785
G. jazdzewski	perch	0.1687	7	204	17	8.39	3.461171
G. jazdzewski	perch	0.1687	7	204	18	8.36	7.461242
G. jazdzewski	perch	0.1687	7	204	19	8.34	7.138738
G. jazdzewski	perch	0.1687	7	204	20	8.34	7.441371
G. jazdzewski	perch	0.1687	7	204	21	8.32	2.398596
G. jazdzewski	perch	0.1687	7	204	22	8.33	3.999959
G. jazdzewski	perch	0.1687	7	204	23	8.31	4.799954
G. jazdzewski	perch	0.1687	7	204	24	8.31	4.119959

Gammarid species	treatment	weight [g]	pre-exposure time	trial	time [min.]	oxygen level [mg/l]	activity
G. jazdzewski	perch	0.1687	7	204	25	8.31	0.101679
G. jazdzewski	perch	0.1687	7	204	26	8.3	2.178298
G. jazdzewski	perch	0.1687	7	204	27	8.28	2.501775
G. jazdzewski	perch	0.1687	7	204	28	8.28	1.298187
G. jazdzewski	perch	0.1687	7	204	29	8.28	0.941911
G. jazdzewski	perch	0.1687	7	204	30	8.28	4.958033
G. jazdzewski	perch	0.2764	7	205	1	8.51	16.55996
G. jazdzewski	perch	0.2764	7	205	2	8.48	9.299736
G. jazdzewski	perch	0.2764	7	205	3	8.46	19.00052
G. jazdzewski	perch	0.2764	7	205	4	8.44	9.540035
G. jazdzewski	perch	0.2764	7	205	5	8.41	12.48001
G. jazdzewski	perch	0.2764	7	205	6	8.43	14.0416
G. jazdzewski	perch	0.2764	7	205	7	8.4	8.997814
G. jazdzewski	perch	0.2764	7	205	8	8.39	7.561249
G. jazdzewski	perch	0.2764	7	205	9	8.37	17.07923
G. jazdzewski	perch	0.2764	7	205	10	8.35	11.44073
G. jazdzewski	perch	0.2764	7	205	11	8.34	8.281719
G. jazdzewski	perch	0.2764	7	205	12	8.33	11.49827
G. jazdzewski	perch	0.2764	7	205	13	8.32	2.539078
G. jazdzewski	perch	0.2764	7	205	14	8.33	0.859992
G. jazdzewski	perch	0.2764	7	205	15	8.34	6.319933
G. jazdzewski	perch	0.2764	7	205	16	8.33	8.163342
G. jazdzewski	perch	0.2764	7	205	17	8.31	10.33888
G. jazdzewski	perch	0.2764	7	205	18	8.32	4.301278
G. jazdzewski	perch	0.2764	7	205	19	8.31	8.500037
G. jazdzewski	perch	0.2764	7	205	20	8.3	12.26132
G. jazdzewski	perch	0.2764	7	205	21	8.3	8.697151
G. jazdzewski	perch	0.2764	7	205	22	8.29	8.719912
G. jazdzewski	perch	0.2764	7	205	23	8.29	7.043058
G. jazdzewski	perch	0.2764	7	205	24	8.27	6.676809
G. jazdzewski	perch	0.2764	7	205	25	8.23	1.099988
G. jazdzewski	perch	0.2764	7	205	26	8.2	8.886879
G. jazdzewski	perch	0.2764	7	205	27	8.21	2.876611
G. jazdzewski	perch	0.2764	7	205	28	8.22	3.456365
G. jazdzewski	perch	0.2764	7	205	29	8.22	1.919981
G. jazdzewski	perch	0.2764	7	205	30	8.21	4.961938
G. jazdzewski	perch	0.2109	7	206	1	8.25	1.379987
G. jazdzewski	perch	0.2109	7	206	2	8.24	5.88024
G. jazdzewski	perch	0.2109	7	206	3	8.23	1.859682
G. jazdzewski	perch	0.2109	7	206	4	8.24	0.379996
G. jazdzewski	perch	0.2109	7	206	5	8.23	0.439995
G. jazdzewski	perch	0.2109	7	206	6	8.25	0.299997
G. jazdzewski	perch	0.2109	7	206	7	8.23	0.02
G. jazdzewski	perch	0.2109	7	206	8	8.23	0.239997
G. jazdzewski	perch	0.2109	7	206	9	8.21	0.439995
G. jazdzewski	perch	0.2109	7	206	10	8.18	0
G. jazdzewski	perch	0.2109	7	206	11	8.17	0.179998
G. jazdzewski	perch	0.2109	7	206	12	8.16	0

Gammarid species	treatment	weight [g]	pre-exposure time	trial	time [min.]	oxygen level [mg/l]	activity
G. jazdzewski	perch	0.2109	7	206	13	8.14	0.199998
G. jazdzewski	perch	0.2109	7	206	14	8.14	0.059999
G. jazdzewski	perch	0.2109	7	206	15	8.12	0.381076
G. jazdzewski	perch	0.2109	7	206	16	8.11	0.439995
G. jazdzewski	perch	0.2109	7	206	17	8.12	0.559995
G. jazdzewski	perch	0.2109	7	206	18	8.1	3.399961
G. jazdzewski	perch	0.2109	7	206	19	8.07	0.579994
G. jazdzewski	perch	0.2109	7	206	20	8.07	0.379996
G. jazdzewski	perch	0.2109	7	206	21	8.06	0.279997
G. jazdzewski	perch	0.2109	7	206	22	8.07	0.079999
G. jazdzewski	perch	0.2109	7	206	23	8.05	0.279997
G. jazdzewski	perch	0.2109	7	206	24	8.04	0.499996
G. jazdzewski	perch	0.2109	7	206	25	8.01	0.139999
G. jazdzewski	perch	0.2109	7	206	26	8.01	0.059999
G. jazdzewski	perch	0.2109	7	206	27	8	0.179998
G. jazdzewski	perch	0.2109	7	206	28	8	0.141859
G. jazdzewski	perch	0.2109	7	206	29	8	0.198138
G. jazdzewski	perch	0.2109	7	206	30	7.98	0.439996
G. jazdzewski	perch	0.3309	7	213	1	9.13	2.760212
G. jazdzewski	perch	0.3309	7	213	2	9.17	5.040253
G. jazdzewski	perch	0.3309	7	213	3	9.15	6.919629
G. jazdzewski	perch	0.3309	7	213	4	9.14	5.060794
G. jazdzewski	perch	0.3309	7	213	5	9.13	4.059121
G. jazdzewski	perch	0.3309	7	213	6	9.12	5.260497
G. jazdzewski	perch	0.3309	7	213	7	9.11	7.799979
G. jazdzewski	perch	0.3309	7	213	8	9.12	10.82254
G. jazdzewski	perch	0.3309	7	213	9	9.12	8.017997
G. jazdzewski	perch	0.3309	7	213	10	9.12	6.700777
G. jazdzewski	perch	0.3309	7	213	11	9.13	6.060836
G. jazdzewski	perch	0.3309	7	213	12	9.13	9.098227
G. jazdzewski	perch	0.3309	7	213	13	9.12	2.524774
G. jazdzewski	perch	0.3309	7	213	14	9.11	3.115168
G. jazdzewski	perch	0.3309	7	213	15	9.1	3.339966
G. jazdzewski	perch	0.3309	7	213	16	9.1	1.619984
G. jazdzewski	perch	0.3309	7	213	17	9.1	0.803592
G. jazdzewski	perch	0.3309	7	213	18	9.1	2.197579
G. jazdzewski	perch	0.3309	7	213	19	9.09	3.099973
G. jazdzewski	perch	0.3309	7	213	20	9.08	1.639984
G. jazdzewski	perch	0.3309	7	213	21	9.08	5.499944
G. jazdzewski	perch	0.3309	7	213	22	9.08	1.359986
G. jazdzewski	perch	0.3309	7	213	23	9.08	1.899981
G. jazdzewski	perch	0.3309	7	213	24	9.07	0.97999
G. jazdzewski	perch	0.3309	7	213	25	9.08	2.379978
G. jazdzewski	perch	0.3309	7	213	26	9.05	3.07997
G. jazdzewski	perch	0.3309	7	213	27	9.05	1.599984
G. jazdzewski	perch	0.3309	7	213	28	9.05	1.259987
G. jazdzewski	perch	0.3309	7	213	29	9.04	2.039979
G. jazdzewski	perch	0.3309	7	213	30	9.03	2.901951

Gammarid species	treatment	weight [g]	pre-exposure time	trial	time [min.]	oxygen level [mg/l]	activity
G. jazdzewski	perch	0.2894	7	214	1	9.09	6.639569
G. jazdzewski	perch	0.2894	7	214	2	9.08	5.659948
G. jazdzewski	perch	0.2894	7	214	3	9.09	5.760307
G. jazdzewski	perch	0.2894	7	214	4	9.07	1.819622
G. jazdzewski	perch	0.2894	7	214	5	9.07	2.020461
G. jazdzewski	perch	0.2894	7	214	6	9.06	6.779988
G. jazdzewski	perch	0.2894	7	214	7	9.07	8.721181
G. jazdzewski	perch	0.2894	7	214	8	9.06	5.758807
G. jazdzewski	perch	0.2894	7	214	9	9.04	2.640033
G. jazdzewski	perch	0.2894	7	214	10	9.05	5.11923
G. jazdzewski	perch	0.2894	7	214	11	9.02	2.340816
G. jazdzewski	perch	0.2894	7	214	12	9.06	7.880883
G. jazdzewski	perch	0.2894	7	214	13	9.04	2.439076
G. jazdzewski	perch	0.2894	7	214	14	9.04	2.93997
G. jazdzewski	perch	0.2894	7	214	15	9.04	3.581046
G. jazdzewski	perch	0.2894	7	214	16	9.04	1.458906
G. jazdzewski	perch	0.2894	7	214	17	9.05	3.179968
G. jazdzewski	perch	0.2894	7	214	18	9.01	0.879991
G. jazdzewski	perch	0.2894	7	214	19	9.04	1.219988
G. jazdzewski	perch	0.2894	7	214	20	9.04	0.719993
G. jazdzewski	perch	0.2894	7	214	21	9.03	4.801394
G. jazdzewski	perch	0.2894	7	214	22	9.01	1.419986
G. jazdzewski	perch	0.2894	7	214	23	9.02	2.719972
G. jazdzewski	perch	0.2894	7	214	24	9	4.539955
G. jazdzewski	perch	0.2894	7	214	25	9.01	5.079953
G. jazdzewski	perch	0.2894	7	214	26	9.01	6.019939
G. jazdzewski	perch	0.2894	7	214	27	9	0.759992
G. jazdzewski	perch	0.2894	7	214	28	9	0
G. jazdzewski	perch	0.2894	7	214	29	9	0.379996
G. jazdzewski	perch	0.2894	7	214	30	8.99	0.439996
G. jazdzewski	perch	0.3435	7	215	1	9.08	6.659995
G. jazdzewski	perch	0.3435	7	215	2	9.1	6.080293
G. jazdzewski	perch	0.3435	7	215	3	9.09	4.299358
G. jazdzewski	perch	0.3435	7	215	4	9.08	4.740792
G. jazdzewski	perch	0.3435	7	215	5	9.08	3.520086
G. jazdzewski	perch	0.3435	7	215	6	9.07	3.559965
G. jazdzewski	perch	0.3435	7	215	7	9.06	0.319997
G. jazdzewski	perch	0.3435	7	215	8	9.07	0.479995
G. jazdzewski	perch	0.3435	7	215	9	9.07	1.459985
G. jazdzewski	perch	0.3435	7	215	10	9.05	0.919991
G. jazdzewski	perch	0.3435	7	215	11	9.05	2.479975
G. jazdzewski	perch	0.3435	7	215	12	9.04	0.639993
G. jazdzewski	perch	0.3435	7	215	13	9.04	1.379987
G. jazdzewski	perch	0.3435	7	215	14	9.04	1.739983
G. jazdzewski	perch	0.3435	7	215	15	9.04	0.639993
G. jazdzewski	perch	0.3435	7	215	16	9.04	2.819973
G. jazdzewski	perch	0.3435	7	215	17	9.03	0.539995
G. jazdzewski	perch	0.3435	7	215	18	9.02	0.699993

Gammarid species	treatment	weight [g]	pre-exposure time	trial	time [min.]	oxygen level [mg/l]	activity
G. jazdzewski	perch	0.3435	7	215	19	9.02	1.359986
G. jazdzewski	perch	0.3435	7	215	20	9.01	0.761373
G. jazdzewski	perch	0.3435	7	215	21	9.01	1.198608
G. jazdzewski	perch	0.3435	7	215	22	9.01	0.381497
G. jazdzewski	perch	0.3435	7	215	23	9	2.139978
G. jazdzewski	perch	0.3435	7	215	24	9.01	1.521604
G. jazdzewski	perch	0.3435	7	215	25	9.01	1.638365
G. jazdzewski	perch	0.3435	7	215	26	9	2.439974
G. jazdzewski	perch	0.3435	7	215	27	9	4.441757
G. jazdzewski	perch	0.3435	7	215	28	8.99	3.418166
G. jazdzewski	perch	0.3435	7	215	29	8.97	3.01997
G. jazdzewski	perch	0.3435	7	215	30	8.97	1.839982
G. jazdzewski	perch	0.3064	7	216	1	9.05	16.05978
G. jazdzewski	perch	0.3064	7	216	2	9.08	17.08049
G. jazdzewski	perch	0.3064	7	216	3	9.09	16.2593
G. jazdzewski	perch	0.3064	7	216	4	9.09	17.2603
G. jazdzewski	perch	0.3064	7	216	5	9.09	16.10044
G. jazdzewski	perch	0.3064	7	216	6	9.09	15.16055
G. jazdzewski	perch	0.3064	7	216	7	9.1	18.15885
G. jazdzewski	perch	0.3064	7	216	8	9.09	19.21928
G. jazdzewski	perch	0.3064	7	216	9	9.1	17.42198
G. jazdzewski	perch	0.3064	7	216	10	9.09	16.62002
G. jazdzewski	perch	0.3064	7	216	11	9.07	18.90167
G. jazdzewski	perch	0.3064	7	216	12	9.08	18.36457
G. jazdzewski	perch	0.3064	7	216	13	9.06	28.19924
G. jazdzewski	perch	0.3064	7	216	14	9.05	21.51708
G. jazdzewski	perch	0.3064	7	216	15	9.05	18.86209
G. jazdzewski	perch	0.3064	7	216	16	9.03	19.48005
G. jazdzewski	perch	0.3064	7	216	17	9.02	19.41644
G. jazdzewski	perch	0.3064	7	216	18	9.03	17.32113
G. jazdzewski	perch	0.3064	7	216	19	9.02	20.38121
G. jazdzewski	perch	0.3064	7	216	20	9	24.80126
G. jazdzewski	perch	0.3064	7	216	21	8.99	10.47863
G. jazdzewski	perch	0.3064	7	216	22	8.98	8.063046
G. jazdzewski	perch	0.3064	7	216	23	8.95	11.95856
G. jazdzewski	perch	0.3064	7	216	24	8.96	13.31837
G. jazdzewski	perch	0.3064	7	216	25	8.95	11.74666
G. jazdzewski	perch	0.3064	7	216	26	8.93	10.25492
G. jazdzewski	perch	0.3064	7	216	27	8.91	7.699985
G. jazdzewski	perch	0.3064	7	216	28	8.89	6.983715
G. jazdzewski	perch	0.3064	7	216	29	8.89	9.403865
G. jazdzewski	perch	0.3064	7	216	30	8.89	10.41613
G. jazdzewski	perch	0.2415	7	217	1	8.98	6.839569
G. jazdzewski	perch	0.2415	7	217	2	8.99	4.781156
G. jazdzewski	perch	0.2415	7	217	3	8.99	6.478728
G. jazdzewski	perch	0.2415	7	217	4	9.01	6.380342
G. jazdzewski	perch	0.2415	7	217	5	9	4.779532
G. jazdzewski	perch	0.2415	7	217	6	9	7.639926

Gammarid species	treatment	weight [g]	pre-exposure time	trial	time [min.]	oxygen level [mg/l]	activity
G. jazdzewski	perch	0.2415	7	217	7	9	4.639955
G. jazdzewski	perch	0.2415	7	217	8	9.01	5.039951
G. jazdzewski	perch	0.2415	7	217	9	9.01	3.680685
G. jazdzewski	perch	0.2415	7	217	10	9.01	4.959228
G. jazdzewski	perch	0.2415	7	217	11	9.01	0.479995
G. jazdzewski	perch	0.2415	7	217	12	9.01	0.840892
G. jazdzewski	perch	0.2415	7	217	13	9	0.179098
G. jazdzewski	perch	0.2415	7	217	14	9.01	0.399996
G. jazdzewski	perch	0.2415	7	217	15	9.02	6.019943
G. jazdzewski	perch	0.2415	7	217	16	9.01	3.639964
G. jazdzewski	perch	0.2415	7	217	17	9.01	5.321143
G. jazdzewski	perch	0.2415	7	217	18	9.01	3.978769
G. jazdzewski	perch	0.2415	7	217	19	8.99	3.05997
G. jazdzewski	perch	0.2415	7	217	20	9.01	0.579994
G. jazdzewski	perch	0.2415	7	217	21	9.01	0.079999
G. jazdzewski	perch	0.2415	7	217	22	9.01	0.119999
G. jazdzewski	perch	0.2415	7	217	23	9.01	0.399996
G. jazdzewski	perch	0.2415	7	217	24	9.01	0.04
G. jazdzewski	perch	0.2415	7	217	25	9.02	0.119999
G. jazdzewski	perch	0.2415	7	217	26	9.01	0.159998
G. jazdzewski	perch	0.2415	7	217	27	9	1.539985
G. jazdzewski	perch	0.2415	7	217	28	9	0.159998
G. jazdzewski	perch	0.2415	7	217	29	9	0
G. jazdzewski	perch	0.2415	7	217	30	8.99	0

Gammarid species	treatment	pre-exposure time	CAT activity [u]
D. villosus	no predator	0	96.39
D. villosus	no predator	0	69.71
D. villosus	no predator	0	84.70
D. villosus	no predator	0	109.98
D. villosus	no predator	0	66.74
D. villosus	no predator	0	62.27
D. villosus	no predator	0	105.60
D. villosus	no predator	0	95.38
D. villosus	no predator	0	66.47
D. villosus	no predator	0	85.23
D. villosus	perch	0	155.79
D. villosus	perch	0	119.37
D. villosus	perch	0	128.42
D. villosus	perch	0	70.49
D. villosus	perch	0	189.17
D. villosus	perch	0	127.49
D. villosus	perch	0	63.85
D. villosus	perch	0	189.25
D. villosus	perch	0	80.00
D. villosus	perch	0	53.74
D. villosus	no predator	1	152.37
D. villosus	no predator	1	133.65
D. villosus	no predator	1	108.41
D. villosus	no predator	1	176.44
D. villosus	no predator	1	239.35
D. villosus	no predator	1	157.79
D. villosus	no predator	1	153.73
D. villosus	no predator	1	121.13
D. villosus	no predator	1	201.33
D. villosus	no predator	1	164.23
D. villosus	no predator	1	101.81
D. villosus	no predator	1	174.12
D. villosus	perch	1	100.05
D. villosus	perch	1	138.60
D. villosus	perch	1	134.85
D. villosus	perch	1	92.59
D. villosus	perch	1	241.13
D. villosus	perch	1	216.80
D. villosus	perch	1	213.30
D. villosus	perch	1	210.38
D. villosus	perch	1	131.19
D. villosus	perch	1	123.13
D. villosus	perch	1	147.38
D. villosus	no predator	7	159.50
D. villosus	no predator	7	112.86
D. villosus	no predator	7	131.00
D. villosus	no predator	7	116.20
D. villosus	no predator	7	104.23

Gammarid species	treatment	pre-exposure time	CAT activity [u]
D. villosus	no predator	7	131.73
D. villosus	no predator	7	123.70
D. villosus	no predator	7	160.19
D. villosus	no predator	7	64.68
D. villosus	no predator	7	154.86
D. villosus	perch	7	117.83
D. villosus	perch	7	72.18
D. villosus	perch	7	182.91
D. villosus	perch	7	100.27
D. villosus	perch	7	127.33
D. villosus	perch	7	104.67
D. villosus	perch	7	119.68
D. villosus	perch	7	107.95
D. villosus	perch	7	44.55
D. villosus	perch	7	106.06
G. jazdzewskii	no predator	0	77.01
G. jazdzewskii	no predator	0	130.15
G. jazdzewskii	no predator	0	132.17
G. jazdzewskii	no predator	0	102.52
G. jazdzewskii	no predator	0	155.45
G. jazdzewskii	no predator	0	109.38
G. jazdzewskii	no predator	0	128.86
G. jazdzewskii	no predator	0	126.67
G. jazdzewskii	no predator	0	130.45
G. jazdzewskii	no predator	0	160.84
G. jazdzewskii	perch	0	253.58
G. jazdzewskii	perch	0	312.64
G. jazdzewskii	perch	0	313.96
G. jazdzewskii	perch	0	396.00
G. jazdzewskii	perch	0	276.23
G. jazdzewskii	perch	0	362.26
G. jazdzewskii	perch	0	366.67
G. jazdzewskii	perch	0	379.13
G. jazdzewskii	perch	0	297.54
G. jazdzewskii	perch	0	258.91
G. jazdzewskii	no predator	1	137.03
G. jazdzewskii	no predator	1	87.00
G. jazdzewskii	no predator	1	50.50
G. jazdzewskii	no predator	1	145.68
G. jazdzewskii	no predator	1	107.27
G. jazdzewskii	no predator	1	108.75
G. jazdzewskii	no predator	1	113.19
G. jazdzewskii	no predator	1	135.66
G. jazdzewskii	perch	1	92.21
G. jazdzewskii	perch	1	94.64
G. jazdzewskii	perch	1	91.17
G. jazdzewskii	perch	1	151.27
G. jazdzewskii	perch	1	112.99

Gammarid species	treatment	pre-exposure time	CAT activity [u]
G. jazdzewskii	perch	1	133.24
G. jazdzewskii	perch	1	123.20
G. jazdzewskii	perch	1	161.49
G. jazdzewskii	perch	1	139.77
G. jazdzewskii	perch	1	106.76
G. jazdzewskii	perch	1	137.30
G. jazdzewskii	no predator	7	199.38
G. jazdzewskii	no predator	7	178.67
G. jazdzewskii	no predator	7	94.35
G. jazdzewskii	no predator	7	88.71
G. jazdzewskii	no predator	7	110.65
G. jazdzewskii	no predator	7	75.49
G. jazdzewskii	no predator	7	47.55
G. jazdzewskii	no predator	7	76.03
G. jazdzewskii	no predator	7	21.94
G. jazdzewskii	no predator	7	75.31
G. jazdzewskii	perch	7	159.13
G. jazdzewskii	perch	7	159.82
G. jazdzewskii	perch	7	181.29
G. jazdzewskii	perch	7	101.64
G. jazdzewskii	perch	7	51.91
G. jazdzewskii	perch	7	30.90
G. jazdzewskii	perch	7	147.37
G. jazdzewskii	perch	7	93.10
G. jazdzewskii	perch	7	88.93

Gammarid species	treatment	pre-exposure time	Hsp70 [pg/ml]
D. villosus	no predator	0	536.66
D. villosus	no predator	0	550.89
D. villosus	no predator	0	577.97
D. villosus	no predator	0	407.96
D. villosus	no predator	0	511.76
D. villosus	no predator	0	735.11
D. villosus	no predator	0	561.70
D. villosus	no predator	0	482.50
D. villosus	no predator	0	1103.08
D. villosus	no predator	0	450.00
D. villosus	perch	0	692.97
D. villosus	perch	0	441.97
D. villosus	perch	0	430.43
D. villosus	perch	0	472.72
D. villosus	perch	0	557.99
D. villosus	perch	0	621.80
D. villosus	perch	0	470.59
D. villosus	perch	0	518.14
D. villosus	perch	0	366.35
D. villosus	perch	0	412.64
D. villosus	no predator	1	465.57
D. villosus	no predator	1	327.76
D. villosus	no predator	1	590.92
D. villosus	no predator	1	505.70
D. villosus	no predator	1	697.37
D. villosus	no predator	1	747.03
D. villosus	no predator	1	477.43
D. villosus	no predator	1	337.59
D. villosus	no predator	1	389.51
D. villosus	no predator	1	437.70
D. villosus	perch	1	537.86
D. villosus	perch	1	462.98
D. villosus	perch	1	449.21
D. villosus	perch	1	391.07
D. villosus	perch	1	502.88
D. villosus	perch	1	531.70
D. villosus	perch	1	544.90
D. villosus	perch	1	504.40
D. villosus	perch	1	339.96
D. villosus	perch	1	563.80
G. jazdzewskii	no predator	0	609.65
G. jazdzewskii	no predator	0	553.27
G. jazdzewskii	no predator	0	537.36
G. jazdzewskii	no predator	0	523.89
G. jazdzewskii	no predator	0	645.82
G. jazdzewskii	no predator	0	1309.54
G. jazdzewskii	no predator	0	544.76
G. jazdzewskii	no predator	0	602.26
G. jazdzewskii	no predator	0	640.00

Gammarid species	treatment	pre-exposure time	Hsp70 [pg/ml]
G. jazdzewskii	no predator	0	603.59
G. jazdzewskii	perch	0	1249.04
G. jazdzewskii	perch	0	1427.83
G. jazdzewskii	perch	0	2810.34
G. jazdzewskii	perch	0	2986.67
G. jazdzewskii	perch	0	816.15
G. jazdzewskii	perch	0	4404.67
G. jazdzewskii	perch	0	2236.78
G. jazdzewskii	perch	0	2660.47
G. jazdzewskii	perch	0	2152.10
G. jazdzewskii	perch	0	3788.79
G. jazdzewskii	no predator	1	1657.34
G. jazdzewskii	no predator	1	1209.81
G. jazdzewskii	no predator	1	880.08
G. jazdzewskii	no predator	1	644.82
G. jazdzewskii	no predator	1	1303.05
G. jazdzewskii	no predator	1	1138.85
G. jazdzewskii	no predator	1	846.52
G. jazdzewskii	no predator	1	577.00
G. jazdzewskii	no predator	1	913.25
G. jazdzewskii	no predator	1	812.69
G. jazdzewskii	perch	1	705.34
G. jazdzewskii	perch	1	622.86
G. jazdzewskii	perch	1	928.07
G. jazdzewskii	perch	1	867.34
G. jazdzewskii	perch	1	912.42
G. jazdzewskii	perch	1	1299.39
G. jazdzewskii	perch	1	1147.01
G. jazdzewskii	perch	1	1198.38

Gammarid species	treatment	pre-exposure time	TBARS [µmol/g wet mass]
D. villosus	no predator	0	4.81
D. villosus	no predator	0	9.85
D. villosus	no predator	0	7.98
D. villosus	no predator	0	8.23
D. villosus	no predator	0	1.81
D. villosus	no predator	0	10.13
D. villosus	no predator	0	8.15
D. villosus	no predator	0	7.44
D. villosus	no predator	0	10.41
D. villosus	no predator	0	8.81
D. villosus	perch	0	5.48
D. villosus	perch	0	6.48
D. villosus	perch	0	7.36
D. villosus	perch	0	4.81
D. villosus	perch	0	4.20
D. villosus	perch	0	5.84
D. villosus	perch	0	4.89
D. villosus	perch	0	4.66
D. villosus	perch	0	3.49
D. villosus	perch	0	3.99
D. villosus	no predator	1	7.36
D. villosus	no predator	1	5.85
D. villosus	no predator	1	4.17
D. villosus	no predator	1	7.38
D. villosus	no predator	1	4.60
D. villosus	no predator	1	6.75
D. villosus	no predator	1	9.06
D. villosus	no predator	1	7.01
D. villosus	no predator	1	4.58
D. villosus	no predator	1	6.84
D. villosus	no predator	1	5.19
D. villosus	no predator	1	4.50
D. villosus	perch	1	7.79
D. villosus	perch	1	10.01
D. villosus	perch	1	7.51
D. villosus	perch	1	5.21
D. villosus	perch	1	8.48
D. villosus	perch	1	4.45
D. villosus	perch	1	8.50
D. villosus	perch	1	5.05
D. villosus	perch	1	4.95
D. villosus	perch	1	6.86
D. villosus	perch	1	4.62
D. villosus	no predator	7	4.67
D. villosus	no predator	7	4.92
D. villosus	no predator	7	3.69
D. villosus	no predator	7	3.14
D. villosus	no predator	7	7.48
D. villosus	no predator	7	7.81

Gammarid species	treatment	pre-exposure time	TBARS [µmol/g wet mass]
D. villosus	no predator	7	8.71
D. villosus	no predator	7	6.87
D. villosus	no predator	7	1.79
D. villosus	no predator	7	8.44
D. villosus	perch	7	4.36
D. villosus	perch	7	5.77
D. villosus	perch	7	5.64
D. villosus	perch	7	2.86
D. villosus	perch	7	9.36
D. villosus	perch	7	8.68
D. villosus	perch	7	8.56
D. villosus	perch	7	9.09
D. villosus	perch	7	6.31
D. villosus	perch	7	7.69
G. jazdzewskii	no predator	0	2.68
G. jazdzewskii	no predator	0	5.37
G. jazdzewskii	no predator	0	1.64
G. jazdzewskii	no predator	0	5.28
G. jazdzewskii	no predator	0	3.47
G. jazdzewskii	no predator	0	1.98
G. jazdzewskii	no predator	0	6.81
G. jazdzewskii	no predator	0	5.40
G. jazdzewskii	no predator	0	0.38
G. jazdzewskii	no predator	0	6.07
G. jazdzewskii	perch	0	19.89
G. jazdzewskii	perch	0	10.08
G. jazdzewskii	perch	0	8.29
G. jazdzewskii	perch	0	41.03
G. jazdzewskii	perch	0	11.40
G. jazdzewskii	perch	0	13.92
G. jazdzewskii	perch	0	24.70
G. jazdzewskii	perch	0	24.03
G. jazdzewskii	perch	0	12.18
G. jazdzewskii	perch	0	17.41
G. jazdzewskii	no predator	1	12.91
G. jazdzewskii	no predator	1	8.12
G. jazdzewskii	no predator	1	15.86
G. jazdzewskii	no predator	1	5.73
G. jazdzewskii	no predator	1	4.23
G. jazdzewskii	no predator	1	13.94
G. jazdzewskii	no predator	1	5.68
G. jazdzewskii	no predator	1	4.87
G. jazdzewskii	no predator	1	13.96
G. jazdzewskii	no predator	1	6.13
G. jazdzewskii	perch	1	8.58
G. jazdzewskii	perch	1	4.43
G. jazdzewskii	perch	1	13.34
G. jazdzewskii	perch	1	3.22
G. jazdzewskii	perch	1	8.18

Gammarid species	treatment	pre-exposure time	TBARS [µmol/g wet mass]
G. jazdzewskii	perch	1	9.48
G. jazdzewskii	perch	1	16.38
G. jazdzewskii	perch	1	7.91
G. jazdzewskii	perch	1	14.89
G. jazdzewskii	perch	1	21.72
G. jazdzewskii	perch	1	5.05
G. jazdzewskii	perch	1	10.36
G. jazdzewskii	no predator	7	6.53
G. jazdzewskii	no predator	7	3.59
G. jazdzewskii	no predator	7	17.59
G. jazdzewskii	no predator	7	6.71
G. jazdzewskii	no predator	7	11.66
G. jazdzewskii	no predator	7	3.30
G. jazdzewskii	no predator	7	6.81
G. jazdzewskii	perch	7	6.67
G. jazdzewskii	perch	7	2.92
G. jazdzewskii	perch	7	7.22
G. jazdzewskii	perch	7	8.75
G. jazdzewskii	perch	7	1.71
G. jazdzewskii	perch	7	6.65
G. jazdzewskii	perch	7	6.37
G. jazdzewskii	perch	7	2.21
G. jazdzewskii	perch	7	4.77
G. jazdzewskii	perch	7	5.30
